# A Review of the Updated Pharmacophore for the Alpha 5 GABA(A) Benzodiazepine Receptor Model

**DOI:** 10.1155/2015/430248

**Published:** 2015-11-10

**Authors:** Terry Clayton, Michael M. Poe, Sundari Rallapalli, Poonam Biawat, Miroslav M. Savić, James K. Rowlett, George Gallos, Charles W. Emala, Catherine C. Kaczorowski, Douglas C. Stafford, Leggy A. Arnold, James M. Cook

**Affiliations:** ^1^Department of Chemistry and Biochemistry, University of Wisconsin-Milwaukee, Milwaukee, WI 53201, USA; ^2^Department of Pharmacology, Faculty of Pharmacy, University of Belgrade, Belgrade, Serbia; ^3^Department of Psychiatry and Human Behavior, University of Mississippi Medical Center, Jackson, MS 39216, USA; ^4^Department of Anesthesiology, Columbia University, New York, NY 10032, USA; ^5^Department of Anatomy and Neurobiology, University of Tennessee Health Science Center, Memphis, TN 38163, USA; ^6^Milwaukee Institute of Drug Discovery, University of Wisconsin-Milwaukee, Milwaukee, WI 53201, USA

## Abstract

An updated model of the GABA(A) benzodiazepine receptor pharmacophore of the *α*5-BzR/GABA(A) subtype has been constructed prompted by the synthesis of subtype selective ligands in light of the recent developments in both ligand synthesis, behavioral studies, and molecular modeling studies of the binding site itself. A number of BzR/GABA(A) *α*5 subtype selective compounds were synthesized, notably *α*5-subtype selective inverse agonist PWZ-029 (**1**) which is active in enhancing cognition in both rodents and primates. In addition, a chiral positive allosteric modulator (PAM), SH-053-2′F-R-CH_3_ (**2**), has been shown to reverse the deleterious effects in the MAM-model of schizophrenia as well as alleviate constriction in airway smooth muscle. Presented here is an updated model of the pharmacophore for *α*5*β*2*γ*2 Bz/GABA(A) receptors, including a rendering of PWZ-029 docked within the *α*5-binding pocket showing specific interactions of the molecule with the receptor. Differences in the included volume as compared to *α*1*β*2*γ*2, *α*2*β*2*γ*2, and *α*3*β*2*γ*2 will be illustrated for clarity. These new models enhance the ability to understand structural characteristics of ligands which act as agonists, antagonists, or inverse agonists at the Bz BS of GABA(A) receptors.

## 1. Introduction

The gamma-amino butyric acid A (GABA_A_) receptor is a heteropentameric chloride ion channel. This channel is generally made up of two *α*-subunits, two *β*-subunits, and a single *γ*-subunit arranged in an *αβαβγ* fashion. The GABA_A_ receptors (GABA_A_R) are responsible for a myriad of brain functions. Positive allosteric modulators (PAMs) and negative allosteric modulators (NAMs) act on the benzodiazepine (BZ) site of the GABA_A_R which can change the conformation of the receptor to inhibit or excite the neurons associated with the ion channel. To date, researchers have been unable to get an X-ray crystal structure of a functional Bz/GABA_A_R ion channel. Recently, Miller and Aricescu [1] have reported the crystal structure of a homopentameric GABA_A_R containing the *β*3-subunit at 3 Å resolution. Although this work provides great promise that other heteropentameric GABA_A_Rs will be crystallized in the near future, molecular modeling and structure-activity-relationships (SARs) still remain key tools to find better subtype-selective binding agents.

## 2. Subtype Selective Ligands for ***α***5 GABA(A)/Bz Receptors

Interest in BzR/GABA(A) *α*5 subtypes began years ago when it was realized that *α*5*β*3*γ*2 Bz/GABA(A) subtypes are located primarily in the hippocampus. More recently this interest has been confirmed by the report of Möhler et al. [2–5] on *α*5 “knock-in mice.” This group has provided strong evidence that hippocampal extrasynaptic *α*5 GABA(A) receptors play a critical role in associative learning as mentioned above [6–11].

Earlier we synthesized a series of *α*5 subtype selective ligands (RY-023, RY-024, RY-079, and RY-080) based on the structure of Ro 15-4513 and reported their binding affinity [6], as well as several ligands by Atack et al. [12]. These ligands are benzodiazepine receptor (BzR) negative modulators* in vivo* and a number of these compounds have been shown to enhance memory and learning [13]. One of these ligands was shown by Bailey et al. [6] to be important in the acquisition of fear conditioning and has provided further evidence for the involvement of hippocampal GABA(A)/BzR in learning and anxiety [13]. This is in agreement with the work of DeLorey et al. [7] in a memory model with a ligand closely related to *α*5 subtype selective inverse agonists RY-024 and RY-079 including PWZ-029 (**1**).

**Figure 1:**
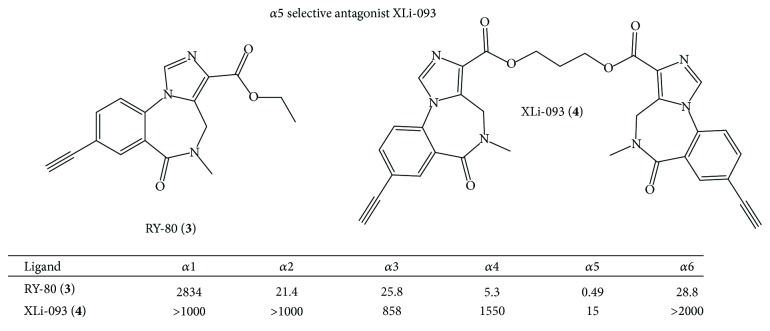
Alpha 5 selective compounds [13]. This figure is modified from that reported in [13].

In order to enhance the *α*5 subtype selectivity, the bivalent form of RY-80 (**3**) was prepared to provide XLi-093 (**4**) [13]. The binding affinity of XLi-093* in vitro* was determined on *α*_1–6_ *β*3*γ*2 LTK cells and is illustrated in Figure 1. This bivalent ligand exhibited little or no affinity at *α*_1–4,6_ *β*3*γ*2 BzR/GABA(A) subtypes, but this *α*5 ligand had a *K*_*i*_ of 15 nM at the *α*5*β*3*γ*2 subtype [14]. Since this receptor binding study indicated bivalent ligand XLi-093 bound almost exclusively to the *α*5 subtype, the efficacy of this ligand on GABA(A) receptor subtypes expressed in* Xenopus* oocytes was investigated by Sieghart, Furtmueller, Li, and Cook [14, 15]. Analysis of the data indicated that XLi-093 up to a concentration of 1 *μ*M did not trigger chloride flux in any one of the GABA(A) subtypes tested. At 1 *μ*M XLi-093 did not modulate GABA induced chloride flux in *α*1*β*3*γ*2, *α*2*β*3*γ*2, or *α*3*β*3*γ*2 receptors, but very slightly inhibited chloride flux in *α*5*β*3*γ*2 subtypes. At 1 *μ*M, XLi-093 barely influenced benzodiazepine (Valium) stimulation of GABA-induced current in *α*1*β*3*γ*2, *α*2*β*3*γ*2, and *α*3*β*3*γ*2 BzR but shifted the diazepam dose response curve to the right in *α*5*β*3*γ*2 receptors in a very significant manner [16]. Importantly, bivalent ligand XLi-093 was able to dose dependently and completely inhibit diazepam-stimulated currents in *α*5*β*3*γ*2 receptors. This was the first subtype selective benzodiazepine receptor site antagonist at *α*5 receptors. This bivalent ligand XLi-093 provided a lead compound for all of the bivalent ligands in this research [16].

**Figure 2:**
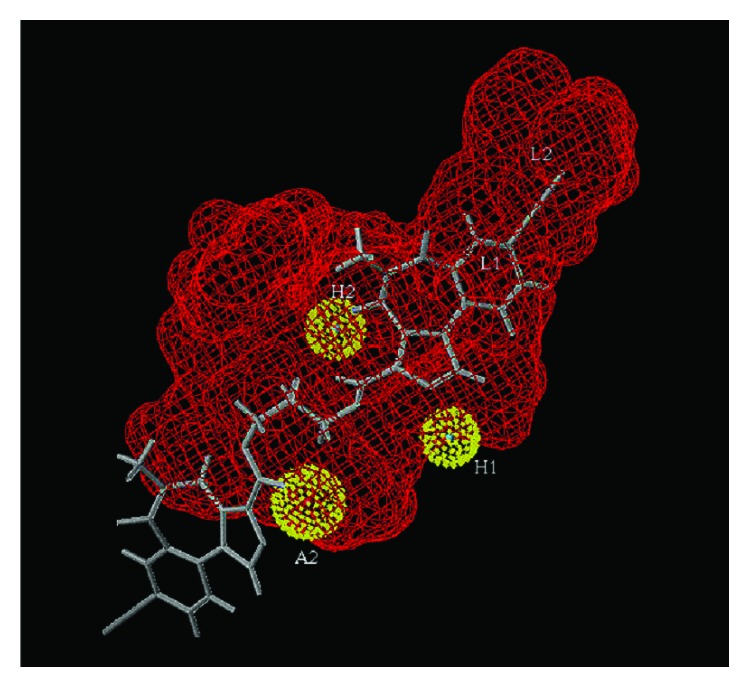
XLi-093 (**4**) aligned in the included volume of the pharmacophore receptor model for the *α*5*β*3*γ*2 subtype [17, 18] (this figure is modified from the figure in Clayton et al., 2007) [23].

Illustrated in Figure 2 is XLi-093 (**4**) aligned excellently within the pharmacophore-receptor model of the *α*5*β*3*γ*2 subtype [14, 16–19]. The fit to the pharmacophore-receptor and the binding data indicate that bivalent ligands will bind to BzR subtypes [14, 19]. It is believed that the dimer enters the binding pocket with one monomeric unit docking while the other monomer tethered by a linker extends out of the protein into the extracellular domain. If this is in fact true that the second imidazole unit is protruding into the extracellular domain of the BzR/GABA(A) *α*5 binding site, it could have a profound effect on the ligand design. This means other homodimers or even heterodimers may bind to BzR/GABA(A)ergic sites.

**Figure 3:**
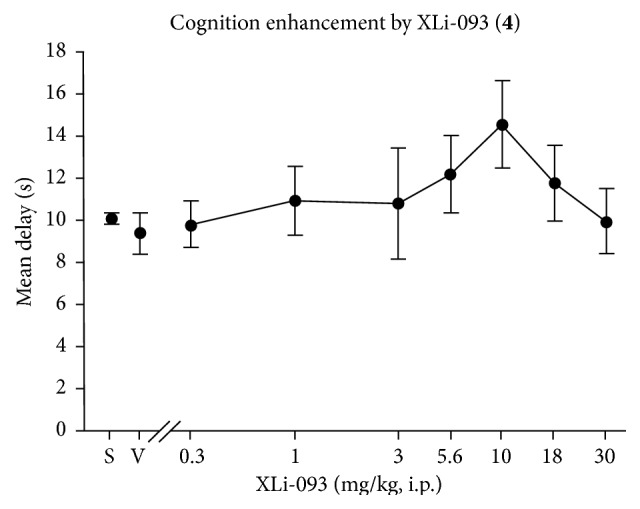
XLi-093 (**4**) effects on cognition enhancement by Wenger et al. (data on statistical significance not shown, unpublished results). Effects of** 4** on cognition from the mean delay achieved by C57BL/6J mice titrating delayed matching-to-position schedule.

In this vein, Wenger, Li, and Cook et al. [13, 20, 21] earlier described preliminary data that XLi-093, an *α*5 subtype selective antagonist, enhances performance of C57BL/6J mice under a titrating delayed matching to position schedule of cognition, as illustrated in Figure 3 [14, 16–19]. This indicates, however, that this agent does cross the blood brain barrier.

Bivalent ligands have a preferred linker of 3–5 methylene units, between the two pharmacophores (see XLi-093). This was established by NMR experiments run at low temperatures, X-ray crystallography, and molecular modeling of the ligands in question and will be discussed [14, 17, 18].

**Figure 4:**
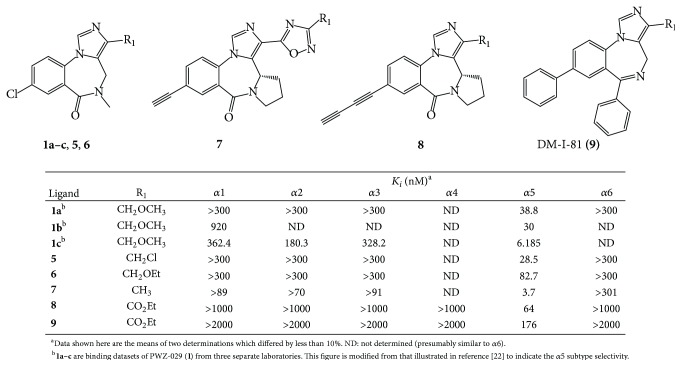
Binding data of selected imidazobenzodiazepines [22].

**Figure 5:**
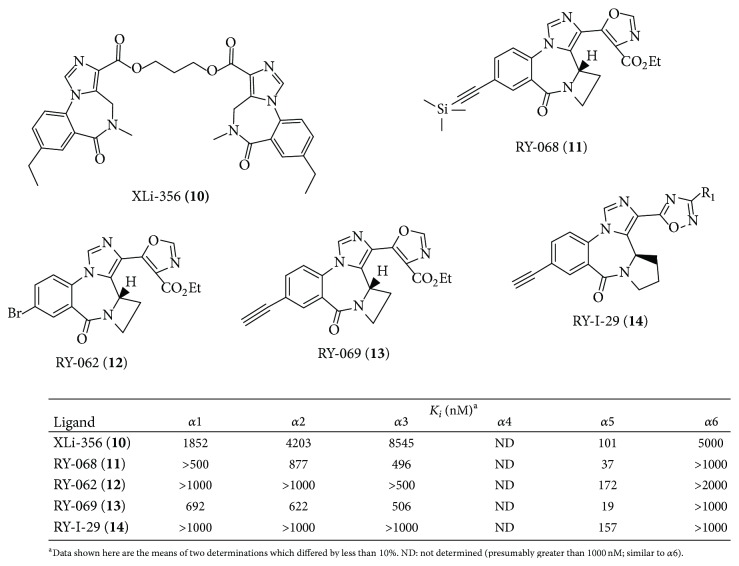
Binding data of selected imidazobenzodiazepines substituted with an E-ring as compared to XLi-356 (**10**).

Based on this data, additional *α*5-subtype selective ligands have been prepared (see Figure 4). The basic imidazobenzodiazepine structure has been maintained [7]; however substituents were varied in regions around the scaffold based on molecular modeling [6]. These are now the most *α*5 subtype selective ligands ever reported [22]. Moreover, the ability to increase the subtype selectivity can be done by selecting specific substituents on these ligands to new agents with 400–1000-fold *α*5-selectivity over the remaining 5 subtypes.* This is an important step forward to understanding the true, unequivocal physiological responses mediated by α5 subtypes in regard to cognition (amnesia), schizophrenia, anxiety, and convulsions, all of which in some degree are influenced by α5 subtypes*. Based on the ligands in Figures 4 and 5, affinity has occurred principally at *α*5 subtypes. In addition, since XLi-093 bound very tightly only to *α*5 BzR subtypes, the bivalent nature and functionality presented here can be incorporated into other dimeric ligands.

As shown previously in Figure 3, *α*5-antagonist XLi-093 (**4**) was shown to enhance cognition. In another study, a reduction of the two acetylenic groups of XLi-093 resulted in ethyl groups [14], providing a new bivalent ligand (XLi-356,** 10**) which shows *α*5-selective binding with very low affinity for *α*1 subtypes (Figure 5). Efficacy (oocyte) data shows XLi-356 is an *α*5 negative allosteric modulator [7, 13]. DeLorey et al. have recently shown in mice that XLi-356 does potently reverse scopolamine induced memory deficits [7]. This bivalent *α*5 inverse agonist enhanced cognition in agreement with work reported from our laboratory on monovalent inverse agonists RY-10 [6] and RY-23 [7].

The dimers XLi-093 (**4**) and XLi-356 (**10**) were sent to Case Western Reserve (NIMH supported PDSP program, Roth et al.) for full panel receptor binding and they do not bind to other receptors at levels of concern (Table 1).

Although XLi-093 (**4**) was found to be an antagonist at the *α*5 subtype, XLi-356 (**10**) was found to be a weak agonist-antagonist. XLi-356 was found to reverse scopolamine induced memory deficits in mice. When XLi-356 was looked at in audio cued fear conditioning, the results show no activity. This suggests that the effect of XLi-356 is selective through *α*5 receptors which are abundant in the hippocampus which is highly associated with contextual memory. Audio cued memory instead is amygdala-based and should not be affected by an *α*5 subtype selective compound [39–42].

**Figure 6:**
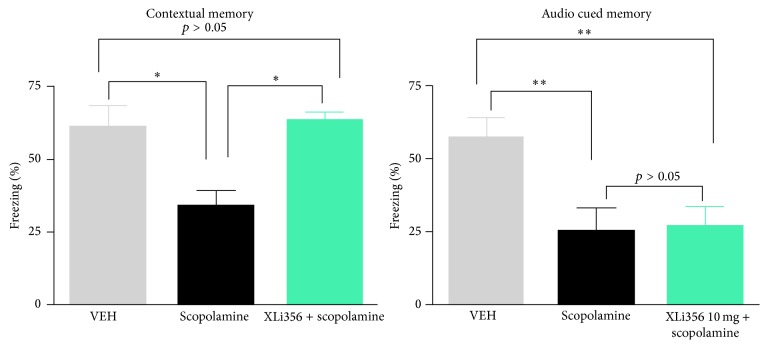
Visual and audio cued data for XLi-356 (**10**). This figure was modified from that in [22].

As illustrated in Figure 6, scopolamine (1 mg/kg) reduced freezing (i.e., impairs memory) generally due to coupling the context (the cage) with a mild shock. XLi-356 (10 mg/kg) attenuated the impairment of memory returning the freezing to the levels on par with subjects dosed with vehicle. In audio cued memory the response was activated by sound, not the context. XLi-356 was not able to reverse this type of memory effect which is amygdala driven. A similar effect was observed for XLi-093 by Harris et al. [43]. XLi-093 is the most selective antagonist for *α*5 subtypes reported to date [13, 43] and is a very useful *α*5 antagonist used by many* in vivo* [22, 44, 45].

Molecular modeling combined with this knowledge was used to generate new lead compounds aimed at the development of *α*5-subtype selective positive and negative allosteric modulators to study cognition as well as amnesia mediated by the hippocampus. All of these compounds have been prepared based on the structure of current *α*5-subtype selective ligands synthesized in Milwaukee [46] (see Figures 4 and 5), as well as the binding affinity (15 nM)/selectivity of bivalent *α*5 antagonist XLi-093 (**4**) [13].

In efforts to enhance *α*5-selectivity in regards to cognition, Cook, Bailey, and Helmstetter et al. have employed RY-024 to study the hippocampal involvement in the benzodiazepine receptor in learning and anxiety [14, 19]. Supporting this Harris, DeLorey et al. show in mice that *α*5 NAMs (**1**) and RY-10 potently reversed scopolamine-induced memory impairment. These *α*5 NAMs provide insight as to how GABA_A_Rs influence contextual memory, an aspect of memory affected in age associated memory impairment and especially in Alzheimer's disease [13, 62–64]. In addition, Savić et al. have used the *α*1 preferring antagonist, BCCt, in passive avoidance studies, in which midazolam's amnesic effects are shown to be due to interaction of agonist ligands at *α*5 in addition to *α*1*β*3*γ*2 BzR subtypes [24, 65].

## 3. PWZ-029: A Negative Allosteric Modulator

PWZ-029 (**1**) has been studied extensively as an *α*5-GABA_A_R inverse agonist and in certain experimental models has been shown to enhance cognition. The binding data from three separate laboratories (Table 2) have all shown that it exhibits remarkable selectivity for the *α*5 subunit-containing receptors, all greater than 60-fold compared to the next subunit.

**Figure 7:**
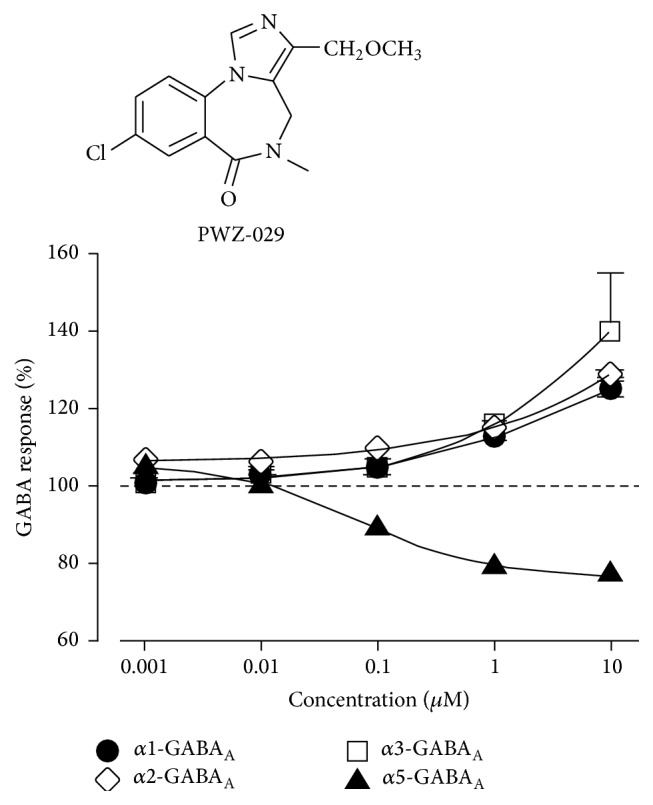
Oocyte electrophysiological data of PWZ-029 (**1**) [24].

Electrophysiological efficacy testing done by Sieghart et al. in oocytes demonstrated that PWZ-029 (**1**) acts as a negative allosteric modulator at the *α*5-subunit, with a very weak agonist activity at the *α*1, *α*2, and *α*3 subunits (Figure 7). At a pharmacologically relevant concentration of 0.1 *μ*M, PWZ-029 exhibits moderate negative modulation at the *α*5-subunit, while showing little or no effect at the *α*1, *α*2, or *α*3-subunits.

**Figure 8:**
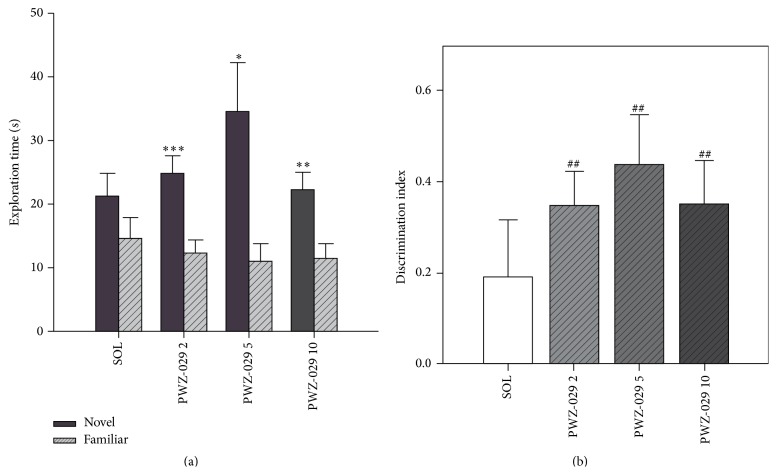
The effects of PWZ-029 (**1**) (2, 5 and 10 mg/kg) on (a) time exploring familiar and novel objects and (b) discrimination indices in the novel object recognition test using a 24 h delay (mean + SEM). Significant differences are indicated with asterisks (paired-samples *t*-test, novel versus familiar, ^*∗*^ *p* < 0.05, ^*∗∗*^ *p* < 0.01, ^*∗∗∗*^ *p* < 0.001). A significant difference from zero is indicated with hashes (one sample *t*-test, ^##^ *p* < 0.01). The number of animals per each treatment group was 10. SOL = solvent [25].

**Figure 9:**
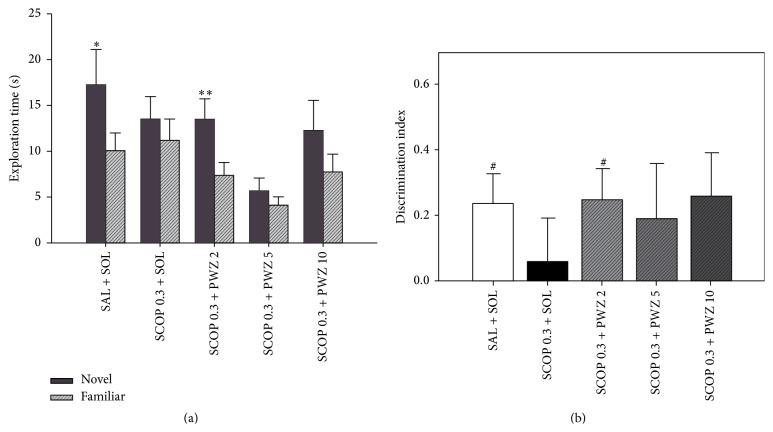
The effects of 0.3 mg/kg scopolamine (SCOP 0.3) and combination of 0.3 mg/kg scopolamine and PWZ-029 (**1**) (2, 5, and 10 mg/kg) on the rats' performance in the object recognition task after a 1 h delay: (a) time exploring familiar and novel objects and (b) discrimination index. Data are represented as mean + SEM. Significant differences are indicated with asterisks (paired-samples *t*-test, novel versus familiar, ^*∗*^ *p* < 0.05, ^*∗∗*^ *p* < 0.01). A significant difference from zero is indicated with hashes (one sample *t*-test, ^#^ *p* < 0.05). The number of animals per each treatment group was 12–15. SAL = saline, SOL = solvent [25].

Milić et al. reported on the effects of PWZ-029 in the widely used novel object recognition test, which differentiates between the exploration time of novel and familiar objects. As shown by significant differences between the exploration times of the novel and familiar object (Figure 8(a)), as well as the respective discrimination indices (Figure 8(b)), all the three tested doses of PWZ-029 (2, 5 and 10 mg/kg) improved object recognition in rats after the 24 h delay period. Additionally, in the procedure with the 1 h delay between training and testing, the lowest of the tested doses of PWZ-029 (2 mg/kg) successfully reversed the deficit in recognition memory induced by 0.3 mg/kg scopolamine (Figure 9) [25].

The results of the described study showed for the first time that inverse agonism at *α*5-GABA_A_ receptors may be efficacious in both improving cognitive performance in unimpaired subjects and ameliorating cognitive deficits in pharmacologically impaired subjects, as assessed in two protocols of the same animal model [25].

**Figure 10:**
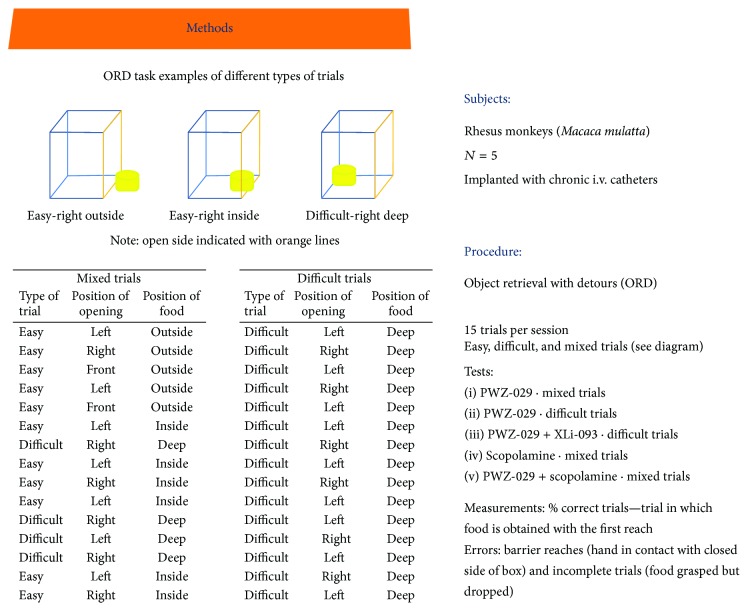
ORD methods and procedure [26].

**Figure 11:**
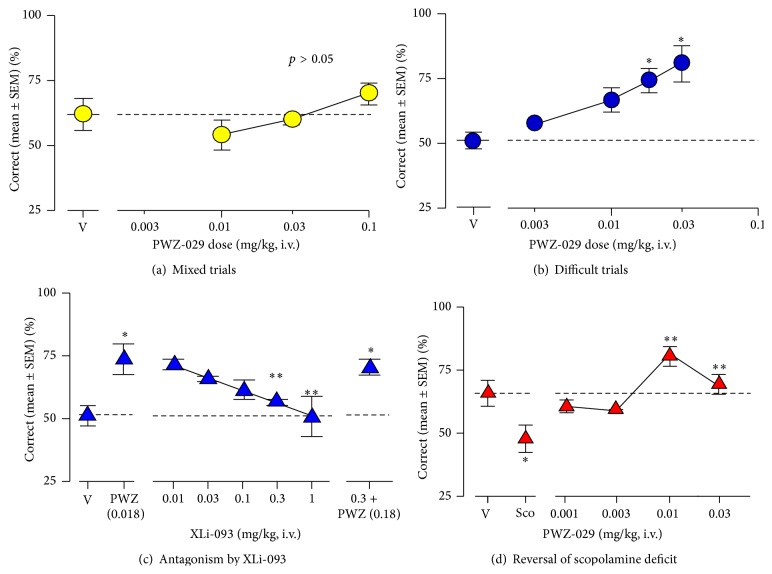
Cognitive-enhancing effects of PWZ-029 in the rhesus monkey Object Retrieval with Detours (ORD) task (*n* = 5 monkeys). (a) Effects of PWZ-029 on ORD tests consist of both easy and difficult trials: (b) PWZ-029 enhanced performance on the ORD task when tested with difficult trials only; (c) enhancement of ORD performance by 0.018 mg/kg of PWZ-029 was attenuated by the *α*5 GABA_A_-preferring antagonist XLi-093 and this antagonism was surmountable by increasing the PWZ-029 dose; (d) PWZ-029 reversed performance impaired by 0.01 mg/kg of scopolamine [26]. ^*∗*^ *p* < 0.05 versus vehicle, ^*∗∗*^ *p* < 0.05 versus Scopolamine.

In a recent by Rowlett et al. [26], negative allosteric modulator PWZ-029 was evaluated in female rhesus monkeys (*n* = 4) in an Object Retrieval test with Detours (ORD; Figure 10 for details).** 1** was administered via i.v. catheters in ORD trained monkeys and evaluated for cognition enhancement. A successful trial was determined by the ability of the subject to obtain a food reward within a transparent box with a single open side, with varying degrees of difficulty (“easy” or “difficult” or “mixed” as a combination of both) based on food placement within the box. In “mixed” trials using PWZ-029, no significant results were observed when compared to vehicle (Figure 11(a)). “Difficult” trials, however, exhibited an increasing dose-dependent curve for successful trials (Figure 11(b)). These results were attenuated by a coadministration *α*5-antagonist XLi-093 (Figure 11(c)). PWZ-029 was also shown to dose-dependently reverse the cholinergic deficits that were induced by scopolamine (Figure 11(d)) [26].

These findings suggest that PWZ-029 can enhance performance on the ORD task, only under conditions in which baseline performance is attenuated. The effects of PWZ-029 were antagonized in a surmountable fashion by the selective *α*5-GABA_A_ ligand, XLi-093, consistent with PWZ-029's effects being mediating via the *α*5-GABA_A_ receptor. The results are consistent with the view that *α*5 GABA_A_ receptors may represent a viable target for discovery of cognitive enhancing agents.

**Figure 12:**
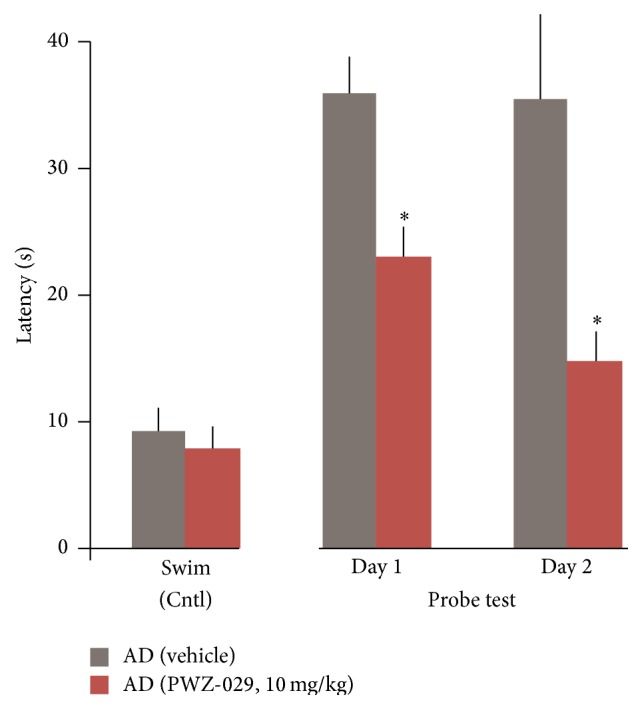
PWZ-029 rescues spatial memory deficits in AD model as evidenced by a decrease in the latency to reach the hidden platform (probe test) in the water maze relative to vehicle (VEH, ^*∗*^ *p* < 0.05).

In addition, we have new data showing that modulation of *α*5-GABA_A_Rs by PWZ-029 rescues Hip-dependent memory in an AD rat model [PMID: 23634826] as evidenced by a significant decrease in the latency to reach the hidden platform (memory probe trials) on spatial water maze task (Figure 12). Roche has employed a similar strategy at *α*5 subtypes and recently has a drug in the clinic to treat symptoms of dementia in Down syndrome patients. It is well known many Down syndrome patients develop Alzheimer's disease or a dementia with a very similar etiology. This is aimed at treating early onset Alzheimer's patients.

## 4. PWZ-029 Docking within ***α***5***γ***2 **GABA_**A**_** Receptor Subunit Homology Model

**Figure 13:**
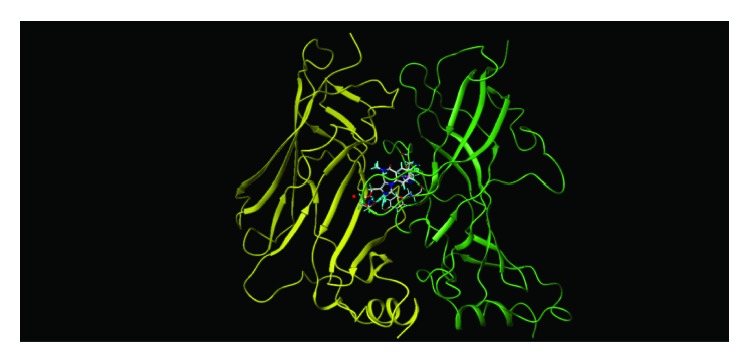
PWZ-029 docked within *α*5*γ*2 BzR binding site (BS).

**Figure 14 fig14:**
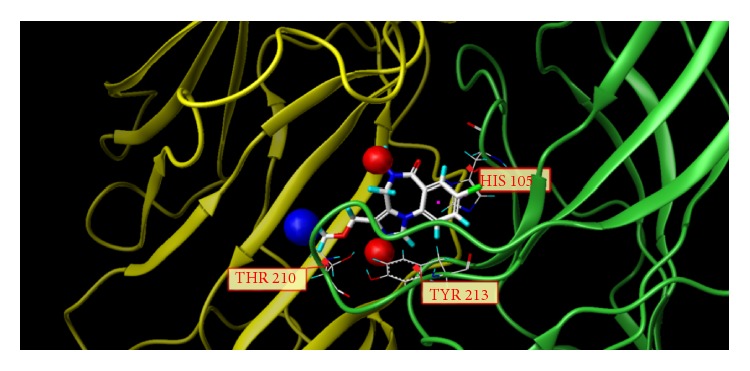
PWZ-029 docked with amino acid residues.

**Figure 15 fig15:**
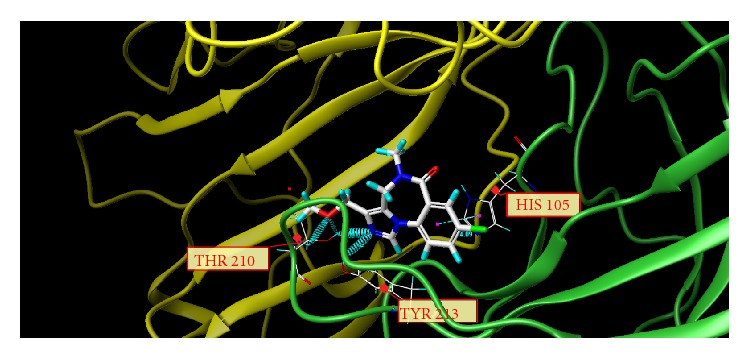
PWZ-029 docked with A.A. residue interactions.

These studies with PWZ-029 led to the molecular model rendering of the compound docked within the *α*5*γ*2 BzR subtype (Figures 13–16). The model figures have the following features:

The docking of PWZ-029 within the GABA_A_/BzR shows the molecule bound and interacting with specific amino acids. The A and B rings of the benzodiazepine framework undergo a *π*-stacking interaction with HIS 105, indicated by the magenta coloring. At the other end of the molecule the methoxy lone pair and imidazole nitrogen lone pair act as a hydrogen bond acceptors with THR 210 and TYR 213, respectively. These interactions are shown by the aqua-blue descriptors.

## 5. Subtype Selective Agonists for ***α***5 **GABA_**A**_**/Bz Receptors

**Figure 17:**
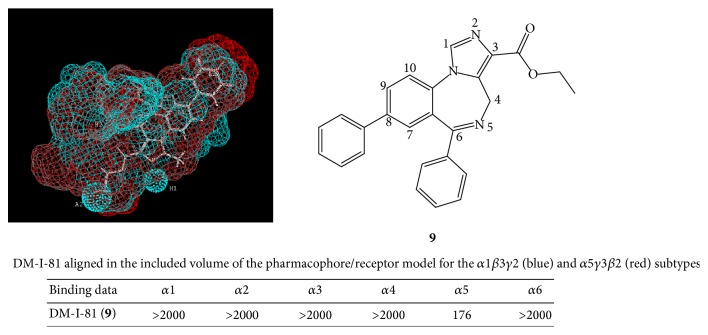
The *α*5 selective agonist DM-I-81 (**9**), bound within the *α*1 and *α*5 subtypes. Binding data shown as *K*_*i*_ (nM).

Möhler has proposed that *α*5 selective inverse agonists or *α*5 selective agonists might enhance cognition [5, 13, 16–18, 86]. This is because of the extrasynaptic pyramidal nature of *α*5*β*3*γ*2 subtypes, located almost exclusively in the hippocampus. Because of this, a new “potential agonist” which binds solely to *α*5*β*3*γ*2 subtypes was designed by computer modeling (see Figure 17). This ligand (DM-I-81,** 9**) has an agonist framework and binds only to *α*5*β*3*γ*2 subtypes [13, 17, 18, 86]. The binding potency at *α*5 subtypes is 176 nM. Although the 8-pendant phenyl of DM-I-81 was lipophilic and bound to the *L*_2_ pocket, additional work on the 8-position of this scaffold has been abandoned and generally left as an acetylene or halide function, with a few exceptions. The steric bulk of the 8-phenyl moiety was felt detrimental to activity and potency which may have led to the weak binding affinity.

## 6. Alpha 5 Positive Allosteric Modulators in Schizophrenia

In addition to inverse agonists, a number of other *α*5-GABA_A_R positive allosteric modulators (PAMs) have been synthesized. These compounds, such as SH-053-2′F-R-CH_3_ (**2**), have been shown to decrease the firing rate of synapses controlling cognition and can be used to treat schizophrenia.

The following is reported by Gill, Cook, and Grace et al. [27–38].

**Figure 18:**
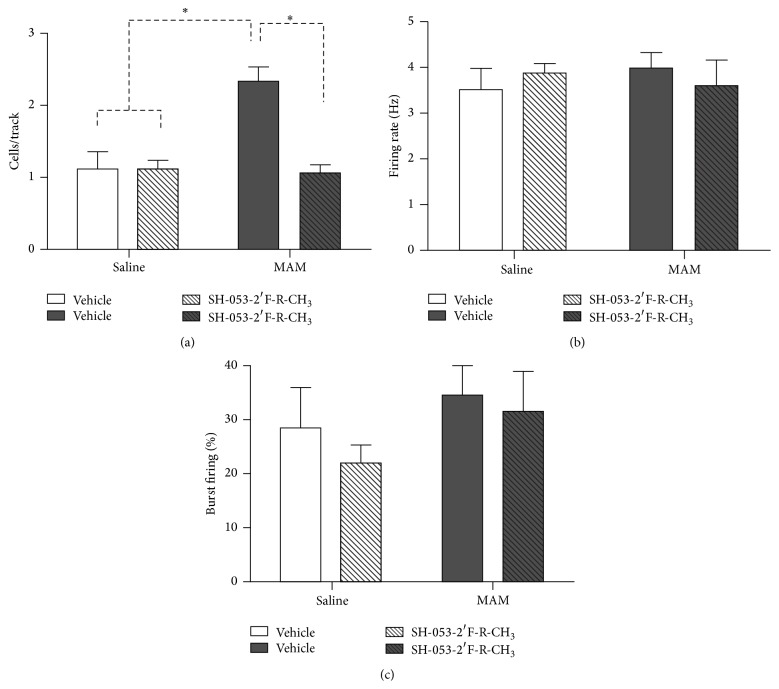
Treatment with SH-053-2′F-R-CH_3_ (0.1 mg/kg, i.v.; patterned bars) normalizes the aberrant increase in the number of spontaneously firing dopamine neurons (expressed as cells/track) in methylazoxymethanol acetate- (MAM-) treated animals (a). There was no effect of SH-053-2′F-R-CH_3_ treatment in control animals (open bars, (a)–(c)) or on firing rate and burst activity in MAM animals (dark bars; (b)-(c)) (^*∗*^ *p* < 0.05, two-way ANOVA, Holm-Sidak* post hoc*; *N* = 5–7 rats/group) [27–38].

**Figure 19:**
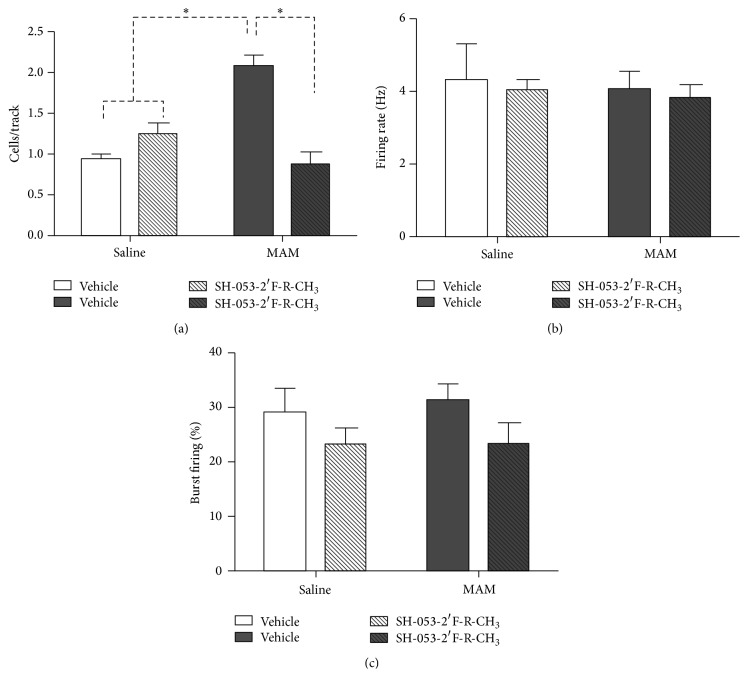
Hippocampal (HPC) infusion of SH-053-2′F-R-CH_3_ (1 *μ*M/side; patterned bars) normalizes the aberrant increase in the number of spontaneously firing dopamine neurons (expressed as cells/track) in methylazoxymethanol acetate- (MAM-) treated animals (a). There was no effect of SH-053-2′F-R-CH_3_ treatment in control animals (open bars, (a)–(c)) or on firing rate in MAM animals (dark bars; (b)). Hippocampal (HPC) infusion of SH-053-2′F-R-CH_3_ significantly reduced the percentage of spikes occurring in bursts of dopamine (DA) neurons in MAM and control animals (c) (^*∗*^ *p* < 0.05, two-way ANOVA, Holm-Sidak* post hoc*; *N* = 7 rats/group) [27–38].

There are a number of novel benzodiazepine-positive allosteric modulators (PAMs), selective for the *α*5 subunit of the GABA_A_ receptor, including SH-053-2′F-R-CH_3_ (**2**), which has been tested for its ability to effect the output of the HPC (hippocampal) in methylazoxymethanol- (MAM-) treated animals, which can lead to hyperactivity in the dopamine system [27–38]. In addition, the effect of this compounds (**2**) response to amphetamine in MAM-animals on the hyperactive locomotor activity was examined. Schizophrenic-like symptoms can be induced into rats when treated prenatally with DNA-methylating agent, methylazoxymethanol, on gestational day (GD) 17. These neurochemical outcomes and changes in behavior mimic those found in schizophrenic patients. Systemic treatment with (**2**) resulted in a reduced number of spontaneously active DA (dopamine) neurons in the VTA (ventral tegmental area) of MAM animals (Figure 18) to levels seen in animals treated with vehicle (i.e., saline). To confirm the location of action,** 2** was also directly infused into the ventral HPC (Figure 19) and was shown to have the same effect. Moreover, HPC neurons in both SAL and MAM animals showed diminished cortical-evoked responses following *α*5-GABA_A_R PAM treatment. This study is important for it supports a treatment of schizophrenia that targets abnormal HPC output, which in turn normalized dopaminergic neuronal activity [27–38]. This is a novel approach to treat schizophrenia.

The pathophysiology of schizophrenia has identified hippocampal (HPC) dysfunction as a major mediator as reported by many including Anthony Grace [27–38]. This included morphological changes, reduced HPC volume, and GAD67 expression [27, 28] that have been reported after death in the brains of patients with schizophrenia. Both HPC activation and morphology changes have been identified that can precede psychotic symptoms or correlate with severity of cognitive deficits [29–33]. This has been shown in a cognitive test during baseline and activation.

Many animal models of schizophrenia were essential to behavioral pathology and have delivered new knowledge about the network disturbances that contribute to CNS disorder. This study shows that the offspring of MAM-treated animals showed both structural and behavioral abnormalities. These were consistent with those observed in patients with schizophrenia. The animals had reduced limbic cortical and HPC volumes with increased cell packing density and showed increased sensitivity to psychostimulants [34–36]. In addition, the startle response in prepulse inhibition was reduced in MAM-treated animals and deficits in latent inhibition were observed [35]. Furthermore, a pathological rise in spontaneous dopamine (DA) activity by the ventral tegmental area (VTA) was observed that can be attributed to aberrant activation within the ventral HPC [36]. It was suggested that reductions in parvalbumin- (PV-) stained interneurons might be the reason for the hyperactivation of the HPC and disruption of normal oscillatory activity in the HPC and cortex of MAM animals [38, 61]. At least this is the prevailing hypothesis at the moment put forth by many investigators (see references cited in [27–38]).

**Figure 20:**
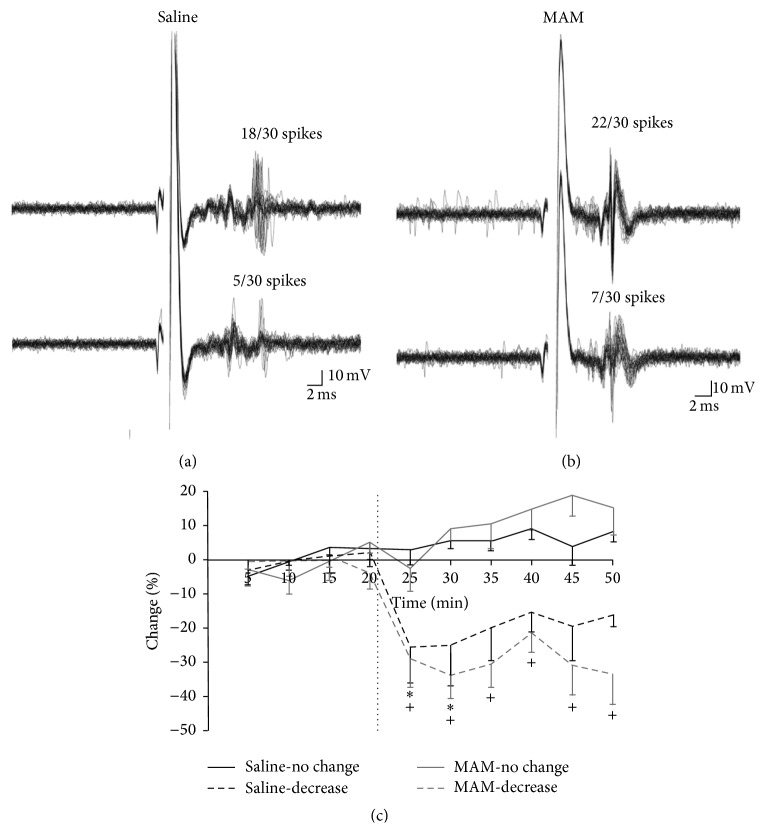
Extracellular recording traces illustrate the reduction in evoked responses in the ventral hippocampal (HPC) to entorhinal cortex stimulation in both MAM- and saline-treated animals (a, b). Treatment with SH-053-2′F-R-CH_3_ (0.1 mg/kg, i.v.) decreases the evoked excitatory response (dashed lines) of ventral HPC neurons to entorhinal cortex stimulation in both MAM- and saline-treated animals (c) (^*∗*^ *p* < 0.05 for saline and ^+^ *p* < 0.05, two-way repeated measures ANOVA, Holm-Sidak* post hoc*) [27–38].

**Figure 21:**
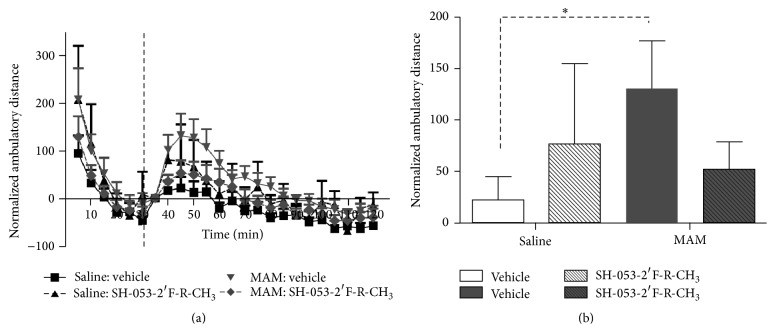
Treatment with SH-053-2′F-R-CH_3_ (10 mg/kg, i.p.) reduced the aberrant increased locomotor response to D-amphetamine (0.5 mg/kg i.p.) observed in MAM rats (a). MAM animals demonstrated a significantly larger peak locomotor response than both saline-treated animals and MAM animals pretreated with the alpha-5 PAM (b) (there was a significant difference between MAM-vehicle and all other groups, ^*∗*^ *p* < 0.05, two-way repeated measures ANOVA, Holm-Sidak* post hoc*) [27–38].

Selective *α*5-GABA_A_R positive allosteric modulator (**2**) was successful in reversing the pathological increase in tonic DA transmission in methylazoxymethanol rats by targeting abnormal hippocampal activity. In addition, the *α*5-PAM was able to reduce the behavioral sensitivity to psychostimulants observed in MAM rats (Figures 20 and 21). This suggests that novel *α*5-partial allosteric modulators should be effective in alleviating dopamine-mediated psychosis. However, if this drug can also restore rhythmicity within HPC-efferent structure, it may also affect other aspects of this disease state such as cognitive disabilities and negative symptoms. This study, using the MAM-model to induce symptoms of schizophrenia, shows that the use of *α*5-GABA_A_R targeting compounds could be an effective treatment in schizophrenic patients. The selective targeting solely of *α*5*β*3*γ*2 subunits, as opposed to unselective BZDs such as diazepam, could provide relief from the psychotic symptoms without producing adverse effects such as sedation [27–38].

As reported by Gill, Grace et al. [36, 38, 47–61].

Often initial antipsychotic drug treatments (APD) for schizophrenia are ineffective, requiring a brief washout period prior to secondary treatment. The impact of withdrawal from initial APD on the dopamine (DA) system is unknown. Furthermore, an identical response to APD therapy between normal and pathological systems should not be assumed. In another study by Gill, Grace et al., *α*5 positive allosteric modulator SH-053-2′F-R-CH_3_ (**2**) was used in the MAM neurodevelopmental model of schizophrenia which was used to study impact of withdrawal from repeated haloperidol (HAL) on the dopamine system [36, 38, 47–61].

The following studies were designed to provide insight as to why a new drug to treat schizophrenia may be effective in Phase II clinical trials but fail in Phase III because of the large number of patients required for the study. Many of these patients in Phase III studies have altered neuronal pathways in the CNS because of long-term treatment with antipsychotics (sometimes 10–20 years) [36, 38, 47–61].

Importantly, spontaneous dopamine activity reduction was observed in saline rats withdrawn from haloperidol with an enhanced locomotor response to amphetamine, indicating the development of dopamine supersensitivity. In addition, PAM treatment, as well as ventral HPC inactivation, removed the depolarization block of DA neurons in withdrawn HAL treated SAL rats. In contrast, methylazoxymethanol rats withdrawn from HAL displayed a reduction in spontaneous dopamine activity and enhanced locomotor response that was unresponsive to PAM treatment with SH-053-2′F-R-CH_3_ or ventral HPC inactivation [36, 38, 47–61].

Prior HAL treatment withdrawal can restrict the efficacy of subsequent pharmacotherapy in the MAM model of schizophrenia. This is an extremely important result indicating that testing a new drug for schizophrenia in humans treated for years with both typical and atypical antipsychotics may result in a false negative with regard to treatment. Studies that support this hypothesis follow here [36, 38, 47–61].

Novel therapeutics for the treatment of schizophrenia that exhibit initial promise in preclinical trials often fail to demonstrate sufficient efficacy in subsequent clinical trials. In addition, relapse or noncompliance from initial treatments is common, necessitating secondary antipsychotic intervention [47, 48]. Studies have shown that between 49 and 74% of schizophrenia patients discontinue the use of antipsychotic drug (APD) treatments within 18 months due to adverse side-effects [48, 49]. Current pharmacotherapies for schizophrenia target the pathological increase in dopamine system activity, as mentioned above. Common clinical practice for secondary antipsychotic application involves a brief withdrawal period from the initial APD. Unfortunately, the success of even secondary treatments is far from being optimal with the rehospitalization of patients being a common occurrence. The impact of repeated antipsychotic treatment and subsequent withdrawal on the dopamine system has not been adequately assessed [36, 38, 47–61].

As indicated above, schizophrenia is a complex chronic psychiatric illness characterized by frequent relapses despite ongoing treatment. The search for more effective pharmacotherapies for the treatment of schizophrenia continues unabated. It is not uncommon for novel pharmaceuticals to demonstrate promise in preclinical trials but fail to show an adequate response in subsequent clinical trials. Indeed, evaluating the benefits of one APD versus another is complicated by clinical trials beset with high attrition rates and poor efficacy in satisfactorily reducing rehospitalization [47, 49–52].

**Figure 22:**
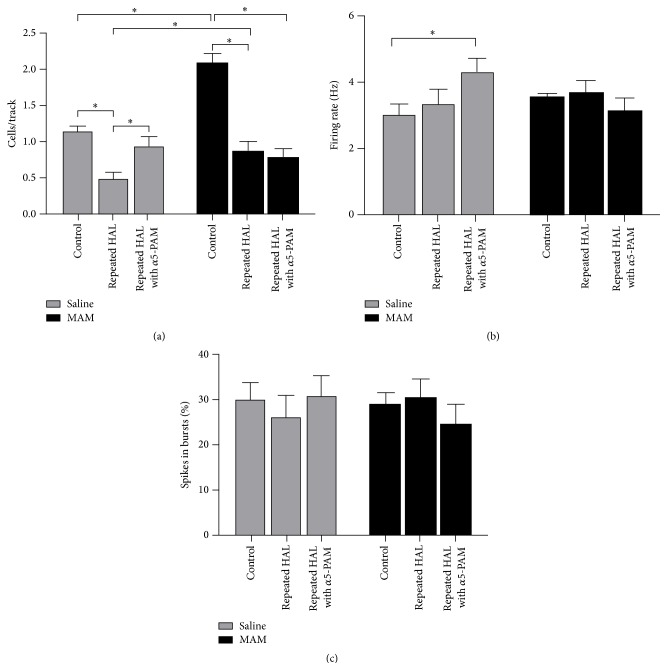
Repeated haloperidol treatment caused a reduction in the number of spontaneously active dopamine neurons in both SAL and MAM rats injected with vehicle compared to untreated control animals. Treatment with SH-053-2′F-R-CH_3_ (0.1 mg/kg, i.v.) reversed the haloperidol-induced reduction in cells/track in SAL, but not MAM, rats (a). Repeated haloperidol treatment had no effect on the firing rate of dopamine neurons recorded in SAL or MAM rats treated with vehicle. However, SH-053-2′F-R-CH_3_ caused an increase in firing rate of dopamine neurons in repeatedly haloperidol-treated SAL rats (b). Repeated haloperidol treatment, as well as SH-053-2′F-R-CH_3_ injection, had no impact on the percentage of spikes occurring in bursts for dopamine neurons recorded in SAL and MAM rats (c) [36, 38, 47–61]. ^*∗*^ *p* < 0.05.

**Figure 23 fig23:**
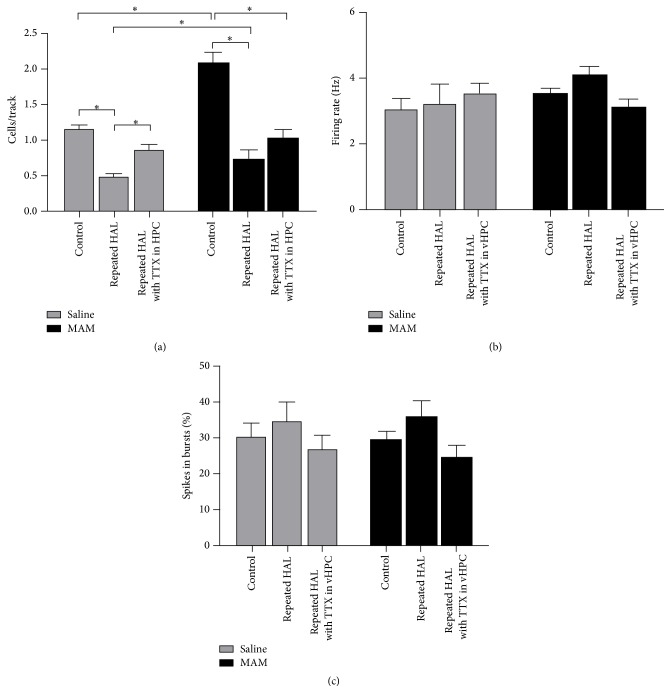
Repeated haloperidol treatment caused a reduction in the number of spontaneously active dopamine neurons in both SAL and MAM rats microinfused with vehicle in the ventral HPC compared to untreated control animals. Infusion of TTX in the ventral HPC reversed the haloperidol-induced reduction in cells/track in SAL, but not MAM, rats (a). Repeated haloperidol treatment had no effect on the firing rate of dopamine neurons recorded in SAL or MAM rats infused with vehicle or TTX in the ventral HPC (b). Repeated haloperidol treatment had no effect on the percentage of spikes occurring in bursts for dopamine neurons recorded in SAL or MAM rats infused with vehicle or TTX in the ventral HPC (c) [36, 38, 47–61]. ^*∗*^ *p* < 0.05.

**Figure 24 fig24:**
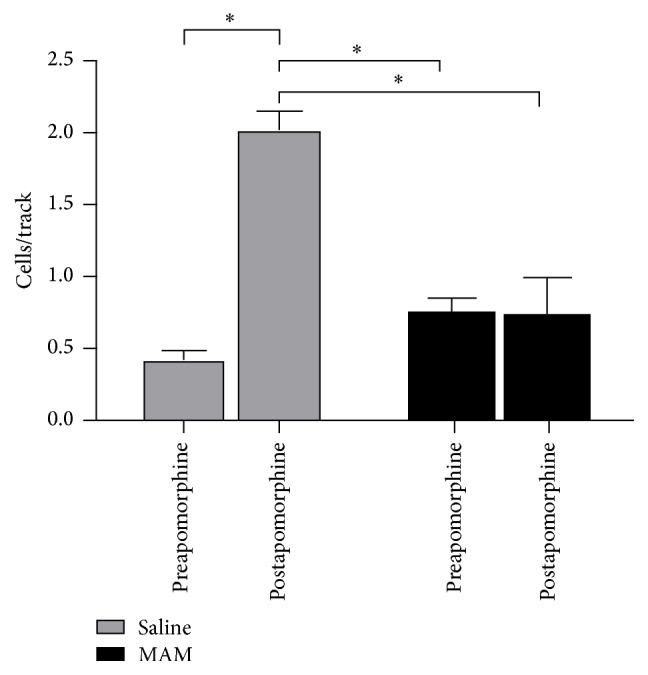
Administration of apomorphine (80 mg/kg i.v.) increased the number of spontaneously active dopamine neurons in SAL rats withdrawn from repeated HAL, while having no effect on the number of active dopamine neurons in MAM rats withdrawn from repeated HAL [36, 38, 47–61]. ^*∗*^ *p* < 0.05.

**Figure 25:**
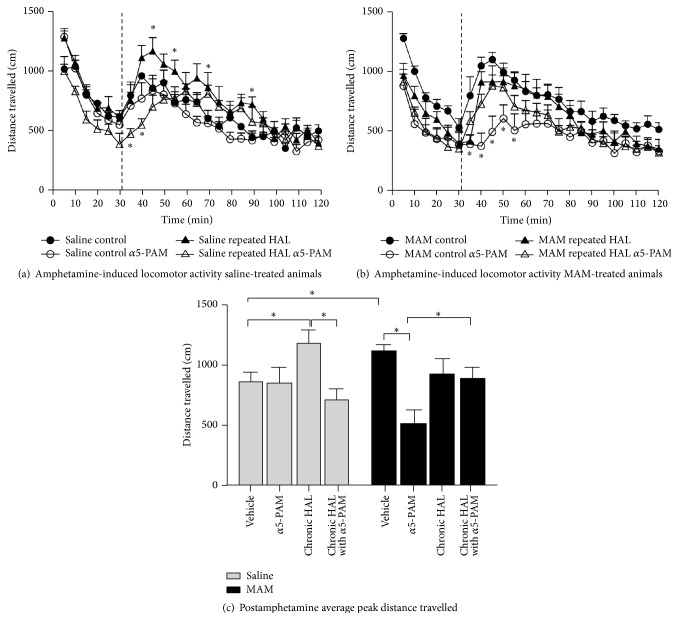
Repeated haloperidol treatment causes an enhancement in the locomotor response to D-amphetamine (0.5 mg/kg, i.p.) in SAL animals that is reduced by pretreatment with SH-053-2′F-R-CH_3_ (10 mg/kg, i.p.) (a). MAM rats treated repeatedly with haloperidol exhibit a locomotor response following D-amphetamine similar to untreated MAM rats. However, repeated haloperidol treatment blocks the effect of SH-053-2′F-R-CH_3_ pretreatment in decreasing the locomotor response in MAM rats (b). Untreated MAM rats demonstrated a significantly larger peak locomotor response than untreated SAL rats. In addition, SH-053-2′F-R-CH_3_ pretreatment significantly reduced the peak locomotor response in untreated MAM rats, while having no effect in repeatedly haloperidol-treated MAM rats. In contrast, repeated haloperidol treatment enhanced the peak locomotor response to amphetamine in SAL rats that was reduced by SH-053-2′F-R-CH_3_ pretreatment (c) [36, 38, 47–61]. ^*∗*^ *p* < 0.05.

Previous work from the Gill, Grace et al.'s laboratory [36, 38, 47–61] with the MAM model of schizophrenia has identified a potential novel therapeutic, a *α*5GABAAR PAM. The dopamine system pathology in the MAM model is likely the result of excessive output from the ventral HPC [36]. The *α*5GABAAR PAM was identified as a potential therapeutic due to the relatively selective expression of *α*5GABAAR in the ventral HPC and its potential for reducing HPC activity [53–60]. When either administered systemically or directly infused into the ventral HPC, the *α*5GABAAR PAM (SH-053-2′F-R-CH_3_) was effective in reducing the dopamine system activation in MAM rats [38]. Anthony Grace, Gill et al. showed *α*5GABAAR PAM treatment was also effective in reducing the enhanced behavioral response to amphetamine in MAM rats, as stated above. Data from the present study sought to delineate whether the *α*5GABAAR PAM (SH-053-2′F-R-CH_3_) would remain effective in MAM rats withdrawn from prior neuroleptic treatment, a common occurrence in the patient population. In both SAL and MAM rats, there was a reduction in the spontaneous activity of dopamine neurons in the VTA after 7 days withdrawal from repeated HAL treatment. However, MAM rats continued to exhibit a greater activation of the dopamine system in comparison to SAL rats. Treatment with the *α*5GABAAR PAM was no longer effective in reducing the activity of dopamine neurons in the VTA in withdrawn HAL treated MAM rats. In contrast, *α*5GABAAR PAM treatment in the withdrawn HAL treated SAL rats instead increased the spontaneous activity of dopamine in the VTA (Figures 22 [Fig fig23] [Fig fig24]–25) [36, 38, 47–61].

Similar to the effects seen following *α*5GABAAR PAM treatment, ventral HPC inactivation in withdrawn HAL treated SAL rats restored normal dopamine system activity by increasing the number of spontaneously active dopamine neurons. The disparate effect of withdrawal from HAL on the dopamine system between SAL and MAM rats provides a vital clue for the inconsistencies between preclinical trials for novel therapeutics that utilize normal subjects and subsequent clinical trials in a patient population [36, 38, 47–61].

The data suggests underlying dopamine system pathology alters the impact of withdrawal from prior repeated HAL in the MAM model of schizophrenia. In addition, subsequent novel APD treatment loses efficacy following withdrawal from repeated HAL in MAM animals. This certainly has relevance to Phase III clinical trials of new drugs to treat schizophrenia [36, 38, 47–61].

## 7. **GABA_**A**_** ***α***5 Positive Allosteric Modulators Relax Airway Smooth Muscle

Emala, Gallos, et al. [66–75] have found that novel *α*5-subtype selective GABA_A_ positive allosteric modulators relax airway smooth muscle from rodents and humans. The clinical need for new classes of bronchodilators for the treatment of bronchoconstrictive diseases such as asthma remains a major medical issue. Few novel therapeutics have been approved for targeting airway smooth muscle (ASM) relaxation or lung inflammation in the last 40 years [66]. In fact, several asthma-related deaths are attributed, in part, to long-acting *β*-agonists (LABA) [67]. Adherence to inhaled corticosteroids, the first line of treatment for airway inflammation in asthma, is very poor [68, 69]. Therapies that break our dependence on *β*-agonism for ASM relaxation would be a novel and substantial advancement.

These ASM studies were undertaken due to a pressing clinical need for novel bronchodilators in the treatment of asthma and other bronchoconstrictive diseases such as COPA. There are only three drug classes currently in clinical use as acute bronchodilators in the United States (methylxanthines, anticholinergics, and *β*-adrenoceptor agonists) [70]. Thus, a novel therapeutic approach that would employ cellular signaling pathways distinct from those used by these existing therapies involves modulating airway smooth muscle (ASM) chloride conductance via GABA_A_ receptors to achieve relaxation of precontracted ASM [71, 72]. However, widespread activation of all GABA_A_ receptors may lead to undesirable side effects (sedation, hypnosis, mucus formation, etc.). Thus, a strategy that selectively targets a subset of GABA_A_ channels, those containing *α* subunits found to be expressed in airway smooth muscle, may be a first step in limiting side effects. Since human airway smooth muscle contains only *α*4 or *α*5 subunits [72], ligands with selectivity for these subunits are an attractive therapeutic option. Concern regarding nonselective GABA_A_ receptor activation is not limited to the airway or other peripheral tissues. GABA_A_ receptor ligands are classically known for their central nervous system effects of anxiolysis, sedation, hypnosis, amnesia, anticonvulsion, and muscle relaxant effects. Such indiscriminate activation of GABA_A_ receptors in the CNS is exemplified by the side effects of classical benzodiazepines (such as diazepam) which were the underpinning for the motivation of a search for benzodiazepine (BZD) ligands that discriminate among the *α* subunits of GABA_A_ receptors [73–75].

A novel approach to identify novel benzodiazepine derivatives to selectively target GABA_A_ channels containing specific *α* subunits was developed by Cook et al. in the 1980s that employed a pharmacophore receptor model based on the binding affinity of rigid ligands to BDZ/GABA_A_ receptor sites (as reviewed in 2007 [23]). From this series of receptor models for *α*_1–6_ *β*3*γ*2 subtypes a robust model for *α*5 subtype selective ligands emerged, the result of which included the synthesis of a novel *α*5*β*3*γ*2 partial agonist modulator, SH-053-2′F-R-CH_3_ (**2**). The discovery of this and related ligands selective for *α*5 BDZ/GABA_A_-ergic receptors and the realization that only *α*4 and *α*5 subunits are expressed in GABA_A_ channels on human airway smooth muscle yielded an ideal opportunity for targeting these *α*5-subunit containing GABA_A_ channels for bronchorelaxation [66–75].

**Figure 26:**
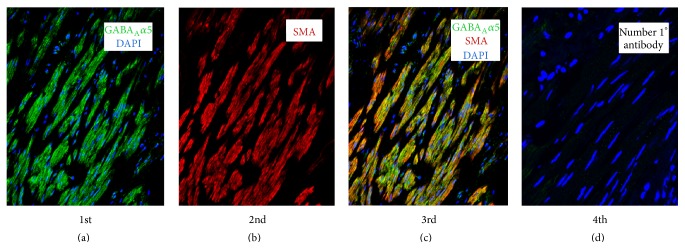
Protein expression of the GABA_A_ *α*5 subunit in intact human trachea-bronchial airway smooth muscle. Representative images of human tracheal airway smooth muscle sections using confocal microscopy are depicted following single, double, and triple immunofluorescence labeling. The antibodies employed were directed against the GABA_A_ *α*5 subunit (green), *α*-smooth muscle actin (SMA; red), and/or the nucleus via DAPI counterstain (blue). Panels illustrate the following staining parameters from left to right: (1st) costaining of DAPI and GABA_A_ *α*5 subunit; (2nd) *α*-SMA staining alone; (3rd) triple-staining of GABA_A_ *α*5, *α*-SMA, and DAPI; (4th) DAPI nucleus counterstain, with primary antibodies omitted as negative control. Modified from [66–75].

The GABA_A_ *α*5 subunit protein was first localized to the ASM layer of human trachea while costaining for the smooth muscle specific protein *α* actin (Figure 26). The first panel of Figure 26 shows GABA_A_ *α*5 protein stained with fluorescent green and blue fluorescent nuclear staining (DAPI). The second panel is the same human tracheal smooth muscle section simultaneously stained with a protein specific for smooth muscle, *α* actin, and the third panel is a merge of the first two panels showing costaining of smooth muscle with GABA_A_ *α*5 and *α* actin proteins. The fourth panel is a control omitting primary antibodies but including nuclear DAPI staining [66–75].

**Figure 27:**
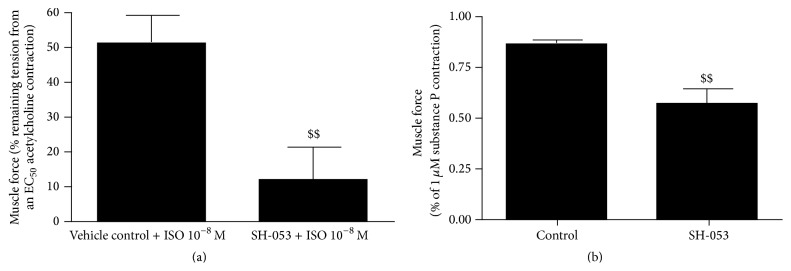
SH-053-2′F-R-CH_3_ (**2**) mediated activation of *α*5 subunit containing GABA_A_ channels induces relaxation of precontracted airway smooth muscle. (a) SH-053-2′F-R-CH_3_ (**2**) (SH-053) potentiates *β*-agonist-mediated relaxation of human airway smooth muscle. Cotreatment of human airway smooth muscle strips with SH-053-2′F-R-CH_3_ (**2**) (50 *μ*M) significantly enhances isoproterenol (10 nM) mediated relaxation of an acetylcholine EC_50_ contraction compared to isoproterenol alone (*N* = 8/group, $$ = *p* < 0.01). Modified from [66–75]. (b) SH-053-2′F-R-CH_3_ (**2**) activation of *α*5 containing GABA_A_ receptors induces direct relaxation of substance P-induced airway smooth muscle contraction. Compiled results demonstrating enhanced spontaneous relaxation (expressed as % remaining force at 30 minutes following a 1 *μ*M substance P mediated contraction) following treatment with SH-053-2′F-R-CH_3_ (**2**) compared to treatment with vehicle control (*n* = 4-5/group, $$ = *p* < 0.01) [66–75].

After demonstrating the protein expression of GABA_A_ receptors containing the *α*5 subunit, functional studies of isolated airway smooth muscle were performed in tracheal airway smooth muscle from two species. Human airway smooth muscle suspended in an organ bath was precontracted with a concentration of acetylcholine that was the EC_50_ concentration of acetylcholine for each individual airway smooth muscle preparation. The induced contraction was then relaxed with a *β*-agonist (isoproterenol) in the absence or presence of the GABA_A_ *α*5 ligand SH-053-2′F-R-CH_3_ (**2**).  Figure 27(a) shows that the amount of relaxation induced by 10 nM isoproterenol was significantly increased if 50 *μ*M SH-053-2′F-R-CH_3_ (**2**) was also present in the buffer superfusing the airway smooth muscle strip. Studies were also performed in airway smooth muscle from another species, guinea pig, that measured direct relaxation of a different contractile agonist, substance P. As shown in Figure 27(b), the amount of remaining contractile force 30 minutes after a substance P-induced contraction was significantly reduced in airway smooth muscle tracheal rings treated with SH-053-2′F-R-CH_3_ (**2**) [66–75].

Following these studies in intact airway smooth muscle, cell based studies were initiated in cultured human airway smooth muscle cells to directly measure plasma membrane chloride currents and the effects of these currents on intracellular calcium concentrations. SH-053-2′F-R-CH_3_ (**2**) induced a Cl^−^ current* in vitro* using conventional whole cell patch clamp techniques [66–75]. These electrophysiology studies were then followed by studies to determine the effect of these plasma membrane chloride currents on intracellular calcium concentrations following treatment of human airway smooth muscle cells with a ligand whose receptor couples through a Gq protein pathway, a classic signaling pathway that mediates airway smooth muscle contraction.

**Figure 28:**
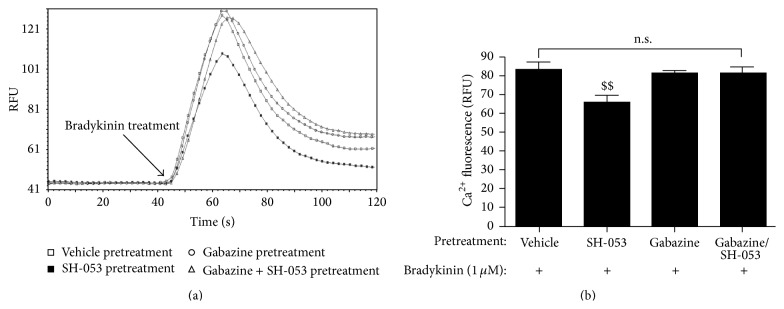
SH-053-2′F-R-CH_3_ (**2**) mediated activation of *α*5 containing GABA_A_ receptors attenuates bradykinin-induced elevations in cytosolic Ca^2+^ in human airway smooth muscle cells. (a) Representative Fluo-4 Ca^2+^ fluorescence (RFU) tracing illustrating pretreatment with SH-053-2′F-R-CH_3_ (**2**) (SH-053) (10 *μ*M) reduces cytosolic Ca^2+^ response to bradykinin (1 *μ*M). This effect is reversed in the presence of gabazine (200 *μ*M, GABA_A_ receptor antagonist). Modified from [66–75]. (b) Compiled results illustrating SH-053-2′F-R-CH_3_ (**2**) pretreatment of GABA_A_ *α*5 receptors on human airway smooth muscle cells attenuates bradykinin-induced elevations in intracellular Ca^2+^ compared to levels achieved following pretreatment with vehicle control ($$ = *p* < 0.01). While gabazine-mediated blockade of GABA_A_ channels does not significantly affect bradykinin-induced intracellular calcium increase compared to vehicle control, gabazine treatment did reverse SH-053-2′F-R-CH_3_ (**2**) ability to attenuate bradykinin-induced elevations in intracellular calcium thereby illustrating a GABA_A_ channel specific effect (n.s. = not significant). Modified from [66–75].

SH-053-2′F-R-CH_3_ (**2**) attenuated an increase in intracellular calcium concentrations induced by a classic Gq-coupled ligand, bradykinin (Figure 28(a)) [66–75]. The attenuation by SH-053-2′F-R-CH_3_ (**2**) was significantly blocked by the GABA_A_ antagonist gabazine (Figure 28(b)) indicating that SH-053-2′F-R-CH_3_ (**2**) was modulating GABA_A_ receptors for these effects on cellular calcium [66–75].

The major findings of these studies are that human airway smooth muscle expresses *α*5 subunit containing GABA_A_ receptors that can be pharmacologically targeted by a selective agonist. The GABA_A_ *α*5 subunit selective ligand SH-053-2′F-R-CH_3_ (**2**) relaxed intact guinea pig airway smooth muscle contracted with substance P and augmented *β*-agonist-mediated relaxation of intact human airway smooth muscle. The mechanism for these effects was likely mediated by plasma membrane chloride currents that contributed to an attenuation of contractile-mediated increases in intracellular calcium, a critical event in the initiation and maintenance of airway smooth muscle contraction [66–75].

## 8. Recent Discovery of Alpha 5 Included Volume Differences:* L*_4_ Pocket as Compared to Other Bz/GABAergic Subtypes

**Figure 29:**
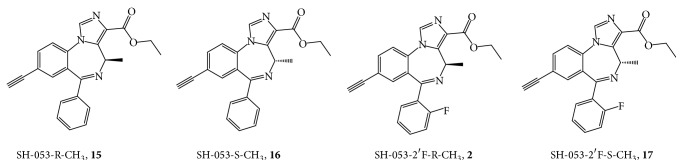
Structures of enantiomers with 2′H (**15**,** 16**) and 2′F (**2**,** 17**).

The findings in both the MAM-model of schizophrenia and the relaxation of airway smooth muscle have led to the study of SH-053-2′F-R-CH_3_ and related compounds bound within the *α*5-GABA_A_/BzR (Figure 29). The SH-053-R-CH_3_ (**15**) and SH-053-S-CH_3_ (**16**) isomers have been previously described [23]. These compounds along with SH-053-2′F-R-CH_3_ and SH-053-2′F-S-CH_3_ have been tested for binding affinity and show selectivity for the *α*5-subunit (Table 3).

**Figure 30:**
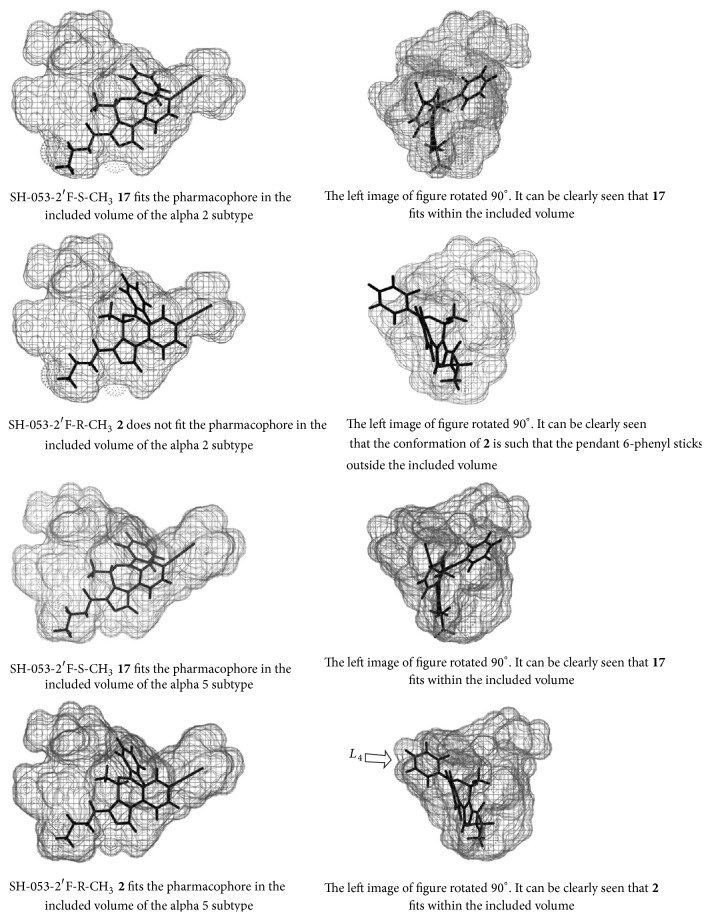
Included volume and ligand occupation of the SH-053-2′F-S-CH_3_ **17** and SH-053-2′F-R-CH_3_ **2** enantiomers in the *α*5 and *γ*2 pharmacophore/receptor models. This figure was modified and reproduced from that reported by Clayton et al. in [22, 23].

From examination of Figure 30 and Tables 3 and 4, it is clear the (R)-isomers bound to the *α*5 subtype while the (S)-isomers were selective for *α*2/*α*3/*α*5 subtypes.

From this data, these compounds were then used in examining the *α*5-binding pocket, most specifically the fluoroseries. In regard to molecular modeling, depicted in Figure 30 is the included volume and ligand occupation of the SH-053-2′F-S-CH_3_ (**17**) and SH-053-2′F-R-CH_3_ (**2**) enantiomers in the *α*5 subtype as well as the *α*2 subtype. It is clear a new pocket (*L*_4_) has been located in the *α*5 subtype permitting** 2** as well as** 17** to bind to the *α*5 subtype. Examination of both ligands in the *α*2 subtype clearly illustrates the analogous region in the *α*2 subtype is not present and thus does not accommodate** 2** for the pendant phenyl which lies outside the included volume in the space allocated for the receptor protein itself [23].

## 9. BzR GABA(A) Subtypes

In terms of potency, examination of the values in Table 4 [87], it is clear the R-isomer (**2**) shows more selectivity towards the *α*5-subunit, while the S-isomer (**17**) is potent at the *α*2/3/5 subunits. It is important, as postulated earlier [23], that the major difference in GABA(A)/Bz receptors subtypes stems from differences in asymmetry in the lipophilic pockets *L*_1_, *L*_2_, *L*_3_, *L*_4_, and *L*_Di_ in the pharmacophore/receptor model and indicates even better functional selectivity is possible with asymmetric BzR ligands.

The synthetic switching of chirality at the C-4 position of imidazobenzodiazepines to induce subtype selectivity was successful. Moreover, increase of the potency of imidazobenzodiazepines can be achieved by substitution of the 2′-position hydrogen atom with an electron rich atom (fluorine) on the pendant phenyl ring in agreement with Haefely et al. [88], Fryer [89, 90], and our own work [22, 91]. The biological data on the two enantiomeric pairs of benzodiazepine ligands confirm the ataxic activity of BZ site agonists is mediated by *α*1*β*2/3*γ*2 subtypes, as reported in [23, 91–93]. The antianxiety activity in primates of the S isomers was preserved with no sedation. In only one study in rodents was any sedation observed; the confounding sedation was observed in both the S isomer (functionally selective for *α*2, *α*3, and *α*5 receptor subtypes) and R isomer (essentially selective for *α*5 subtype) and may involve at least, in part, agonist activity at *α*5 BzR subtypes. There are some *α*5 BzR located in the spinal cord which might be the source of the decrease in locomotion with SH-053-2′F-R-CH_3_ and SH-053-2′F-S-CH_3_; however, this is possibly some type of stereotypical behavior. Hence in agreement with many laboratories including our own [23, 92, 93] the best potential nonsedative, nonamnesic, antianxiety agents stem from ligands with agonist efficacy at *α*2 subtypes essentially silent at *α*1 and *α*5 subtypes (to avoid sedation) [91]. It must be pointed out again; however, in primates Fischer et al. [87] observed a potent anxiolytic effect with no sedation with the 2′F-S-CH_3_ (**17**) isomer, while the 2′F-R-CH_3_ (**2**) isomer exhibited only a very weak anxiolytic effect.

Numerous groups have done modeling and SAR studies on different classes of compounds which have resulted in a few different pharmacophore models based on the benzodiazepine binding site (BS) of the GABA_A_ receptor [94]. These models are employed to gain insight in the interactions between the BS and the ligand. These have been put forth by Loew [7, 95, 96], Crippen [97, 98], Codding [76, 77, 99–101], Fryer [89, 90, 94], Gilli and Borea [102–105], Tebib et al. [106], and Gardner [107], as well as from Professors Sieghart, Cromer, and our own laboratory [21, 39, 40, 76, 78–82, 108–118].

**Figure 31:**
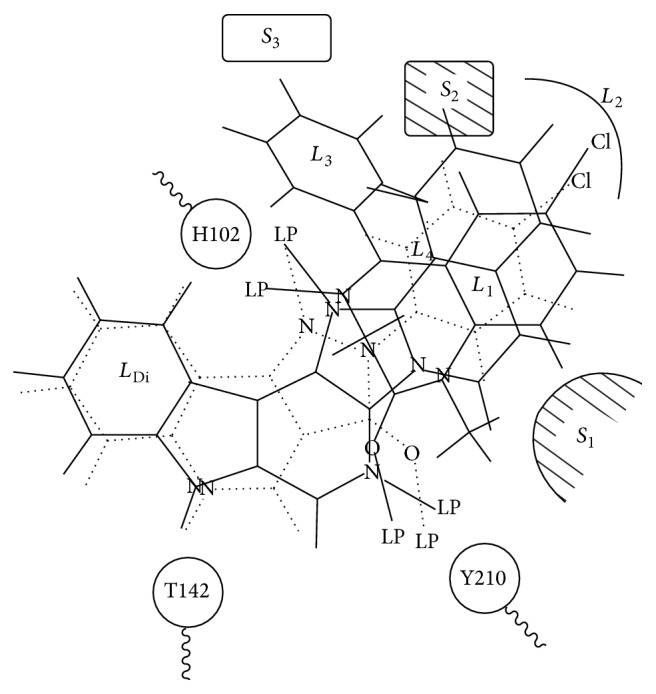
The two-dimensional representation of the Milwaukee-based unified pharmacophore with 3 amino acids in the binding site based on the rigid ligand template [23, 39, 42, 76–80]. This figure has been modified from that reported for PAMs, NAMs, and antagonists in [22, 23].

The Milwaukee-based pharmacophore/receptor model is a comprehensive building of the BzR using radioligand binding data and receptor mapping techniques based on 12 classes of compounds [20, 23, 39, 40, 42, 111, 119–122]. This model (Figure 31) [79] has brought together previous models which have used data from the activity of antagonists, positive allosteric modulators, and negative allosteric modulators and included the new models for the “diazepam-insensitive” (DI) sites [123]. Four basic anchor points, *H*_1_, *H*_2_, *A*_2_, and *L*_1_, were assigned, and 4 additional lipophilic regions were defined as *L*_2_, *L*_3_, *L*_Di_, and the new *L*_4_ (see captions in Figure 31 for details); regions *S*_1_, *S*_2_, and *S*_3_ represent negative areas of steric repulsion. As previously reported, the synthesis of both partial agonists and partial inverse agonists has been achieved by using parts of this model [99, 100, 104, 105, 119, 124–127].

The cloning, expression, and anatomical localization of multiple GABA(A) subunits have facilitated both the identification and design of subtype selective ligands. With the availability of binding data from different recombinant receptor subtypes, affinities of ligands from many different structural classes of compounds have been evaluated.

Illustrated in Figure 31 is the [*3,4-c*]quinolin-3-one CGS-9896 (**18**) (dotted line), a diazadiindole (**19**) (thin line), and diazepam (**20**) (thick line) fitted initially to the inclusive pharmacophore model for the BzR. Sites *H*_1_ (Y210) and *H*_2_ (H102) represent hydrogen bond donor sites on the receptor protein complex while *A*_2_ (T142) represents a hydrogen bond acceptor site necessary for potent inverse activity* in vivo*. *L*_1_, *L*_2_, *L*_3_, *L*_4_, and *L*_Di_ are four lipophilic regions in the binding pharmacophore. Descriptors *S*_1_, *S*_2_, and *S*_3_ are regions of negative steric repulsion.

**Figure 32:**
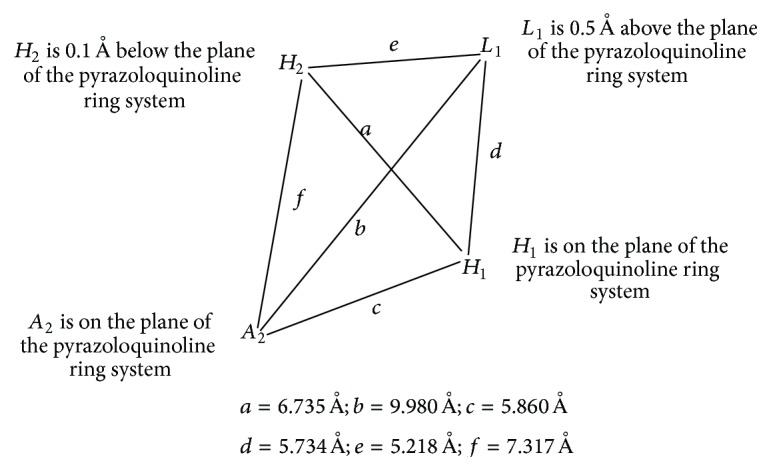
The schematic representation of the descriptors for the initial inclusive BzR pharmacophore based on the rigid ligands (diindoles) [79, 81–84]. This figure has been modified from that reported in [79].

Based on SAR data obtained for these ligands at 6 recombinant BzR subtypes [128–132], an effort has been undertaken to establish different pharmacophore/receptor models for BzR subtypes. The alignment of the twelve different structural classes of benzodiazepine receptor ligands was earlier based on the least squares fitting of at least three points. The coordinates of the four anchor points (*A*_2_, *H*_1_, *H*_2_, and *L*_1_) employed in the alignment are outlined in Figure 32. Herein are described the results from ligand-mapping experiments at recombinant BzR subtypes of 1,4-benzodiazepines, imidazobenzodiazepines, *β*-carbolines, diindoles, pyrazoloquinolinones, and others [126]. Some of the differences and similarities among these subtypes can be gleaned from this study and serve as a guide for future drug design.

## 10. ***α***1 Updates

### 10.1. Beta-Carbolines

**Figure 33:**
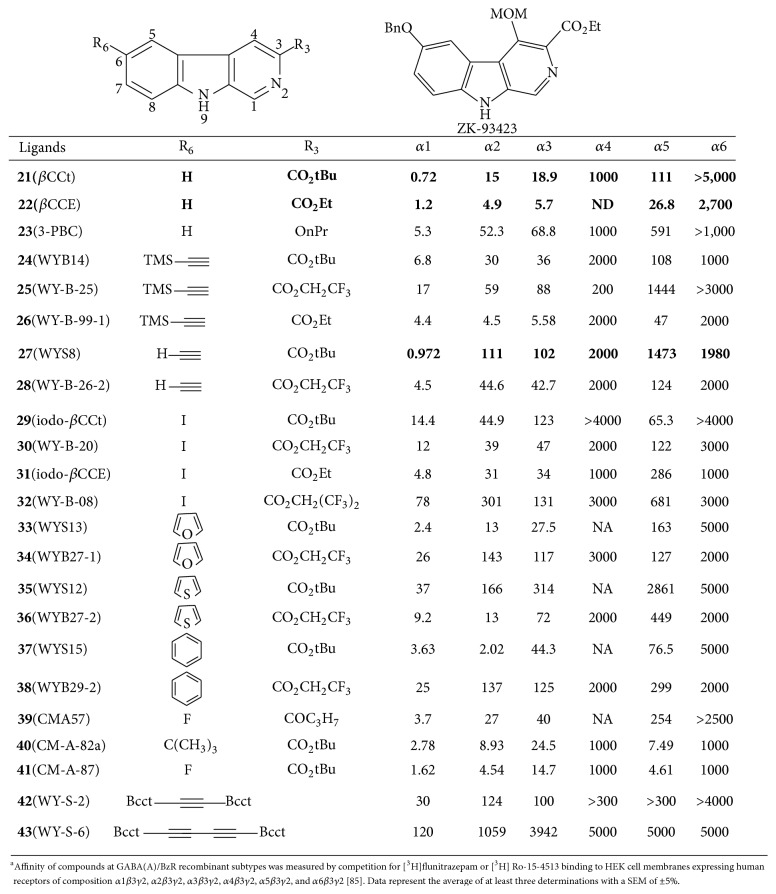
^a^Affinities (*K*_*i*_ = nM) of 3,6-disubstituted *β*-carbolines at *αxβ*3*γ*2 (*x* = 1–3, 5, 6) receptor subtypes [85]. The structures versus code numbers of all ligands in the tables of this review can be found in the Ph.D. thesis of Terry Clayton (Ph.D. thesis, University of Wisconsin-Milwaukee, Milwaukee, WI, December, 2011) [22] and in the Supporting Information.

A series of 3,6-disubstituted *β*-carbolines was prepared and evaluated for their* in vitro* affinity at *αxβ*3*γ*2 GABA(A)/BzRr subtypes by radioligand binding assays in search of *α*1*β*3*γ*2 subtype selective compounds (Figure 33). A potential therapeutic application of such antagonist analogs is to treat alcohol abuse [133, 134]. Analogues of *β*CCt (**21**) were synthesized* via* a carbonyldiimidazole-mediated method by Yin et al. [85] and the related 6-substituted *β*-carboline-3-carboxylates including WYS8 (**27**) were synthesized from 6-iodo *β*CCt (**29**). Bivalent ligands (**42** and** 43**) were also synthesized to increase the scope of the structure-activity relationships (SAR) to larger ligands. An initial SAR on the first analogs demonstrated that compounds with larger side-groups at C6 were well tolerated as they projected into the *L*_Di_ domain (see** 42** and** 43**) [85]. Moreover, substituents located at C3 exhibited a conserved stereo interaction in lipophilic pocket *L*_1_, while N2 likely participated in hydrogen bonding with *H*_1_. Three novel *β*-carboline ligands (**21**,** 23**, and** 27**) permitted a comparison of the pharmacological properties with a range of classical benzodiazepine receptor antagonists (flumazenil, ZK93426) from several structural groups and indicated these *β*-carbolines were “near GABA neutral antagonists.” Based on the SAR, the most potent (*in vitro*) *α*1 selective ligand was the 6-substituted acetylenyl *β*CCt (WYS8,** 27**). In a previous study both** 21** and** 23** were able to reduce the rate at which rats self-administrated alcohol in alcohol preferring and HAD rats but had little or no effect on sucrose self-administration [85]. 3-PBC (**23**) was also active in baboons [134]. This data has been used in updating the pharmacophore model in the *α*1-subtype.

## 11. The Updated Included Volume Models

**Figure 34:**
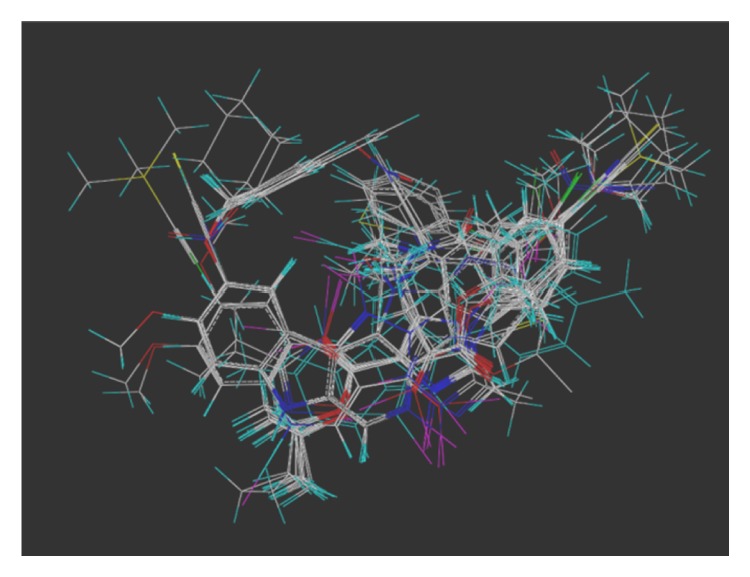
Overlay of selected compounds for *α*1*β*3*γ*2 subtype from Table 5.

Illustrated in Figure 34 is the included volume of the updated pharmacophore receptor model of the *α*1*β*3*γ*2 subtype of Clayton [22]. The current model for the *α*1*β*3*γ*2 subtype has several new features. The cyclopropyl group of CD-214 extended 2 Å past the *A*_2_ descriptor slightly increasing its volume. The trimethylsilyl group of QH-II-82 and WYS7 illustrates how well bulky groups are tolerated near the entrance of the binding pocket. Despite not being as potent, dimers of beta carbolines, WYS2 and WYS6, bound to *α*1 subtypes at 30 nM and 120 nM, respectively. Their ability to bind, albeit weakly, supports the location of the binding site entrance from the extracellular domain. The included volume of the *α*1*β*3*γ*2 subtype was previously 1085.7 cubic angstroms. The volume has now been measured as 1219.2 cubic angstroms. Volume measurements should be used carefully as the binding site is not enclosed and the theoretical opening near *L*_DI_ is not clearly demarcated. Dimers were excluded from the included volume exercise because although they bound to the receptor, they represented compounds which were felt to extend outside the receptor binding pocket when docked to the protein. Where appropriate, their monomers were included in the included volume analysis. Ligands considered for the included volume in Table 5 exhibited potent binding at *α*1 subtypes (*K*_*i*_ ≤ 20 nM) but were not necessarily subtype selective. The binding data for ligands at *α*_2–6_-subtypes follow (Tables 6–10; structures located in Clayton [22] and Supporting Information, Appendix III in Supplementary Material available online at http://dx.doi.org/10.1155/2015/430248).

## 12. The ***α***1***β***3***γ***2 Receptor Subtype

**Figure 35:**
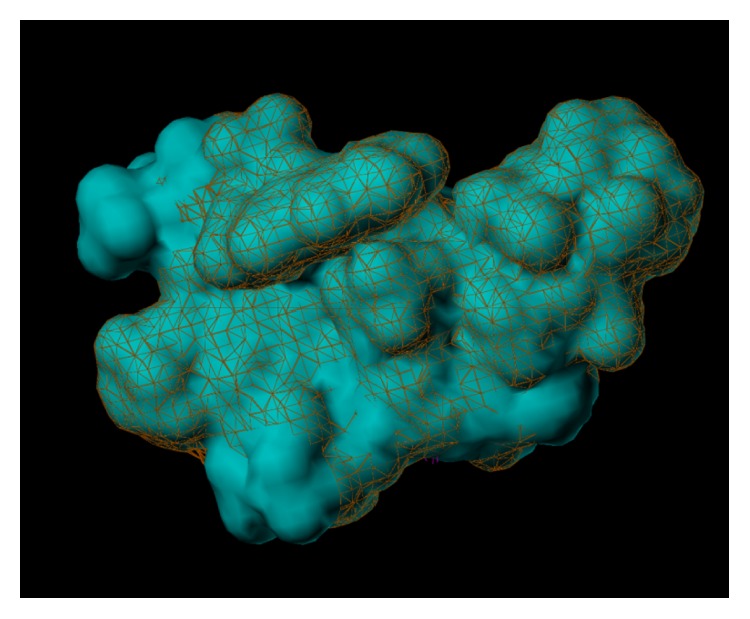
Updated *α*1*β*3*γ*2 subtype (blue solid) overlaid with the previous model (red wire). Overlap identified where wire and solid overlap.

The focus of this research was aimed at diazepam sensitive receptors; additional features to the *α*4*β*3*γ*2 and *α*6*β*3*γ*2 receptors were not identified (see Table 5, Figures 34 and 35). The major new feature identified for the *α*5*β*3*γ*2 receptor was a new *L*_4_ pocket. This new lipophilic pocket was identified with SH-053-R-CH_3_ (**15**) and SH-053-S-CH_3_ (**16**) chiral enantionmers as well as the 2′F analogs [74, 135, 136].

## 13. The ***α***2***β***3***γ***2 Receptor Subtype

**Figure 36:**
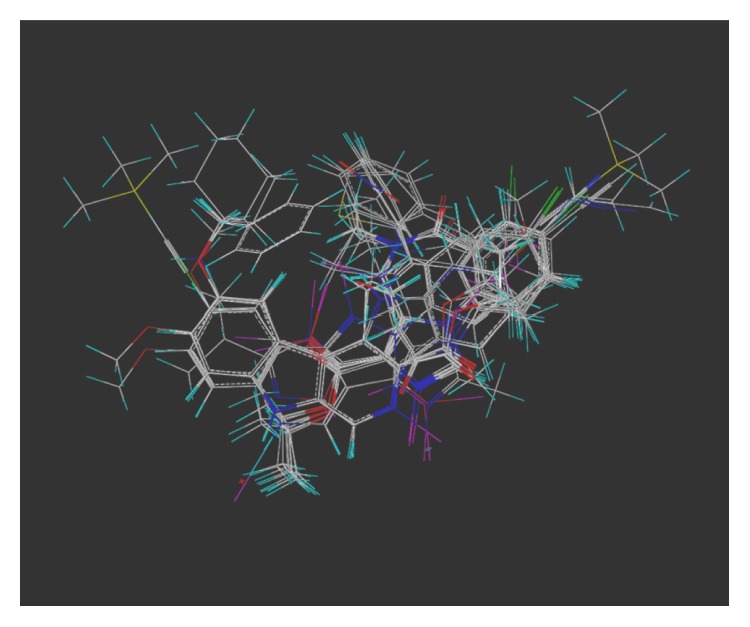
Overlay of compounds selective for *α*2*β*3*γ*2 subtype.

**Figure 37:**
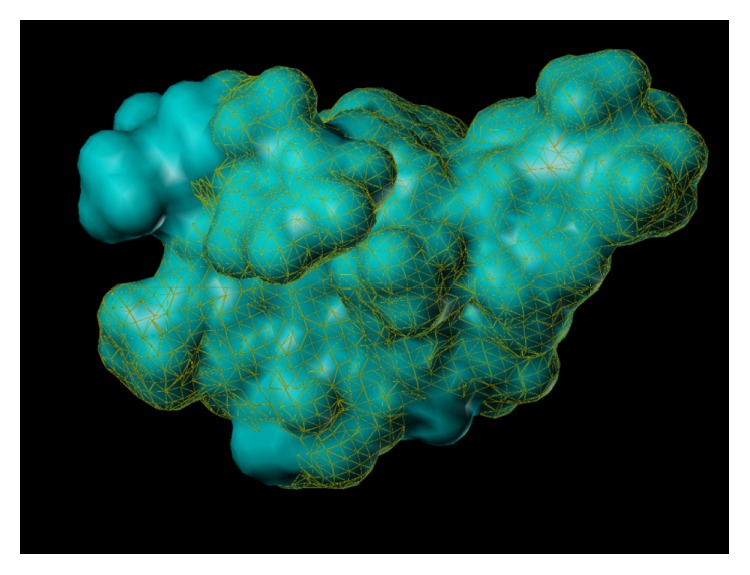
Updated *α*2*β*3*γ*2 subtype (solid) overlaid with the previous model (red wire). Overlap identified where wire and solid overlap.

See Table 6 and Figures 36 and 37.

## 14. The ***α***3***β***3***γ***2 Receptor Subtype

**Figure 38:**
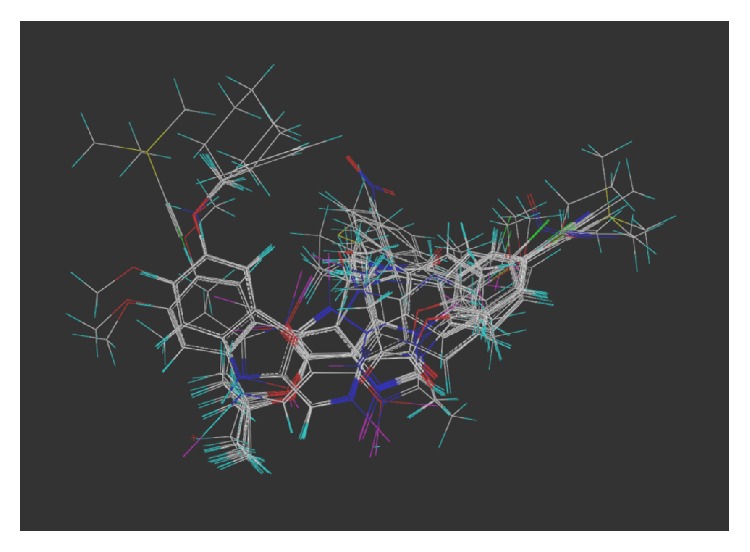
Overlay of compounds selective for *α*3*β*3*γ*2 subtype.

**Figure 39:**
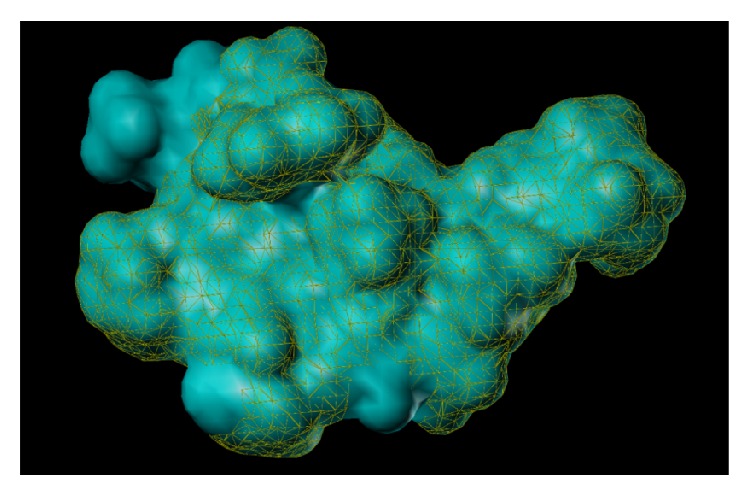
Updated *α*3*β*3*γ*2 subtype (blue solid) overlaid with the previous model (red wire). Overlap identified where wire and solid overlap.

See Table 7 and Figures 38 and 39.

## 15. The ***α***4***β***3***γ***2 Receptor Subtype

**Figure 40:**
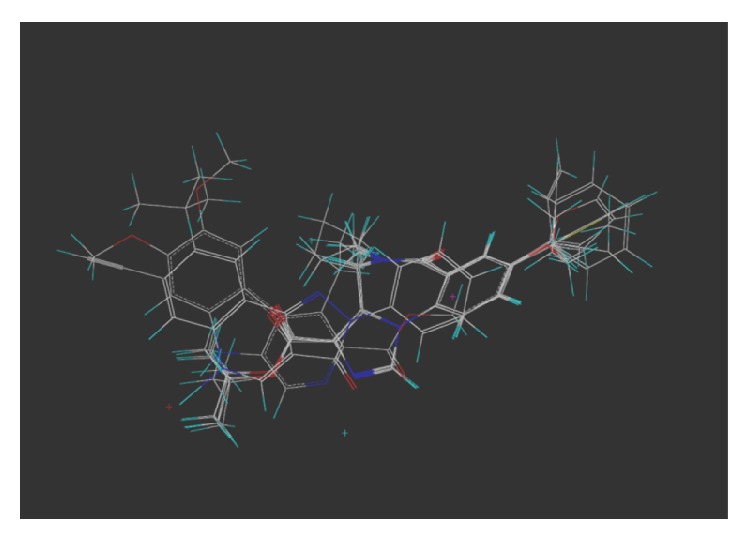
Overlay of selected compounds selective for *α*4*β*3*γ*2 subtype.

**Figure 41:**
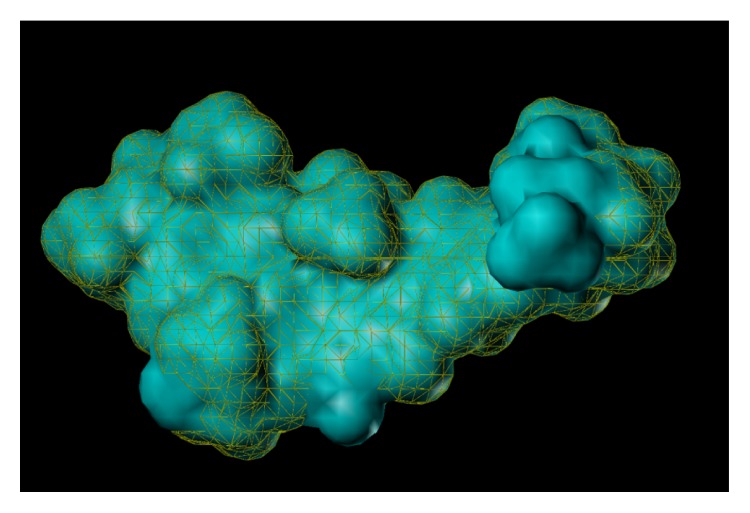
Updated *α*4*β*3*γ*2 subtype (blue solid) overlaid with the previous model (yellow wire). Overlap identified where wire and solid overlap.

See Table 8 and Figures 40 and 41.

## 16. The ***α***5***β***3***γ***2 Receptor Subtype

**Figure 42:**
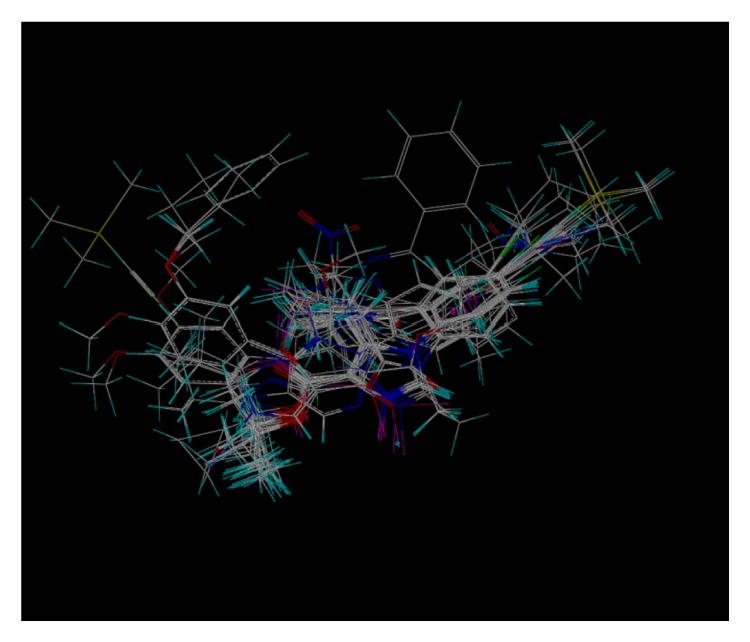
Overlay of selected compounds selective for *α*5*β*3*γ*2 subtype.

**Figure 43 fig43:**
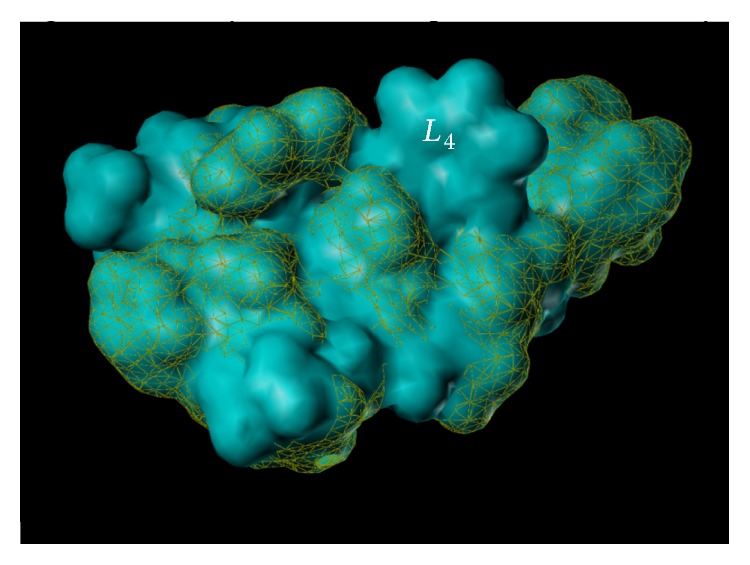
Updated *α*5*β*3*γ*2 subtype (blue solid) overlaid with the previous model (yellow wire). Overlap identified where wire and solid overlap.

**Figure 44 fig44:**
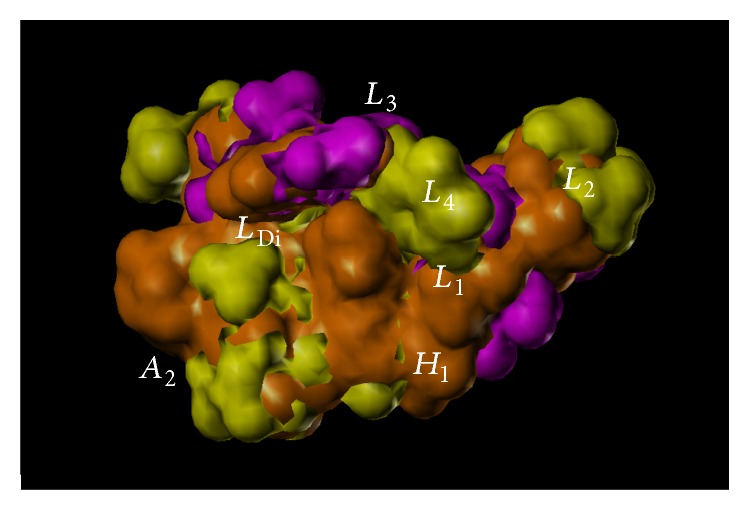
Overlay of the *α*5*β*3*γ*2 receptor (yellow) subtype with the *α*1*β*3*γ*2 receptor (magenta) subtype. Orange surfaces indicate overlapping regions.

**Figure 45:**
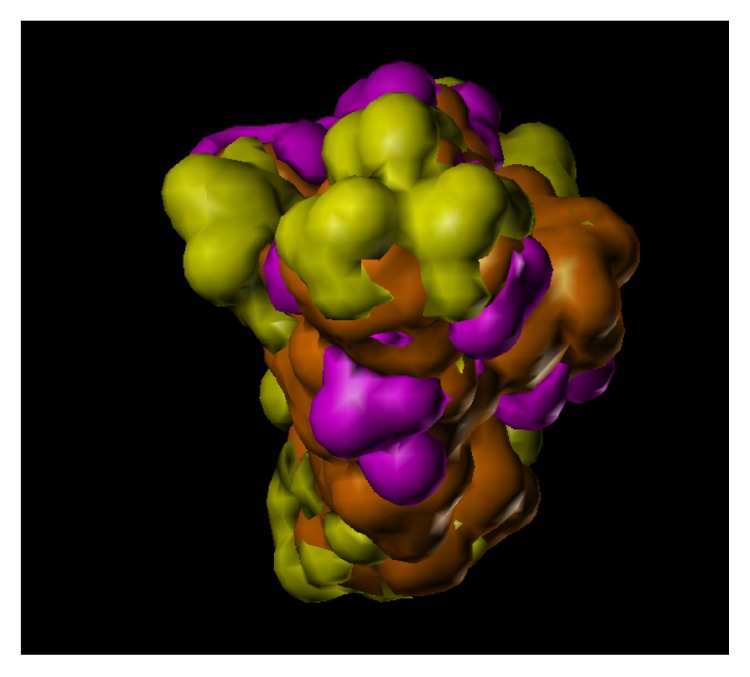
Overlay of the *α*5*β*3*γ*2 receptor (yellow) subtype with the *α*1*β*3*γ*2 receptor (magenta) subtype (Figure 44 rotated 90°). Orange surfaces indicate overlapping regions.

**Figure 46:**
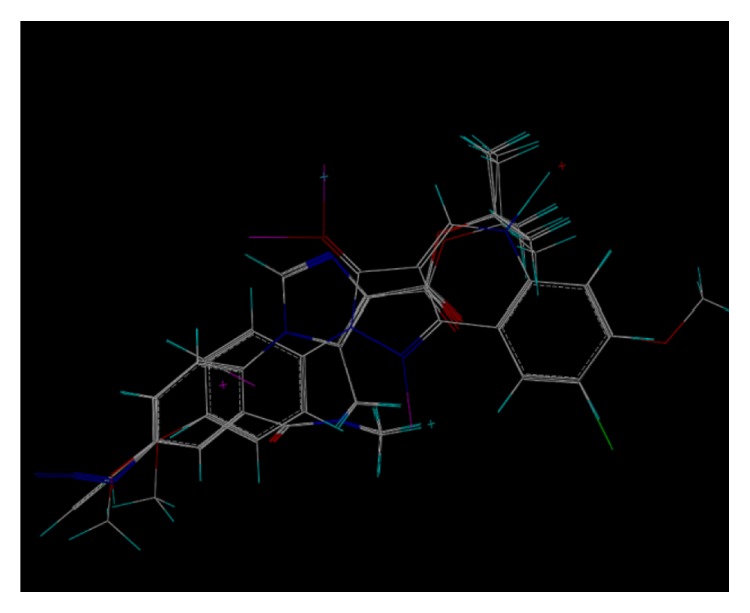
Overlay of selected compounds selective for *α*6*β*3*γ*2 subtype.

**Figure 47:**
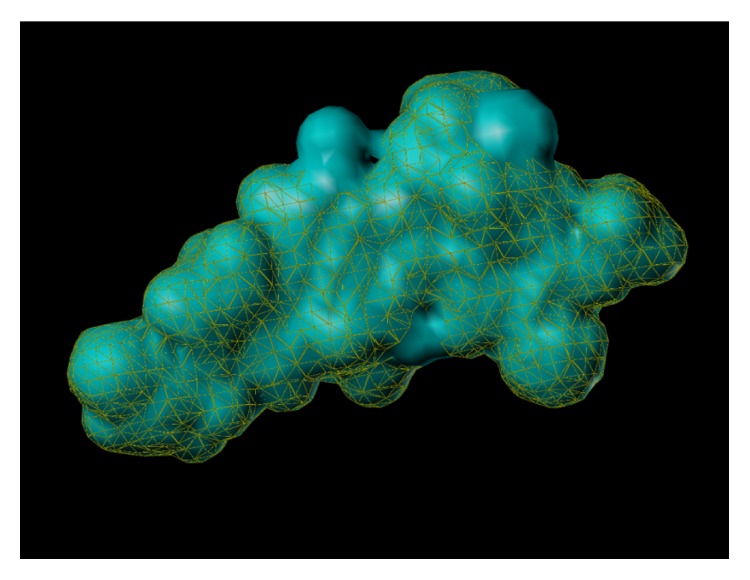
Updated *α*6*β*3*γ*2 subtype (blue solid) overlaid with the previous model (yellow wire). Overlap identified where wire and solid overlap.

The multiple volume contours displayed in Figures 34–47 were created using the mvolume function (multiple volume contour function) in Sybyl and compounds with binding affinity at the receptor less than or equal to 20 nM. To create the overlays, first, the display (dsp) and contour (cnt) files were created for the *α*5*β*3*γ*2 receptor subtype and the *α*1*β*3*γ*2 receptor subtype by overlaying the compounds for each of these receptors (see Table 9 and Figures 42 [Fig fig43] [Fig fig44]–45). Using the mvolume function, a logical expression was entered to create the surfaces making up the union as well as the included volume for each receptor subtype itself. It is clear from the included volume overlay that the *L*_2_ pocket is deeper for the *α*5 subtype, as determined previously [13, 21–23, 110, 119]. The new *L*_4_ pocket can be distinguished as the new yellow region of the *α*5*β*3*γ*2 subtype which is due to recently designed R-isomers by Huang [135], Poe and Li.

## 17. The ***α***6***β***3***γ***2 Receptor Subtype

See Table 10 and Figures 46 and 47.

## 18. Updates to the Previous Model

**Figure 48:**
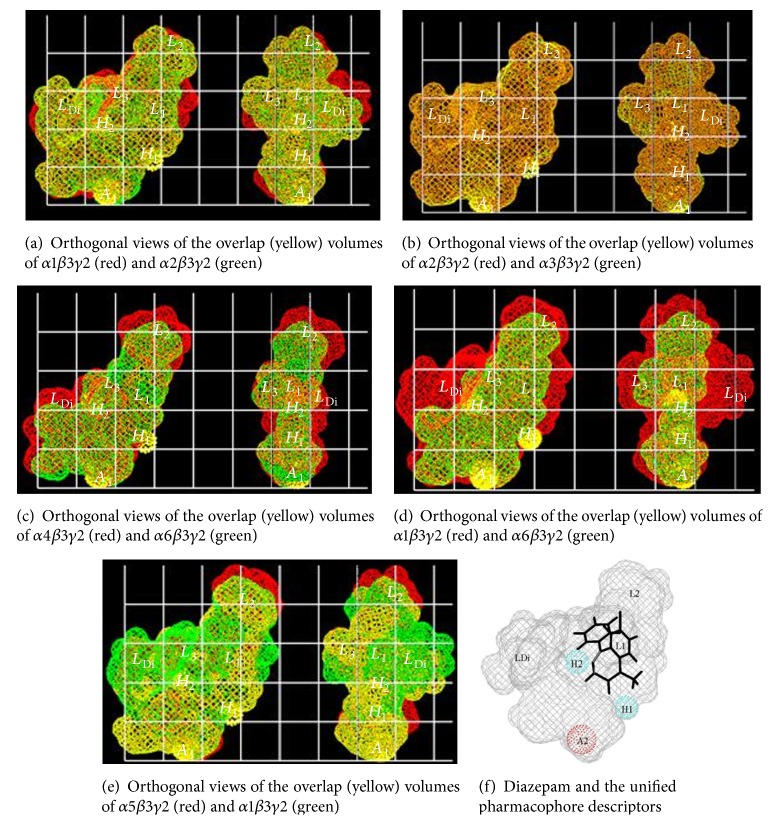
The previous benzodiazepine subtype selective receptor pharmacophore models [23]. (1) The *L*_2_ region in the *α*5 subtype is larger than the *α*1 subtypes. This is a key result. It is the principle difference between *α*5 subtypes compared to *α*2 and *α*3 subtypes, but especially in regard to *α*1 subtypes (*L*_2_ smaller in *α*1). (2) The *L*_3_ region is larger in the *α*5 subtype as compared to the *α*1, *α*2, *α*3, *α*4, and *α*6 BzR sites. R analogs of benzodiazepines with pendant phenyls had increased affinity to *α*5 supporting the larger *L*_3_ pocket in this receptor subtype, while S isomers bound to *α*2, *α*3, and *α*5 subtypes because of different conformational constraints.

In addition to the newly discovered *L*_4_ pocket, the updated library of binding affinity led to two specific updates in the previous model (Figure 48).

## 19. QSAR

A nontraditional quantitative structure activity relationship (QSAR) approach was executed to observe steric and electrostatic preferences for each receptor subtype. A subset of the compounds used in each subtype pharmacophore/receptor model were chosen with a good cross section of scaffold variety. The compounds used in the COMFA maps are the imidazobenzodiazepines published previously [110, 137] and additionally alternative scaffolds which bound with <20 nM at the respective subtype [22].

The interest here was in creation of steric and electrostatic maps of the comparative molecular field analyses (COMFA) created from molecular spreadsheets. A variety of compounds selective for each subtype were selected and placed into a dataset used to build the CoMFA models. Activities (*K*_*i*_ values) were converted to logarithmic units for this study. A CoMFA descriptor set was created based on the –log (*K*_*i*_) of over 70 structures. The goal was to derive an alternative three-dimensional shape of the receptor using biological activity of the most selective compounds. Structures were determined by crystal structure where available or by calculation. Charges were provided based on the Gasteiger-Huckel model. Conformations were kept consistent based on previous studies of low energy conformations [110]. It should be noted that this was not a traditional QSAR study as nonselective compounds were excluded. Therefore, *K*_*i*_ values did not cross 3 log units. This was acceptable since the goal was not to create a predictive QSAR predictive algorithm, rather a map of the receptor based on sterics and electrostatics. Hydrogen acceptor radii were set to 3.0 and the hydrogen donor radii were set to 2.6 based on recommendations from Certara (Tripos). Analyses were executed using PLS (partial least squares). The details of modeling will be further discussed in the SI.

For each of the following QSAR models (Figures 49 [Fig fig50] [Fig fig51] [Fig fig52] [Fig fig53] [Fig fig54] [Fig fig55] [Fig fig56] [Fig fig57] [Fig fig58] [Fig fig59] [Fig fig60] [Fig fig61] [Fig fig62] [Fig fig63] –64), green areas represent desirable steric bulk and yellow represents undesirable steric bulk. Positive electrostatic contributions are represented by blue and negative electrostatic contributions are represented by red.

## 20. The ***α***1***β***3***γ***2 Receptor Subtype

See Figures 49–52.

## 21. The ***α***2***β***3***γ***2 Receptor Subtype

See Figures 53–56.

## 22. The ***α***3***β***3***γ***2 Receptor Subtype

See Figures 57–60.

## 23. The ***α***5***β***3***γ***2 Receptor Subtype

**Figure 61 fig61:**
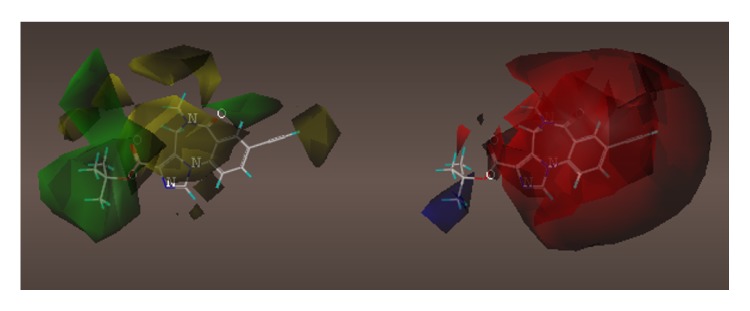
Steric (left) and electrostatic maps of the *α*5*β*3*γ*2 receptor subtype shown in the transparent mode as seen from the classic perspective.

**Figure 62 fig62:**
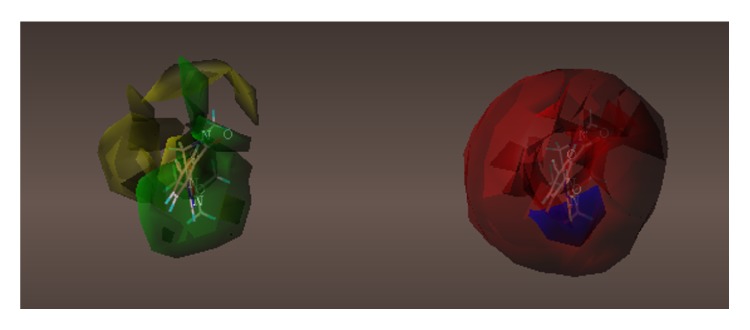
Steric (left) and electrostatic maps of the *α*5*β*3*γ*2 receptor subtype shown in the transparent mode as seen from the classic perspective rotated 90°.

**Figure 63 fig63:**
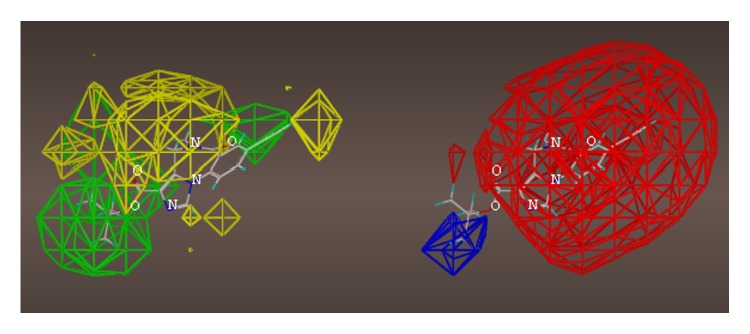
Steric (left) and electrostatic maps of the *α*5*β*3*γ*2 receptor subtype shown in line mode as seen from the classic perspective.

**Figure 64:**
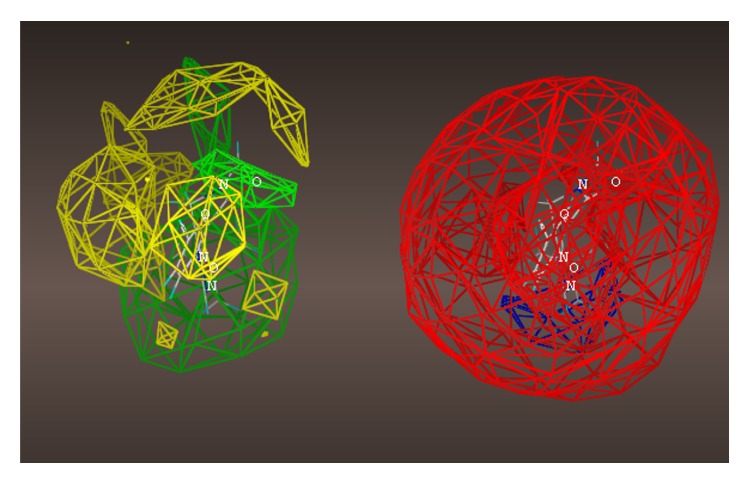
Steric (left) and electrostatic maps of the *α*5*β*3*γ*2 receptor subtype shown in line mode as seen from the classic perspective rotated 90°.

**Figure 65:**
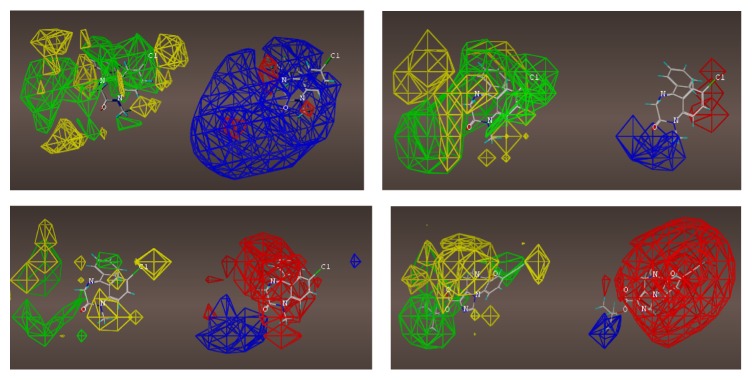
Clockwise from the top left, line maps of the *α*1*β*3*γ*2, *α*2*β*3*γ*3, *α*3*β*3*γ*2, and *α*5*β*3*γ*2 CoMFA.

From the CoMFA maps several observations (Figure 65) can be made. The yellow steric regions near *L*_3_ in the *α*5*β*3*γ*2 map are unique. This illustrated that, in general, benzodiazepines lacking a pendant phenyl are more suited to targeting the *α*5 subtype. The *L*_Di_ region of the *α*1 subtype is most tolerable for compounds with steric interactions in this location while the *α*3 subtype receptor compounds prefer no steric interaction in this location. Negative electrostatics are most preferred by the *L*_3_ pocket of the *α*2 and *α*5 receptors. In general, the *α*1 subtype receptor prefers molecules without a dipole. It should be noted that none of the analogs are ionic in nature and the charges for this model were provided by the Gasteiger-Huckel model. For this reason more emphasis is placed on the steric relationships which exclude interactions in the pharmacophores. In the future a QSAR study which includes nonbinding benzodiazepines in the data set along with activity data will permit the creation of a predictive algorithm which will be very useful in lead targeting (see Figures 61–65).

## 24. Conclusion

Benzodiazepines, *β*-carbolines, and other classes of compounds readily target the GABA_A_ receptors. The difficulty is finding subtype selective ligands, since there is no crystal structure of the Bz/GABA_A_-ergic site itself, just one composed of five beta-subunits which has no Bz site to date. The *α*5-BzR/GABA_A_ subunit has recently been shown to be important in the search to treat numerous cognition-based illnesses including Alzheimer's, schizophrenia, bipolar, and depression, as well as more recently a bronchodilator, potentially important in the treatment of asthma. As an inverse agonist, PWZ-029 was able to counteract the memory-impairing effects of scopolamine, a muscarinic antagonist, in both object recognition tests and object retrieval tests in rodents, and was active in primates, as well as samaritan Alzheimer's rats. The implications of these tests point to a use as a possible treatment for Alzheimer's disease. The docking of PWZ-029 in the *α*5*γ*2 GABA_A_R-subunit details the interactions between the pharmacophore/receptor model binding site and this important negative allosteric modulator. Furthermore, *α*5-BzR/GABA_A_ positive allosteric modulator, SH-053-2′F-R-CH_3_, was shown to reverse deleterious effects in the MAM-model of schizophrenia. The recent discovery of *α*5-GABA_A_R in airway smooth muscle by Emala et al. has also lead to the testing of SH-053-2′F-R-CH_3_ as a bronchodilator. This SH-053-2′F-R-CH_3_ was found to be effective in relaxing preconstricted airway smooth muscle, as well as attenuating calcium-ion entry through the plasma membrane. In addition, XLi-093 (an *α*5 receptor antagonist), a potently binding *α*5-subtype selective bivalent ligand, has been shown to inhibit the *α*5-cognition deficits effected by diazepam and is a very good *α*5 benzodiazepine receptor site antagonist. It has also been shown to reverse the effects of *α*5 PAMs and NAMs in both rodent and primate models. These findings led to the exploration of the *α*5-binding pocket in the Milwaukee-based pharmacophore.

New features have been introduced to the unified pharmacophore/receptor model based on many substance classes that act at the diazepam sensitive and diazepam insensitive BzR binding sites of GABA_A_ receptors. The major new feature identified for the *α*5*β*3*γ*2 receptor was a new *L*_4_ pocket which was found by using pendant 6-phenyl benzodiazepines with a R-CH_3_ at the prochiral center at C4. Further enhancement of potency was achieved by addition of 2′-F or 2′-N substituent in the pendant phenyl ring at C-6. While these changes have led to enhanced subtype selective ligands, the overall development guided by this pharmacophore model described here has led to new agents with varying, fascinating pharmacological profiles, ranging from use in cognition-based diseases such as Alzheimer's and schizophrenia, to use as a bronchodilator. This research on updating the Milwaukee-based pharmacophore/receptor model can be used in the rational design for improving the selectivity of *α*5 ligands. As the library of compounds increases, the data which follows can then be further evaluated and can lead to more insight to the identification of the possible roles each individual residue may have with the binding pocket.

The X-ray structure determination of the *α*5*β*3*γ*2 GABA(A) receptor is eagerly awaited, while that with five *β*3-subunits has been reported recently (Miller and Aricescu,* Nature* 2014). It is hoped that the proposed orientation may be used by others to gain additional insight into the potential mechanisms underlying binding and modulation at the Bz site, all of which will lead to a better understanding of the structure and function of GABA(A) receptors, ultimately targeted toward treatment of diseases.

## 25. Synthesis of Ligands with ***α***5 BzR Subtype Selectivity

**Scheme 1:**
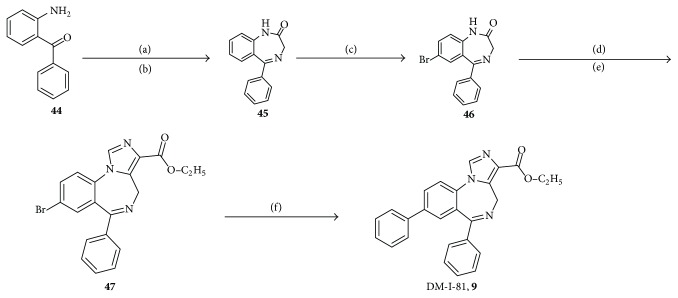
Synthesis of 8-substituted imidazobenzodiazepines following chemistry earlier developed by Sternbach, Fryer et al.* Reagents and Conditions*. (a) Bromoacetyl bromide, sodium bicarbonate, and chloroform; (b) ammonia (anhydrous), methanol, and reflux; (c) bromine, sulfuric acid, and acetic acid; (d) sodium hydride, diethyl chlorophosphate, and tetrahydrofuran; (e) sodium hydride, ethyl isocyanoacetate, and tetrahydrofuran, −30°C to r.t.; (f) tributyl(phenyl)stannane, Pd(PPh_3_)_4_.

Briefly, bromoacetyl bromide was added to 2-aminobenzophenone** 44**, followed by treatment with methanol, which had been saturated with ammonia (g) under the cooling of an ice-water bath. The benzodiazepine,** 45**, was brominated to provide** 46** and then reacted with ethyl isocyanoacetate to generate the imidazobenzodiazepine,** 47**. A much better one-pot process has now been devised using KtBuO at −30°C [140]. The bromide** 48** was subjected to a Stille-type coupling to give DM-I-81 (**9**) [126]. This route (Scheme 1) can be executed on several hundred gram scales.

**Scheme 2:**
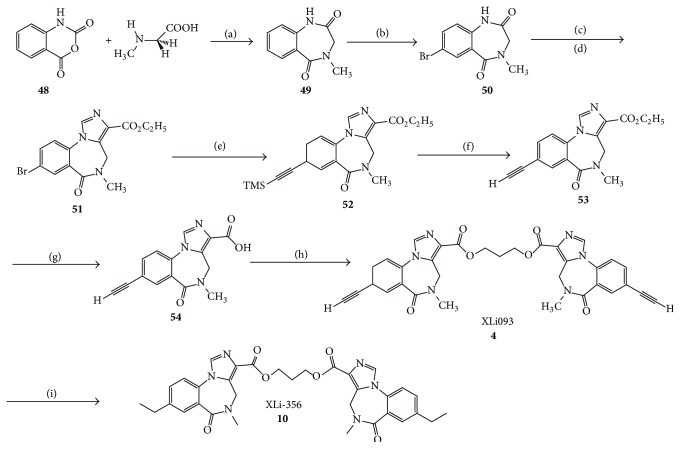
Synthesis of 8-substituted imidazobenzodiazepine bivalent ligands.* Reagents and Conditions*. (a) DMSO, 180°C, 90%; (b) bromine, sodium acetate, and acetic acid, r.t., 80%; (c) LDA, THF, and diethyl chlorophosphate, 0°C; (d) LDA, THF, and ethyl isocyanoacetate; (e) trimethylsilyl acetylene, Pd(OAc)_2_(PPh_3_)_2_, triethylamine, acetonitrile, and reflux, 80%; (f) tetrabutylammonium fluoride, THF, and H_2_O, r.t., 88%; (g) 2N NaOH and ethanol, 70°C, 90%; (h) CDI, DMF, HO(CH_2_)_3_OH, and DBU, 60%; (i) Pd/C, H_2_, ethanol, and DCM, 90%.

The benzodiazepine monomers were prepared by the method of Fryer and Gu [89, 141]. The isatoic anhydride was heated with sarcosine in dimethyl sulfoxide to provide amide** 49**. Bromination of** 49** in a mixture of acetic acid, bromine, and sodium acetate afforded the corresponding monosubstituted bromide** 50** in good yield. Deprotonation of** 50** with lithium diisopropyl amide (LDA) in THF was followed by treatment with diethyl chlorophosphate to provide the intermediate enol phosphate. The enol phosphate was stirred with a solution of ethyl isocyanoacetate and LDA to yield the imidazo congener. Again, a better one-pot procedure has been developed using KtBuO at −30°C in place of LDA at 0°C. A Heck type coupling reaction was employed with the bromide** 51** with bis(acetate)bis(triphenylphosphine)palladium(II) to provide the TMS-acetylene** 52**. Treatment of** 52** with Bu_4_NF removed the trimethylsilyl group. Hydrolysis of the ester function of** 53** provided the acid** 54** in excellent yield and this material was dried scrupulously and subjected to a standard CDI-mediated coupling reaction to furnish bivalent ligand XLi-093 (**4**). The imidazobenzodiazepine diethyl diester XLi-356 (**10**) was obtained from XLi-093 (Scheme 2) in high yield* via* catalytic hydrogenation (Pd/C, H_2_).

## 26. Synthesis of Bivalents

**Scheme 3:**
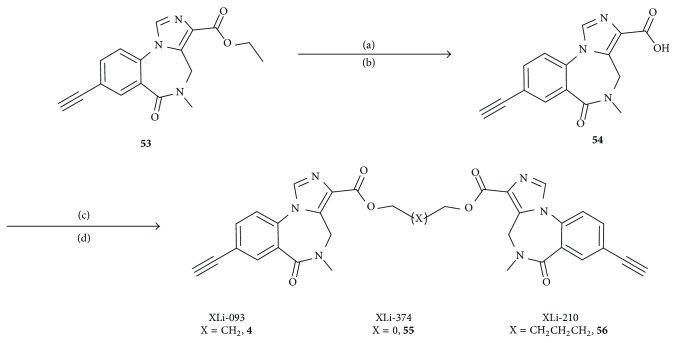
Synthesis of bivalent analogs of XLi-093 (**4**).* Reagents and Conditions*. (a) 2 M NaOH, EtOH, 70°C; (b) 10% aq HCl; (c) CDI, DMF; (d) diol, DBU.

Inverse agonist** 53** was synthesized via the reported procedure. Hydrolysis of the ester function of** 53** provided the acid** 54** in excellent yield. This material was dried scrupulously and was subjected to a standard CDI-mediated coupling reaction to furnish bivalent ligands** 4**,** 55**, and** 56** in 60% yield (Scheme 3) [13].

**Scheme 4:**
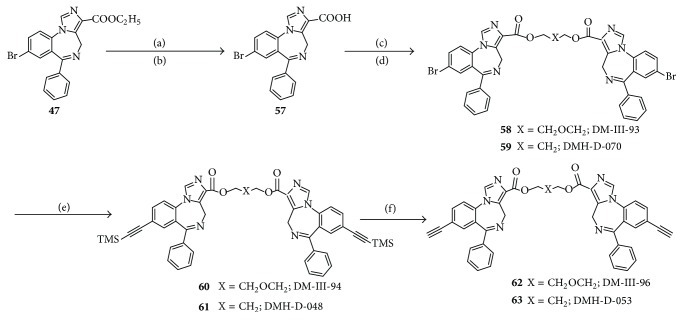
Synthesis of bivalent analogues of DMH-D-053 (**63**).* Reagents and Conditions*. (a) 2 N NaOH, EtOH, and reflux; (b) 10% aq. HCl; (c) CDI, DMF; (d) diol, DBU; (e) trimethylsilylacetylene, Pd(OAc)_2_(PPH_3_)_2_, Et_3_N, CH_3_CN, and reflux; (f) TBAF*∗*0.5H_2_O, THF, −78°C.

The acid** 57**, obtained from the ester** 47**, which was available from the literature [13], was stirred with CDI in DMF, followed by stirring with the required diol and DBU to provide bromide substituted dimers** 58** or** 59**, respectively. They were converted into the trimethylsilylacetylenyl** 60** or** 61**, respectively, under standard conditions (Pd-mediated, Heck-type coupling) [142]. The bisacetylene** 62** or** 63** (individually) was easily obtained by treatment of the trimethylsilyl ligand** 60** or** 61** with fluoride anion, as shown in Scheme 4.

## 27. Materials, Methods, and Experimental

### 27.1. Materials and General Instrumentation

Chemicals were purchased from Aldrich Chemical Co. or Tokyo Chemical Industries and were used without further purification except where otherwise noted. Anhydrous THF was distilled from sodium/benzophenone ketyl. TLC analyses were carried out on Merck Kieselgel 60 F_254_, and flash column chromatography was performed on silica gel 60b purchased from E. M. Laboratories. Melting points were taken on a Thomas-Hoover melting point apparatus or an Electrothermal Model IA8100 digital melting point apparatus and are reported uncorrected. NMR spectra were recorded on a Bruker 300 or 500 MHz multiple-probe spectrometer. Infrared spectra were recorded on a Nicolet DX FTIR BX V5.07 spectrometer or a Mattson Polaris IR-10400 instrument. Low-resolution mass spectral data (EI/CI) were obtained on a Hewlett-Packard 5985B GC-mass spectrometer, while high resolution mass spectral data were taken on a VG autospectrometer (Double Focusing High Resolution GC/Mass Spectrometer, UK). Microanalyses were performed on a CE Elantech EA1110 elemental analyzer. Methods of specific experiments can be found in corresponding cited works.

### 27.2. Competition Binding Assays

Competition binding assays were performed in a total volume of 0.5 mL of a 50 mM Tris-acetate at 4° degree centigrade for 1 hour using [^3^H]flunitrazepam as the radioligand. For these binding assays, 20–50 mg of membrane protein harvested with hypotonic buffer (50 mM Tris-acetate pH 7.4 at 4 degree) was incubated with the radiolabel as previously described [139, 143]. Nonspecific binding was defined as radioactivity bound in the presence of 100 *μ*M diazepam and represented less than 20% of total binding. Membranes were harvested with a Brandel cell harvester followed by three ice-cold washes onto polyethyleneimine-pretreated (0.3%) Whatman GF/C filters. Filters were dried overnight and then soaked in Ecoscint A liquid scintillation cocktail (National Diagnostics; Atlanta, GA). Bound radioactivity was quantified by liquid scintillation counting. Membrane protein concentrations were determined using an assay kit from Bio-Rad (Hercules, CA) with bovine serum albumin as the standard.

### 27.3. Radioligand Binding Assays (Drs. McKernan and Atack) [12]

In brief, the affinity of compounds for human recombinant GABA(A) receptors was measured by competition binding using 0.5 nM [^3^H]flunitrazepam. Transfected HEK cells (beta2 gamma2 and desired alpha subtype) were harvested into phosphate-buffered saline, centrifuged at 3,000 g, and stored at −70°C until required. On the day of the assay, pellets were thawed and resuspended in sufficient volume of 50 mM Tris/acetate (pH 7.4 at 4°C) to give a total binding of approximately 1500–2000 dpm. Nonspecific binding was defined in the presence of 100 mM (final concentration) diazepam. Test compounds were dissolved in DMSO at a concentration of 10 mM and diluted in assay buffer to give an appropriate concentration range in the assay, such that the final DMSO concentration in the assay was always less than 1%. Total assay volume was 0.5 mL and assays were carried out in 96-well plates and incubation time started by the addition of 0.1 mL of resuspended cell membranes. Following incubation for 1 hour at 4°C, assays were terminated by filtration through GF/B filters, washed with 10 mL ice cold buffer, dried, and then counted using a liquid scintillation counter. The percentage of inhibition of [^3^H]flunitrazepam binding, the IC_50_, and the *K*_*i*_ values were calculated using the Activity Base Software Package (ID Business Solutions, Guildford, UK) according to the Cheng-Prusoff equation [143]. We have previously reported the synthesis of the following.

*1,3-Bis(8-acetyleno-5,6-dihydro-5-methyl-6-oxo-4H-imidazo[1,5a][1,4]benzodiazepine-3-carboxy) propyl diester* ***4*** (XLi-093) (Procedure A), experimental details previously reported [17].

*1,5-Bis(8-acetyleno-5,6-dihydro-5-methyl-6-oxo-4H-imidazo[1,5a][1,4]benzodiazepine-3-carboxy) pentyl diester* ***56*** (XLi-210), experimental details previously reported [17].

*1,3-Bis(8-ethyl-5,6-dihydro-5-methyl-6-oxo-4H-imidazo[1,5a][1,4]benzodiazepine-3-carboxy) propyl diester* ***10*** (Xli-356), experimental previously published [144].

*Bis(8-acetyleno-5,6-dihydro-5-methyl-6-oxo-4H-imidazo[1,5a][1,4]benzodiazepine-3-carboxy) dimethyl glycol diester* ***55*** (Xli-374), experimental details previously reported [17].

*8-Bromo-6-phenyl-4H-benzo[f]imidazo[1,5-a][1,4]diazepine-3-carboxylic acid* ***57***, experimental details previously reported [17].

*1,3-Bis(8-bromo-6-phenyl-4H-benzo[f]imidazo[1,5-a][1,4]diazepine-3-carboxy) propyl diester* ***59*** (DMH-D-070) (Procedure B), experimental details previously reported [17].

*1,3-Bis(8-trimethylsilylacetylenyl-6-phenyl-4H-benzo[f]imidazo[1,5-a][1,4]-diazepine-3-carboxy) propyl diester* ***61*** (DMH-D-048) (Procedure C), experimental details previously reported [17].

*1,3-Bis(8-acetylenyl-6-phenyl-4H-benzo[f]imidazo[1,5-a][1,4]diazepine-3-carboxy) propyl diester* ***63*** (DMH-D-053): experimental details previously reported [17].

*Bis(8-bromo-6-phenyl-4H-benzo[f]imidazo[1,5-a][1,4]diazepine-3-carboxy) diethylene glycol diester* ***58*** (DM-III-93), experimental details previously reported [17].

*Bis(8-trimethylsilylacetylenyl-6-phenyl-4H-benzo[f]imidazo[1,5-a][1,4]diazepine-3-carboxy) diethylene glycol diester* ***60*** (DM-III-94), experimental details previously reported [17].

*Bis(8-acetylenyl-6-phenyl-4H-benzo[f]imidazo[1,5-a][1,4]diazepine-3-carboxy) diethylene glycol diester* ***62*** (DM-III-96), experimental details previously reported [17].

## Supplementary Material

The supporting information contains details on the construction of the Unified Pharmacophore/Receptor Model. In addition, the crystallographic data (excluding structure factors) for the structures in this report have been deposited with the Cambridge Crysallographic Data Centre as supplementary publication numbers 687205 (DMH-D-053), 222395 (XLi-093), and 222396 (DM-II-96). Copies of the data can be obtained, free of charge, on application to CCDC, 12 Union Road, Cambridge CB2 1EZ, UK, (fax +44-(0)1223-336033 or email: deposit@ccdc.cam.ac.uk). Structures of all compounds found within Tables 5-9 are also contained within the supporting information under Appendix III.

## Figures and Tables

**Figure 16:**
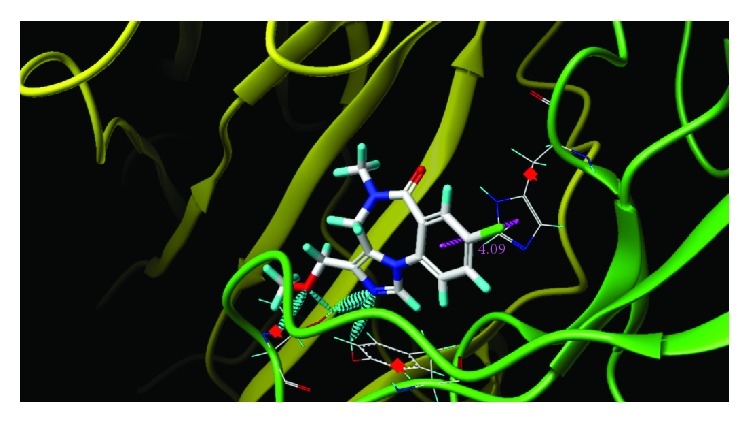
PWZ-029 docked with interactions. (1) HIS 105 *π*-stacking interaction with centroid of PWZ-029. (2) TYR 213 phenol OH hydrogen bonding to imidazole nitrogen lone pair. (3) THR 210 OH and lone pair on methoxy of PWZ029. (4) *α*5 ribbon being green. (5) *γ*2 ribbon being yellow. (6) Hydrogen bonding being aqua blue. (7) *π*-stacking being magenta.

**Figure 49:**
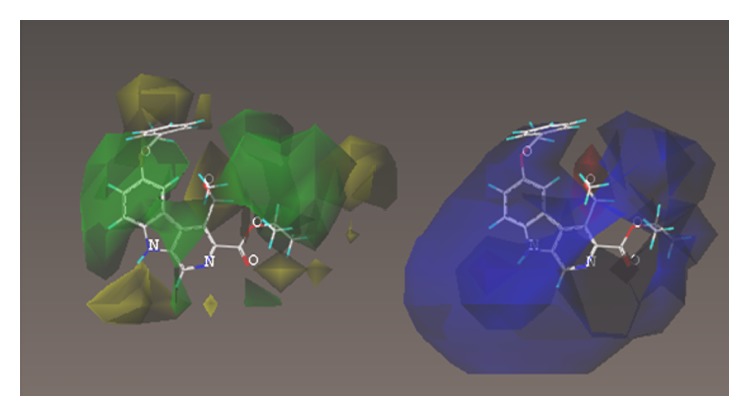
Steric (left) and electrostatic maps of the *α*1*β*3*γ*2 receptor subtype shown in the transparent mode as seen from the classic perspective.

**Figure 50 fig50:**
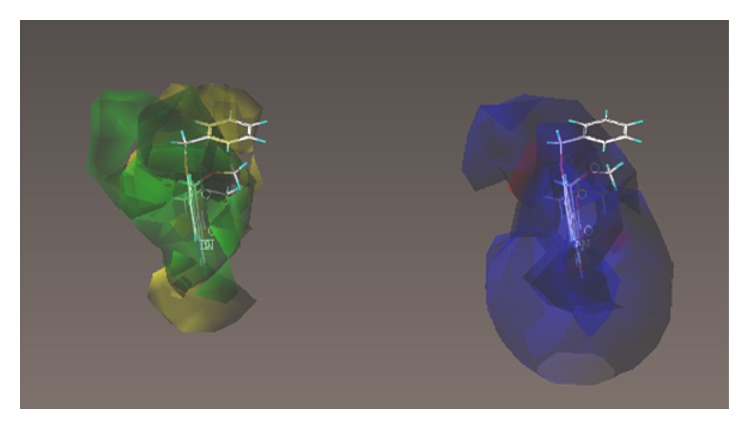
Steric (left) and electrostatic maps of the *α*1*β*3*γ*2 receptor subtype shown in the transparent mode as seen from the classic perspective (Figure 45) rotated 90°.

**Figure 51 fig51:**
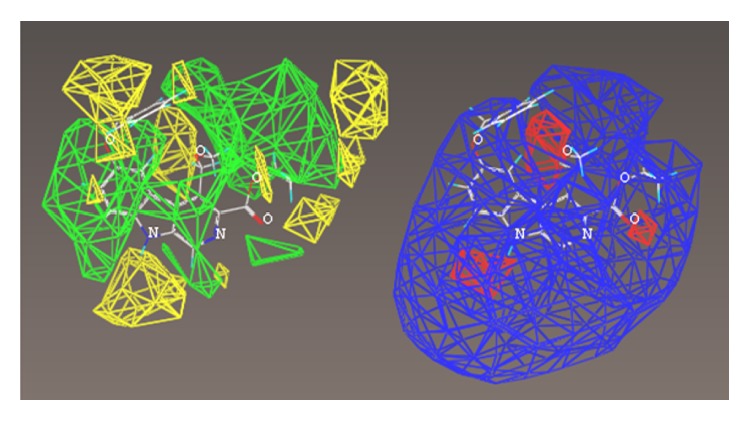
Steric (left) and electrostatic maps of the *α*1*β*3*γ*2 receptor subtype shown in line mode as seen from the classic perspective.

**Figure 52 fig52:**
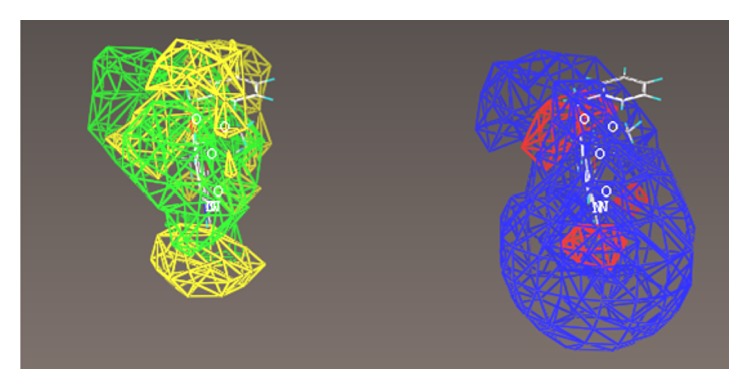
Steric (left) and electrostatic maps of the *α*1*β*3*γ*2 receptor subtype shown in line mode as seen from the classic perspective rotated 90°.

**Figure 53 fig53:**
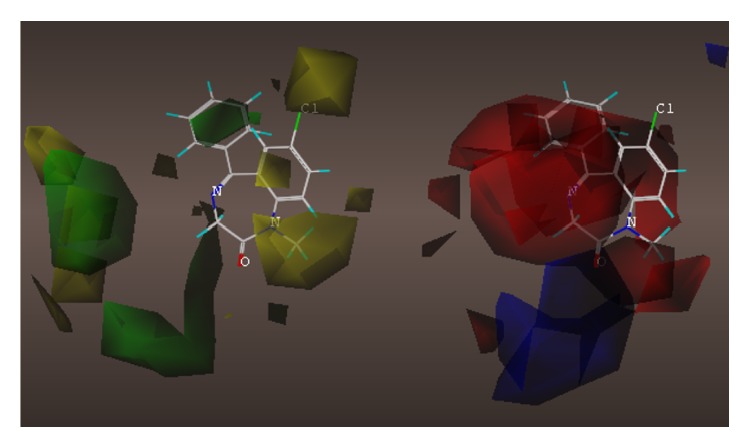
Steric (left) and electrostatic maps of the *α*2*β*3*γ*2 receptor subtype shown in the transparent mode as seen from the classic perspective.

**Figure 54 fig54:**
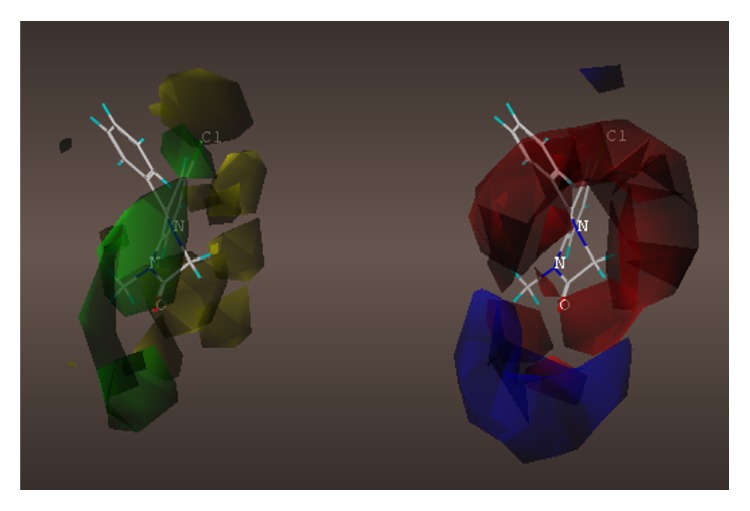
Steric (left) and electrostatic maps of the *α*2*β*3*γ*2 receptor subtype shown in the transparent mode as seen from the classic perspective rotated 90°.

**Figure 55 fig55:**
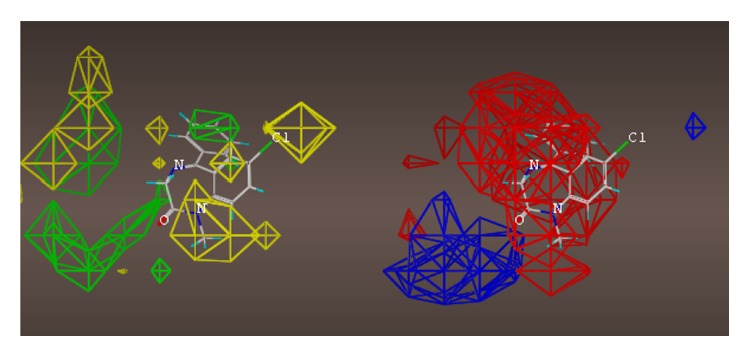
Steric (left) and electrostatic maps of the *α*2*β*3*γ*2 receptor subtype shown in line mode as seen from the classic perspective.

**Figure 56 fig56:**
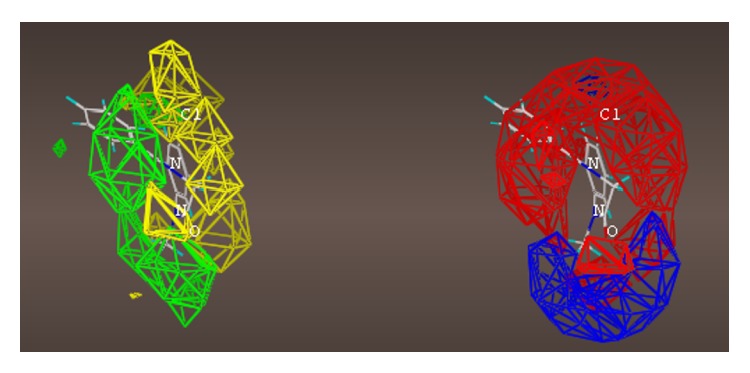
Steric (left) and electrostatic maps of the *α*2*β*3*γ*2 receptor subtype shown in line mode as seen from the classic perspective rotated 90°.

**Figure 57 fig57:**
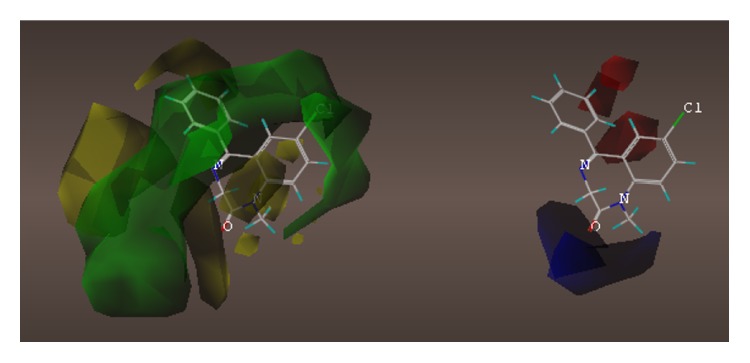
Steric (left) and electrostatic maps of the *α*3*β*3*γ*2 receptor subtype shown in the transparent mode as seen from the classic perspective.

**Figure 58 fig58:**
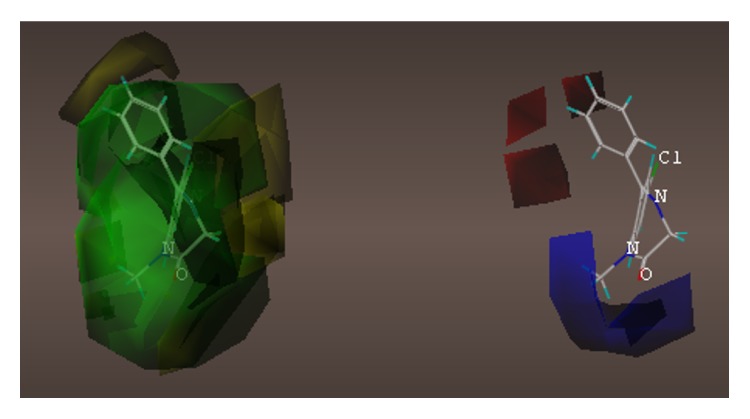
Steric (left) and electrostatic maps of the *α*3*β*3*γ*2 receptor subtype shown in the transparent mode as seen from the classic perspective rotated 90°.

**Figure 59 fig59:**
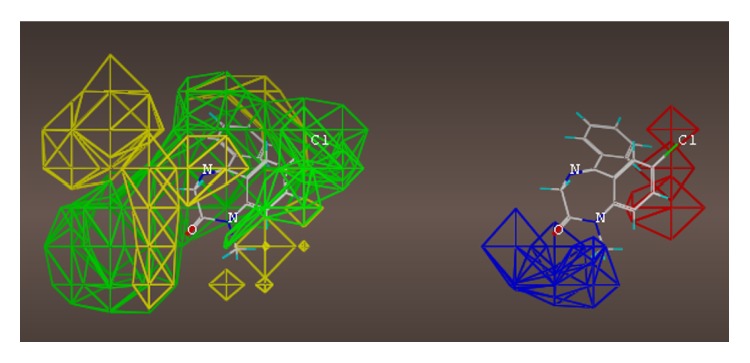
Steric (left) and electrostatic maps of the *α*3*β*3*γ*2 receptor subtype shown in line mode as seen from the classic perspective.

**Figure 60 fig60:**
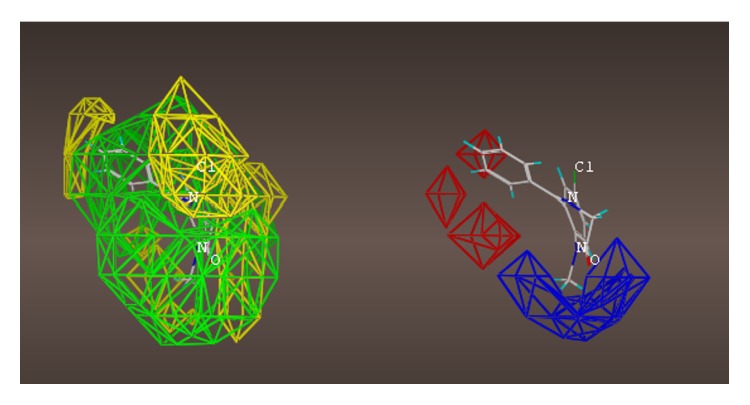
Steric (left) and electrostatic maps of the *α*3*β*3*γ*2 receptor subtype shown in line mode as seen from the classic perspective rotated 90°.

**Table 1:** Full PDSP panel receptor binding reported (Roth [138]) for XLi-093 and XLi-356.

Cook code	5ht1a	5ht1b	5ht1d	5ht1e	5ht2a	5ht2b	5ht2c	5ht3	5ht5a	5ht6	5ht7	*α*1A	*β*1B	*α*2A	*α*2B
XLi093	*∗*	Repeat	*∗*	*∗*	*∗*	*∗*	*∗*	*∗*	*∗*	*∗*	*∗*	*∗*	*∗*	*∗*	*∗*
XLi356	*∗*	*∗*	*∗*	*∗*	*∗*	*∗*	*∗*	*∗*	*∗*	*∗*	*∗*	*∗*	*∗*	*∗*	*∗*

Cook code	*α*2C	Beta1	Beta2	CB1	CB2	D1	D2	D3	D4	D5	DAT	DOR	H1	H2	H3

XLi093	*∗*	*∗*	*∗*	*∗*	*∗*	*∗*	*∗*	*∗*	*∗*	*∗*	*∗*	*∗*	*∗*	*∗*	*∗*
XLi356	*∗*	*∗*	*∗*	*∗*	*∗*	*∗*	*∗*	*∗*	*∗*	*∗*	*∗*	*∗*	*∗*	*∗*	*∗*

Cook code	H4	Imidaz oline	KOR	M1	M2	M3	M4	M5	MDR	MOR	NET	NMDA	SERT	*σ*1	*σ*2

XLi093	*∗*	*∗*	***2,024.00***	*∗*	*∗*	*∗*	*∗*	*∗*	*∗*	*∗*	*∗*	*∗*	*∗*	*∗*	*∗*
XLi356	*∗*	*∗*	**6,118.00**	*∗*	*∗*	*∗*	*∗*	*∗*	*∗*	*∗*	*∗*	*∗*	*∗*	*∗*	*∗*

Data (“secondary binding”) are *K*_*i*_ values. *K*_*i*_ values are reported in nanomolar concentration, Case Western Reserve University. “*∗*” indicates “primary missed” (<50% inhibition at 10 *µ*M). See full data of the PDSP screen in the report of Clayton [22].

**Table 2:** Affinity of PWZ-029 (**1**); *K*_*i*_ (nM)^a^.

Code	MW	*α*1	*α*2	*α*3	*α*4	*α*5	*α*6
PWZ-029 (**1**)	291.73	>300	>300	>300	ND	**38.8**	>300
PWZ-029 (**1**)	291.73	920	ND	ND	ND	**30**	ND
PWZ-029 (**1**)	291.73	362	180	328	ND	**6**	ND

^a^Data from three separate laboratories.

**Table 3:** Binding affinity at *αxβ*2*γ*2 GABA_A_ receptor subtypes (values are reported in nM).

Compound^a^	*α*1	*α*2	*α*3	*α*4	*α*5	*α*6
SH-053-R-CH_3_, (**15**)	2026	2377	1183	>5000	949.1	>5000
SH-053-S-CH_3_, (**16**)	1666	1263	1249	>5000	206.4	>5000
SH-053-2′F-R-CH_3_, (**2**)	759.1	948.2	768.8	>5000	95.17	>5000
SH-053-2′F-S-CH_3_, (**17**)	350	141	1237	>5000	19.2	>5000

^a^Data shown here are the means of two determinations which differed by less than 10%.

**Table 4:** Oocyte electrophysiological data of benzodiazepines^a^ [87].

Compound	*α*1	*α*2	*α*3	*α*5
SH-053-2′F-R-CH_3_ (**2**)	111/154	124/185	125/220	183/387
SH-053-2′F-S-CH_3_ (**17**)	116/164	170/348	138/301	218/389

^a^Efficacy at *αxβ*3*γ*2 GABA_A_ receptor subtypes as % of control current at 100 nM and 1 *μ*M concentrations. Data presented as percent over baseline (100) at concentrations of 100 nM/1 *μ*M.

**Table 5:** These ligands bound with potent affinity for *α*1; ligands bound with *K*_*i*_ values <20 nM at this subtype.

Cook code^a^	*α*1	*α*2	*α*3	*α*4	*α*5	*α*6
WY-TSC-4 (WYS8)	0.007	0.99	1.63		51.04	
SH-TSC-2 (BCCT)	0.03	0.0419	0.035		69.32	
QH-II-090 (CGS-8216)	0.05	0.08	0.12		0.25	17
XLI-286	0.051	0.064	0.118		0.684	
QH-II-077	0.06	0.08	0.05		0.12	4
QH-II-092	0.07	0.03	0.04	ND	0.17	ND
JYI-57	0.076	0.076	0.131	ND	0.036	ND
QH-II-085	0.08	0.06	0.02	ND	0.08	ND
XHE-II-024	0.09	0.18	0.32	14	0.24	11
PWZ-007A	0.11	0.1	0.09	ND	0.2	10
CGS8216	0.13	ND	ND	ND	ND	46
SPH-121	0.14	1.19	1.72	ND	4	479
QH-II-075	0.18	0.21	0.25	ND	1.3	40
PZII-028	0.2	ND	0.2	ND	0.32	1.9
CGS9895	0.21	ND	ND	ND	ND	9.3
PWZ-0071	0.23	0.17	0.12	ND	0.44	17.31
XHE-III-24	0.25	ND	8	222	10	328
JYI-42	0.257	0.146	0.278	ND	0.256	ND
CGS9896	0.28	ND	ND	ND	ND	181
JYI-64 (C17H12N4FBr)	0.305	1.111	0.62	ND	0.87	5000
PZII-029	0.34	ND	0.79	ND	0.52	10
BRETAZENIL	0.35	0.64	0.2	ND	0.5	12.7
FG8205	0.4	2.08	1.16	ND	1.54	227
YT-5	0.421	0.6034	36.06	ND	1.695	ND
6-PBC	0.49	1.21	2.2	ND	2.39	1343
QH-146	0.49	ND	0.76	ND	7.7	10000
DM-II-90 (C17H12N4BrCl)	0.505	1	0.63	ND	0.37	5000
SPH-165	0.63	2.79	4.85	ND	10.4	1150
BCCt	0.72	15	18.9	ND	110.8	5000
SH-I-048A	0.774	0.1723	0.383	ND	0.11	ND
alprazolam	0.8	0.59	1.43	ND	1.54	10000
Ro15-1788	0.8	0.9	1.05	ND	0.6	148
WYS10 C14H9F3N2O2	0.88	36	25.6	ND	548.7	15.3
WY-B-15	0.92	0.83	0.58	2080	4.42	646
WY-A-99-2 (WYS8)	0.972	111	102	2000	208	1980
XHE-III-06a	1	2	1	5	1.8	37
Xli366 C22H21N3O2	1	ND	ND	ND	ND	ND
JYI-59 (C22H13N3O2F4)	1.08	2.6	11.82	ND	11.5	5000
WYSC1 C16H16N2O2	1.094	5.44	12.3	ND	69.8	21.2
MLT-I-70	1.1	1.2	1.1	ND	40.3	1000
SVO-8-30	1.1	5.3	5.3	2.8	0.6	15
BCCE	1.2	4.9	5.7	ND	26.8	2700
XHE-III-04	1.2	2	1.1	219	0.4	500
XLi350 C17H11ClN2O	1.224	1.188	ND	ND	2.9	ND
XHE-III-49	1.3	5.5	4.2	38.7	11.3	85.1
PWZ-009A1	1.34	1.31	1.26	ND	0.84	2.03
DM-239	1.5	ND	0.53	ND	0.14	6.89
XLi351 C21H21ClN2OSi	1.507	0.967	ND	ND	1.985	ND
XLi352 C18H13ClN2O	1.56	0.991	ND	ND	1.957	ND
TG-4-39	1.6	34	24	5.6	1.4	23
TG-II-82	1.6	2.9	2.8	ND	1	1000
CM-A87	1.62	4.54	14.73	1000	4.61	1000
QH-II-082	1.7	1.8	1.6	ND	6.1	100
JYI-49 (C20H12N3O2F4Br)	1.87	2.38	ND	ND	6.7	3390
LJD-III-15E	1.93	14	19	ND	70.8	1000
SPH-38	2	5.4	10.8	ND	18.5	3000
XHE-I-093	2	7.1	8.9	1107	20	1162
MSA-IV-35	2.1	16	21	ND	995	3000
JYI-19 (C23H23N3O3S)	2.176	205	ND	ND	34	12.7
FLUNITRAZEPAM	2.2	2.5	4.5	ND	2.1	2000
YCT-5	2.2	11.46	16.3	ND	200	10000
TJH-IV-51	2.39	17.4	14.5	ND	316	10000
WYS13 C20H18N2O3	2.442	13	27.5	ND	163	5000
YT-III-25	2.531	5.786	5.691	ND	0.095	ND
XHE-III-14	2.6		10	13	2	7
WYS9 C16H15IN2O2	2.72	22.2	23.1	ND	562	122
JYI-47	2.759	2.282	0.511	ND	0.427	ND
CM-A82a	2.78	8.93	24.51	1000	7.49	1000
TG-4-29	2.8	3.9	2.7	2.1	0.18	3.9
XLi268 C17H13BrN4	2.8145	0.6862	ND	ND	0.6243	ND
JYI-54 (C24H15N3O3F4)	2.89	172	6.7	ND	57	1890
MMB-II-74	3	24.5	41.7	500	125.7	1000
MMB-III-016	3	1.97	2	1074	0.26	211
MMB-III-16	3	1.97	2	1074	0.26	211
QH-II-080b	3	3.7	4.7	ND	24	1000
YCT-7A	3	23.8	30.5	ND	240	10000
JYI-32 (C20H15N3O2BrF)	3.07	4.96	ND	ND	2.92	52.24
Ro15-4513	3.3	2.6	2.5	ND	0.26	3.8
XHE-II-017	3.3	10	7	258	17	294
XLi-JY-DMH ANX3	3.3	0.58	1.9	ND	4.4	5000
MLT-II-18	3.4	11.7	11	ND	225	10000
TJH-V-88	3.41		30	ND	140.9	10000
XLI-2TC	3.442	1.673	44.08	ND	1.121	
WYS15 C22H20N2O2	3.63	2.02	44.3	ND	76.5	5000
CM-A57	3.7	27	40	ND	254	1000
XHE-II-006b	3.7	15	12	1897	144	1000
JYI-60	3.73	1.635	4.3	ND	1.7	5000
RY-008	3.75	7.2	4.14	ND	1.11	44.3
MLT-II-18	3.9	12.2	24.4	ND	210	10000
OMB-18	3.9	1.2	3.4	1733	0.8	5
WY-B-09-1	3.99	8	32	1000	461	2000
SHU-1-19	4	12	7	48	14	84
ZK 93423	4.1	4.2	6	ND	4.5	1000
WY-B-23-2 (WYS11)	4.2	37.7	39	2000	176	69.4
WY-B-23-2 (WYS11)	4.2	37.7	73	ND	176	69.4
WY-B-99-1	4.4	4.5	5.58	2000	47	2000
WY-B-26-2	4.45	44.57	42.66	2000	124	2000
XHE-II-006a	4.7	4.4	20	1876	89	3531
CM-B01	4.8	31	34	1000	286	1000
PWZ-085	4.86	13	8.5	ND	0.55	40
MLT-II-16	5.05	10.41	18.4	ND	260	10000
3 PBC	5.3	52.3	68.8	ND	591	1000
MA-3-PROPOXYL	5.3	52.3	68.8	ND	591	1000
TJH-IV-43	5.42	30.19	48.9	ND	475	10000
DMCM	5.69	8.29	4	ND	1.04	134
DM-139	5.8	ND	169	ND	9.25	325
XHE-II-073A (R ENRICHED)	5.9	11	10	15	1.18	140
MSR-I-032	6.2	18.7	4	ND	3.3	74.9
JYI-70 (C19H13N4F)	6.3	2.1	ND	ND	0.56	5000
XLi343 C20H19ClN2OSi	6.375	17.71	ND	ND	150.5	ND
3 EBC	6.43	25.1	28.2	ND	826	1000
DM-146	6.44	ND	148	ND	4.23	247
DM-215	6.74	ND	7.42	ND	0.293	8.28
ZG69A	6.8	16.3	9.2	ND	0.85	54.6
ZG-69a (Ro15-1310)	6.8	16.3	9.2	ND	0.85	54.6
WY-B-14 (WYS7)	6.84	30	36	2000	108	1000
YT-II	6.932	0.8712	3.518	ND	5.119	ND
SVO-8-67	7	41	26	15	2.3	191
MLT-II-34	7.04	15.95	22.3	ND	158	1000
SPH-195	7.2	168.5	283.5	ND	271	10000
XHE-I-065	7.2	17	18	500	57	500
ZG-234	7.25	22.14	9.84	ND	0.3	5.25
SH-I-04	7.3	6.136	5.1	ND	7.664	ND
XHE-I-038	7.3	5	34	ND	132	1000
XHE-III-13	7.3	ND	7.1	880	1.6	311
WY-B-25	7.6	40	66	2000	263	2000
CM-A49 (R)	7.7	32.5	43	ND	69	1000
SVO-8-14	8	25	8	6.9	0.9	14
TG-4-29	8.3	10.2	6.9	ND	0.4	7.61
XHE-II-002	8.3	18	13	3.9	1.5	11
WY-B-14 (WYS7)	8.5	165	245	ND	1786	5000
XHE-II-011	9	60	39	3233	90	1000
WY-B-27-2	9.19	111	72	2000	449	2000
QH-II-063	9.4	9.3	31	ND	7.7	3000
JC184 C13H9BrN2OS	9.606	10.5	ND	ND	6.709	ND
ZG-208	9.7	11.2	10.9	ND	0.38	4.6
RY-I-31	10	45	19	ND	6	1000
WY-B-23-1	10	33	43	1000	189	2000
RY-098	10.1	22.2	16.5	ND	1.68	100
Hz148 C18H15N3	10.98	5000	ND	ND	256	5000
SVO-8-20	11	40	28	19	8.6	138
XHE-II-073B (S-ENRICHED)	11	17	12	33	2.1	269
SH-I-085	11.08	4.866	13.75	ND	0.24	ND
PWZ-096	11.1	36	16.9	ND	1.07	51.5
ZG-168	11.2	10.7	9.2	ND	0.47	9.4
CM-A77	11.51	51.9	105.16	1000	42.62	1000
WY-B-20	12	39	47	2000	122	3000
ABECARNIL	12.4	15.3	7.5	ND	6	1000
SH-I-89S	12.78	8.562	8.145	ND	3.23	ND
ZG-213	12.8	49.8	30.2	ND	3.5	22.5
EDC-I-071	12.9	83.1	ND	ND	314	5000
MMB-III-14	13	13	6.9	333	1.1	333
DM-173	13.1	ND	38.1	ND	0.78	118
XLI-348	13.56	11.17	1.578	ND	82.05	ND
EDC-I-093	13.6	423	ND	ND	2912	5000
diazepam	14	20	15	ND	11	ND
XLi223 C22H20BrN3O2	14	8.7	18	1000	10	2000
WYSC2 C15H11F3N2O2	14.14	113	170	ND	518	61.2
SH-I-030	14.42	11.04	19.09	ND	1.89	ND
CM-A100	14.49	44.91	123.8	1000	65.31	1000
RY-033	14.8	56	25.3	ND	1.72	22.9
HJ-I-037	15.07	8.127	28.29	ND	0.818	ND
YT-6	15.31	87.8	60.49	ND	1.039	ND
EDC-II-044	15.4	ND	293	ND	323	1000
CM-A58	16	120	184	ND	1000	1012
QH-II-067a	16	31	52	ND	199	3000
CD-214	16.4	48.2	42.5	ND	9.8	168
JYI-06 (C23H23N3O4)	16.5	5.48	5000	ND	12.6	5000
CM-A50 (S)	17	59	88	ND	144	1000
RY-061	17	13	6.7	ND	0.3	31
ZG-224	17.1	33.7	50	ND	2.5	30.7
ZG-63A	17.3	21.6	29.1	ND	0.65	4
DM-II-30 (C20H13N3O2BrF3)	17.6	13.4	28.51	ND	7.8	5000
CM-A64	18	60	116	ND	216	1000
RY-071	19	56	91	ND	7.2	266
WZ-113	19.2	13.2	13.4	ND	11.5	300
YT-III-23	19.83	23.65	19.87	ND	1.105	ND
CM-E09b	20	22	19	55	0.45	69
MMB-II-90	20	24	5.7	9	0.25	36

^a^Affinity of compounds at GABA_A_/BzR recombinant subtypes was measured by competition for [^3^H]flunitrazepam or [^3^H] Ro15-4513 binding to HEK cell membranes expressing human receptors of compositions *α*1*β*3*γ*2, *α*2*β*3*γ*2, *α*3*β*3*γ*2, *α*4*β*3*γ*2, *α*5*β*3*γ*2, and *α*6*β*3*γ*2 [139]. Data represent the average of at least three determinations with a SEM of ±5%. The structures of these ligands are in the Ph.D. thesis of Clayton (2011) [22] and Supporting Information.

**Table 6:** Ligands with potent affinity for *α*2; ligands bound with *K*_*i*_ values <20 nM at this subtype. The structures of these ligands are in the Ph.D. thesis of Clayton (2011) [22].

Cook code^a^	*α*1	*α*2	*α*3	*α*4	*α*5	*α*6
QH-II-092	0.07	0.03	0.04	ND	0.17	ND
SH-TSC-2 (BCCT)	0.03	0.0419	0.035	ND	69.32	ND
QH-II-085	0.08	0.06	0.02	ND	0.08	ND
XLI-286	0.051	0.064	0.118	ND	0.684	ND
JYI-57	0.076	0.076	0.131	ND	0.036	ND
QH-II-090 (CGS-8216)	0.05	0.08	0.12	ND	0.25	17
QH-II-077	0.06	0.08	0.05	ND	0.12	4
PWZ-007A	0.11	0.1	0.09	ND	0.2	10
JYI-42	0.257	0.146	0.278	ND	0.256	ND
PWZ-0071	0.23	0.17	0.12	ND	0.44	17.31
SH-I-048A	0.774	0.1723	0.383	ND	0.11	ND
XHE-II-024	0.09	0.18	0.32	14	0.24	11
QH-II-075	0.18	0.21	0.25	ND	1.3	40
XLi-JY-DMH ANX3	3.3	0.58	1.9	ND	4.4	5000
alprazolam	0.8	0.59	1.43	ND	1.54	10000
YT-5	0.421	0.6034	36.06	ND	1.695	ND
BRETAZENIL	0.35	0.64	0.2	ND	0.5	12.7
XLi268 C17H13BrN4	2.8145	0.6862	ND	ND	0.6243	ND
WY-B-15	0.92	0.83	0.58	2080	4.42	646
YT-II	6.932	0.8712	3.518	ND	5.119	
Ro15-1788	0.8	0.9	1.05	ND	0.6	148
XLi351 C21H21ClN2OSi	1.507	0.967	ND	ND	1.985	ND
WY-TSC-4 (WYS8)	0.007	0.99	1.63	ND	51.04	ND
XLi352 C18H13ClN2O	1.56	0.991	ND	ND	1.957	ND
DM-II-90 (C17H12N4BrCl)	0.505	1	0.63	ND	0.37	5000
JYI-64 (C17H12N4FBr)	0.305	1.111	0.62	ND	0.87	5000
XLi350 C17H11ClN2O	1.224	1.188	ND	ND	2.9	ND
SPH-121	0.14	1.19	1.72	ND	4	479
MLT-I-70	1.1	1.2	1.1	ND	40.3	1000
OMB-18	3.9	1.2	3.4	1733	0.8	5
6-PBC	0.49	1.21	2.2	ND	2.39	1343
YT-III-271	32.54	1.26	2.35	ND	103	ND
PWZ-009A1	1.34	1.31	1.26	ND	0.84	2.03
DM-II-72 (C15H10N20BrCl)	5000	1.37	ND	ND	2.02	5000
JYI-60 (C17H11N2OF)	3.73	1.635	4.3	ND	1.7	5000
XLI-2TC	3.442	1.673	44.08	ND	1.121	ND
QH-II-082	1.7	1.8	1.6	ND	6.1	100
TC-YT-II-76	101.1	1.897	5.816	ND	11.99	ND
MMB-III-016	3	1.97	2	1074	0.26	211
MMB-III-16	3	1.97	2	1074	0.26	211
XHE-III-06a	1	2	1	5	1.8	37
XHE-III-04	1.2	2	1.1	219	0.4	500
WYS15 C22H20N2O2	3.63	2.02	44.3	ND	76.5	5000
FG8205	0.4	2.08	1.16	ND	1.54	227
JYI-70 (C19H13N4F)	6.3	2.1	ND	ND	0.56	5000
JYI-47	2.759	2.282	0.511	ND	0.427	ND
JYI-49 (C20H12N3O2F4Br)	1.87	2.38	ND	ND	6.7	3390
FLUNITRAZEPAM	2.2	2.5	4.5	ND	2.1	2000
JYI-59 (C22H13N3O2F4)	1.08	2.6	11.82	ND	11.5	5000
Ro15-4513	3.3	2.6	2.5	ND	0.26	3.8
SPH-165	0.63	2.79	4.85	ND	10.4	1150
YT-II-76	95.34	2.797	0.056	ND	0.04	ND
TG-II-82	1.6	2.9	2.8	ND	1	1000
QH-II-080b	3	3.7	4.7	ND	24	1000
TG-4-29	2.8	3.9	2.7	2.1	0.18	3.9
PS-1-34B C20H17N4BrO	ND	4.198	3.928	ND	ND	ND
ZK 93423	4.1	4.2	6	ND	4.5	1000
XHE-II-006a	4.7	4.4	20	1876	89	3531
WY-B-99-1	4.4	4.5	5.58	2000	47	2000
CM-A87	1.62	4.54	14.73	1000	4.61	1000
OMB-19	22	4.6	20	3333	3.5	40
SH-I-085	11.08	4.866	13.75	ND	0.24	ND
BCCE	1.2	4.9	5.7	ND	26.8	2700
JYI-32 (C20H15N3O2BrF)	3.07	4.96	ND	ND	2.92	52.24
XHE-I-038	7.3	5	34	ND	132	1000
SVO-8-30	1.1	5.3	5.3	2.8	0.6	15
SPH-38	2	5.4	10.8	ND	18.5	3000
WYSC1 C16H16N2O2	1.094	5.44	12.3	ND	69.8	21.2
JYI-06 (C23H23N3O4)	16.5	5.48	5000	ND	12.6	5000
XHE-III-49	1.3	5.5	4.2	38.7	11.3	85.1
YT-III-25	2.531	5.786	5.691	ND	0.095	ND
SH-I-04	7.3	6.136	5.1	ND	7.664	ND
XHE-I-093	2	7.1	8.9	1107	20	1162
RY-008	3.75	7.2	4.14	ND	1.11	44.3
DMH-D-053 (C43H30N6O4)	236	7.4	272	5000	194.2	5000
WY-B-09-1	3.99	8	32	1000	461	2000
HJ-I-037	15.07	8.127	28.29	ND	0.818	ND
DMCM	5.69	8.29	4	ND	1.04	134
SH-I-89S	12.78	8.562	8.145	ND	3.23	ND
XLi223 C22H20BrN3O2	14	8.7	18	1000	10	2000
CM-A82a	2.78	8.93	24.51	1000	7.49	1000
QH-II-063	9.4	9.3	31	ND	7.7	3000
	9.4	9.3	31	ND	7.7	3000
XHE-II-017	3.3	10	7	258	17	294
TG-4-29	8.3	10.2	6.9	ND	0.4	7.61
MLT-II-16	5.05	10.41	18.4	ND	260	10000
JC184 C13H9BrN2OS	9.606	10.5	ND	ND	6.709	ND
ZG-168	11.2	10.7	9.2	ND	0.47	9.4
XHE-II-073A (R ENRICHED)	5.9	11	10	15	1.18	140
XLI-8TC	21.52	11.01	2.155	ND	4.059	ND
SH-I-030	14.42	11.04	19.09	ND	1.89	ND
XLI-348	13.56	11.17	1.578	ND	82.05	ND
ZG-208	9.7	11.2	10.9	ND	0.38	4.6
YT-TC-3	141.4	11.43	118.1	ND	29.22	ND
YCT-5	2.2	11.46	16.3	ND	200	10000
MLT-II-18	3.4	11.7	11	ND	225	10000
XHE-II-O53-ACID	50.35	11.8	44	ND	5.9	5000
SHU-1-19	4	12	7	48	14	84
RY-067	21	12	10	ND	0.37	42
DM-III-01 (C18H12N3O2Br)	5000	12	ND	ND	4.73	5000
MLT-II-18	3.9	12.2	24.4	ND	210	10000
SH-053-2′F	21.99	12.34	34.9	ND	0.671	ND
WYS13 C20H18N2O3	2.442	13	27.5	ND	163	5000
PWZ-085	4.86	13	8.5	ND	0.55	40
MMB-III-14	13	13	6.9	333	1.1	333
RY-061	17	13	6.7	ND	0.3	31
WZ-113	19.2	13.2	13.4	ND	11.5	300
YT-II-83	32.74	13.22	24.1	ND	3.548	ND
DM-II-30 (C20H13N3O2BrF3)	17.6	13.4	28.51	ND	7.8	5000
LJD-III-15E	1.93	14	19	ND	70.8	1000
YT-III-272	295.9	14.98	10.77	ND	103.3	ND
BCCt	0.72	15	18.9	ND	110.8	5000
XHE-II-006b	3.7	15	12	1897	144	1000
ABECARNIL	12.4	15.3	7.5	ND	6	1000
MLT-II-34	7.04	15.95	22.3	ND	158	1000
MSA-IV-35	2.1	16	21	ND	995	3000
JYI-04 (C21H23N3O3)	28.3	16	ND	ND	0.51	1.57
PS-1-35 C23H22N5OBr	ND	16.03	24.41	ND	ND	ND
ZG69A	6.8	16.3	9.2	ND	0.85	54.6
ZG-69a (Ro15-1310)	6.8	16.3	9.2	ND	0.85	54.6
YT-III-42	382.9	16.83	44.04	ND	9.77	ND
XHE-I-065	7.2	17	18	500	57	500
XHE-II-073B (S-ENRICHED)	11	17	12	33	2.1	269
TJH-IV-51	2.39	17.4	14.5	ND	316	10000
SH-I-047	1710	17.52	1222	ND	1519	ND
XLi343 C20H19ClN2OSi	6.375	17.71	ND	ND	150.5	ND
XHE-II-002	8.3	18	13	3.9	1.5	11
YT-III-38	1461	18.21	14.63	ND	3999	
JYI-72 (C22H21N4SiF)	48.5	18.5	ND	ND	11.5	5000
MSR-I-032	6.2	18.7	4	ND	3.3	74.9
JC208 C15H10N2OS	22.42	18.89	ND	ND	5.039	ND
diazepam	14	20	15	ND	11	ND

^a^Affinity of compounds at GABA_A_/BzR recombinant subtypes was measured by competition for [^3^H]flunitrazepam or [^3^H] Ro15-4513 binding to HEK cell membranes expressing human receptors of compositions *α*1*β*3*γ*2, *α*2*β*3*γ*2, *α*3*β*3*γ*2, *α*4*β*3*γ*2, *α*5*β*3*γ*2, and *α*6*β*3*γ*2 [22, 139]. Data represent the average of at least three determinations with a SEM of ±5%. ND: not determined.

**Table 7:** Ligands with potent affinity for *α*3; ligands bound with *K*_*i*_ values <20 nM at this subtype. The structures of these ligands are in the Ph.D. thesis of Clayton (2011) [22].

Cook code^a^	*α*1	*α*2	*α*3	*α*4	*α*5	*α*6
QH-II-085	0.08	0.06	0.02	ND	0.08	ND
SH-TSC-2 (BCCT)	0.03	0.0419	0.035	ND	69.32	ND
QH-II-092	0.07	0.03	0.04	ND	0.17	ND
QH-II-077	0.06	0.08	0.05	ND	0.12	4
YT-II-76	95.34	2.797	0.056	ND	0.04	ND
PWZ-007A	0.11	0.1	0.09	ND	0.2	10
XLI-286	0.051	0.064	0.118	ND	0.684	ND
QH-II-090 (CGS-8216)	0.05	0.08	0.12	ND	0.25	17
PWZ-0071	0.23	0.17	0.12	ND	0.44	17.31
JYI-57	0.076	0.076	0.131	ND	0.036	ND
BRETAZENIL	0.35	0.64	0.2	ND	0.5	12.7
PZII-028	0.2	ND	0.2	ND	0.32	1.9
QH-II-075	0.18	0.21	0.25	ND	1.3	40
JYI-42	0.257	0.146	0.278	ND	0.256	ND
XHE-II-024	0.09	0.18	0.32	14	0.24	11
SH-I-048A	0.774	0.1723	0.383	ND	0.11	ND
JYI-55	41.39	ND	0.504	ND	24.75	ND
JYI-47	2.759	2.282	0.511	ND	0.427	ND
DM-239	1.5	ND	0.53	ND	0.14	6.89
WY-B-15	0.92	0.83	0.58	2080	4.42	646
JYI-64 (C17H12N4FBr)	0.305	1.111	0.62	ND	0.87	5000
DM-II-90 (C17H12N4BrCl)	0.505	1	0.63	ND	0.37	5000
QH-146	0.49	ND	0.76	ND	7.7	10000
PZII-029	0.34	ND	0.79	ND	0.52	10
WYS19 C26H32N2O4Si	ND	ND	0.89	ND	ND	ND
XHE-III-06a	1	2	1	5	1.8	37
Ro15-1788	0.8	0.9	1.05	ND	0.6	148
MLT-I-70	1.1	1.2	1.1	ND	40.3	1000
XHE-III-04	1.2	2	1.1	219	0.4	500
FG8205	0.4	2.08	1.16	ND	1.54	227
PWZ-009A1	1.34	1.31	1.26	ND	0.84	2.03
alprazolam	0.8	0.59	1.43	ND	1.54	10000
XLI-348	13.56	11.17	1.578	ND	82.05	ND
QH-II-082	1.7	1.8	1.6	ND	6.1	100
WY-TSC-4 (WYS8)	0.007	0.99	1.63	ND	51.04	ND
SPH-121	0.14	1.19	1.72	ND	4	479
XLi-JY-DMH ANX3	3.3	0.58	1.9	ND	4.4	5000
MMB-III-016	3	1.97	2	1074	0.26	211
MMB-III-16	3	1.97	2	1074	0.26	211
XLI-8TC	21.52	11.01	2.155	ND	4.059	ND
6-PBC	0.49	1.21	2.2	ND	2.39	1343
YT-III-271	32.54	1.26	2.35	ND	103	ND
Ro15-4513	3.3	2.6	2.5	ND	0.26	3.8
TG-4-29	2.8	3.9	2.7	2.1	0.18	3.9
TG-II-82	1.6	2.9	2.8	ND	1	1000
OMB-18	3.9	1.2	3.4	1733	0.8	5
YT-II	6.932	0.8712	3.518	ND	5.119	ND
PS-1-34B C20H17N4BrO	ND	4.198	3.928	ND	ND	ND
DMCM	5.69	8.29	4	ND	1.04	134
MSR-I-032	6.2	18.7	4	ND	3.3	74.9
RY-008	3.75	7.2	4.14	ND	1.11	44.3
XHE-III-49	1.3	5.5	4.2	38.7	11.3	85.1
JYI-60 (C17H11N2OF)	3.73	1.635	4.3	ND	1.7	5000
FLUNITRAZEPAM	2.2	2.5	4.5	ND	2.1	2000
XLI-317	60.24	24.05	4.562	ND	0.295	ND
QH-II-080b	3	3.7	4.7	ND	24	1000
SPH-165	0.63	2.79	4.85	ND	10.4	1150
SH-I-04	7.3	6.136	5.1	ND	7.664	ND
SVO-8-30	1.1	5.3	5.3	2.8	0.6	15
WY-B-99-1	4.4	4.5	5.58	2000	47	2000
YT-III-25	2.531	5.786	5.691	ND	0.095	ND
BCCE	1.2	4.9	5.7	ND	26.8	2700
MMB-II-90	20	24	5.7	9	0.25	36
TC-YT-II-76	101.1	1.897	5.816	ND	11.99	ND
ZK 93423	4.1	4.2	6	ND	4.5	1000
RY-061	17	13	6.7	ND	0.3	31
JYI-54 (C24H15N3O3F4)	2.89	172	6.7	ND	57	1890
TG-4-29	8.3	10.2	6.9	ND	0.4	7.61
MMB-III-14	13	13	6.9	333	1.1	333
XHE-II-017	3.3	10	7	258	17	294
SHU-1-19	4	12	7	48	14	84
XHE-III-13	7.3	ND	7.1	880	1.6	311
DM-215	6.74	ND	7.42	ND	0.293	8.28
ABECARNIL	12.4	15.3	7.5	ND	6	1000
SVO-8-14	8	25	8	6.9	0.9	14
XHE-III-24	0.25	ND	8	222	10	328
SH-I-89S	12.78	8.562	8.145	ND	3.23	ND
PWZ-085	4.86	13	8.5	ND	0.55	40
XHE-I-093	2	7.1	8.9	1107	20	1162
ZG-168	11.2	10.7	9.2	ND	0.47	9.4
ZG69A	6.8	16.3	9.2	ND	0.85	54.6
ZG-69a (Ro15-1310)	6.8	16.3	9.2	ND	0.85	54.6
ZG-234	7.25	22.14	9.84	ND	0.3	5.25
XHE-II-073A (R ENRICHED)	5.9	11	10	15	1.18	140
RY-067	21	12	10	ND	0.37	42
XHE-III-14	2.6	ND	10	13	2	7
YT-III-272	295.9	14.98	10.77	ND	103.3	ND
SPH-38	2	5.4	10.8	ND	18.5	3000
ZG-208	9.7	11.2	10.9	ND	0.38	4.6
MLT-II-18	3.4	11.7	11	ND	225	10000
DM-II-33 (C20H13N3O2BrCl3)	88.6	85	11.6	ND	26.2	5000
JYI-59 (C22H13N3O2F4)	1.08	2.6	11.82	ND	11.5	5000
XHE-II-006b	3.7	15	12	1897	144	1000
XHE-II-073B (S-ENRICHED)	11	17	12	33	2.1	269
CM-B44 (ss)	32	43	12	379	4.3	485
WYSC1 C16H16N2O2	1.094	5.44	12.3	ND	69.8	21.2
JYI-48	75.59	90.68	12.78	ND	31.28	ND
XHE-II-002	8.3	18	13	3.9	1.5	11
RY-076	26	27	13	ND	0.7	22
WZ-113	19.2	13.2	13.4	ND	11.5	300
SH-I-085	11.08	4.866	13.75	ND	0.24	ND
CM-E10	23	26	14	215	0.51	96
TJH-IV-51	2.39	17.4	14.5	ND	316	10000
YT-III-38	1461	18.21	14.63	ND	3999	ND
CM-A87	1.62	4.54	14.73	1000	4.61	1000
diazepam	14	20	15	ND	11	ND
RY-053	49	29	15	ND	1	46
YCT-5	2.2	11.46	16.3	ND	200	10000
RY-098	10.1	22.2	16.5	ND	1.68	100
PWZ-096	11.1	36	16.9	ND	1.07	51.5
XLi223 C22H20BrN3O2	14	8.7	18	1000	10	2000
XHE-I-065	7.2	17	18	500	57	500
SH-I-02B	29.82	1315	18	ND	74.05	ND
MLT-II-16	5.05	10.41	18.4	ND	260	10000
RY-024 C19H19N3O3	26.9	26.3	18.7	ND	0.4	5.1
BCCt	0.72	15	18.9	ND	110.8	5000
LJD-III-15E	1.93	14	19	ND	70.8	1000
CM-E09b	20	22	19	55	0.45	69
RY-I-31	10	45	19	ND	6	1000
SH-I-030	14.42	11.04	19.09	ND	1.89	ND
YT-III-23	19.83	23.65	19.87	ND	1.105	ND
XHE-II-006a	4.7	4.4	20	1876	89	3531
OMB-19	22	4.6	20	3333	3.5	40
XHE-III-06b	32	33	20	299	28.6	740

^a^Affinity of compounds at GABA_A_/BzR recombinant subtypes was measured by competition for [^3^H]flunitrazepam or [^3^H] Ro15-4513 binding to HEK cell membranes expressing human receptors of compositions *α*1*β*3*γ*2, *α*2*β*3*γ*2, *α*3*β*3*γ*2, *α*4*β*3*γ*2, *α*5*β*3*γ*2, and *α*6*β*3*γ*2 [22, 139]. Data represent the average of at least three determinations with a SEM of ±5%. ND: not determined.

**Table 8:** Ligands with potent affinity for *α*4; ligands bound with *K*_*i*_ values <20 nM at this subtype. The structures of these ligands are in the Ph.D. thesis of Clayton (2011) [22].

Cook code^a^	*α*1	*α*2	*α*3	*α*4	*α*5	*α*6
CM-D45 C19H21N3O4	90.5	65.5	30.3	0.15	1.65	0.23
CM-D44	34.3	56.3	20.7	0.33	0.57	0.92
XHE-III-74	77	105	38	0.42	2.2	5.8
TG-4-29	2.8	3.9	2.7	2.1	0.18	3.9
SVO-8-30	1.1	5.3	5.3	2.8	0.6	15
XHE-II-002	8.3	18	13	3.9	1.5	11
XHE-III-06a	1	2	1	5	1.8	37
RY-080 C17H15N3O3	28.4	21.4	25.8	5.3	0.49	28.8
TG-4-39	1.6	34	24	5.6	1.4	23
SVO-8-14	8	25	8	6.9	0.9	14
RY-023 C22H27N3O3Si	197	142.6	255	7.8	2.61	58.6
MMB-II-90	20	24	5.7	9	0.25	36
XHE-III-14	2.6		10	13	2	7
XHE-II-024	0.09	0.18	0.32	14	0.24	11
XHE-II-073A (R ENRICHED)	5.9	11	10	15	1.18	140
SVO-8-67	7	41	26	15	2.3	191
CM-B31i (ss)	90	184	78	18	4.9	121
SVO-8-20	11	40	28	19	8.6	138

^a^Affinity of compounds at GABA_A_/BzR recombinant subtypes was measured by competition for [^3^H]flunitrazepam or [^3^H] Ro15-4513 binding to HEK cell membranes expressing human receptors of compositions *α*1*β*3*γ*2, *α*2*β*3*γ*2, *α*3*β*3*γ*2, *α*4*β*3*γ*2, *α*5*β*3*γ*2, and *α*6*β*3*γ*2 [22, 139]. Data represent the average of at least three determinations with a SEM of ±5%. ND: not determined.

**Table 9:** Ligands with potent affinity for *α*5; ligands bound with *K*_*i*_ values <20 nM at this subtype. The structures of these ligands are in the Ph.D. thesis of Clayton (2011) [22].

Cook code^a^	*α*1	*α*2	*α*3	*α*4	*α*5	*α*6
JYI-57	0.076	0.076	0.131	ND	0.036	ND
YT-II-76	95.34	2.797	0.056	ND	0.04	ND
QH-II-085	0.08	0.06	0.02	ND	0.08	ND
YT-III-25	2.531	5.786	5.691	ND	0.095	ND
SH-I-048A	0.774	0.1723	0.383	ND	0.11	ND
QH-II-077	0.06	0.08	0.05	ND	0.12	4
DM-239	1.5	ND	0.53	ND	0.14	6.89
QH-II-092	0.07	0.03	0.04	ND	0.17	ND
TG-4-29	2.8	3.9	2.7	2.1	0.18	3.9
SH-I-75	1487	989.9	773	ND	0.1825	ND
PWZ-007A	0.11	0.1	0.09	ND	0.2	10
XHE-II-024	0.09	0.18	0.32	14	0.24	11
SH-I-085	11.08	4.866	13.75	ND	0.24	ND
MMB-II-90	20	24	5.7	9	0.25	36
QH-II-090 (CGS-8216)	0.05	0.08	0.12	ND	0.25	17
JYI-42	0.257	0.146	0.278	ND	0.256	ND
MMB-III-016	3	1.97	2	1074	0.26	211
MMB-III-16	3	1.97	2	1074	0.26	211
Ro15-4513	3.3	2.6	2.5	ND	0.26	3.8
DM-215	6.74	ND	7.42	ND	0.293	8.28
XLI-317	60.24	24.05	4.562	ND	0.295	ND
RY-061	17	13	6.7	ND	0.3	31
ZG-234	7.25	22.14	9.84	ND	0.3	5.25
PZII-028	0.2	ND	0.2	ND	0.32	1.9
RY-067	21	12	10	ND	0.37	42
DM-II-90 (C17H12N4BrCl)	0.505	1	0.63	ND	0.37	5000
ZG-208	9.7	11.2	10.9	ND	0.38	4.6
XHE-III-04	1.2	2	1.1	219	0.4	500
TG-4-29	8.3	10.2	6.9	ND	0.4	7.61
RY-024 C19H19N3O3	26.9	26.3	18.7	ND	0.4	5.1
JYI-47	2.759	2.282	0.511	ND	0.427	ND
PWZ-0071	0.23	0.17	0.12	ND	0.44	17.31
CM-E09b	20	22	19	55	0.45	69
ZG-168	11.2	10.7	9.2	ND	0.47	9.4
RY-080 C17H15N3O3	28.4	21.4	25.8	5.3	0.49	28.8
BRETAZENIL	0.35	0.64	0.2	ND	0.5	12.7
CM-E10	23	26	14	215	0.51	96
JYI-04 (C21H23N3O3)	28.3	16	ND	ND	0.51	1.57
PZII-029	0.34	ND	0.79	ND	0.52	10
PWZ-085	4.86	13	8.5	ND	0.55	40
JYI-70 (C19H13N4F)	6.3	2.1	ND	ND	0.56	5000
CM-D44	34.3	56.3	20.7	0.33	0.57	0.92
SVO-8-30	1.1	5.3	5.3	2.8	0.6	15
Ro15-1788	0.8	0.9	1.05	ND	0.6	148
XLi268 C17H13BrN4	2.8145	0.6862	ND	ND	0.6243	ND
ZG-63A	17.3	21.6	29.1	ND	0.65	4
SH-053-2′F	21.99	12.34	34.9	ND	0.671	ND
XLI-286	0.051	0.064	0.118	ND	0.684	ND
SH-I-S66	22.93	30.36	55.26	ND	0.69	ND
RY-076	26	27	13	ND	0.7	22
DM-173	13.1	ND	38.1	ND	0.78	118
OMB-18	3.9	1.2	3.4	1733	0.8	5
HJ-I-037	15.07	8.127	28.29	ND	0.818	ND
PWZ-009A1	1.34	1.31	1.26	ND	0.84	2.03
ZG69A	6.8	16.3	9.2	ND	0.85	54.6
ZG-69a (Ro15-1310)	6.8	16.3	9.2	ND	0.85	54.6
JYI-64 (C17H12N4FBr)	0.305	1.111	0.62	ND	0.87	5000
SVO-8-14	8	25	8	6.9	0.9	14
JYI-03 (C21H21N3O3)	185.4	107	ND	ND	0.954	3.34
TG-II-82	1.6	2.9	2.8	ND	1	1000
RY-053	49	29	15	ND	1	46
YT-6	15.31	87.8	60.49	ND	1.039	ND
DMCM	5.69	8.29	4	ND	1.04	134
PWZ-096	11.1	36	16.9	ND	1.07	51.5
MMB-III-14	13	13	6.9	333	1.1	333
YT-III-23	19.83	23.65	19.87	ND	1.105	ND
RY-008	3.75	7.2	4.14	ND	1.11	44.3
XLI-2TC	3.442	1.673	44.08	ND	1.121	ND
XHE-II-073A (R ENRICHED)	5.9	11	10	15	1.18	140
QH-II-075	0.18	0.21	0.25	ND	1.3	40
RY-054	59	44	27	ND	1.3	126
TG-4-39	1.6	34	24	5.6	1.4	23
XHE-II-002	8.3	18	13	3.9	1.5	11
RY-031 (RY-10)	20.4	27	26.1	ND	1.5	176
FG8205	0.4	2.08	1.16	ND	1.54	227
alprazolam	0.8	0.59	1.43	ND	1.54	1000
XHE-III-13	7.3	ND	7.1	880	1.6	311
CM-D45 C19H21N3O4	90.5	65.5	30.3	0.15	1.65	0.23
RY-098	10.1	22.2	16.5	ND	1.68	100
YT-5	0.421	0.6034	36.06	ND	1.695	ND
JYI-60 (C17H11N2OF)	3.73	1.635	4.3	ND	1.7	5000
RY-033	14.8	56	25.3	ND	1.72	22.9
XHE-III-06a	1	2	1	5	1.8	37
SH-I-030	14.42	11.04	19.09	ND	1.89	ND
XLi352 C18H13ClN2O	1.56	0.991	ND	ND	1.957	ND
XLi351 C21H21ClN2OSi	1.507	0.967	ND	ND	1.985	ND
XHE-III-14	2.6	ND	10	13	2	7
DM-II-72 (C15H10N20BrCl)	5000	1.37	ND	ND	2.02	5000
XHE-II-073B (S-ENRICHED)	11	17	12	33	2.1	269
FLUNITRAZEPAM	2.2	2.5	4.5	ND	2.1	2000
XHE-III-74	77	105	38	0.42	2.2	5.8
SVO-8-67	7	41	26	15	2.3	191
6-PBC	0.49	1.21	2.2	ND	2.39	1343
RY-058	86	40	85	ND	2.4	150
ZG-224	17.1	33.7	50	ND	2.5	31.7
RY-066	83	60	48	ND	2.6	180
RY-023 C22H27N3O3Si	197	142.6	255	7.8	2.61	58.6
XLi350 C17H11ClN2O	1.224	1.188	ND	ND	2.9	ND
JYI-32 (C20H15N3O2BrF)	3.07	4.96	ND	ND	2.92	52.24
SH-I-89S	12.78	8.562	8.145	ND	3.23	ND
MSR-I-032	6.2	18.7	4	ND	3.3	74.9
OMB-19	22	4.6	20	3333	3.5	40
ZG-213	12.8	49.8	30.2	ND	3.5	22.5
YT-II-83	32.74	13.22	24.1	ND	3.548	ND
RY-059	89	70	91	ND	3.7	301
SPH-121	0.14	1.19	1.72	ND	4	479
RY-047	200	124	79	ND	4	340
XLI-8TC	21.52	11.01	2.155	ND	4.059	ND
YT-I-38	945.9	326.8	245.9	ND	4.07	ND
DM-146	6.44	ND	148	ND	4.23	247
CM-B44 (ss)	32	43	12	379	4.3	485
CM-B47	32	63	34	2007	4.4	717
XLi-JY-DMH ANX3	3.3	0.58	1.9	ND	4.4	5000
WY-B-15	0.92	0.83	0.58	2080	4.42	646
ZK 93423	4.1	4.2	6	ND	4.5	1000
JYI-12 (C19H16N3O3F3)	91	39	ND	ND	4.5	6.8
CM-A87	1.62	4.54	14.73	1000	4.61	1000
DM-III-01 (C18H12N3O2Br)	5000	12	ND	ND	4.73	5000
RY-057	73	85	97	ND	4.8	333
JYI-15 (C19H14N3O3F3)	205	812	ND	ND	4.8	22
CM-B31i (ss)	90	184	78	18	4.9	121
RY-079	121.1	141.9	198.4	159	5	113.7
JC208 C15H10N2OS	22.42	18.89	ND	ND	5.039	ND
YT-II	6.932	0.8712	3.518	ND	5.119	ND
XLi270 C19H14N4	36.39	25.81	ND	ND	5.291	ND
XHE-I-051	35	39	42	ND	5.3	979
MMB-II-87	200	333	107	109	5.4	333
XLI-210	231	661	2666	ND	5.4	54.22
XHE-II-O53-ACID	50.35	11.8	44	ND	5.9	5000
ABECARNIL	12.4	15.3	7.5	ND	6	1000
RY-I-31	10	45	19	ND	6	1000
QH-II-082	1.7	1.8	1.6	ND	6.1	100
SH-TSC-1 (PWZ-029)	362.4	180.3	328.2	ND	6.185	ND
XHE-II-065	1000	409	216	37	6.4	175
JYI-49 (C20H12N3O2F4Br)	1.87	2.38	ND	ND	6.7	3390
JC184 C13H9BrN2OS	9.606	10.5	ND	ND	6.709	ND
QH-II-066	76.3	42.1	47.4	2000	6.8	3000
XLI-381	619.9	285.6	3639	ND	7.051	ND
RY-071	19	56	91	ND	7.2	266
RY-I-28	283	318	102	ND	7.2	61
CM-A82a	2.78	8.93	24.51	1000	7.49	1000
YT-III-31	36.39	67.85	129.7	ND	7.59	ND
SH-I-04	7.3	6.136	5.1	ND	7.664	ND
QH-146	0.49	ND	0.76	ND	7.7	1000
QH-II-063	9.4	9.3	31	ND	7.7	3000
JC221 ANX1	106.175	49.405	182	ND	7.7495	362
DM-II-30 (C20H13N3O2BrF3)	17.6	13.4	28.51	ND	7.8	5000
SH-TS-CH_3_	107.2	50.09	20.95	ND	8.068	ND
RY-073	156	88	122	ND	8.5	267
SVO-8-20	11	40	28	19	8.6	138
SHU-221-1	66	41	43	3000	9	3000
YT-III-231	51.09	61.46	26.34	ND	9.124	ND
CM-E09a	176	192	122	490	9.2	718
DM-139	5.8	ND	169	ND	9.25	325
YT-III-42	382.9	16.83	44.04	ND	9.77	ND
CD-214	16.4	48.2	42.5	ND	9.8	168
XHE-III-24	0.25	ND	8	222	10	328
XLi223 C22H20BrN3O2	14	8.7	18	1000	10	2000
SPH-165	0.63	2.79	4.85	ND	10.4	1150
JYI-01 (C19H20N3O3Br)	59.2	159	96	ND	10.6	2.88
diazepam	14	20	15	ND	11	ND
XHE-III-49	1.3	5.5	4.2	38.7	11.3	85.1
WZ-113	19.2	13.2	13.4	ND	11.5	300
JYI-59 (C22H13N3O2F4)	1.08	2.6	11.82	ND	11.5	5000
JYI-72 (C22H21N4SiF)	48.5	18.5	ND	ND	11.5	5000
TC-YT-II-76	101.1	1.897	5.816	ND	11.99	ND
JYI-10 (C17H13N3O3F3Br)	5000	368	ND	ND	12.3	23
WZ-069	40	30.5	38.5	ND	12.6	1000
JYI-06 (C23H23N3O4)	16.5	5.48	5000	ND	12.6	5000
RY-072	220	150	184	ND	12.7	361
JYI-14 (C17H14N3O3F3)	32	25	ND	ND	13	565
XHE-II-053	287	45	96	1504	13.8	1000
Xli-347 C34H28N6O7	828.05	690.2	ND	ND	13.87	ND
SHU-1-19	4	12	7	48	14	84
CM-C28 (SR)	176	752	244	290	14	141
CM-E11	333	308	161	394	14	750
XHE-II-012	49	24	31	1042	14	2038
MMB-III-018	117	140	78	3500	14	976
MMB-III-18	117	140	78	3500	14	976
CM-B31c (ss)	118	319	173	37	15	137
CM-B45	230	557	336	265	15	230
XLI-093	1000	1000	858	1550	15	2000
DM-II-20 (C22H14N3O2F3)	54.3	27.14	35.68	ND	15.35	5000
XLi269 C22H22N4Si	221.8	154.2	ND	ND	15.51	ND
SH-O53-S-CH_3_-2′F	350	141	1237	ND	16	5000
JYI-13 (C21H16N3O4F3)	5000	63.7	ND	ND	16	8.38
CM-B34	472	451	223	114	17	175
XHE-II-017	3.3	10	7	258	17	294
JC222 C16H12N2OS	86.7	45.11	ND	ND	17.63	ND
SPH-38	2	5.4	10.8	ND	18.5	3000
WZ-070	72.7	30.7	53.2	ND	18.6	300
RY-069	692	622	506	ND	19	1000
SH-053-2′F-S-CH_3_	468.2	33.27	291.5	ND	19.2	ND
XHE-I-093	2	7.1	8.9	1107	20	1162

^a^Affinity of compounds at GABA_A_/BzR recombinant subtypes was measured by competition for [^3^H]flunitrazepam or [^3^H] Ro15-4513 binding to HEK cell membranes expressing human receptors of compositions *α*1*β*3*γ*2, *α*2*β*3*γ*2, *α*3*β*3*γ*2, *α*4*β*3*γ*2, *α*5*β*3*γ*2, and *α*6*β*3*γ*2 [22, 139]. Data represent the average of at least three determinations with a SEM of ±5%. ND: not determined.

**Table 10:** Ligands with potent affinity for *α*6; ligands bound with *K*_*i*_ values <20 nM at this subtype. The structures of these ligands are in the Ph.D. thesis of Clayton (2011) [22].

Cook code^a^	*α*1	*α*2	*α*3	*α*4	*α*5	*α*6
CM-D45 C19H21N3O4	90.5	65.5	30.3	0.15	1.65	0.23
CM-D44	34.3	56.3	20.7	0.33	0.57	0.92
JYI-04 (C21H23N3O3)	28.3	16	ND	ND	0.51	1.57
PZII-028	0.2	ND	0.2	ND	0.32	1.9
PWZ-009A1	1.34	1.31	1.26	ND	0.84	2.03
JYI-01 (C19H20N3O3Br)	59.2	159	96	ND	10.6	2.88
JYI-03 (C21H21N3O3)	185.4	107	ND	ND	0.954	3.34
Ro15-4513	3.3	2.6	2.5	ND	0.26	3.8
TG-4-29	2.8	3.9	2.7	2.1	0.18	3.9
JYI-11 (C22H22N3O3F3Si)	5000	5000	ND	ND	648	3.97
QH-II-077	0.06	0.08	0.05	ND	0.12	4
ZG-63A	17.3	21.6	29.1	ND	0.65	4
ZG-208	9.7	11.2	10.9	ND	0.38	4.6
OMB-18	3.9	1.2	3.4	1733	0.8	5
RY-024 C19H19N3O3	26.9	26.3	18.7	ND	0.4	5.1
ZG-234	7.25	22.14	9.84	ND	0.3	5.25
XHE-III-74	77	105	38	0.42	2.2	5.8
JYI-12 (C19H16N3O3F3)	91	39	ND	ND	4.5	6.8
DM-239	1.5	ND	0.53	ND	0.14	6.89
XHE-III-14	2.6	ND	10	13	2	7
TG-4-29	8.3	10.2	6.9	ND	0.4	7.61
DM-215	6.74	ND	7.42	ND	0.293	8.28
JYI-13 (C21H16N3O4F3)	5000	63.7	ND	ND	16	8.38
CGS9895	0.21	ND	ND	ND	ND	9.3
ZG-168	11.2	10.7	9.2	ND	0.47	9.4
PWZ-007A	0.11	0.1	0.09	ND	0.2	10
PZII-029	0.34	ND	0.79	ND	0.52	10
XHE-II-024	0.09	0.18	0.32	14	0.24	11
XHE-II-002	8.3	18	13	3.9	1.5	11
BRETAZENIL	0.35	0.64	0.2	ND	0.5	12.7
JYI-19 (C23H23N3O3S)	2.176	205	ND	ND	34	12.7
SVO-8-14	8	25	8	6.9	0.9	14
SVO-8-30	1.1	5.3	5.3	2.8	0.6	15
WYS10 C14H9F3N2O2	0.88	36	25.6	ND	548.7	15.3
QH-II-090 (CGS-8216)	0.05	0.08	0.12	ND	0.25	17
PWZ-0071	0.23	0.17	0.12		0.44	17.31

^a^The affinity of compounds at GABA_A_/BzR recombinant subtypes was measured by competition for [^3^H]flunitrazepam binding to HEK cell membranes expressing human receptors of compositions *α*1*β*3*γ*2, *α*2*β*3*γ*2, *α*3*β*3*γ*2, *α*4*β*3*γ*2, *α*5*β*3*γ*2, and *α*6*β*3*γ*2 [139]. Data represent the average of at least three determinations with a SEM of ±5%. ND: not determined.

## Abbreviations

APD: Antipsychotic drug
ASM: Airway smooth muscle
BS: Binding site
BZD, Bz: Benzodiazepine
BzR: Benzodiazepine receptor
DA: Dopamine
DAPI: 4′,6-Diamidino-2-phenylindole
GABA: Gamma amino butyric acid
GABA_A_: Gamma amino butyric acid A
GABA_A_R: Gamma amino butyric acid A receptor
HAL: Haloperidol
HEK: Human embryonic kidney
HPC: Hippocampal
LTK: Leukocyte tyrosine kinase
MAM: Methylazoxymethanol
NAM: Negative allosteric modulator
QSAR: Quantitative structure-activity relationship
PAM: Positive allosteric modulator
PV: Parvalbumin
SAL: Saline
SH-053: SH-053-2′F-R-CH_3_
SMA: Smooth muscle actin
TTX: Tetrodotoxin
VTA: Ventral tegmental area.
