# Supplementary material for: A Review of the Updated Pharmacophore for the Alpha 5 GABA(A) Benzodiazepine Receptor Model
Source: Int J Med Chem. 2015 Nov 10;2015:430248. doi: 10.1155/2015/430248 (PMC4657098; doi:10.1155/2015/430248)
Supplement: Supplementary file 1 — The supporting information contains details on the construction of the Unified Pharmacophore/Receptor Model. In addition, the crystallographic data (excluding structure factors) for the structures in this report have been deposited with the Cambridge Crysallographic Data Centre as supplementary publication numbers 687205 (DMH-D-053), 222395 (XLi-093), and 222396 (DM-II-96). Copies of the data can be obtained, free of charge, on application to CCDC, 12 Union Road, Cambridge CB2 1EZ, UK, (fax +44-(0)1223-336033 or email: deposit@ccdc.cam.ac.uk). Structures of all compounds found within Tables 5-9 are also contained within the supporting information under Appendix III. [file 430248.f1.pdf]

A Review of the Updated Pharmacophore for the Alpha 5 GABA(A) Benzodiazepine Receptor Model

Clayton, Terry[1†]; Poe, Michael M.[1†]; Rallapalli, Sundari[1]; Biawat, Poonam[1]; Savić, Miroslav M.[2]; Rowlett, James K.[3], Gallos, George[4]; Emala, Charles W.[4]; Kaczorowski, Catherine C.[5]; Stafford, Douglas C.[6]; Arnold, Leggy A.[1,6]; and Cook, James M.[1,6]*

[1]Department of Chemistry and Biochemistry, University of Wisconsin-Milwaukee, Milwaukee, Wisconsin, 53201; [2]Department of Pharmacology, Faculty of Pharmacy, University of Belgrade, Belgrade, Serbia; [3]Department of Psychiatry and Human Behavior, University of Mississippi Medical Center, Jackson, Mississippi, 39216; [4]Department of Anesthesiology, Columbia University, New York, New York, 10032; [5]Department of Anatomy and Neurobiology, University of Tennessee Health Science Center, Memphis, Tennessee, 38163; [6]Milwaukee Institute of Drug Discovery, University of Wisconsin-Milwaukee, Milwaukee, Wisconsin, 53201.

† These authors contributed equally to this work.

[*]Corresponding author, Department of Chemistry and Biochemistry, University of Wisconsin-Milwaukee, Milwaukee, Wisconsin, 53201, capncook@uwm.edu

Supporting Information

With References and Appendices.

Comparative Model of the Benzodiazepine Binding Site

A crystal structure of the heteropentameric GABA(A) receptors has not been reported so far, however the structure determination of the similar acetylcholine binding protein (AChBP)[1] has been of great interest for the GABA(A) receptor researchers. A homopentamer of the GABA channel with the β3 subunit has been solved by Miller et al. (*Nature* 2014). The AChBP protein shares less than 20% sequence homology with the extracellular domain of the GABA(A) receptor,[2] however the overall structural similarity is estimated to be around 60 – 75 %.[2] Comparative modeling studies have been carried out that used the structure of AChBP to develop models of the extracellular domain of GABA(A)R.[1, 3, 4] In addition, electron cryomicroscopy (cryo-EM) structures that represent the extracellular and transmembrane portion of the nACh receptor were used the modeling of GABA(A) receptors.[2] However, the homology of the sequence is not very high, making the prediction of detailed features highly uncertain. Therefore, docking studies with Bz BS ligands[5] have a more qualitative character and do not explain all differential interactions of GABA(A)R ligands with the existing subdomains. Different experimental structures of the nAChR[1, 2, 6] demonstrated the structure variation of between members of the same family, [1, 2, 6] which can help us to extrapolate to which extent GABA(A) receptor subunits differ from each other.

The majority of Bz/GABA(A)R have 1γ, 2α and 2β subunits and each domain has a "plus" and a "minus" side (Figure 1). As a consequence, each subdomain interfaces with the plus and minus sides of neighboring subdomains. The Bz binding site can be found between the $\alpha^{+}\gamma^{-}$ subunits and is significantly bigger but similar to the two GABA binding pockets, which are positioned between the $\beta^{+}\alpha^{-}$ subunits.[6-8] Overall, the coordination of the α1β2γ2 GABA(A) receptor can be described as γβαβα started counter clockwise from plus to minus (Figure 1).[4, 5, 9, 10]

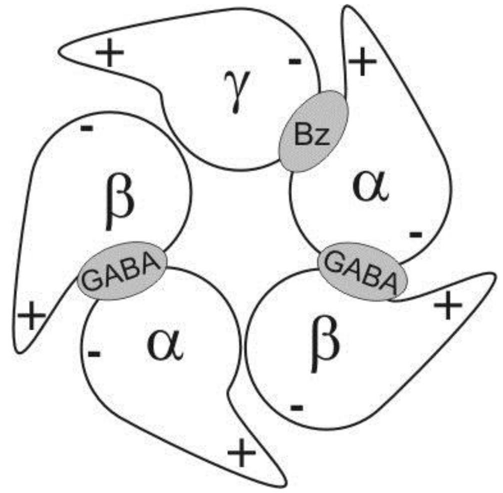

Figure 1. Absolute subunit arrangement of the α1β2γ2 GABA(A) receptor when viewed from the synaptic cleft.[25]

The GABA(A) binding sites are located at the $\beta^+\alpha^-$ subunit interfaces and the modulatory benzodiazepine binding site is located at the $\alpha^+\gamma$ subunit interface.

The γ2 subdomain is important for interacting the benzodiazepines in addition to many other molecules that act via the Bz BS.[11, 12] Sequence variations that differ between α and γ subdomains are responsible for the subtype selectivity and efficacy of Bz BS ligands.[11-14] Three segments have been proposed to generate the Bz BS: 1) The α^+ side, 2) the loops A, B and C, and 3) three segments of the γ^- called loops D, E and F.[11, 12] X-ray crystallography and EM-structures AChBP[1] and the nAChR[15] confirmed these segments, which create a groove-like pocket between subdomains and that are conserved among the entire superfamily. Trudell and Hagen proposed a near planar cleft BzR binding sites in 1980.[16, 17]

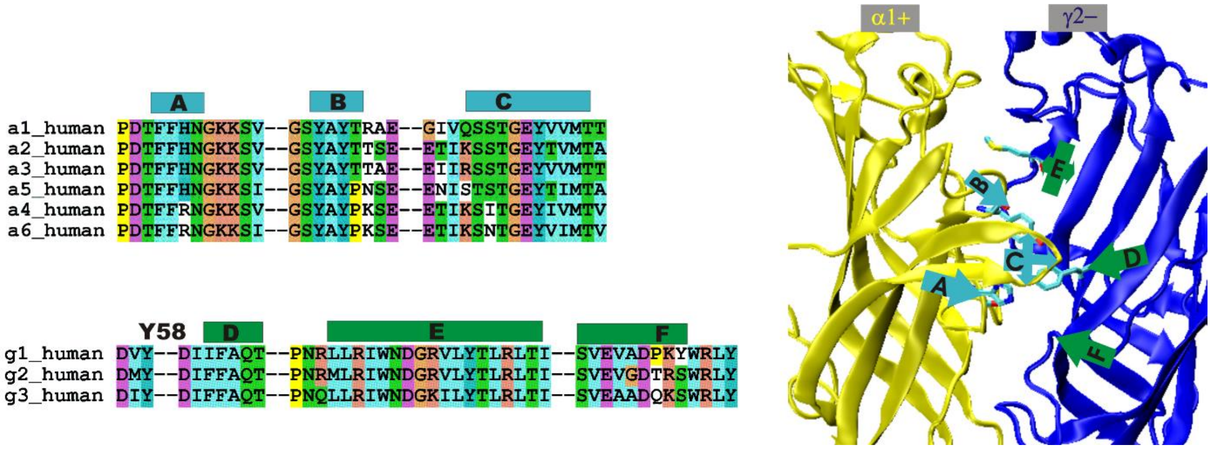

Figure 2. Alignment and homology model depiction of the so called "loops A-F" and flanking regions of the human sequences of the Bz recognition site in different subunits.[1,11-15,25]

View of the homology model (Figure 2) with the channel in between units. The membrane can be pictured parallel to the top edge of the structure. Stick representation is used for key residues: Loop A His102, loop B Tyr 160 and loop D Phe77.

After the nAChR structure was available, newer reports of liganded AChBP crystal structures were published.[18] Importantly, these structures showed that the local conformation of the BS is influenced by the ligand. This is particularly the case for the loop C. Thus, this highly mobile structure element responds towards ligand binding and induces smaller changes along its boundary.[18] This phenomena has been observed for other receptors as well and it has been hypothesized that low energy conformers can be separately stabilized by different ligands. Unfortunately, *a priori* prediction of the appropriate conformer/ligand pair is not conclusive at this time. The models can predict changes in binding sites as large as 40% and distances of key residues can vary several Ångstroms.

Small changes of the protein conformation can severely change the efficacy of Bz BS ligands.[19] In addition, GABA(A) receptor ligands have different efficacies towards particular GABA(A) receptor subtypes.[20, 21] The stabilization of the active state can be as small as 1 kcal/mol underlining the fact that proteins are inherently dynamic and can adopt many

conformations.[22] Therefore, the prediction of the absolute assignments of specific side chains to specific descriptors for any particular conformation is not possible at present. However, a "suggested" orientation of the pharmacophore in the receptor can be predicted implying conformational flexibility of residues that satisfy pharmacophoric descriptors. The combination of a pharmacophore model and a homology results in a model that assigns large lipophilic areas to specific regions and enables flexible assignment of for instance H-bond bridges or π-π stacking. Water molecules can significantly complicate prediction of binding. Therefore, our group favors the pharmacophore/receptor models for drug design based on the rigid planar ligands (K_i = 5nM–20nM) that have introduced by Trudell et al.[17]

Relative Orientation of the Pharmacophore within the Comparative Model

The Milwaukee group published a review prior to structure determination of the AChBP that included results of site-directed mutagenesis and gave valuable insights in respect to important side chains that formed the Bz BS and represent pharmacophoric descriptors.[23] The combination of that model and the recent homology models to lymnea AChBP,[2] aplysia AChBP and the nAChR[24] resulted in a novel model that is discussed here. Experimental evidence induces some degree of conformational flexibility of the model that hint towards variable assignments for H-bridge interactions. In addition, specific areas of lipophilic interactions create a binding site geometry flanked by particular amino acid residues (Figure 3).

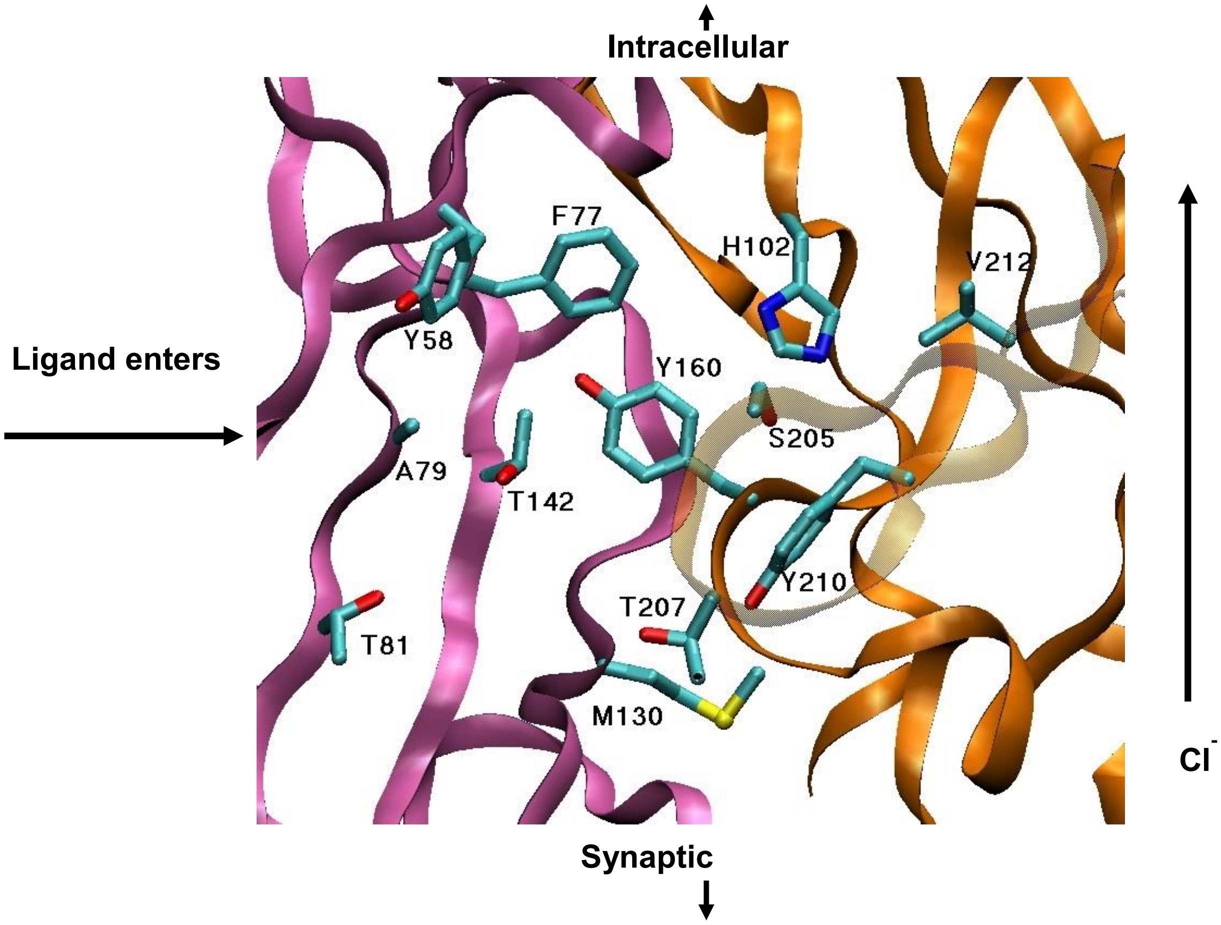

Figure3. Ribbon illustration of the GABA(A) BzR binding pocket conceived by Clayton et al.[25]

Combining Homology Model and Experimental Evidence

Docking of diazepam and flumazenil was performed to test our computational ligand docking to homology models as described by the two assignments above. Different models based on different templates were used[1, 15, 18] and the top 100 ligand poses per model were retained. A query among these poses was conducted based on the following parameters: The ring conformation of diazepam or flumazenil (Figure 4) should be in the active state;[26] residues α1H102/α1V203 and α1Y210/α1V212 are new the substituent in the seven position (consistent with the covalent labeling data); the 3′-ester group of flumazenil is near residues γ2A79 and γ2T81. The resulting docking poses were consistent with all covalent labeling studies and the steric requirements of 3′ substituted imidazobenzodiazepines[27] binding.

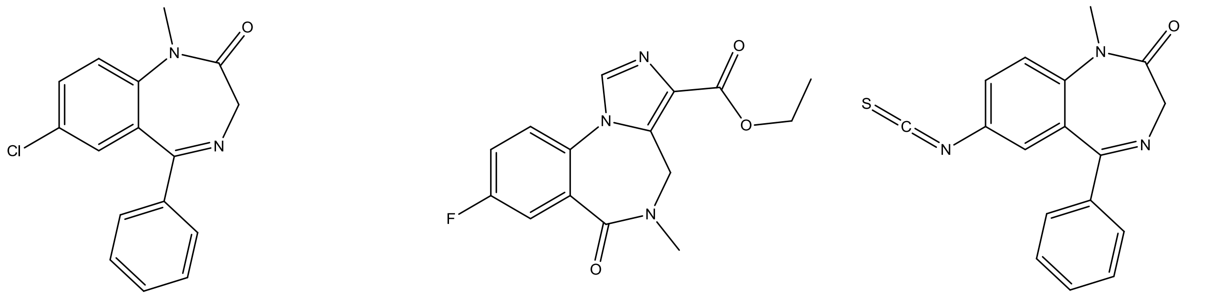

Diazepam Flumazenil 7-isothiocyano-1,4-benzodiazepine

Figure 4. Structures of BZDs.

All poses were found in the database. Representatively, diazepam is shown in Figure 5.

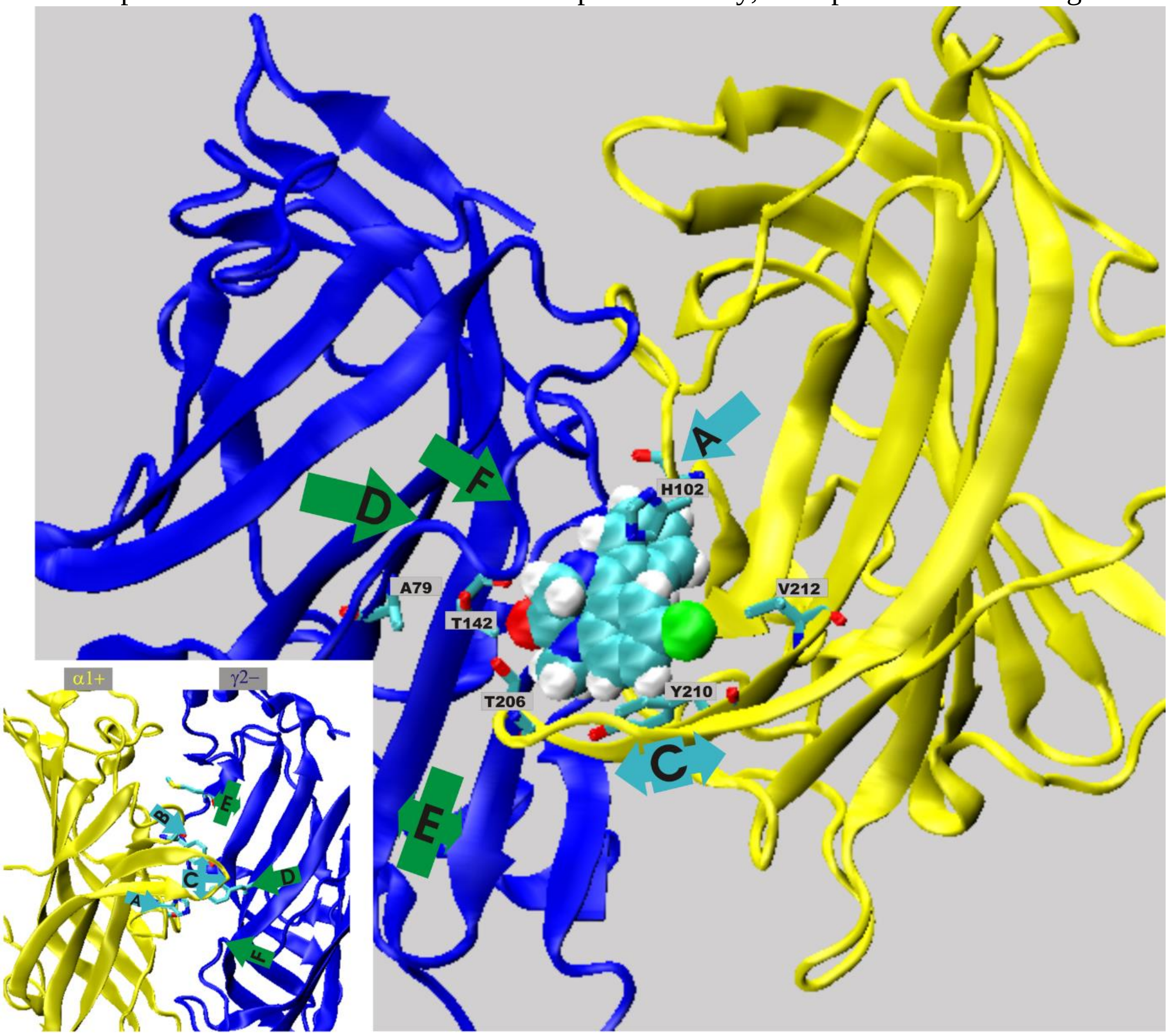

Figure 5. Diazepam docking pose in α1γ2. The beta subunit is not in the binding pocket.[25]

In Figure 5, diazepam was shown as space filling structure (turquoise: carbon, white: hydrogen, green: halogen, red: oxygen, and blue: nitrogen). The receptor was presented at ribbon with key amino acids in stick representation. The insert figure represents the empty pocket in the

"upright" position. This orientation is different from the main figure, where the structure has been turned and tilted to bring diazepam into the orientation of the unified pharmacophore model. In this orientation, His102 and Thr207 satisfy H_2 and H_1, respectively, and His 102, Val 212 and Tyr 210 would represent L_2 a hydrophobic pocket. This orientation of the 7-substituted reactive compounds such as isothiocyanate (see Figure 4, third structure) is in position to react with His102, Tyr 210, Val 212, and Val 203. This orientation was developed in collaboration with Professor Werner Seighart.[25]

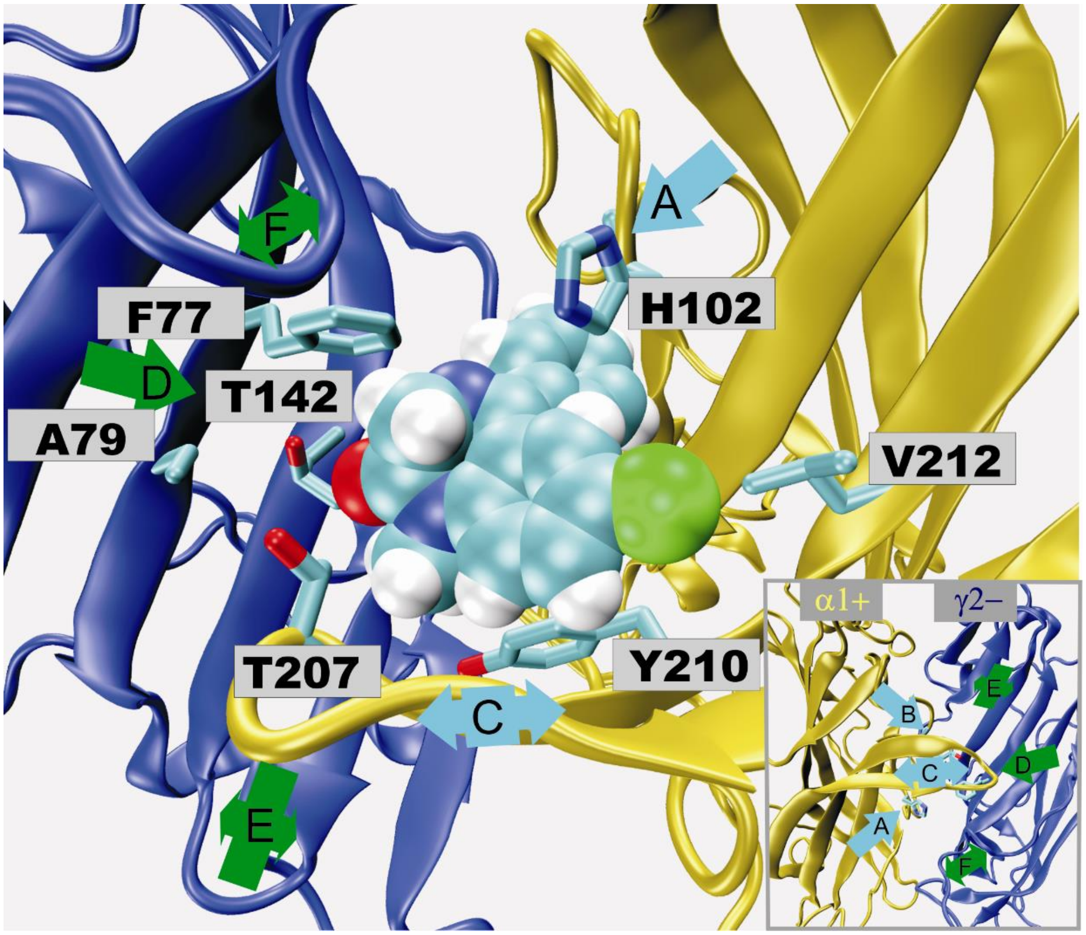

Figure 6. High resolution image of diazepam docking pose in α1γ2 binding pocket.[25]

The docking pose in Figure 6 and similar predictions satisfy the two definitions for L_2 and A_2 that are discussed above and the following descriptors: H_1 is aligned with the side chains near the top of loop C, the L_{DI} region is close the subdomain interaction (the α1 loop B (Y160) and the γ2 region are forming the sheet involving M130, T142 and F77); A_2 is represented by H-bond interaction near α1Y160, γ2A79, γ2T81, γ2M130 and γ2T142. If these proximity relations that are found in the docking poses are compared with the unified pharmacophore model we can predict the following orientation the residues.

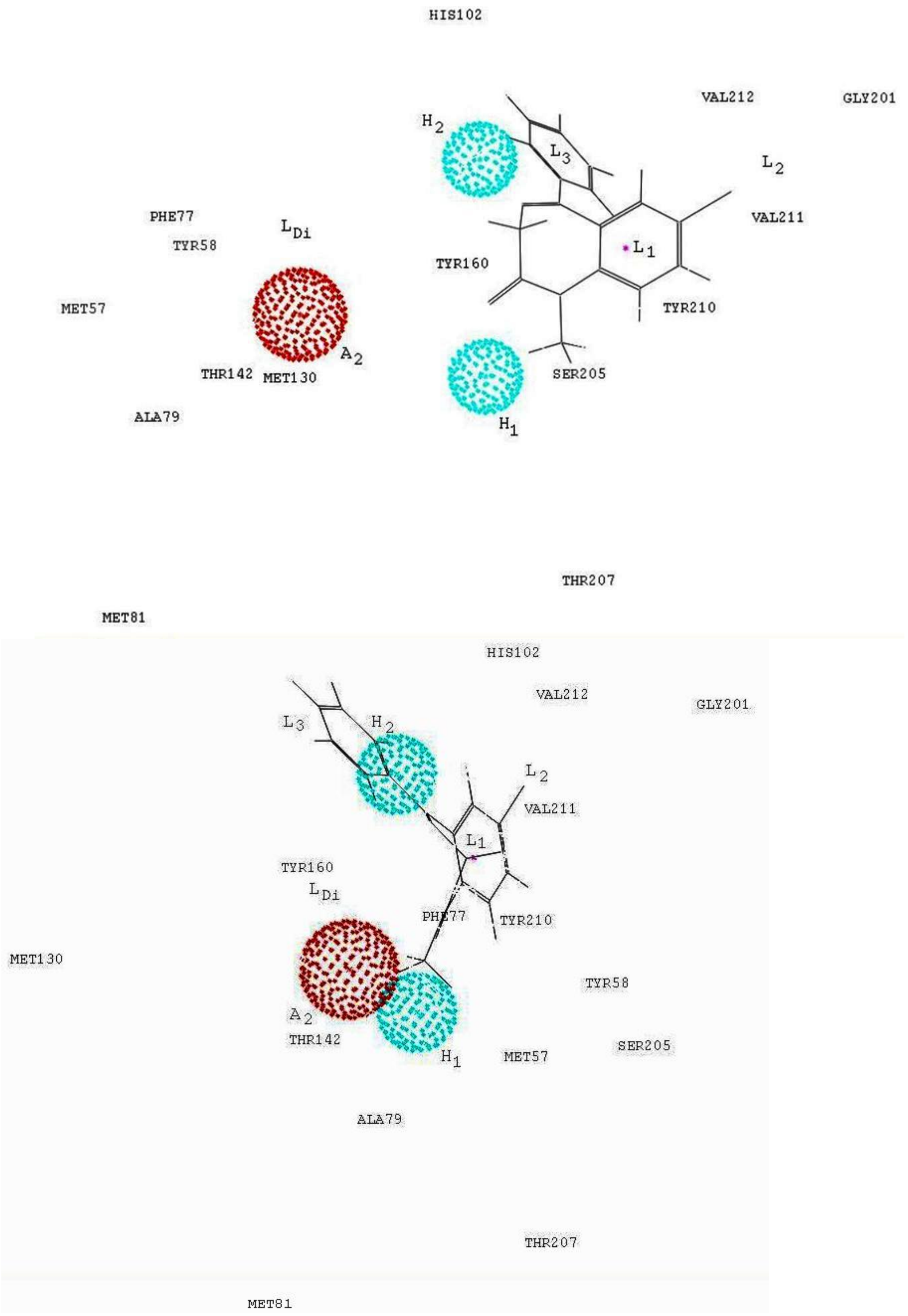

Figure 7. Orthogonal views of the location of residues with respect to pharmacophore in the α1β3γ2 BzR binding pocket.[25]

The Figures 7 and 8 show the orientation of Bz BS residues relative to the pharmacophore model in two views that correspond to a 90° rotation representing the evaluation

of experimental data and the comparative model of the $\alpha1\beta2\gamma2$ GABA(A). The ligand is diazepam. It is possible that more than one side chain could satisfy the same descriptor because inverse agonists stabilize protein conformations that vary from the conformations preferred by agonists,

In addition, there is still some degree of variation in structural details of the poses that represent this orientation. The factors of these structure variations: template and alignment choices, construction of the model and its refinement, and docking algorithms.

With the alignment of XLi-093 and docked Ro15-1788,[25] it is becomes clear that these compounds are able to orientate towards the extracellular domain instead of solvent accessible space through the subunit boundary. Orientations of the pharmacophore model inside the structural model of the GABA(A) receptor have been proposed in the past.[28-30]

Current refinements of the predicted protein-ligand complexes with the support of the unified pharmacophore model is a promising approach to create 3D structures that are more accurate and applicable for structure-guided drug design. New approaches that combine protein modeling and pharmacophore elucidation are currently investigated.

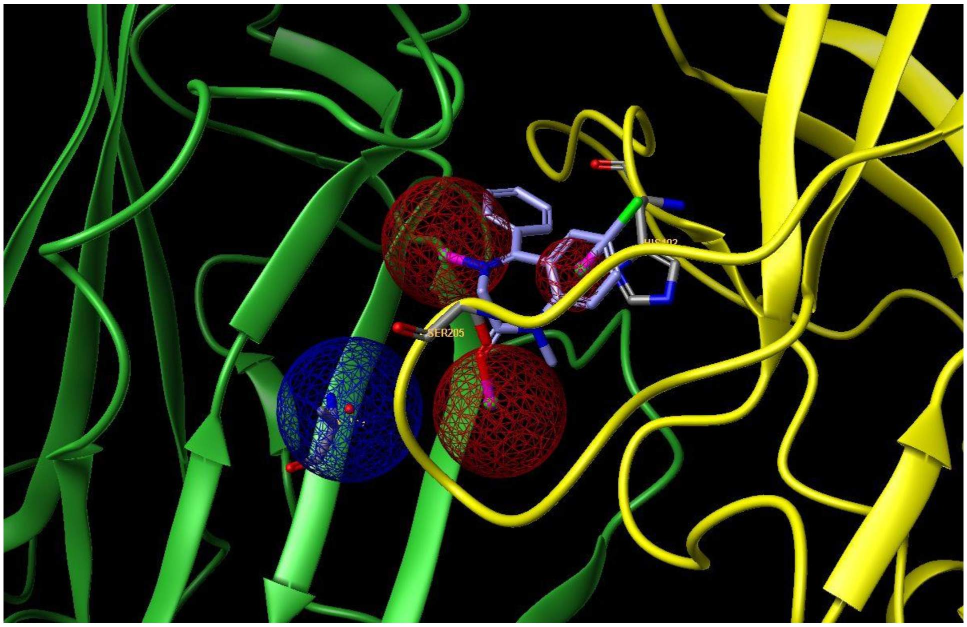

Figure 8. Unified model of the $\alpha1\gamma2$ subtype benzodiazepine receptor with diazepam docked in the binding site.

Construction of the Unified Pharmacophore/Receptor Model

The 150+ BZ ligands[31, 32] that belong to fifteen different scaffolds were used to create a unified pharmacophore/receptor model. The relative affinities, efficacies and functional effects from ligands of the same structural class at the diazepam-sensitive and diazepam-insensitive benzodiazepine receptor binding sites applied. In addition, we took the approximate locations of descriptors (hydrogen bond donor sites, hydrogen bond acceptor sites, lipophilic regions, and regions of steric repulsion) that were based primarily on in vitro binding affinities in account. The compounds from different scaffolds were superimposed in order to satisfy the same descriptors, creating the unified pharmacophore model this included the rigid planar templates (Ki = 5nM-20nM) of Trudell. [17]

The model has two hydrogen bond donating groups (H_1 and H_2), one hydrogen bond accepting group (A_2) and one lipophilic site (L_1). There are also lipophilic regions of interaction (L_2, L_3, L_4, and L_{Di}) and regions of negative steric repulsion (S_1, S_2 and S_3). Important for positive allosteric modulation are occupation of L_2 and/or L_3, and L_4 as well as interactions at H_1, H_2, and L_1. Inverse agonists only require interactions with the H_1, L_1, and A_2 descriptors for good activity *in vivo*.[33-37] The L_3 region is lipophilic of nature differentiating diazepam sensitive (DS) and the diazepam insensitive (DI) coordination. Figure 8 depicts the relative locations of the different descriptors and regions and classes of ligands for GABA(A) R are shown in Table 1.

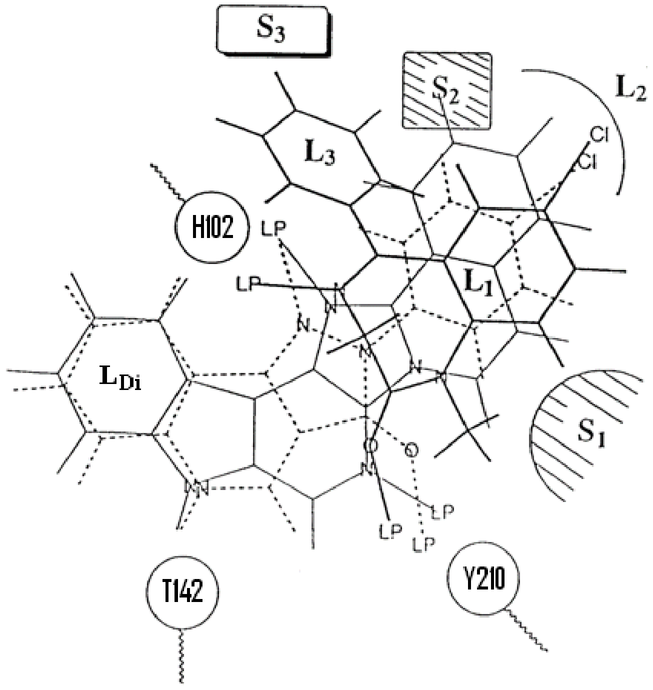

Figure 8. The Milwaukee-based unified pharmacophore.[25]

Table 1. Structures of ligands and their modulation at the α1 subtype.[25]

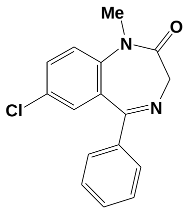	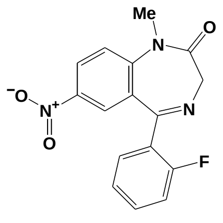	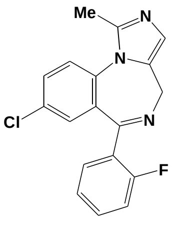	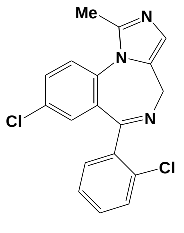
diazepam<br>agonist	flunitrazepam<br>agonist	midazolam<br>agonist	triazolam<br>agonist

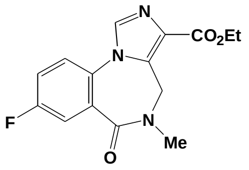

Ro15-1788

antagonist

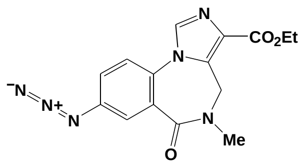

Ro15-4513

inverse agonist

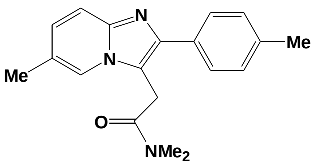

zolpidem

agonist

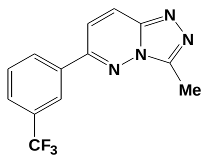

CL-218,872

partial agonist

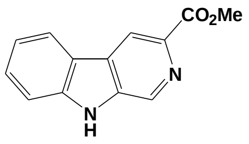

β-CCM

inverse agonist

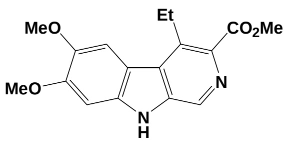

DMCM

inverse agonist

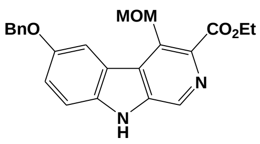

ZK-93423

agonist

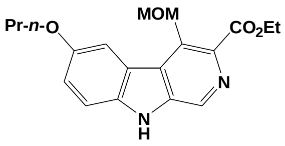

6-PBC

partial agonist

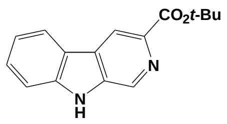

β-CCT

antagonist

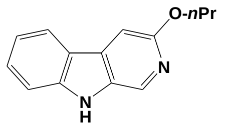

3-PBC

antagonist

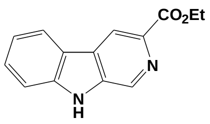

β-CCE

inverse agonist

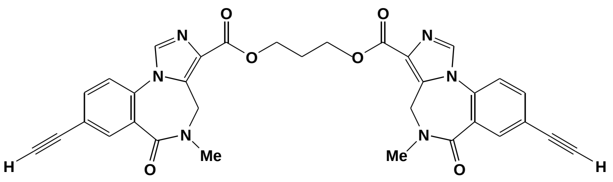

XLi-093

antagonist at α5

The alignments of several Bz BS ligands within this model are shown in Figure 9.

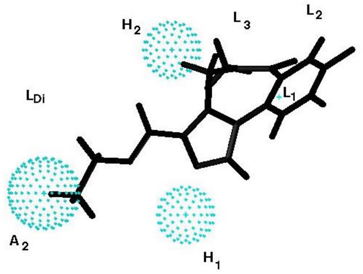

Ro 15-1788

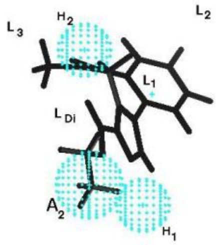

Ro 15-1788 rotated 90º

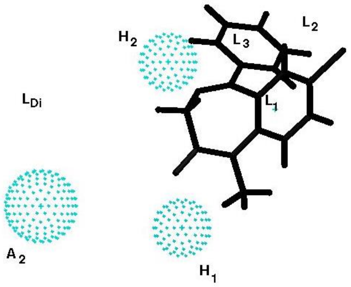

diazepam

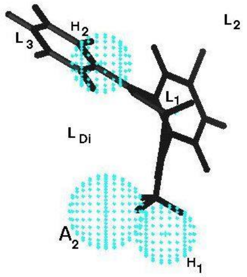

diazepam rotated 90º

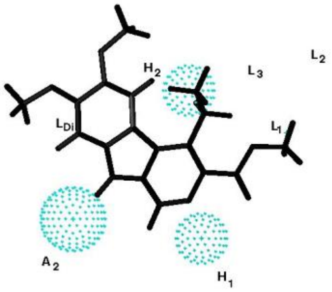

DMCM

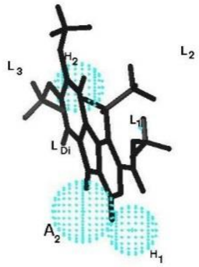

DMCM rotated 90º

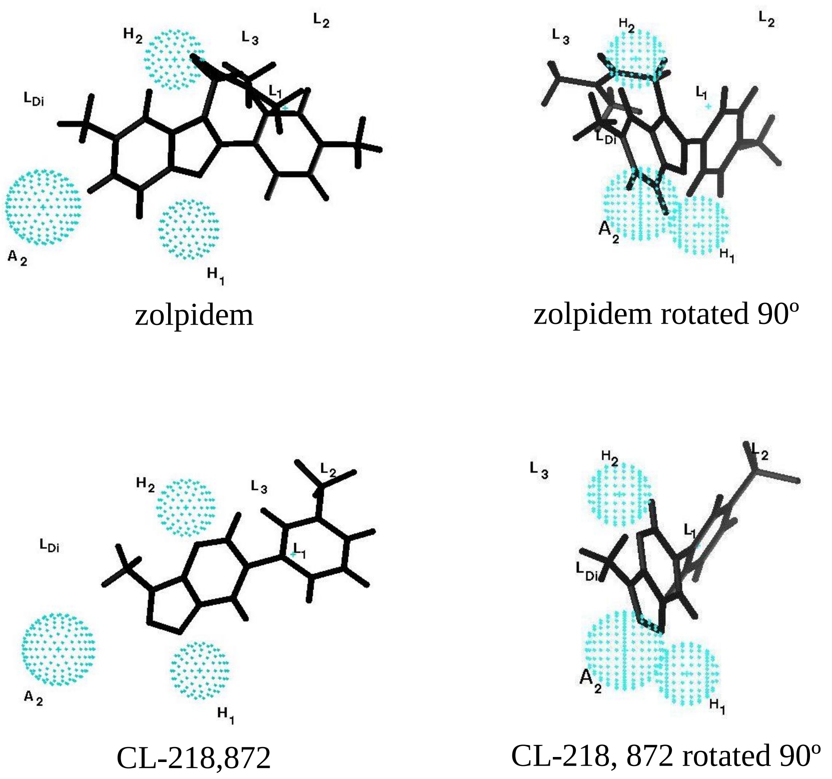

Figure 9. Alignments of several Bz BS ligands within the pharmacophore model.[25]

A unified pharmacophore model incorporating many substance classes that act at the DS and DI benzodiazepine binding sites of GABA(A) receptors has been updated to include new substance classes. Compound development guided by this pharmacophore model has led to new agents with interesting pharmacological profiles, particularly enhanced preference for α2 or α5 containing GABA(A) receptor subtypes. Based on the evaluation of experimental data and comparative models of the α1β2γ2 GABA(A) receptor, the location of several residues relative to the descriptors of the pharmacophore/receptor model has been proposed. Although no absolute assignments were made regarding which amino acids satisfy the pharmacophoric descriptors, experimental data strongly indicated definite trends with regard to how ligands of varying pharmacological activity are oriented within the receptor. Because the unified pharmacophore/receptor model accounts for the binding and activity profiles at the six GABA(A) receptor subtypes containing any one of the different alpha subunits, the proposed orientation should also be similar within the different models[1] of the various receptor subtypes. Information to be immediately gained from these proposed orientations can have far reaching benefits, not only for the rational design of selective ligands and the interpretation of ligand docking results, but also for the identification and evaluation of possible roles certain residues may have within the pocket. As structure determination of the GABA(A) receptor is eagerly awaited, it is hoped that these proposed orientations may be used by others to gain additional insight into the potential mechanisms underlying binding and modulation at the Bz site, all of

which will lead to a better understanding of the structure and function of GABA(A) receptors. In the next section the methods used to build the protein model will be explained.

Homology Models of the Benzodiazepine Receptor

The γ-aminobutyric-acid (GABA(A) and $GABA_C$) pentameric ligand gated ion channels are members of a superfamily of allosteric transmembrane proteins which includes the nicotinic acetylcholine (nAChR), serotonin 5-HT3, and glycine receptors. Electrophysiological data on GABA(A) is available but attempts at atomic resolution to acquire structural data have so far been unsuccessful. However, in 2001 Smit[38] discovered a homologue of the nACh receptor ligand-binding domain from the snail *Lymnaea stagnalis.* Acetylcholine-binding protein (AChBP) is produced in the glial cells of *Lymnaea stagnalis.* In the synaptic cleft, AChBP modulates synaptic transmission. Subunits of the snail glial cells form a stable homopentamer with conserved N terminal domains. Known agonists and antagonists of nAChRs bind to the homologue. For this reason, it is a valuable template for modeling the N-terminus domains of pentameric ligand gated ion channels.

Brejc *et al.* successfully solved the crystal structure of AChBP by X-ray crystallography using weak Pb multiple wavelength anomalous diffraction (MAD) data in two crystal forms.[1] The crystals were grown at room temperature using the hanging-drop vapour diffusion technique. The crystal structure revealed a radially symmetric homopentamer with extracellular dimensions of the nicotinic acetylcholine receptor (nAChR) which correlated with measurements taken using electron microscopy.[1]

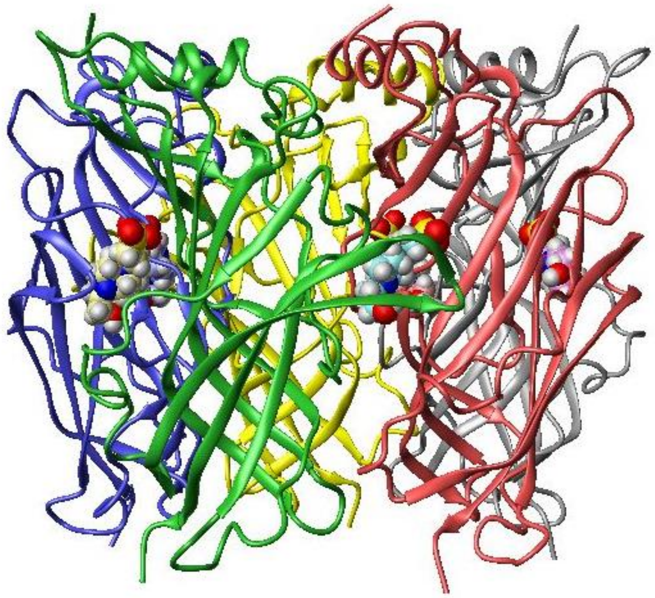

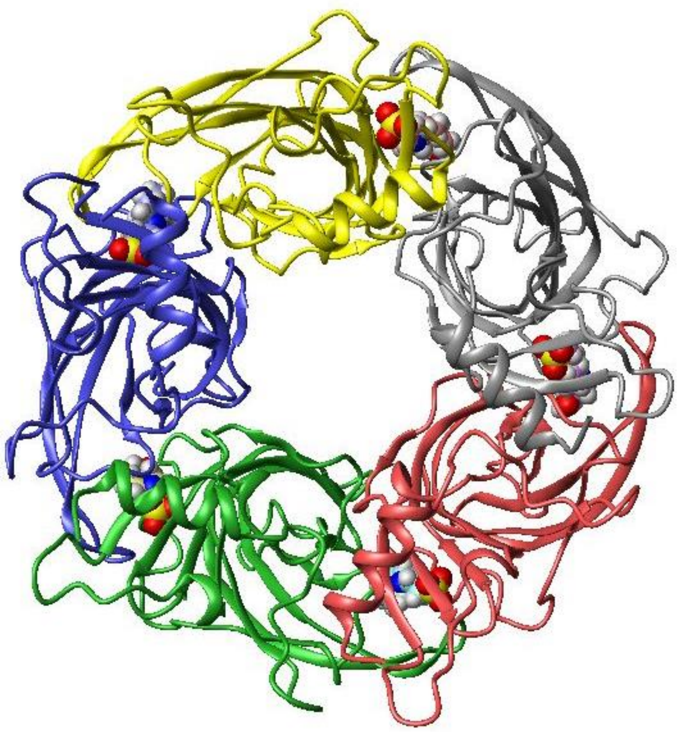

Figure 10. Orthogonal views of the homopentameric acetycholine binding protein crystallized from *Lymnaea stagnalis*. The subunits have been assigned different colors for clarity.[2]

Subunits are homologous and have been colored by chain to distinguish them. In space filling models, HEPES (N-2-hydroxyethylpeperazine-N'-2-ethanesulphonic acid (present during the crystallization process) can be seen bound in the ligand binding site (Figure 10) between each subunit interface.[2]

The N terminus is located at the "mouth" of the ion channel in the synaptic cleft. A molecule of HEPES was present in the acetylcholine binding pocket. This was verified as the residues implicated with agonist binding in nAChR and AChBP were conserved in the receptor. The residues lie in four regions identified as loops A, B, C, and D. Loops A, B, and C are located on what is referred to as the 'plus' face while loops D, E, and F lie on the 'minus' face (see Figures 11 and 12). In the heteromeric nAChR, agonist binding takes place between the 'plus' face of a subunit and the 'minus' face of an α or γ subunit. Site directed mutagenesis and radioligand binding assays have established that residues of the GABA(A) receptor involved in ligand binding align with residues in loops A, B, and C of the α subunit and loops D and E of the γ subunit.[2, 23, 25]

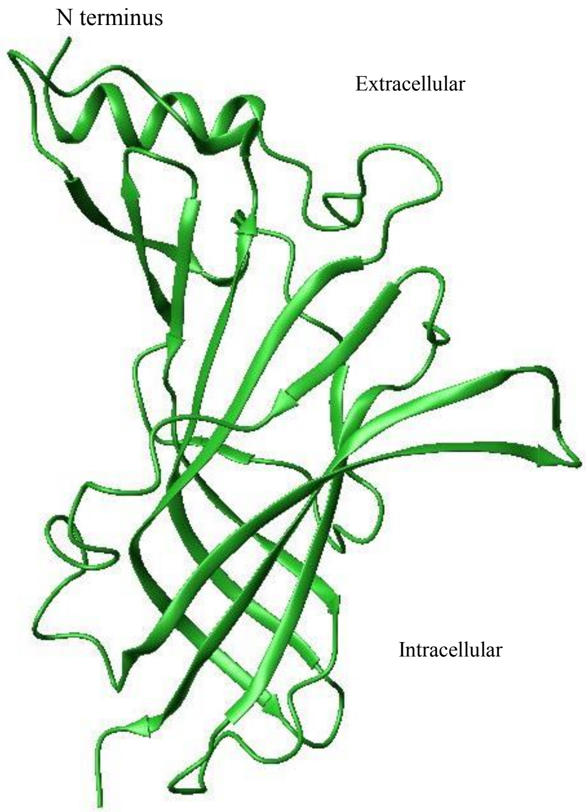

Figure 11. Single subunit of acetycholine binding protein crystallized from Lymnaea stagnalis (side view).[2,23]

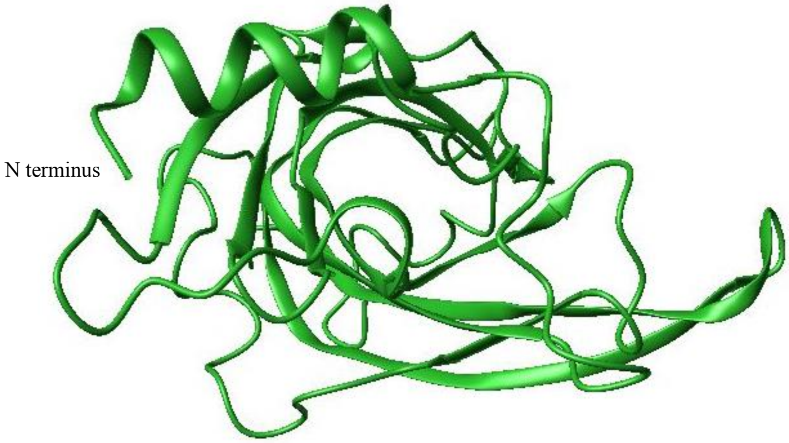

Figure 12. Single subunit of acetycholine binding protein crystallized from *Lymnaea stagnalis* (top view).

Since the AChBP is a member of the Cys-loop LGIC superfamily it offers a template for building models of the the ligand binding domains of the Cys-loop superfamily. The AChBP was used to build homology models of the subtypes of the GABA(A) receptors. The ligand binding domains of human GABA(A) receptors (α1, α2, α3, α5, β2, and γ2) were obtained from the Research Collaboratory for Structural Bioinformatics (RCSB). Originated in 1971 at

Brookhaven National Laboratories, this is the home of the Protein Data Bank. The PDB archive is the repository of information about the 3D structures of large biological molecules such as proteins and nucleic acids. With the protein sequences in hand, a critical first step in building a homology model is to align the raw protein sequence with the protein sequence of a target protein or template structure. Alignments are prepared using multiple sequence alignment software for DNA and proteins.[39]

After calculating the best match for the selected sequences, they are lined up so identities, similarities, and differences can be seen. Programs which perform this include MAFFT, Muscle, Multalin, ClustalW, and BlastAlign (http://pbil.univ-lyon1.fr/alignment.html). Templates can also be found by performing a query of the raw protein sequence against the Protein databank. This is performed using the protein database search program, Gapped Blast.[39, 40] The report will contain a list of sequences producing significant alignments, PDB code, protein description, a normalized alignment score and an Expect (E) value. In general, a lower E score describes the background noise that exists between sequences. A lower E value correlates to a more significant score. Once a proper alignment of protein sequences was performed, the raw protein sequence was threaded onto the AChBP using Deep View. The crystal structure of the AChBP was downloaded from the Swiss Protein Databank Viewer. Deep View is a Swiss Protein Databank Viewer (http://ca.expasy.org/spdbv). Threading is performed after the alignment is complete. Amino acids which have been identified as equivalent between the proteins are "fit". This is similar, but more precise than the "3 corresponding atoms" technique. Deep View includes a tool, *Magic Fit*,[39] which performs the threading procedure for all residues across a raw sequence and a reference protein. Once the preliminary model has been built, a manual inspection can be performed. Considerations during manual inspection include, identifying areas of low homology, and manually aligning gap regions with loops in the reference protein.[39, 40] Loop regions which correspond to gaps were in the alignment can be modeled by fitting structures from a loop database. In the current model, loop regions were mapped to a loop database by Cromer et al.[2, 15]

Upon completion of the preliminary model, the following properties can be studied using Deep View and Sybyl[25]:

1. Surface hydrophobicity
2. Solvent accessibility of N-glycosylation sites after pentamer assembly
3. Acidic,basic, and non polar maps
4. Sybyl X minimizations
5. Steric clashes
6. RMSD of alpha carbons
7. Location of disulfide bonds

In order to improve the prediction of areas of low homology PHD was utilized by Cromer et al.[2, 39, 40] PHD or more commonly referred to as PredictProtein is a sequence analysis tool and the prediction of protein structure and function. Protein sequences or alignments are submitted to Columbia University; PredictProtein returns multiple sequence alignments, PROSITE sequence motifs, low-complexity regions (SEG), nuclear localisation signals, regions lacking regular structure (NORS) and predictions of secondary structure, solvent accessibility, globular regions, transmembrane helices, coiled-coil regions, structural switch regions, disulfide-

bonds, sub-cellular localization, and functional annotations. Upon request the Rost group will perform fold recognition by prediction-based threading, CHOP domain assignments, predictions of transmembrane strands and inter-residue contacts are also available. PredictProtein is run by Burkhard Rost at Columbia University in New York (www.rostlab.org).[40] As an alternative, Swiss Model can be used to model subunit loop regions by searching structures from loop databases.

Once subunits were completed, a pentamer was constructed taking into consideration residues determined experimentally to be present in the allosteric and GABA binding site. On the α subunit, this includes αTyr160, αTyr210, γPhe77, and αSer205.[23, 25] After manually assembling the subunits and comparing an overlay with AChBP, the structure was energy minimized.[25, 39]

With protein receptor models for α1β3γ2, α2β3γ2, α3β3γ2, and α5β3γ2 completed a truly *unified* pharmacophore model was assembled for each receptor subtype. Using the Tripos Biopolymer software, the pharmacophore was manually aligned into each protein. With mutagenesis data on residue interaction on key ligands, the pharmacophore was carefully adjusted so as to recreate the protein-ligand interactions discussed previously. The protein models below represent the cumulative results of over 20 years of research. The proteins are oriented with the γ2 on the left and the α subunit on the right with loop C reaching over the middle to γ2. Diazepam has been docked in each receptor and the pharmacophore is visible.

Protein Models of the α1β3γ2, α2β3γ2, α3β3γ2 and α5β3γ2 Subtypes

Presented here are the complete unified protein-pharmacophore models. The Milwaukee-based pharmacophore has been inserted into each of the homology models for the receptor subtypes of the benzodiazepine receptor. The models show the similarities we expected. The two oxygens of serine 206 represent the hydrogen bond acceptor A_2. The hydrogen bond donor, H_1, is due to the hydroxyl group oxygens of Threonine 231 and Tyrosine 234. Threonine 193 is responsible for the H_2 hydrogen bond donor as its hydroxyl group coordinates with lone pairs of ligands. The imidazole ring of histidine 102 coordinates with phenyl rings of ligands through π–π stacking interactions.

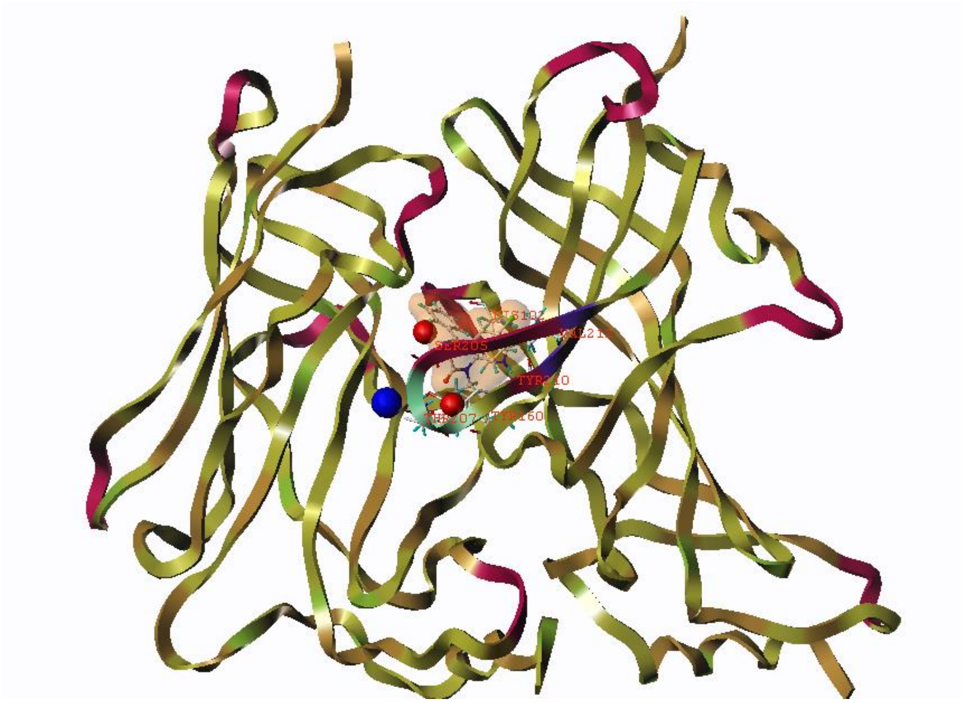

Figure 13. The α1β3γ2 subtype receptor with key residues shown

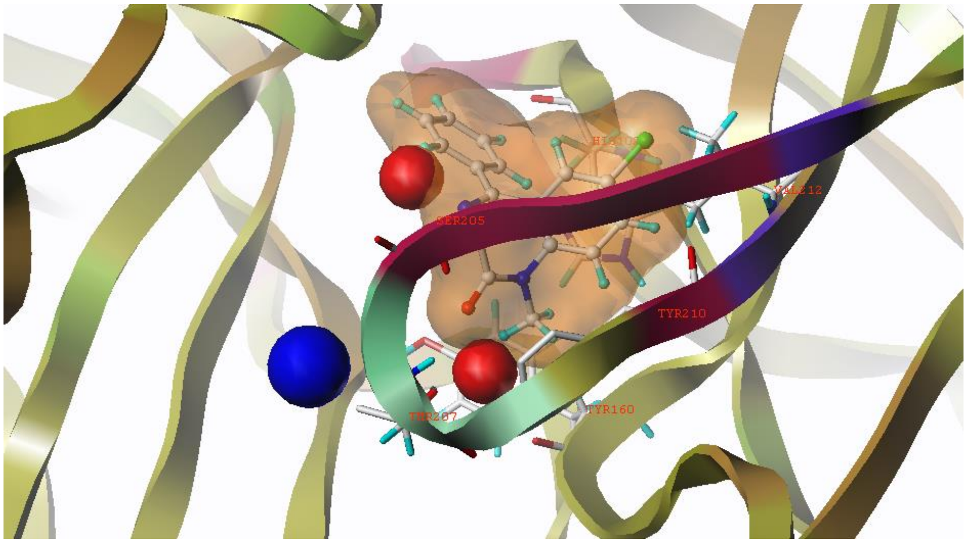

Figure 14. The benzodiazepine binding site of the α1β3γ2 receptor

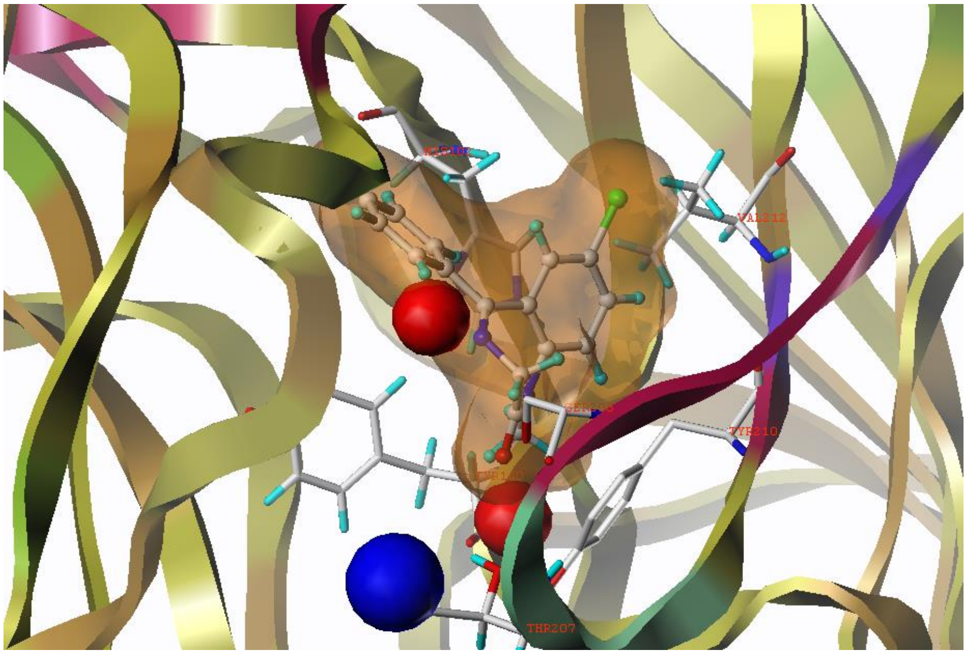

Figure 15. The benzodiazepine binding site of the α1β3γ2 receptor rotated 90°

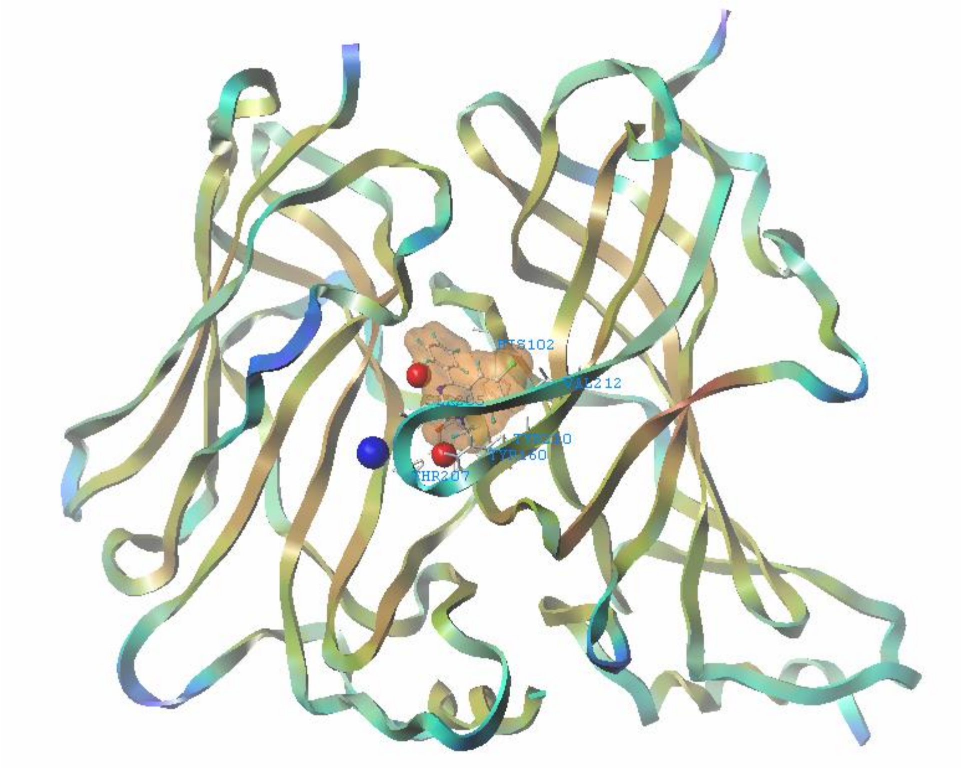

Figure 16. The α2β3γ2 subtype receptor with key residues shown

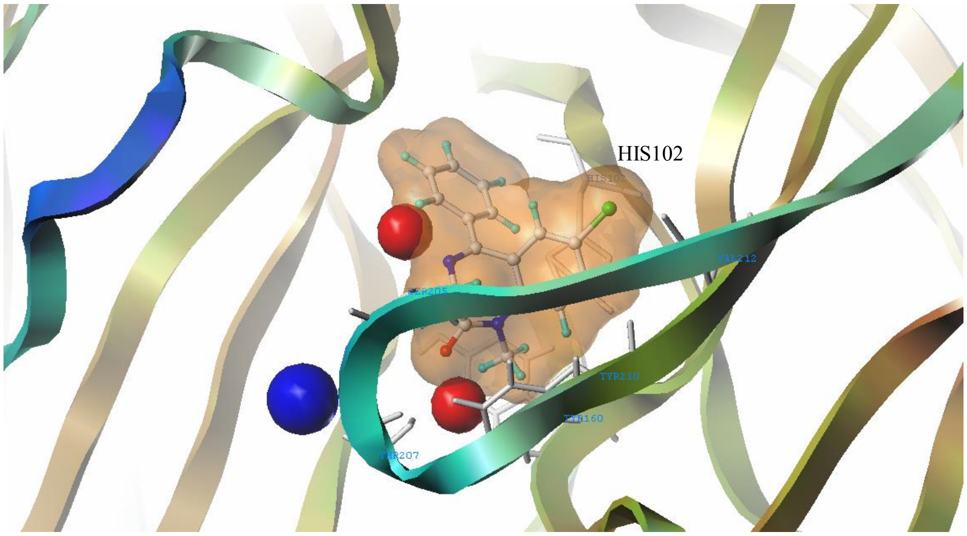

Figure 17. The benzodiazepine binding site of the α2β3γ2 receptor

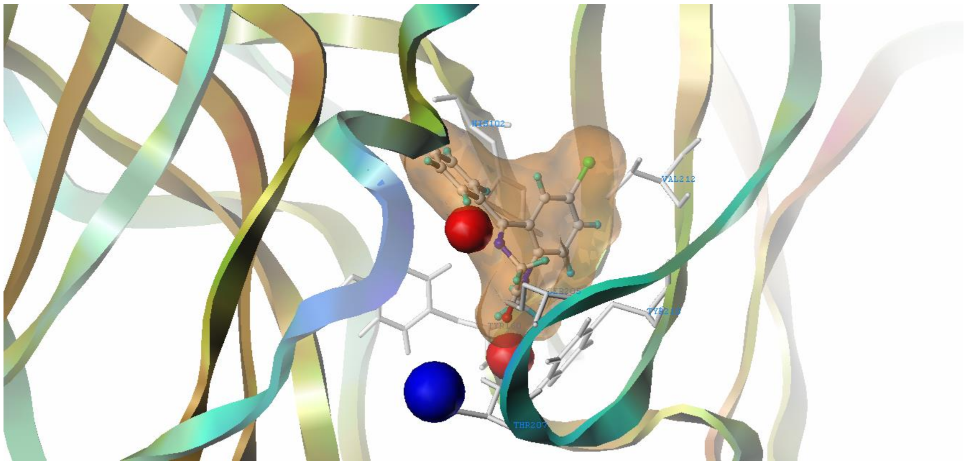

Figure 18. The benzodiazepine binding site of the α2β3γ2 receptor rotated 90°

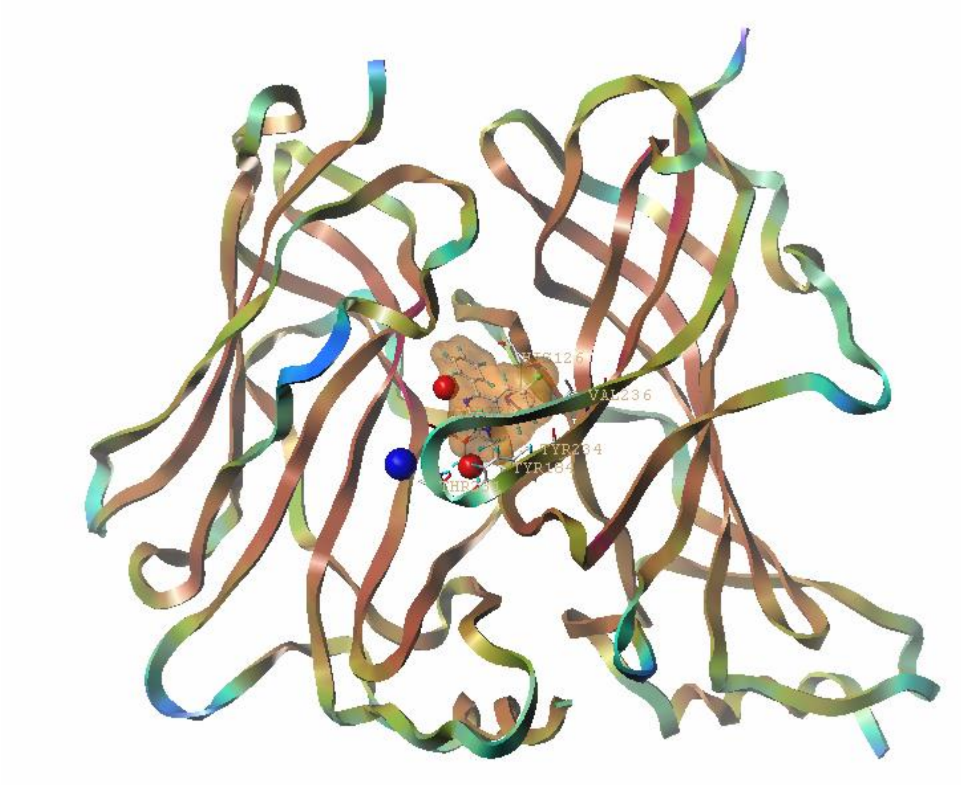

Figure 19. The α3β3γ2 subtype receptor with key residues shown

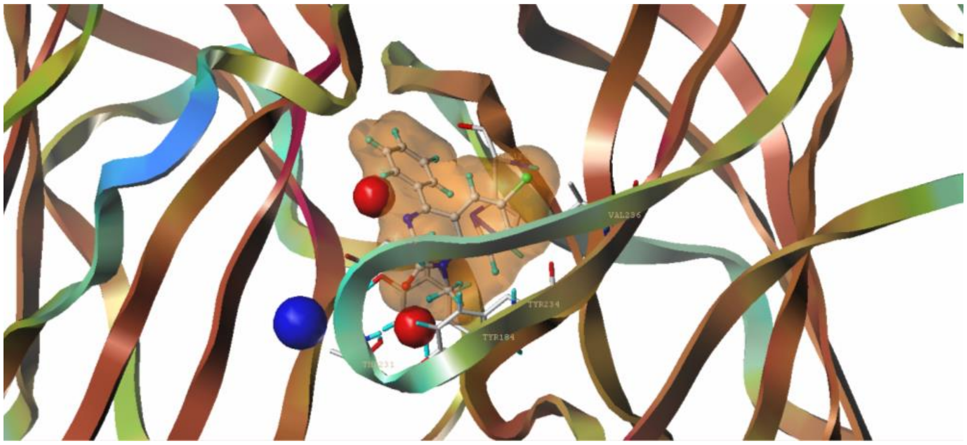

Figure 20. The benzodiazepine binding site of the α3β3γ2 receptor within the active site

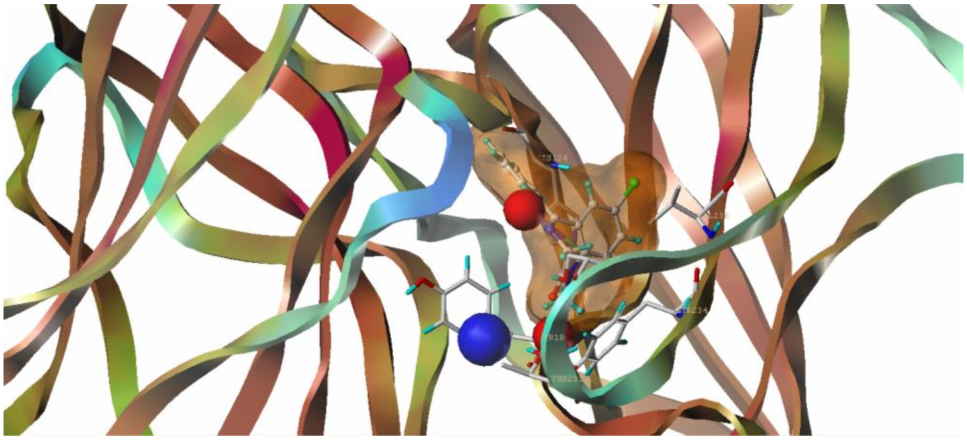

Figure 21. The benzodiazepine binding site of the α3β3γ2 receptor rotated 90°

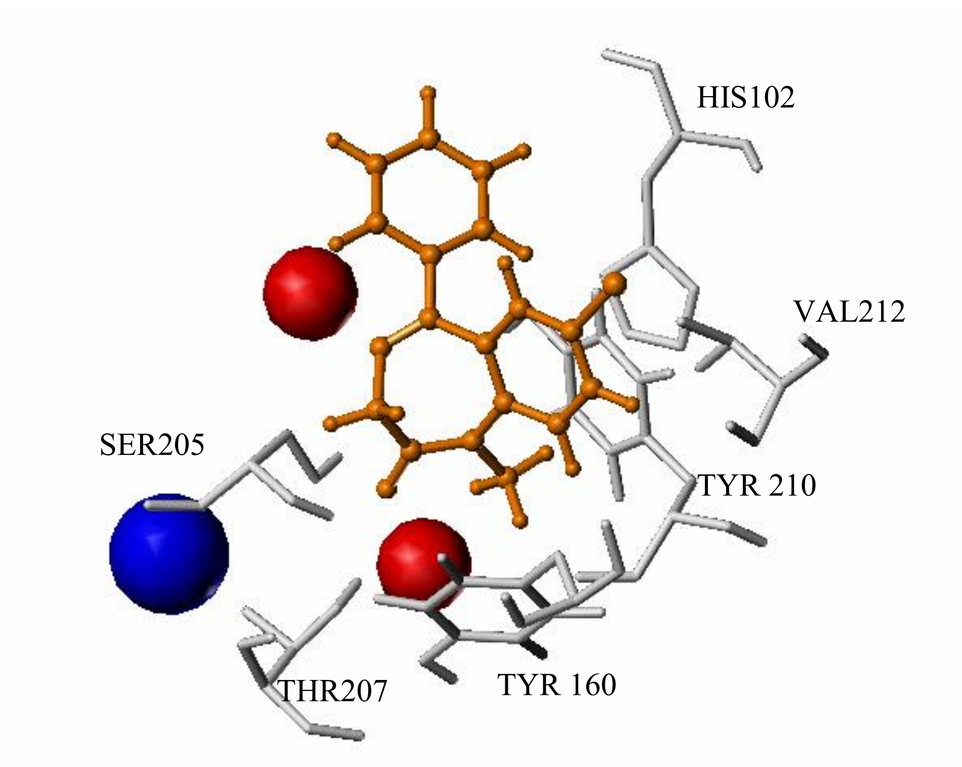

Figure 22. The benzodiazepine binding site of the α1β3γ2 receptor with diazepam in the binding pocket.

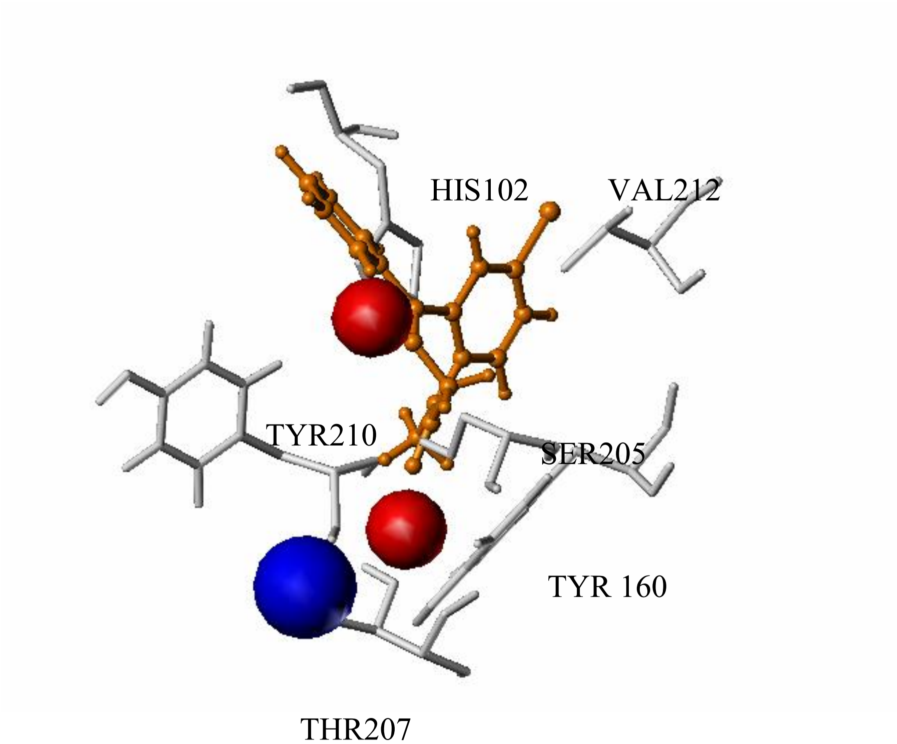

Figure 23. The benzodiazepine binding site of the α1β3γ2 receptor rotated 90° with diazepam in the binding site

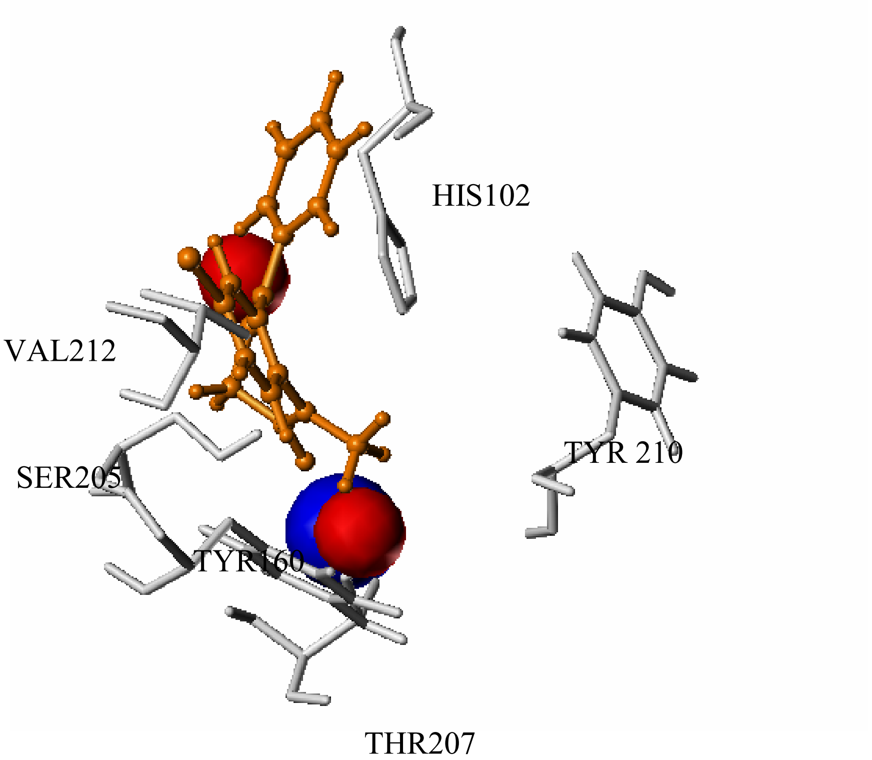

Figure 24. The benzodiazepine binding site of the α1β3γ2 receptor rotated 90° (reverse direction) with diazepam in the binding site

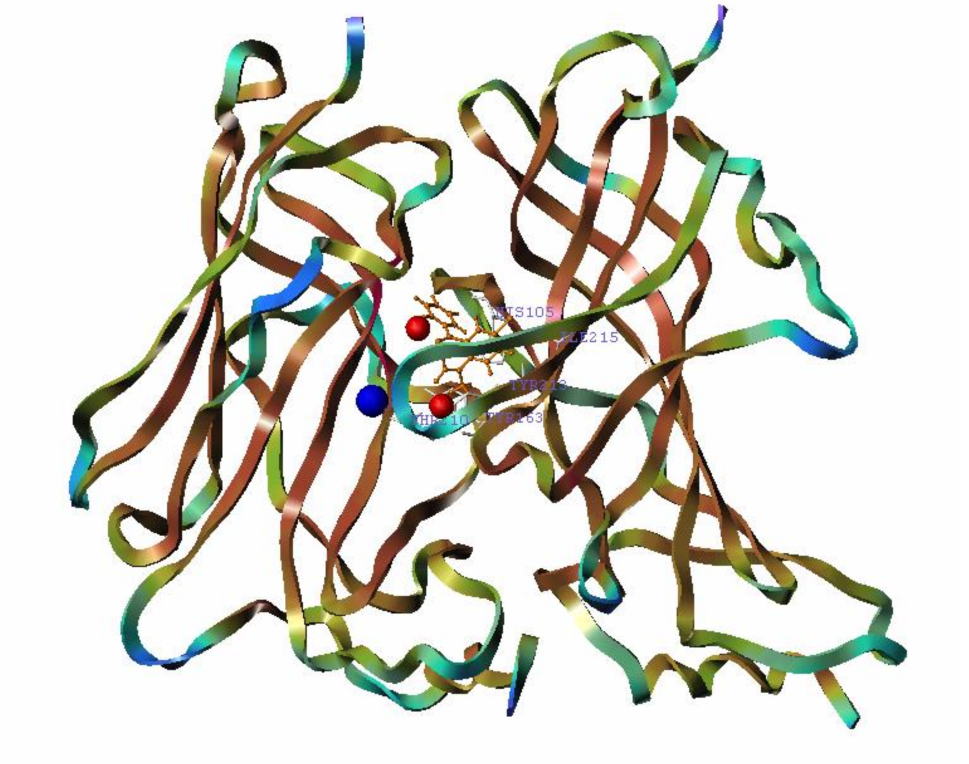

Figure 25. The α5β3γ2 subtype receptor with key residues shown with diazepam in the binding site

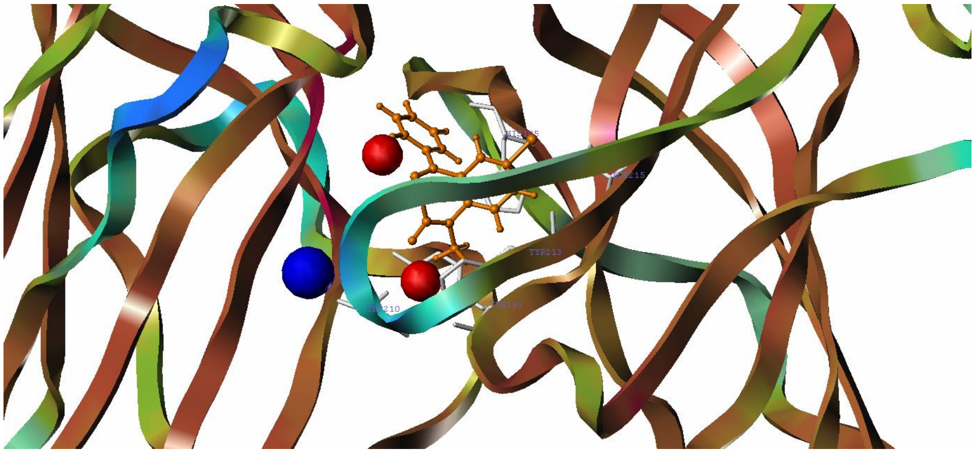

Figure 26. The benzodiazepine binding site of the α5β3γ2 receptor with diazepam in the binding site

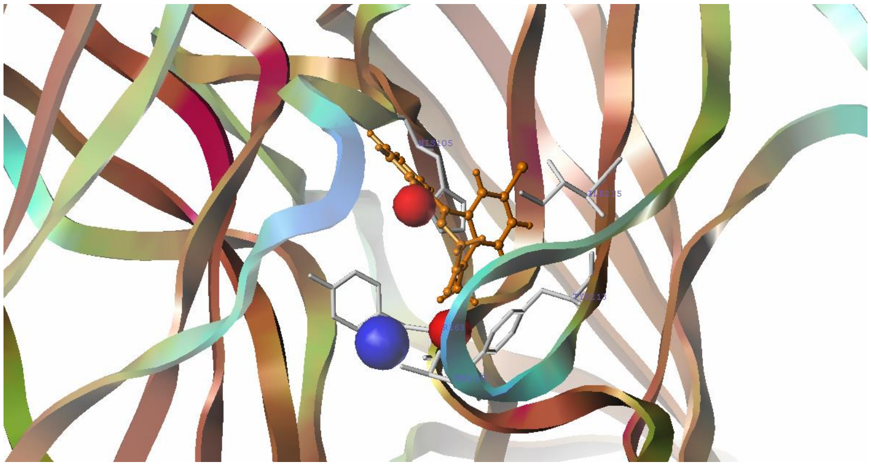

Figure 27. The benzodiazepine binding site of the α5β3γ2 receptor rotated 90° with diazepam in the binding site

From this unified model which was constructed on binding data from hundreds of compounds, included volume analysis, site directed mutagenesis, manual as well as Sybyl Flexidock and AutoDock algorithms, one can now see how any compound will dock in the benzodiazepine binding site of GABA(A) . Presented below is a compound for further research on drug abuse, WYS8.

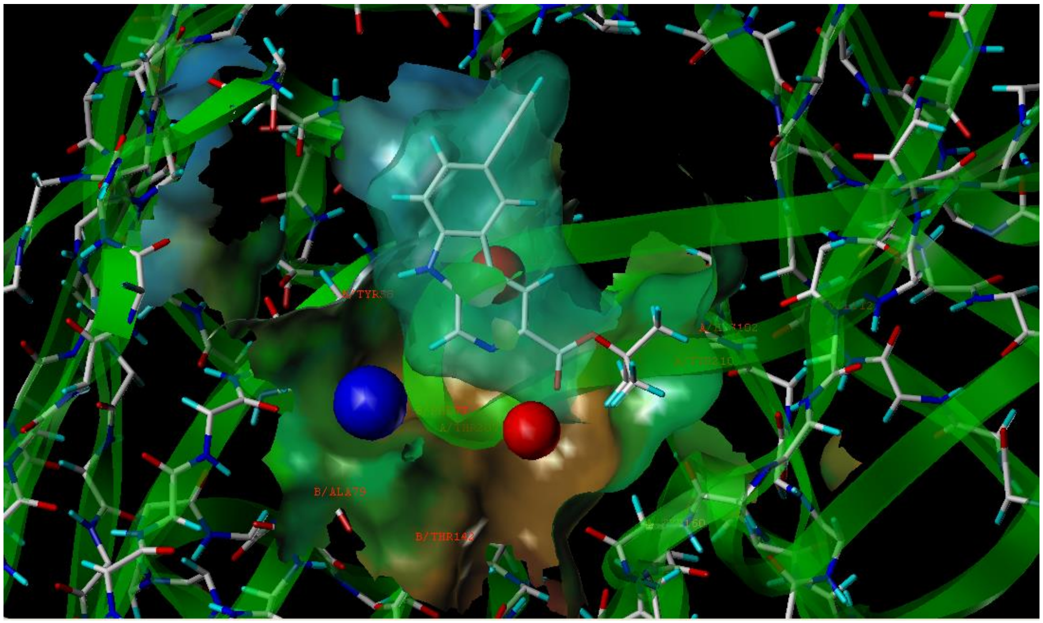

Figure 28. Flexidock fit of WYS8

Refinement of WYS8 in the unified pharmacophore receptor was completed using FlexiDock. The protein with the pharmacophore set inside the benzodiazepine receptor is displayed with a ligand docked to the pharmacophore. From the compute function, Flexidock is executed. The pocket needs to be defined for Sybyl if it has not already. Pick atoms which are located near the binding site. All atoms within 4 Angstroms will be automatically selected by Sybyl to assist. For Flexidock to work with rigid proteins the following steps must be taken:

1. Water must be removed
2. Select atoms around the binding pocket
3. Add hydrogens
4. Pick 4Å radius to display around binding pocket
5. Have Sybyl add charges if necessary
6. Set rotatable bonds

From these models, the individual pharmacophores, and the compound database the medicinal chemist has a powerful set of tools to determine future ligands to synthesize in order to deliver subtype selective drugs with reduced side effects. For example, knowing how to dock ligands into the pharmacophore, one can begin taking measurements of ligands and residues and analyzing interactions. In the $\alpha2\beta2\gamma2$ Bz receptor His 102 and the phenyl ring of diazepam have a π stacking distance of 4.1 Angstroms. Next we will discuss how one can properly dock ligands into the binding site.

Docking Studies Using AutoDock 4.2

Docking of ligands into the new binding sites of the homology models was also executed using the suite of *Autodock Tools* developed at Scripps Institute.[41, 42] The general procedure entails preparing *pdb* file formats of the receptor (protein) as well as the ligand. Once these docking grids and docking parameters are set, the docking run will produce multiple potential docking reports. Prior knowledge from SAR studies and mutagenesis studies is then used to select those for a parallel run (clusters) and a final docking prediction is developed.

Preparing the Ligand for Autodock

The ligand is loaded into Autodock as a pdb file. Once in *Autodock*, Gasteiger partial charges are determined along with aromatic carbons, rotatable bonds and torsional degrees of freedom. A new "pdgqt" file is created which has this information attached to it. Preparation of the receptor begins with loading it too as a pdb file into Autodock. Water molecules must be removed from the protein if present for *Autodock* processing. If any residues are flexible, these need to be identified in the receptor. Once identified, torsion angles can be assigned. Bonds in side chains can be manually or automatically selected as rotatable, unrotatable, or non-rotatable. *Autodock* needs two files when docking ligands in proteins with flexible residues, a flexible PDBQT file and a Rigid PDBQT file. Before docking can begin the receptor is checked for Gasteiger charges. A map is created which identifies possible hydrogen bonding interactions.

Next, a grid box (Figure 29) is constructed around the general vicinity of the receptor if this is known.

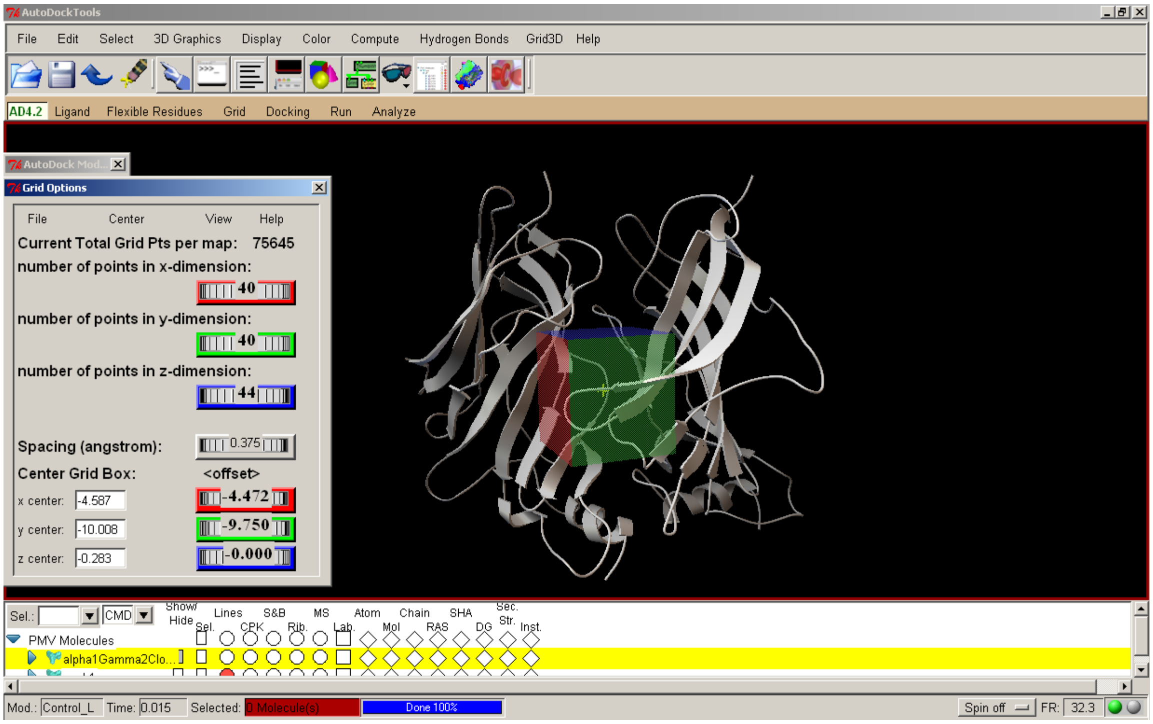

Figure 29. Setting the autogrid in Autodock for the Bz BS protein

With all the appropriate information stored in a parameter file, the docking program will know which map file to use, details about the ligand rules, the potential presence of flexible residues and which docking algorithm to use. Four different docking algorithms are currently available in AutoDock. Monte Carlo simulated annealing (SA) was used. Briefly, the ligand makes random moves in the receptor and the energy of the new position is compared to the old one. Moves in which the energy is lowered are accepted.

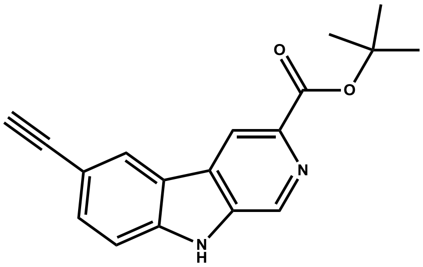

Figure 30. WYS8

Docking WYS8

WYS8 was drawn in ChemDraw Ultra 8.0 (Figure 30). Lone pairs were added and the molecule was minimized in ChemDraw 3D using MM2 to a minimum RMS gradient of 0.100. This was converted to a PDB file to be loaded into AutoDock 4. Upon loading into Autodock,

Gasteiger charges were added, 14 non-polar hydrogens were merged, 10 aromatic carbons were identified, 3 rotatable bonds were detected, and TORSDOF was set to 3.

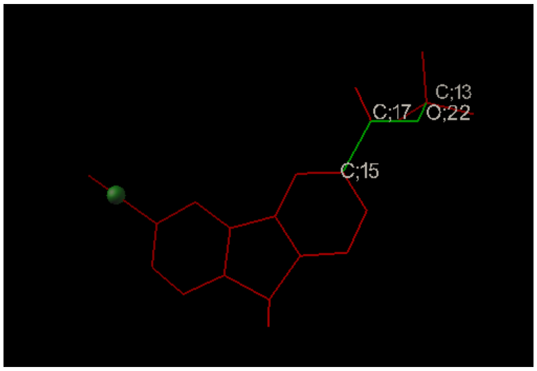

Figure 31. Rotatable bonds are identified along with root atom in ligand WYS8

The root atom with the smallest largest sub-tree (in this case an acetylenic hydrogen) is marked with a green sphere and the root portion (red) is identified. The rotatable bonds are colored green. The receptor was rigid for this analysis.

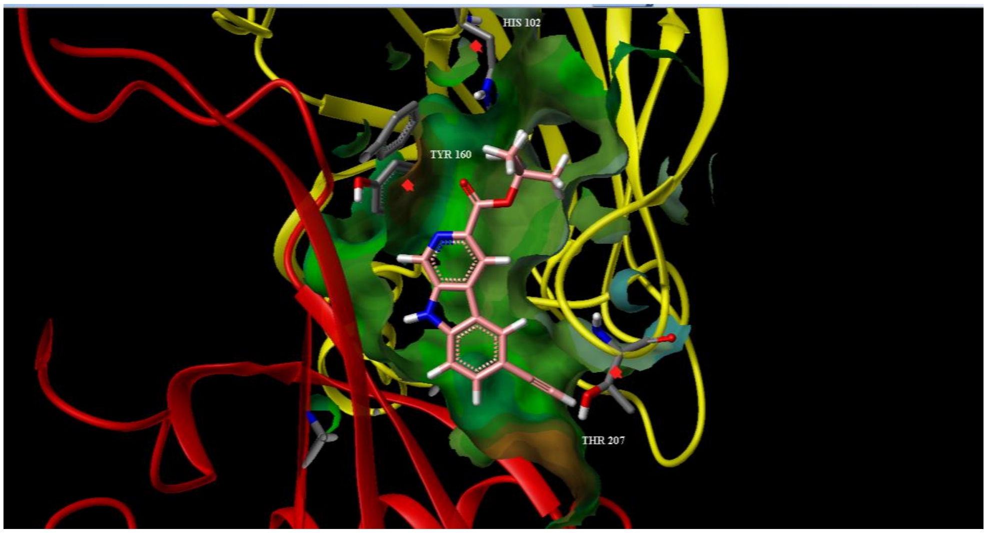

Figure 32. AutoDock result rendering was performed using Tripos Benchware.

Using Tripos Benchware 3D Explorer 2.6, one of the results was modeled (Figure 32). Key residues were identified and the protein surface five angstroms from the ligand was mapped showing its lipophilic potential. The results using AutoDock and a rigid protein will be much improved when flexible residues are allowed in the binding pocket along with rotatable bonds. The docking in Figure 28 is preferred when considering the orientation in the Milwaukee-based pharmacophore.

Homology models which have the pharmacophore aligned in the binding pocket have been presented for the α1β2γ2, α2β2γ2, α3β2γ2, and α5β2γ2 GABA(A) receptors. The binding site was constructed in light of years of work including structure-activity-relationships (SAR), site directed mutagenesis, and docking studies of monomer and dimers alike. The

$\alpha4\beta2\gamma2$ and $\alpha6\beta2\gamma2$ receptors have not been made as these "diazepam-insensitive" binding sites are not a focus of our research to date. An updated included volume analysis has been presented using ligands from 15 different structural families. The new included volume analyses and the unified pharmacophore protein models will continue to drive the design of novel and selective benzodiazepine receptor ligands. From these new ligands, scientists will increase their understanding of the physiological responses of the specific receptors, the design of pharmaceuticals with reduced side effects, and treatments for schizophrenia, drug addiction and Alzheimer's disease. The $\alpha5\beta2\gamma2$ receptors are of particular interest because of their concentration in the hippocampus. The discovery of a larger L_3 pocket in the $\alpha5\beta2\gamma2$ receptor versus the other subtypes will provide medicinal chemists with a structural advantage in designing more subtype selective ligands. From the unified pharmacophore protein models presented here, the design of new compounds for the GABA(A) will be more productive than ever.

Future Work

Screening and modeling of more subtype selective ligands will further our current understanding of the benzodiazepine receptor and its function. Continued work on the molecular spreadsheet which will include all the compounds designed in the Milwaukee laboratory as well as other organizations will create a powerful QSAR algorithm for answering the age old question, "What compound should we make next?" This study will need to include all binding and non-binding compounds in order to create a predictive program with a high degree of accuracy (r^2, q^2) in predicting activity.

Site directed mutagenesis needs to be continued in which receptors of all subtypes are tested to determine how a residues substitution affects each of the subtypes. Such experimentation will shed further light on how ligands are positioned in the binding pocket and what residues they are interacting with. Adding to the new pharmacophore protein alignments which can now be executed, docking runs using *Autodock* in which the residues in the receptor are set to flexible may give further information as to docking preferences of ligands helping to better understand key interactions in each of the different subtypes.

All of these activities have allowed us to develop the current ligands as well as propose next generation ligands. The new pharmacophores will aid in furthering our understanding of GABA and in designing better drugs for the CNS until a complete GABA(A) protein receptor ion channel is captured in high resolution.

References

1. Brejc, K.; van Dijk, W.; Klassen, R.; Schuurmans, M.; van Der Oost, J.; Smit, A. B.; Sixma, T., Crystal structure of ACh-binding protein reveals the ligand-binding domain of nicotinic receptors. *Nature* **2001,** 411, 269-276.
2. Cromer, B.; Morton, C.; Parker, M. W., Cromer's model, Anxiety of GABA(A) receptor structure relieved by AChBP. *Trends Biochem. Sci.* **2002,** 27, 280-287.
3. Grutter, T.; de Carvalho, L. P.; Dufresne, V.; Taly, A.; Fischer, M.; Changeux, J. P., A chimera encoding the fusion of an acetylcholine-binding protein to an ion channel is stabilized in a state close to the desensitized form of ligand-gated ion channels. *Comptes Rendus Biologies* **2005,** 328, (3), 223-234.
4. Trudell, J. R., Unique assignment of inter-subunit association in GABA(Alpha) alpha 1 beta 3 gamma 2 receptors determined by molecular modeling. *Biochim. Biophys. Acta* **2002,** 1565, (1), 91-96.
5. Ernst, M.; Brauchart, D.; Boresch, S.; Sieghart, W., Comparative modeling of $GABA_A$ receptors: Limits, insights, future developments. *Neuroscience* **2003,** 119, (4), 933-943.
6. Sigel, E., Mapping of the benzodiazepine recognition site on $GABA_A$ receptors. *Curr. Top. Med. Chem.* **2002,** 2, 833-840.
7. Sigel, E.; Buhr, A., The benzodiazepine binding site of GABA(A) receptors. *Trends Pharmacol. Sci.* **1997,** 18, (11), 425-429.
8. Baumann, S. W.; Baur, R.; Sigel, E., Forced subunit assembly in $\alpha 1\beta 2\gamma 2$ $GABA_A$ receptors. *J. Biol. Chem.* **2002,** 277, (48), 46020-46025.
9. Neish, C. S.; Martin, I. L.; Davies, M.; Henderson, R. M.; Edwardson, J. M., Atomic force microscopy of ionotropic receptors bearing subunit-specific tags provides a method for determining receptor architecture. *Nanotechnol.* **2003,** 14, (8), 864-872.
10. Chou, K. C., Modelling extracellular domains of GABA-A receptors: subtypes 1, 2, 3, and 5. *Biochem. Biophys. Res. Commun.* **2004,** 316, (3), 636-642.
11. Buhr, A.; Baur, R.; Malherbe, P.; Sigel, E., Point mutations of the alpha 1 beta 2 gamma 2 gamma-aminobutyric acid(A) receptor affecting modulation of the channel by ligands of the benzodiazepine binding site. *Mol. Pharmacol.* **1996,** 49, (6), 1080-1084.
12. Sigel, E.; Baur, R.; Trube, G.; Möhler, H.; Malherbe, P., The effect of subunit composition of rat-brain GABA-A receptors on channel function. *Neuron* **1990,** 5, 703-711.
13. Sieghart, W., Structure and pharmacology of gamma-aminobutyric $Acid_A$ receptor subtypes. *Pharmacol. Rev.* **1995,** 47, (2), 181-234.

14. Khom, S.; Baburin, I.; Timin, E. N.; Hohaus, A.; Sieghart, W.; Hering, S., Pharmacological properties of GABA(A) receptors containing gamma 1 subunits. *Mol. Pharmacol.* **2006,** 69, (2), 640-649.
15. Unwin, N., Refined structure of the nicotinic acetylcholine receptor at 4A resolution. *J. Mol. Biol.* **2005,** 346, (4), 967-89.
16. Hagen, T. J. PhD Thesis. University of Wisconsin, Milwaukee, Milwaukee, 1988.
17. Trudell, M. L. PhD Thesis. University of Wisconsin - Milwaukee, Milwaukee, 1989.
18. Hansen, S. B.; Sulzenbacher, G.; Huxford, T.; Marchot, P.; Taylor, P.; Bourne, Y., Structures of Aplysia AChBP complexes with nicotinic agonists and antagonists reveal distinctive binding interfaces and conformations. *Embo J* **2005,** 24, (20), 3635-46.
19. Mihic, S. J.; Whiting, P.; Klein, R. L.; Wafford, K.; Harris, R. A., A single amino acid of the human γ-aminobutyric acid type A receptor γ2 subunit determines benzodiazepine efficacy. *J. Biol. Chem.* **1994,** 269, 32768-32773.
20. Minier, F.; Sigel, E., Positioning of the α-subunit isoforms confers a functional signature to γ-aminobutyric acid type A receptors. *Proc. Natl. Acad. Sci. U.S.A.* **2004,** 101, (20), 7769-7774.
21. Atack, J. R., The benzodiazepine binding site of $GABA_A$ receptors as a target for the development of novel anxiolytics. *Expert Opin. Invest. Drugs* **2005,** 14, (5), 601-618.
22. Downing, S.; Lee, Y. F.; Farb, D. H.; Gibbs, T. T., Benzodiazepine modulation of partial agonist efficacy and spontaneously active $GABA_A$ receptors supports an allosteric model of modulation. *Br. J. Pharmacol.* **2005,** 145, 894-906.
23. He, X. H.; Zhang, C. C.; Cook, J. M., Model of the BzR binding site: Correlation of data from site-directed mutagenesis and the pharmacophore/receptor model. *Med. Chem. Res.* **2001,** 10, (5), 269-308.
24. Ernst, M.; Bruckner, S.; Boresch, S.; Sieghart, W., Comparative models of GABA(A) receptor extracellular and transmembrane domains: Important insights in pharmacology and function. *Mol. Pharmacol.* **2005,** 68, (5), 1291-1300.
25. Clayton, T.; Chen, J. L.; Ernst, M.; Richter, L.; Cromer, B. A.; Morton, H. N.; Kaczorowski, C. C.; Helmstetter, F. J.; Furtmuller, R.; Ecker, G.; Parker, M. W.; Sieghart, W.; Cook, J. M., Analysis of the Benzodiazepine Binding Site on γ-Aminobutyric acidA Receptors: Correlation of Experimental Data with Pharmacophore and Comparative Models. *Curr. Med. Chem.* **2007,** 14, (26), 2755-2775.
26. Haefely, W., Kyburz, E., Gerecke, M., and Mohler, H. , *Recent Advances in the Molecular Pharmacology of Benzodiazepine Receptors and in the Structure-Activity Relationships of their Agonists and Antagonists*. Academic Press: New York, 1985; Vol. 99, p 165-322.

27. Kucken, A. M.; Teissere, J. A.; Seffinga-Clark, J.; Wagner, D. A.; Czajkowski, C., Structural requirements for imidazobenzodiazepines binding to $GABA_A$ receptors. *Mol. Pharmacol.* **2003,** 63, 289-296.
28. Kucken, A. M.; Wagner, D. A.; Ward, P. R.; Teissere, J. A.; Boileau, A. J.; Czajkowski, C., Identification of benzodiazepine binding site residues in the gamma 2 subunit of the gamma-aminobutyric acid(A) receptor. *Mol. Pharmacol.* **2000,** 57, (5), 932-939.
29. Sawyer, G. W.; Chiara, D. C.; Olsen, R. W.; Cohen, J. B., Identification of the bovine gamma-aminobutyric acid type A receptor alpha subunit residues photolabeled by the imidazobenzodiazepine [^{3}H] Ro15-4513. *J. Biol. Chem.* **2002,** 277, (51), 50036-50045.
30. Sancar, F.; Ericksen, S. S.; Kucken, A. M.; Teissere, J. A.; Czajkowski, C., Structural Determinants for High-Affinity Zolpidem Binding to GABA-A receptors. *Mol. Pharmacol.* **2007,** 71, (1), 38-46.
31. Zhang, P. W.; Zhang, W. J.; Liu, R. Y.; Harris, B.; Skolnick, P.; Cook, J. M., Synthesis and SAR study of novel imidazobenzodiazepines at 'diazepam-insensitive' benzodiazepine receptors. *J. Med. Chem.* **1995,** 38, (10), 1679-1688.
32. Zhang, W.; Diaz-Arauzo, H.; Allen, M. S.; Koehler, K. F.; Cook, J. M., *Chemical and computer assisted development of the inclusive pharmacophore of benzodiazepine receptors, in Studies in Medicinal Chemistry, M.I., Choudhary, Editor.* Harwood Academic Publishers: 1996; p 303.
33. Zhang, W.; Koehler, K. F.; Zhang, P.; Cook, J. M., Development of a comprehensive pharmacophore model for the benzodiazepine receptor. *Drug Des. Discov.* **1995,** 12, (3), 193-248.
34. Allen, M. S.; Tan, Y. C.; Trudell, M. L.; Narayanan, K.; Schindler, L.; Martin, M. J.; Schultz, C. A.; Hagen, T. J.; Koehler, K. F.; Codding, P.; Skolnick, P.; Cook, J., Synthetic and computer-assisted analyses of the pharmacophore for the benzodiazepine receptor inverse agonist site. *J. Med. Chem.* **1990,** 33, 2343-2357.
35. Zhang, P. W., Synthesis of Novel Imidazobenzodiazepines as Probes of the Pharmacophore for "Diazepam-Insensitive" $GABA_A$ Receptors. *J. Med. Chem.* **1995,** 38, 1679-1688.
36. Allen, M. S.; Laloggia, A. J.; Dorn, L. J.; Martin, M. J.; Constantino, G.; Hagen, T. J.; Koehler, K. F.; Skolnick, P.; Cook, J. M., Predictive binding of β-carboline inverse agonists and antagonists via the CoMFA/GOLPF approach. *J. Med. Chem.* **1992,** 35, 4001-4010.
37. Wong, G.; Koehler, K. F.; Skolnick, P.; Gu, Z. Q.; Ananthan, S.; Schönholzer, P.; Hunkeler, W.; Zhang, W. J.; Cook, J. M., Synthetic and computer-assisted analysis of the structural requirements for selective, high-affinity ligand-binding to diazepam-insensitive benzodiazepine receptors. *J. Med. Chem.* **1993,** 36, (13), 1820-1830.

38. Smit, A. B., A glia-derived acetylcholine-binding protein that modulates synaptic transmission. *Nature* **2001,** 411, (261-268).
39. Guex, N.; Peitsch, M. C., SWISS-MODEL and the Swiss-PdbViewer: An environment for comparative protein modeling. *Electrophoresis* **1997,** 18, 2714-2723.
40. Rost, B.; Yachdav, G.; Liu, L., The PredictProtein server. *Nucleic Acids Research* **2004,** 32 (suppl 2), W321-W326.
41. Goodsell, D. S.; Olson, A. J., Automated docking of substrates to proteins by simulated annealing. *Proteins: Structure, Function, and Genetics* **1990,** 8, 195-202.
42. Morris, G. M.; Goodsell, D. S.; Halliday, R. S.; Huey, R.; Hart, W. E.; Belew, R. K.; Olson, A. J., Automated docking using Lamarckian genetic algorithm and an empirical binding free energy function. *J. Comp. Chem* **1998,** 19, 1639-1662.

Appendix I. Key Amino Acid Sequences

Data Table 1. Amino acid sequence of the alpha subunit of the acetylcholine receptor in the *lymnaea stagnalis* (Great Pond Snail)

>sp|P58154|ACHP_LYMST Acetylcholine-binding protein OS=Lymnaea stagnalis PE=1 SV=1
MRRNIFCLACLWIVQACLSLDRADILYNIRQTSRPDVIPTQRDRPVAVSVSLKFINILEV
NEITNEVDVVFWQQTTWSDRTLAWNSSHSPDQVSVPISSLWVPDLAAYNAISKPEVLTPQ
LARVVSDGEVLYMPSIRQRFSCDVSGVDTESGATCRIKIGSWTHHSREISVDPTTENSDD
SEYFSQYSRFEILDVTQKKNSVTYSCCPEAYEDVEVSLNFRKKGRSEIL

Data Table 2. Amino acid sequence of the alpha subunit of the acetylcholine receptor in the *Torpedo californica* (Pacific Electric Ray)

>sp|P02710|ACHA_TORCA Acetylcholine receptor subunit alpha OS=Torpedo californica GN=CHRNA1 PE=1 SV=1
MILCSYWHVGLVLLLFSCCGLVLGSEHETRLVANLLENYNKVIRPVEHHTHFVDITVGLQ
LIQLISVDEVNQIVETNVRLRQQWIDVRLRWNPADYGGIKKIRLPSDDVWLPDLVLYNNA
DGDFAIVHMTKLLLDYTGKIMWTPPAIFKSYCEIIVTHFPFDQQNCTMKLGIWTYDGTKV
SISPESDRPDLSTFMESGEWVMKDYRGWKHWVYYTCCPDTPYLDITYHFIMQRIPLYFVV
NVIIPCLLFSFLTGLVFYLPTDSGEKMTLSISVLLSLTVFLLVIVELIPSTSSAVPLIGK
YMLFTMIFVISSIIITVVVINTHHRSPSTHTMPQWVRKIFIDTIPNVMFFSTMKRASKEK
QENKIFADDIDISDISGKQVTGEVIFQTPLIKNPDVKSAIEGVKYIAEHMKSDEESSNAA
EEWKYVAMVIDHILLCVFMLICIIGTVSVFAGRLIELSQEG

Data Table 3. Amino acid sequence of the alpha 1 subunit of the rat GABA(A) receptor

>sp|P62813|GBRA1_RAT Gamma-aminobutyric acid receptor subunit alpha-1 OS=Rattus norvegicus GN=Gabra1 PE=1 SV=1
MKKSRGLSDYLWAWTLILSTLSGRSYGQPSQDELKDNTTVFTRILDRLLDGYDNRLRPGL
GERVTEVKTDIFVTSFGPVSDHDMEYTIDVFFRQSWKDERLKFKGPMTVLRLNNLMASKI
WTPDTFFHNGKKSVAHNMTMPNKLLRITEDGTLLYTMRLTVRAECPMHLEDFPMDAHACP
LKFGSYAYTRAEVVYEWTREPARSVVVAEDGSRLNQYDLLGQTVDSGIVQSSTGEYVVMT
THFHLKRKIGYFVIQTYLPCIMTVILSQVSFWLNRESVPARTVFGVTTVLTMTTLSISAR
NSLPKVAYATAMDWFIAVCYAFVFSALIEFATVNYFTKRGYAWDGKSVVPEKPKKVKDPL
IKKNNTYAPTATSYTPNLARGDPGLATIAKSATIEPKEVKPETKPPEPKKTFNSVSKIDR
LSRIAFPLLFGIFNLVYWATYLNREPQLKAPTPHQ

Data Table 4. Amino acid sequence of the gamma 2 subunit of the rat GABA(A) receptor

>sp|P18508|GBRG2_RAT Gamma-aminobutyric acid receptor subunit gamma-2 OS=Rattus norvegicus GN=Gabrg2 PE=1 SV=1
MSSPNTWSTGSTVYSPVFSQKMTLWILLLLSLYPGFTSQKSDDDYEDYASNKTWVLTPKV
PEGDVTVILNNLLEGYDNKLRPDIGVKPTLIHTDMYVNSIGPVNAINMEYTIDIFFAQTW
YDRRLKFNSTIKVLRLNSNMVGKIWIPDTFFRNSKKADAHWITTPNRMLRIWNDGRVLYT
LRLTIDAECQLQLHNFPMDEHSCPLEFSSYGYPREEIVYQWKRSSVEVGDTRSWRLYQFS
FVGLRNTTEVVKTTSGDYVVMSVYFDLSRRMGYFTIQTYIPCTLIVVLSWVSFWINKDAV
PARTSLGITTVLTMTTLSTIARKSLPKVSYVTAMDLFVSVCFIFVFSALVEYGTLHYFVS
NRKPSKDKDKKKKNPAPTIDIRPRSATIQMNNATHLQERDEEYGYECLDGKDCASFFCCF
EDCRTGAWRHGRIHIRIAKMDSYARIFFPTAFCLFNLVYWVSYLYL

Data Table 5. Amino acid sequence of the alpha 1 subunit of the human GABA(A) receptor

>sp|P14867|GBRA1_HUMAN Gamma-aminobutyric acid receptor subunit alpha-1 OS=Homo sapiens GN=GABRA1 PE=1 SV=3

**MRKSPGLSDCLWAWILLLSTLTGRSYGQPSLQDELKDNTTVFTRILDRLLDGYDNRLRPG
LGERVTEVKTDIFVTSFGPVSDHDMEYTIDVFFRQSWKDERLKFKGPMTVLRLNNLMASK
IWTPDTFFHNGKKSVAHNMTMPNKLLRITEDGTLLYTMRLTVRAECPMHLEDFPMDAHAC
PLKFGSYAYTRAEVVYEWTREPARSVVVAEDGSRLNQYDLLGQTVDSGIVQSSTGEYVVM
TTHFHLKRKIGYFVIQTYLPCIMTVILSQVSFWLNRESVPARTVFGVTTVLTMTTLSISA
RNSLPKVAYATAMDWFIAVCYAFVFSALIEFATVNYFTKRGYAWDGKSVVPEKPKKVKDP
LIKKNNTYAPTATSYTPNLARGDPGLATIAKSATIEPKEVKPETKPPEPKKTFNSVSKID
RLSRIAFPLLFGIFNLVYWATYLNREPQLKAPTPHQ**

Data Table 6. Amino scid sequence of the gamma 2 subunit of the human GABA(A) receptor

>sp|P18507|GBRG2_HUMAN Gamma-aminobutyric acid receptor subunit gamma-2 OS=Homo sapiens GN=GABRG2 PE=1 SV=2

**MSSPNIWSTGSSVYSTPVFSQKMTVWILLLLSLYPGFTSQKSDDDYEDYASNKTWVLTPK
VPEGDVTVILNNLLEGYDNKLRPDIGVKPTLIHTDMYVNSIGPVNAINMEYTIDIFFAQT
WYDRRLKFNSTIKVLRLNSNMVGKIWIPDTFFRNSKKADAHWITTPNRMLRIWNDGRVLY
TLRLTIDAECQLQLHNFPMDEHSCPLEFSSYGYPREEIVYQWKRSSVEVGDTRSWRLYQF
SFVGLRNTTEVVKTTSGDYVVMSVYFDLSRRMGYFTIQTYIPCTLIVVLSWVSFWINKDA
VPARTSLGITTVLTMTTLSTIARKSLPKVSYVTAMDLFVSVCFIFVFSALVEYGTLHYFV
SNRKPSKDKDKKKKNPAPTIDIRPRSATIQMNNATHLQERDEEYGYECLDGKDCASFFCC
FEDCRTGAWRHGRIHIRIAKMDSYARIFFPTAFCLFNLVYWVSYLYL**

Data Table 7. Amino acid sequence of the Beta 2 Subunit of the Human GABA(A) Receptor

>sp|P47870|GBRB2_HUMAN Gamma-aminobutyric acid receptor subunit beta-2 OS=Homo sapiens GN=GABRB2 PE=1 SV=1

**MWRVRKRGYFGIWSFPLIIAAVCAQSVNDPSNMSLVKETVDRLLKGYDIRLRPDFGGPPV
AVGMNIDIASIDMVSEVNMDYTLTMYFQQAWRDKRLSYNVIPLNLTLDNRVADQLWVPDT
YFLNDKKSFVHGVTVKNRMIRLHPDGTVLYGLRITTTAACMMDLRRYPLDEQNCTLEIES
YGYTTDDIEFYWRGDDNAVTGVTKIELPQFSIVDYKLITKKVVFSTGSYPRLSLSFKLKR
NIGYFILQTYMPSILITILSWVSFWINYDASAARVALGITTVLTMTTINTHLRETLPKIP
YVKAIDMYLMGCFVFVFMALLEYALVNYIFFGRGPQRQKKAAEKAASANNEKMRLDVNKM
DPHENILLSTLEIKNEMATSEAVMGLGDPRSTMLAYDASSIQYRKAGLPRHSFGRNALER
HVAQKKSRLRRRASQLKITIPDLTDVNAIDRWSRIFFPVVFSFFNIVYWLYYVN**

Appendix II.

Data Table 8. Coordinates of the Pharmacophore

```
REMARK    File generated by Swiss-PdbViewer  4.00b0
REMARK    http://www.expasy.org/spdbv/
HETATM    1  *   hip    0      2.246   2.113   1.413  0.00100.00
HETATM    2  O   hip    0     -5.691  -0.267  -4.151  0.00100.00
HETATM    3  H1  hip    0      0.742   3.154  -3.474  0.00100.00
HETATM    4  H2  hip    0     -2.472  -1.062   0.681  0.00100.00
SPDBVT       1.0000000000       0.0000000000       0.0000000000
SPDBVT       0.0000000000       1.0000000000       0.0000000000
SPDBVT       0.0000000000       0.0000000000       1.0000000000
SPDBVT       0.0000000000       0.0000000000       0.0000000000
SPDBVT       0.0000000000       0.0000000000       0.0000000000
SPDBVV default;
SPDBVV    15.426431672954    1678.699418606842     20.000000000000
SPDBVV       0.4758893755       0.5102135228      -0.7163877885
SPDBVV       0.3084772896       0.6659613576       0.6792181034
```

SPDBVV 0.8236328455 -0.5442220423 0.1595346499
SPDBVV -1.7224999666 1.0460000038 -1.3689999580
SPDBVV 0.0000000000 0.0000000000 0.0000000000
SPDBVf 27
SPDBVl 1.00 1.00 1.00
SPDBVb 0.00 0.00 0.20
SPDBVg 64
SPDBVi 1 1 1 0 1 1 1 1 0 1 0 0 1 0 0
SPDBVp 0
END

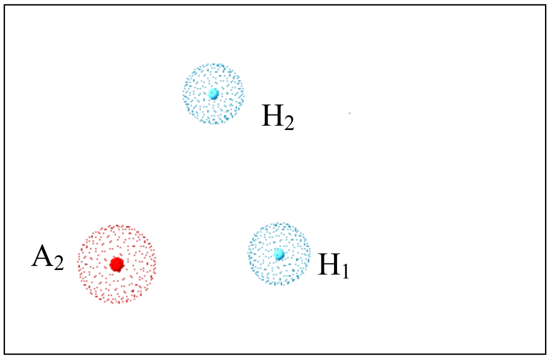

Appendix III.

Structures of compounds, as reported in Clayton.[21]

STRUCTURE	COOK CODE	a1	a2	a3	a4	a5	a6
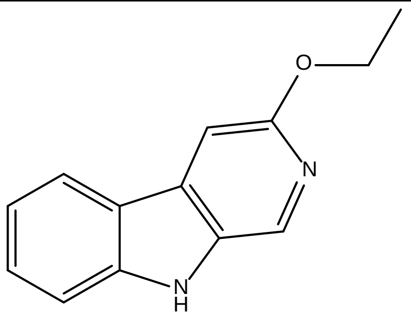 $C_{13}H_{12}N_2O$	3 EBC	6.43	25.1	28.2		826	1000
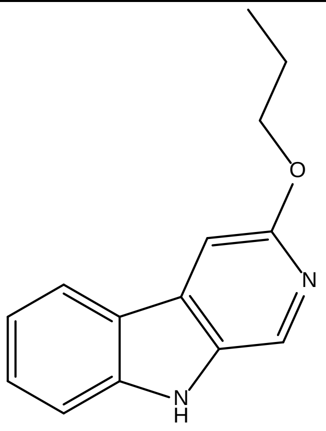 $C_{14}H_{14}N_2O$	3 PBC	5.3	52.3	68.8		591	1000
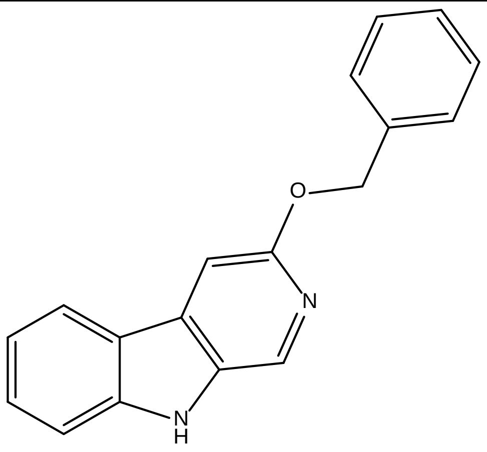 $C_{18}H_{14}N_2O$	3-BENZOXY bC	830	3000	3000		10000	10000
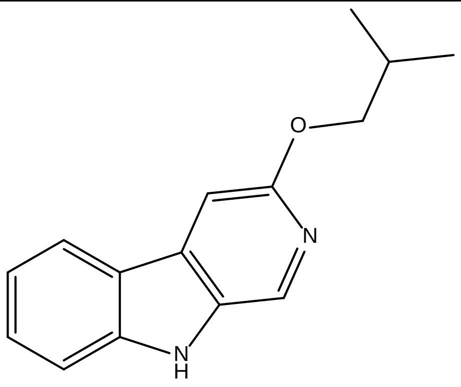 $C_{15}H_{16}N_2O$	3-isobutoxy bC	24.9	123.6	139.2		1000	10000
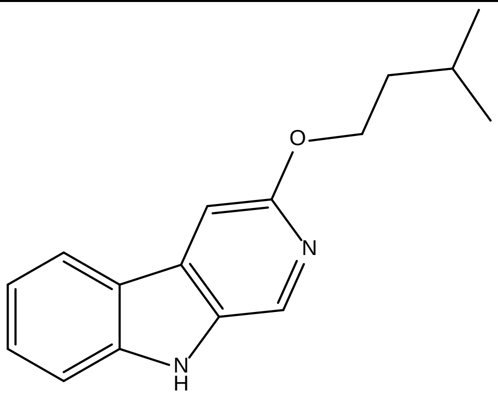 $C_{16}H_{18}N_2O$	3-isopentoxy bC	350.2	3000	3000		3000	10000
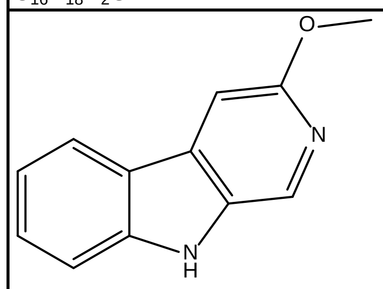 $C_{12}H_{10}N_2O$	3MBC						
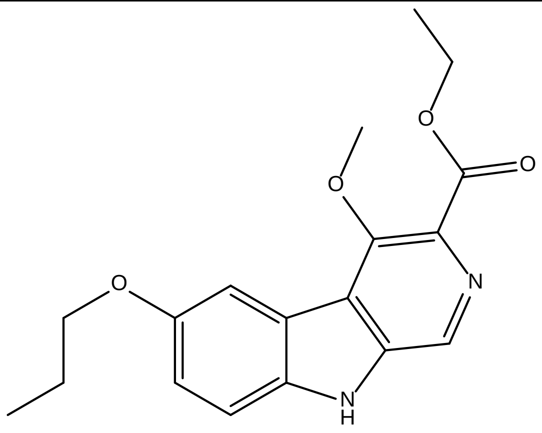 $C_{18}H_{20}N_2O_4$	6-PBC	0.49	1.21	2.2		2.39	1343

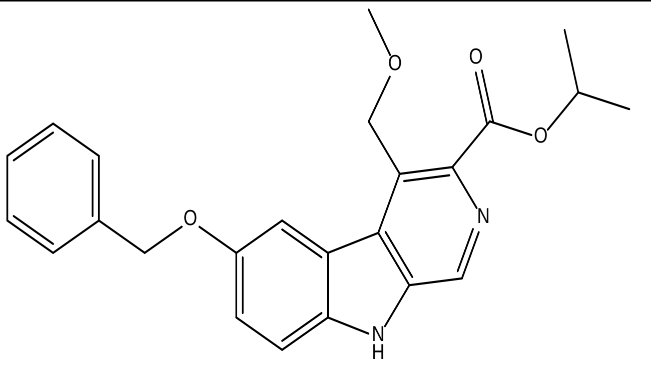 $C_{24}H_{24}N_2O_4$	ABECARNIL	12.4	15.3	7.5		6	1000
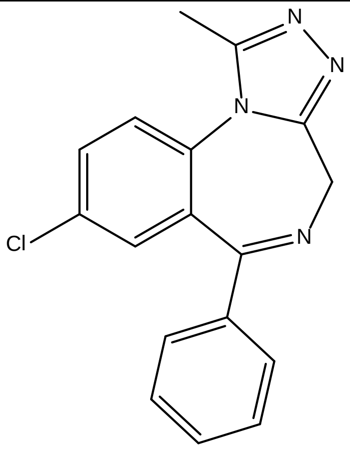 $C_{17}H_{13}ClN_4$	alprazolam	0.8	0.59	1.43		1.54	10000
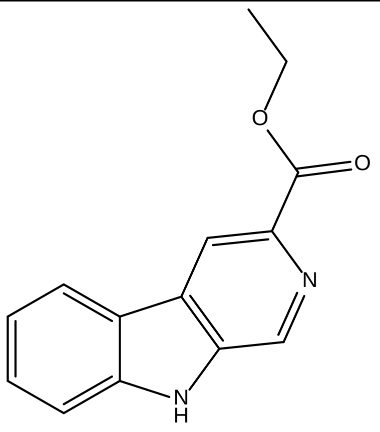 $C_{14}H_{12}N_2O_2$	BCCE	1.2	4.9	5.7		26.8	2700
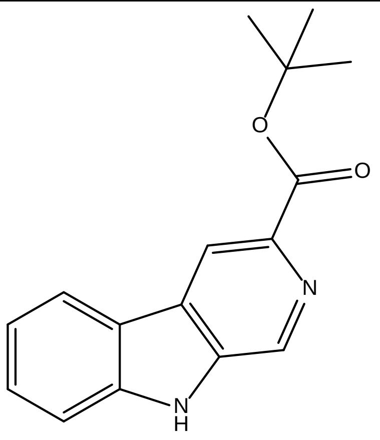 $C_{16}H_{16}N_2O_2$	BCCt	0.72	15	18.9		110.8	5000
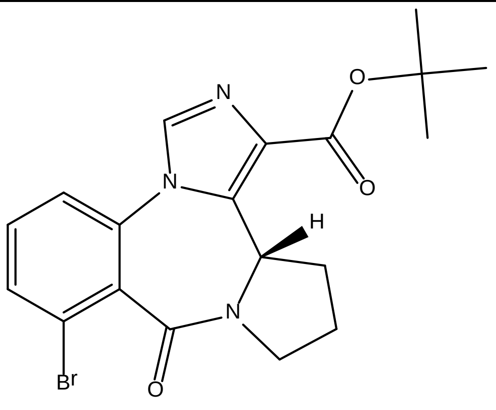 $C_{19}H_{20}BrN_3O_3$	BRETAZENIL	0.35	0.64	0.2		0.5	12.7
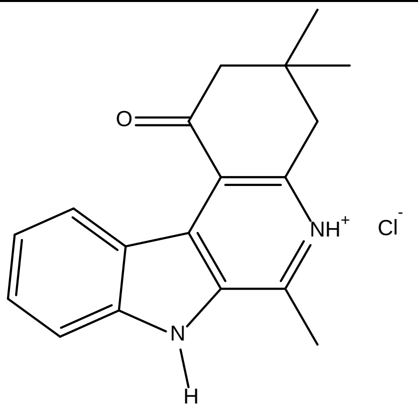 $C_{18}H_{19}ClN_2O$	C-383	1000		1000	1000	1000	1000
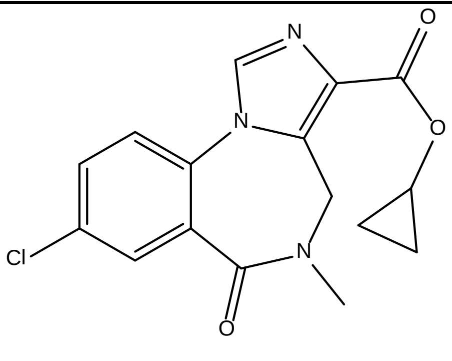 $C_{16}H_{14}ClN_3O_3$	CD-214	16.4	48.2	42.5		9.8	168

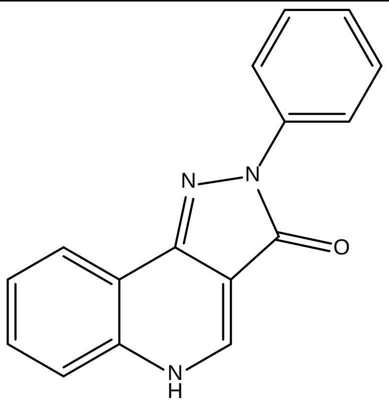 $C_{16}H_{11}N_3O$	CGS8216	0.13					46
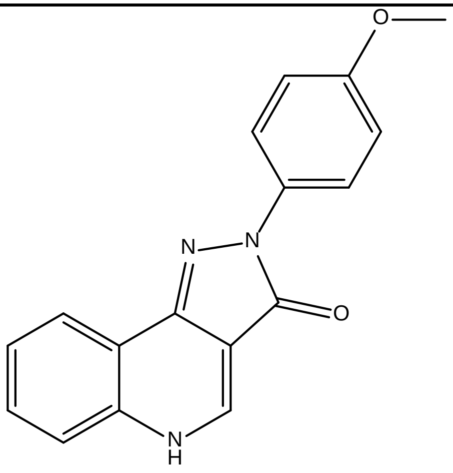 $C_{17}H_{13}N_3O_2$	CGS9895	0.21					9.3
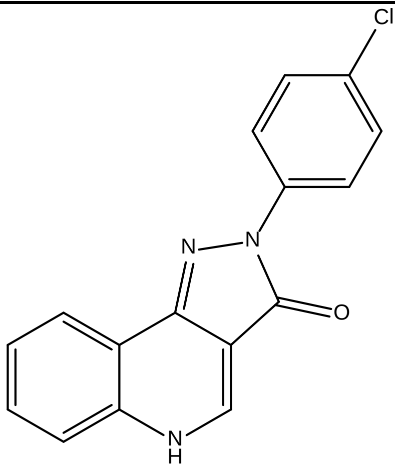 $C_{16}H_{10}ClN_3O$	CGS9896	0.28					181
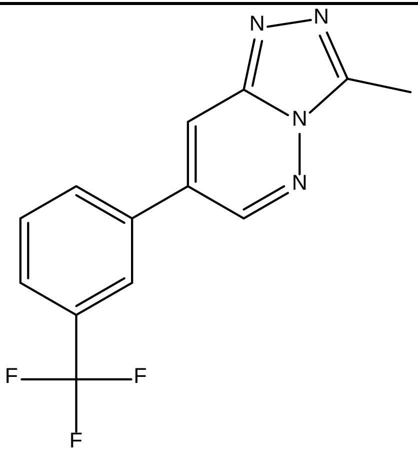 $C_{13}H_9F_3N_4$	CL 218,872	57	1964	1161		561	10000
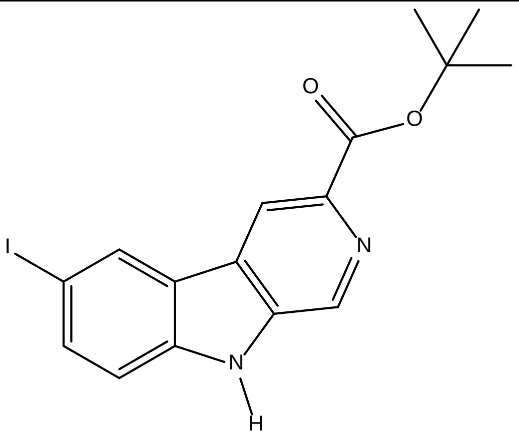 $C_{16}H_{15}IN_2O_2$	CM-A100	14.49	44.91	123.8	1000	65.31	1000
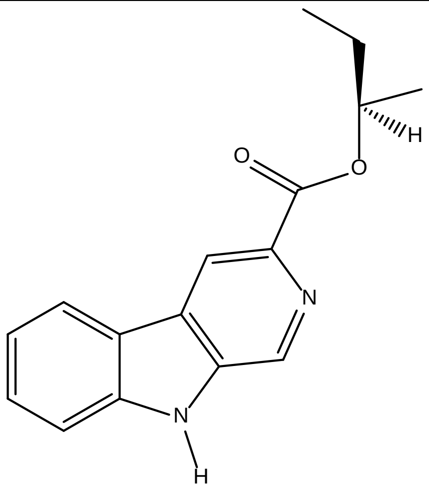 $C_{16}H_{16}N_2O_2$	CM-A49(R)	7.7	32.5	43		69	1000

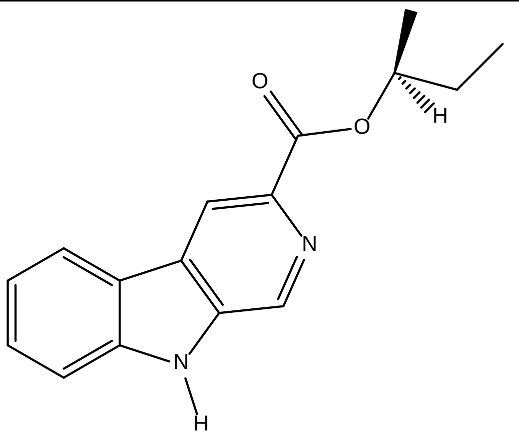 $C_{16}H_{16}N_2O_2$	CM-A50(S)	17	59	88		144	1000
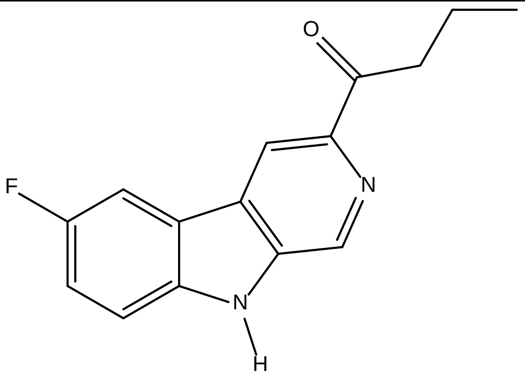 $C_{15}H_{13}FN_2O$	CM-A57	3.7	27	40		254	1000
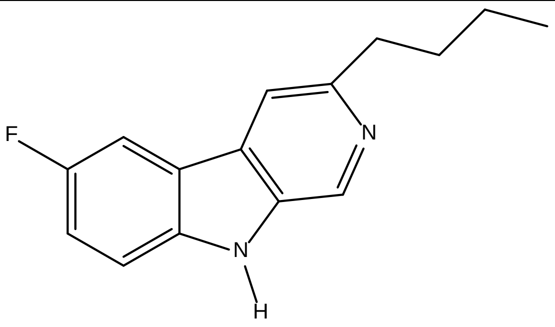 $C_{15}H_{15}FN_2$	CM-A58	16	120	184		1000	1012.2Nm
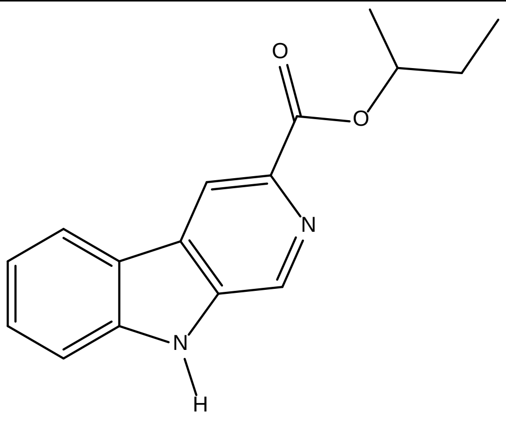 $C_{16}H_{16}N_2O_2$	CM-A64	18	60	116		216	1000
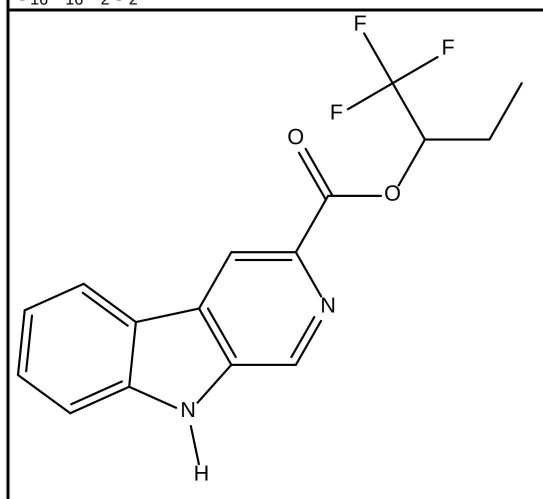 $C_{16}H_{13}F_3N_2O_2$	CM-A69	1000	1000	1000		1000	1000
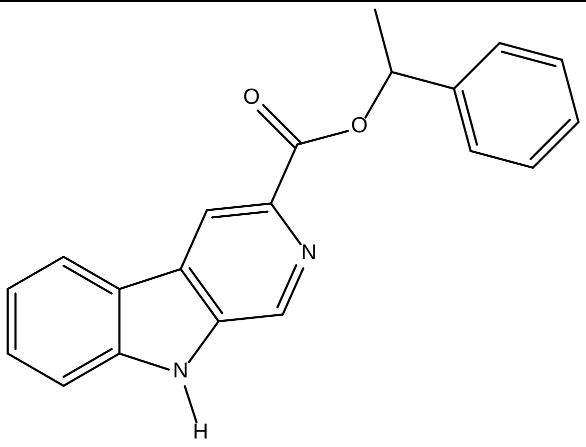 $C_{20}H_{16}N_2O_2$	CM-A71	333.57	1000	1000	1000	1000	3000

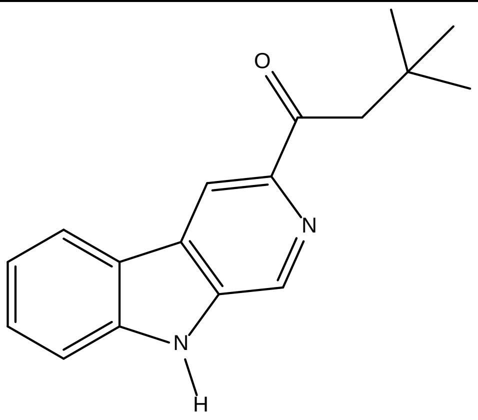 C17H18N2O	CM-A77	11.51	51.9	105.16	1000	42.62	1000
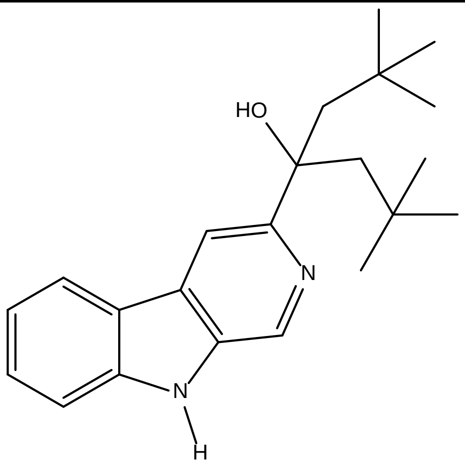 C22H30N2O	CM-A77a	27.4	111.5	301.3	1000	61.61	1000
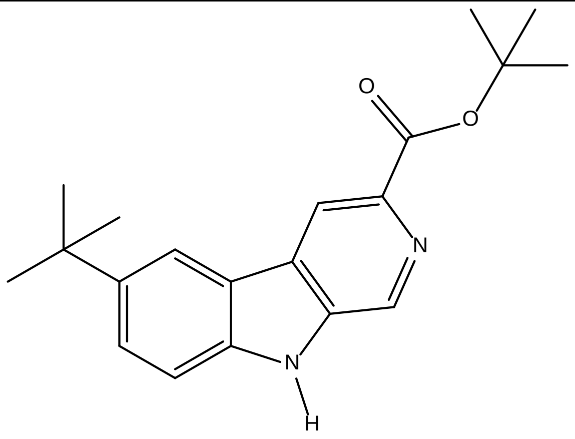 C20H24N2O2	CM-A82a	2.78	8.93	24.51	1000	7.49	1000
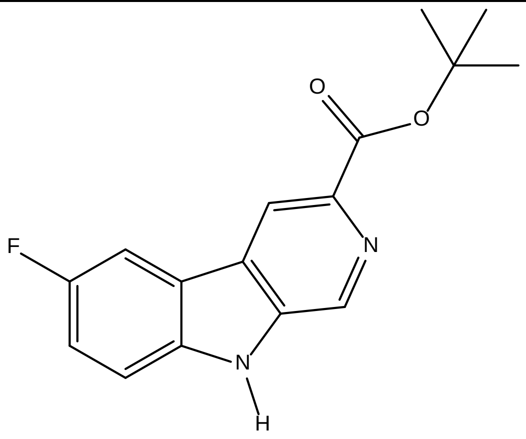 C16H15FN2O2	CM-A87	1.62	4.54	14.73	1000	4.61	1000
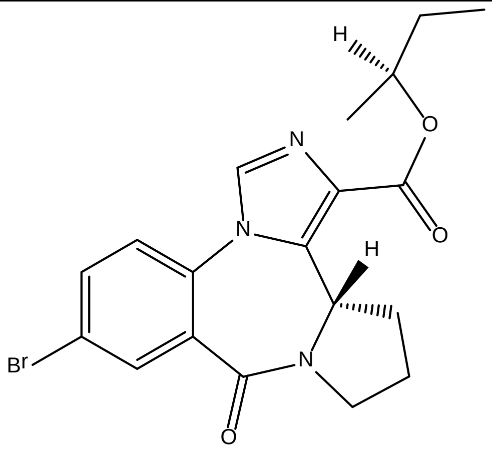 C19H20BrN3O3	CM-A95(ss)	141.5	225	59.45	30.36	146.3	107.1
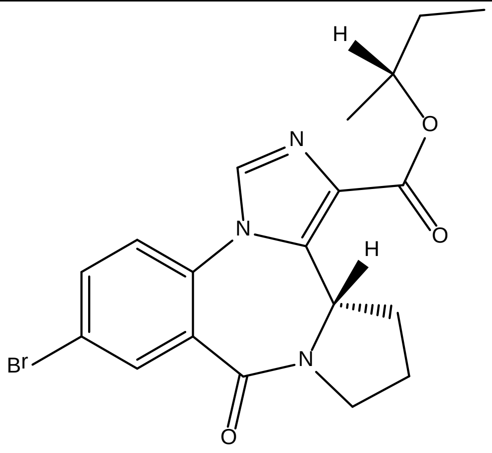 C19H20BrN3O3	CM-A97(sr)	70.81	89.48	30.64	40.73	67.01	38.12

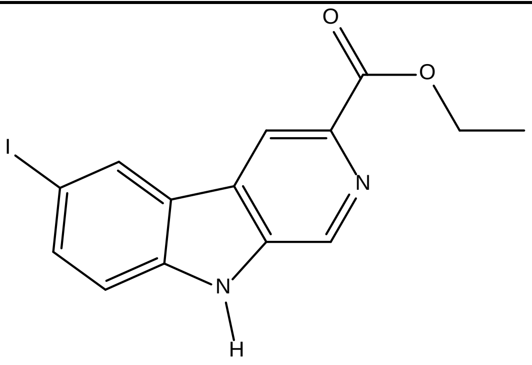<br>$C_{14}H_{11}IN_2O_2$	CM-B01	4.8	31	34	1000	286	1000
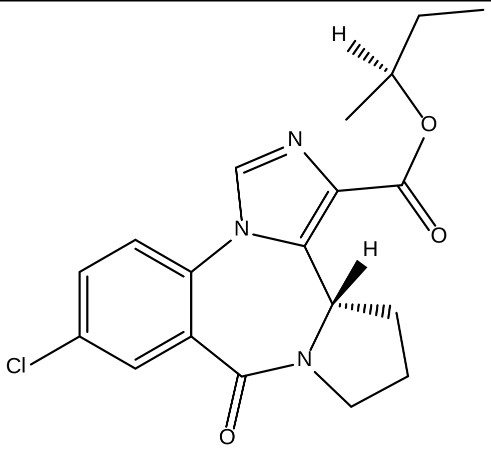<br>$C_{19}H_{20}ClN_3O_3$	CM-B31c(ss)	118	319	173	37	15	137
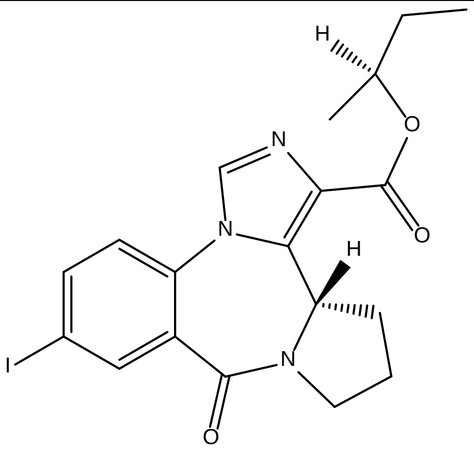<br>$C_{19}H_{20}IN_3O_3$	CM-B31i(ss)	90	184	78	18	4.9	121
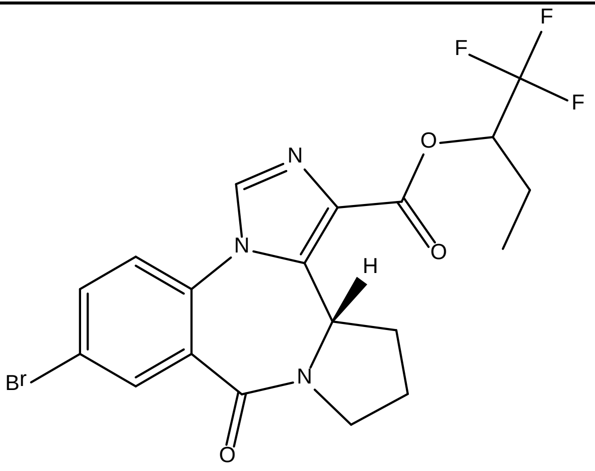<br>$C_{19}H_{17}BrF_3N_3O_3$	CM-B34	472	451	223	114	17	175
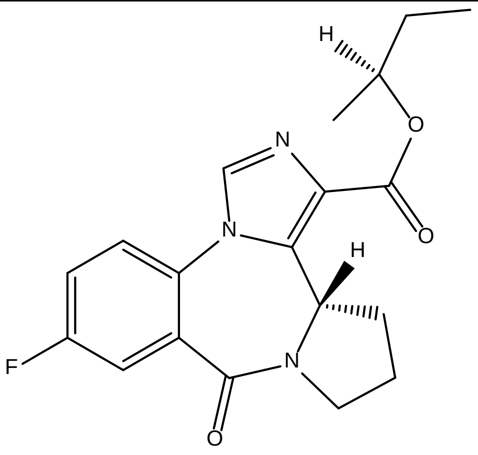<br>$C_{19}H_{20}FN_3O_3$	CM-B44(ss)	32	43	12	379	4.3	485
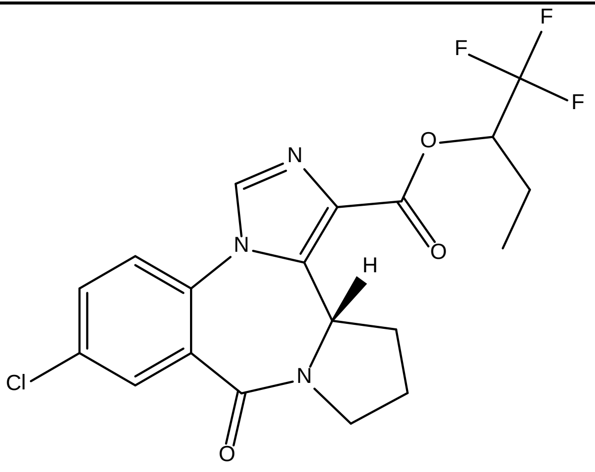<br>$C_{19}H_{17}ClF_3N_3O_3$	CM-B45	230	557	336	265	15	230

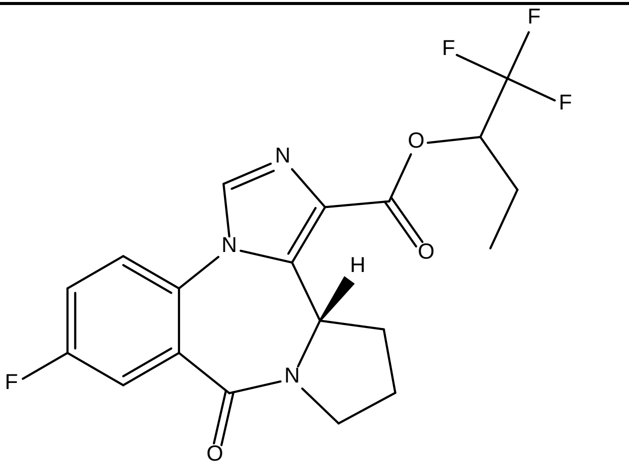 $C_{19}H_{17}F_4N_3O_3$	CM-B47	32	63	34	2007	4.4	717
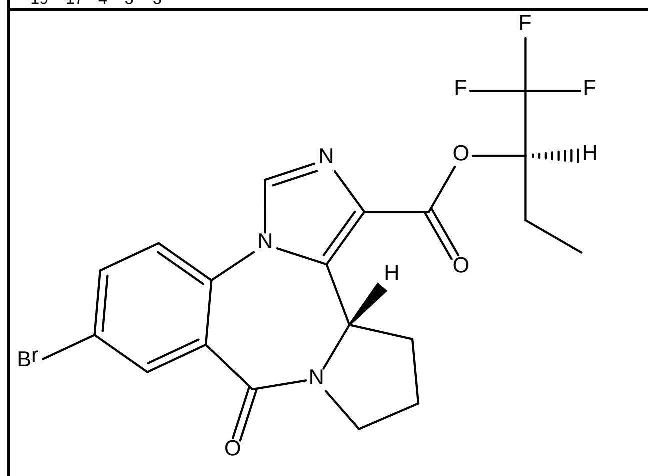 $C_{19}H_{17}BrF_3N_3O_3$	CM-C28(SR)	176	752	244	290	14	141
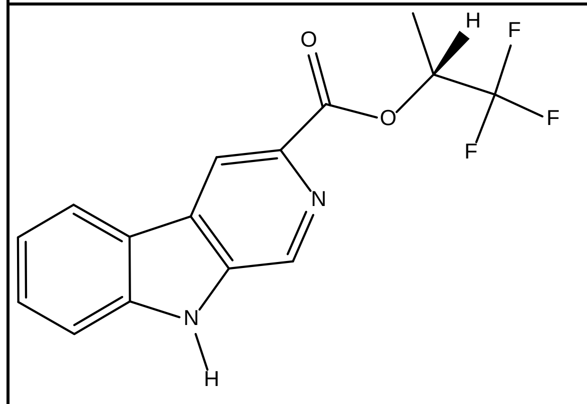 $C_{15}H_{11}F_3N_2O_2$	CM-D30(R)	27	343.3	453	3000	1847	2000
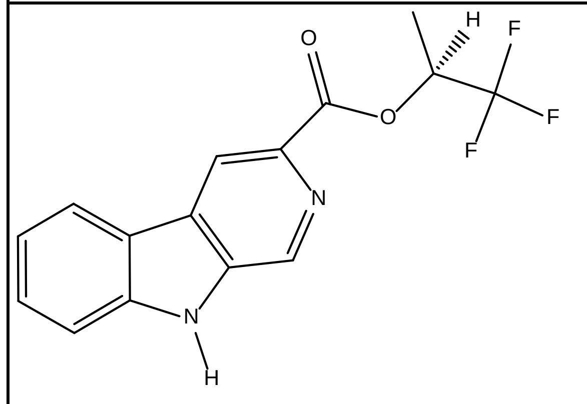 $C_{15}H_{11}F_3N_2O_2$	CM-D30(S)	90	931	172	3000	1000	3000
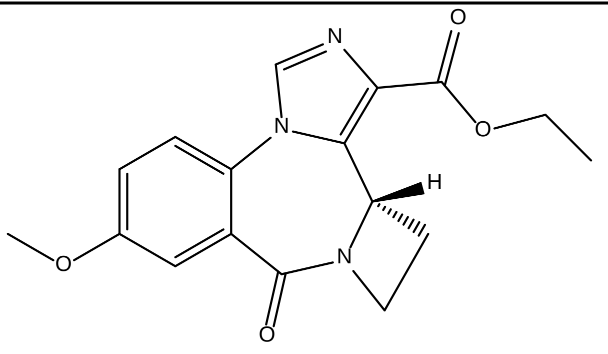 $C_{17}H_{17}N_3O_4$	CM-D44	34.3	56.3	20.7	0.33	0.57	0.92
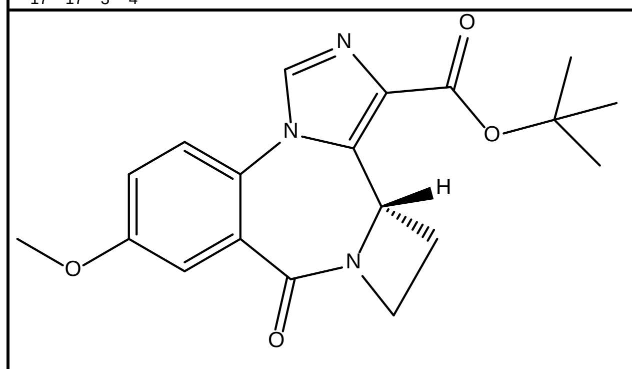 $C_{19}H_{21}N_3O_4$	CM-D45 C19H21N3O4	90.5	65.5	30.3	0.15	1.65	0.23
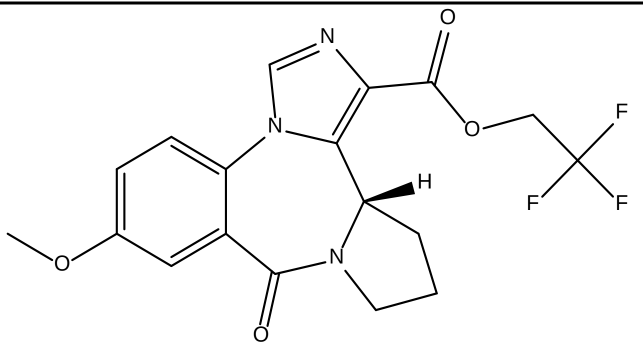 $C_{18}H_{16}F_3N_3O_4$	CM-E09a	176	192	122	490	9.2	718

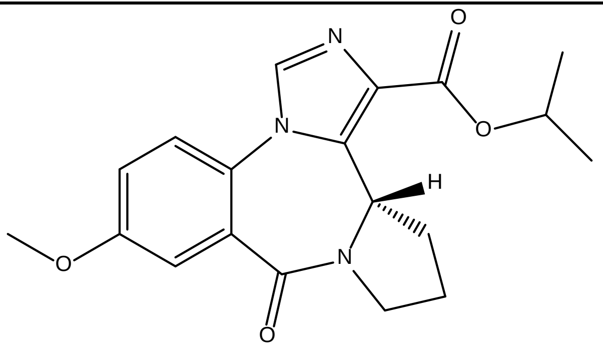 $C_{19}H_{21}N_3O_4$	CM-E09b	20	22	19	55	0.45	69
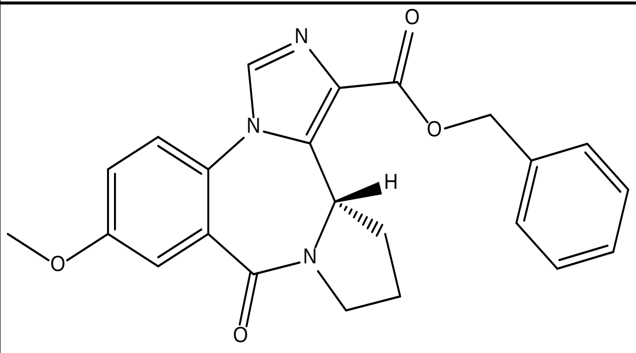 $C_{23}H_{21}N_3O_4$	CM-E10	23	26	14	215	0.51	96
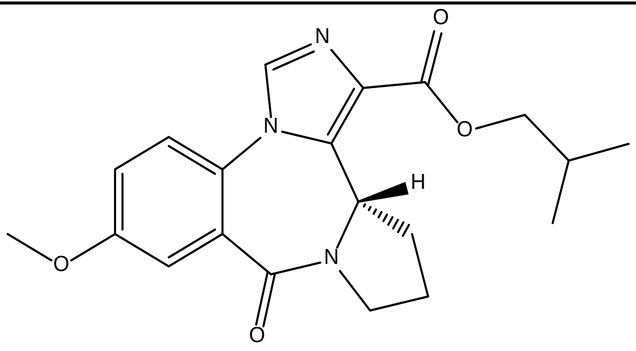 $C_{20}H_{23}N_3O_4$	CM-E11	333	308	161	394	14	750
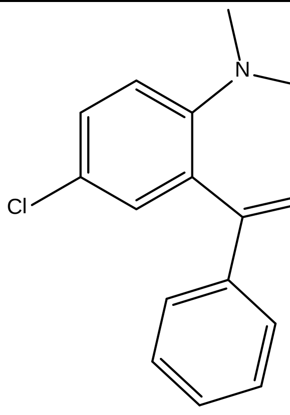 $C_{16}H_{13}ClN_2O$	diazepam	14	20	15		11	
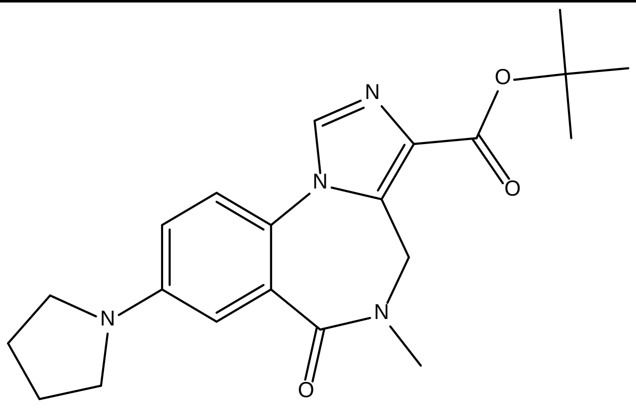 $C_{21}H_{26}N_4O_3$	DM-139	5.8		169		9.25	325
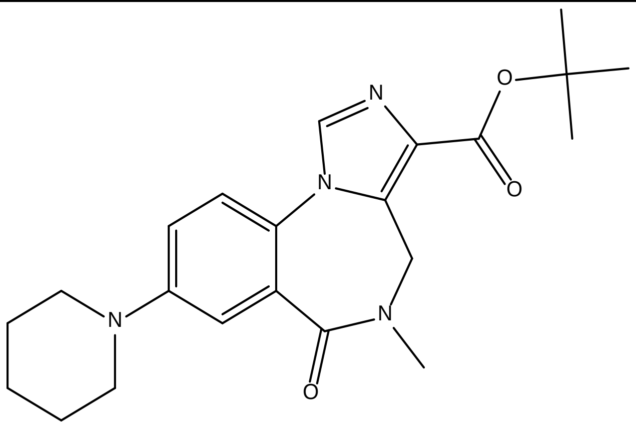 $C_{22}H_{28}N_4O_3$	DM-146	6.44		148		4.23	247
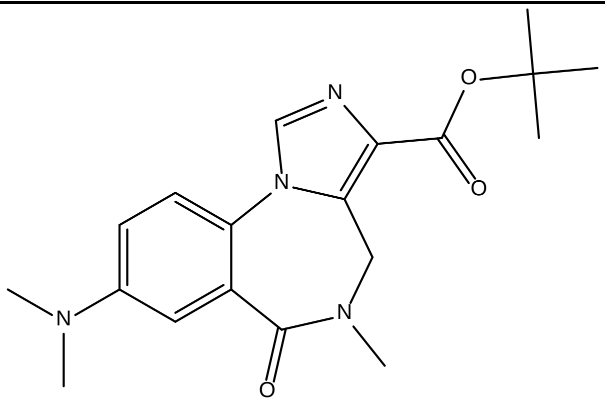 $C_{19}H_{24}N_4O_3$	DM-173	13.1		38.1		0.78	118

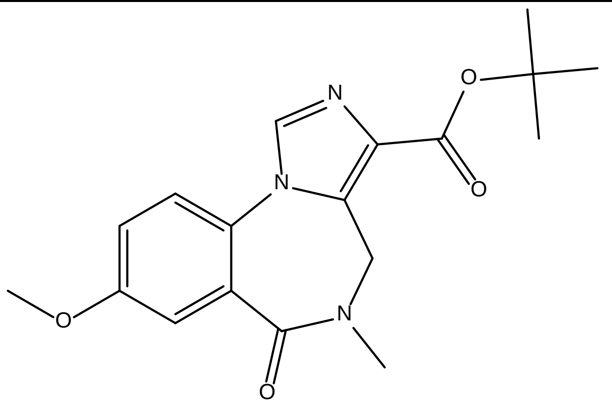 $C_{18}H_{21}N_3O_4$	DM-215	6.74		7.42		0.293	8.28
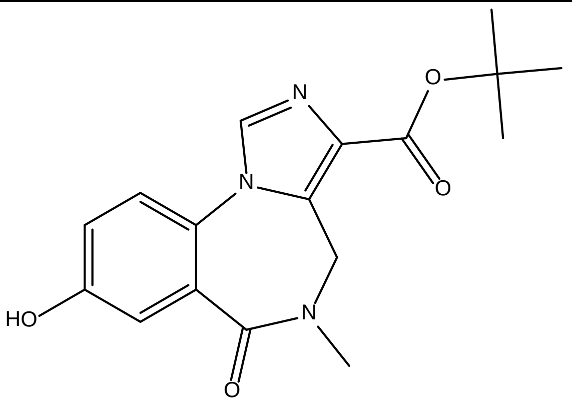 $C_{17}H_{19}N_3O_4$	DM-239	1.5		0.53		0.14	6.89
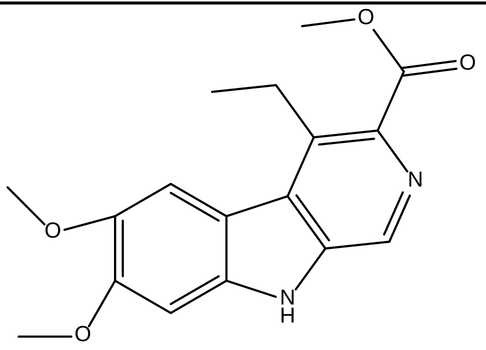 $C_{17}H_{18}N_2O_4$	DMCM	5.69	8.29	4		1.04	134
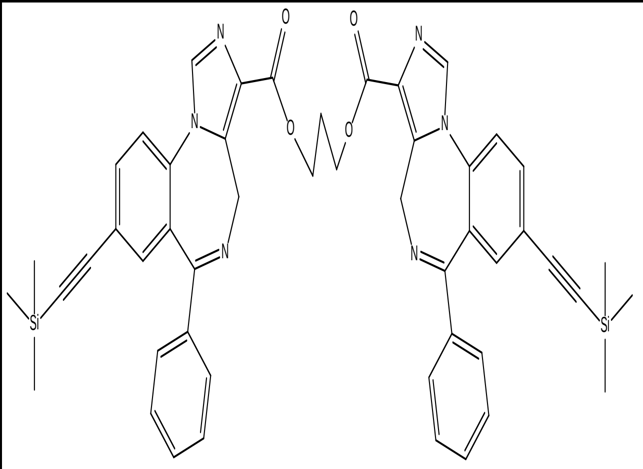 $C_{49}H_{46}N_6O_4Si_2$	DMH-D-048 (C49H46N6O4Si2)	5000	5000	5000		5000	5000
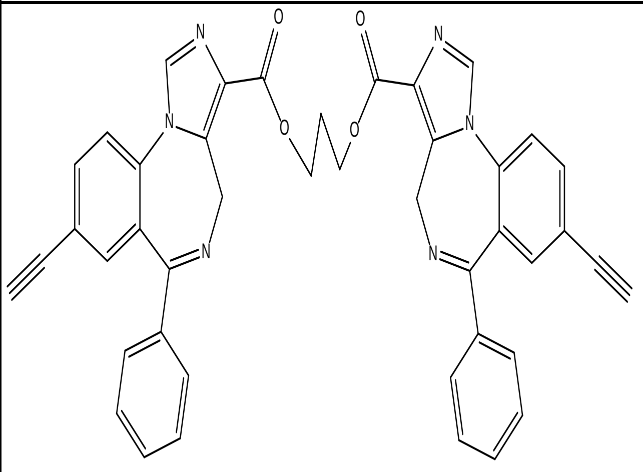 $C_{43}H_{30}N_6O_4$	DMH-D-053 (C43H30N6O4)	236	7.4	272	5000	194.2	5000
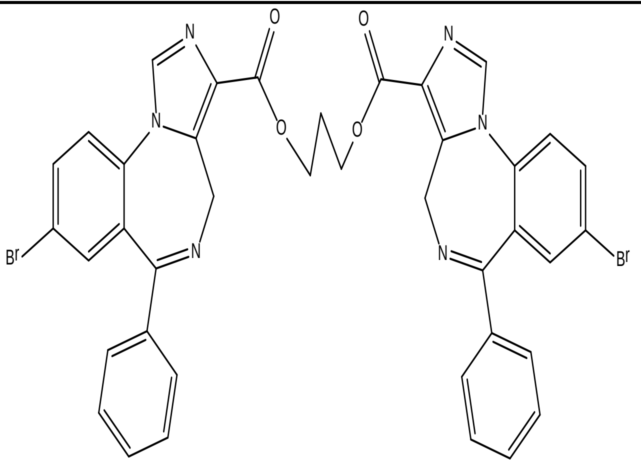 $C_{39}H_{28}Br_2N_6O_4$	DMH-D-070 (C39H28N6O4Br2)	5000	1901			5000	5000
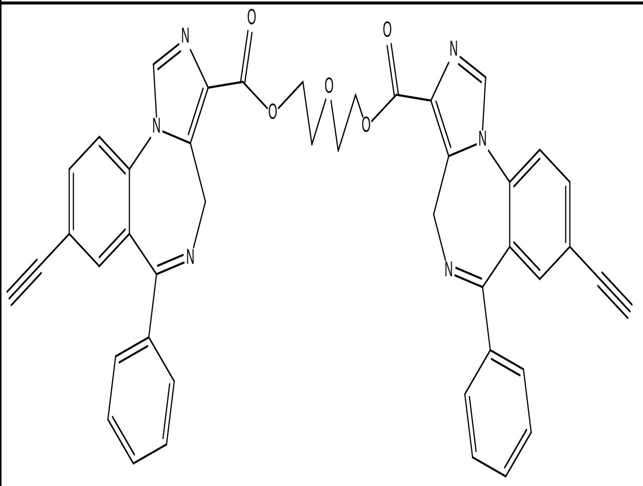 $C_{44}H_{32}N_6O_5$	DMH-III-96 (C44H32N6O5)	460	5000			5000	5000

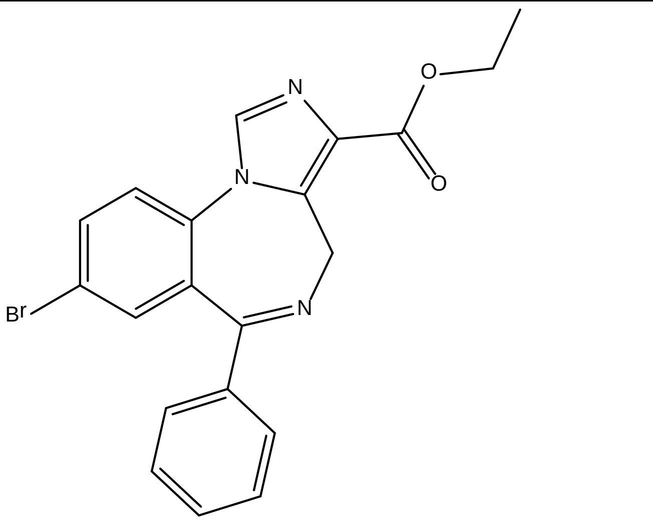 $C_{20}H_{16}BrN_3O_2$	DM-I-070						
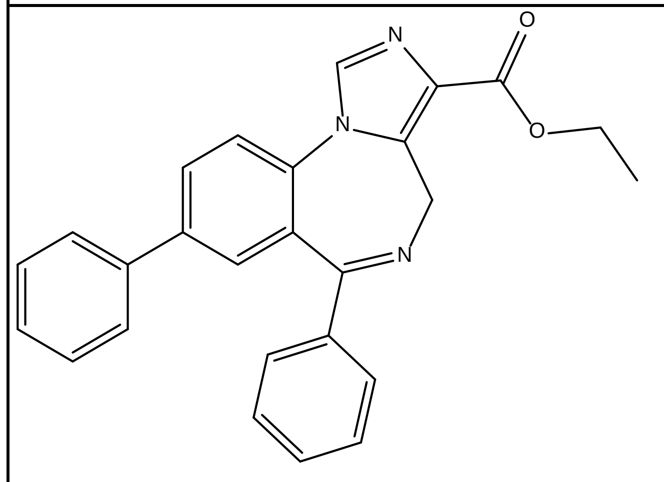 $C_{26}H_{21}N_3O_2$	DM-I-81	2000	2000	2000	2000	176	2000
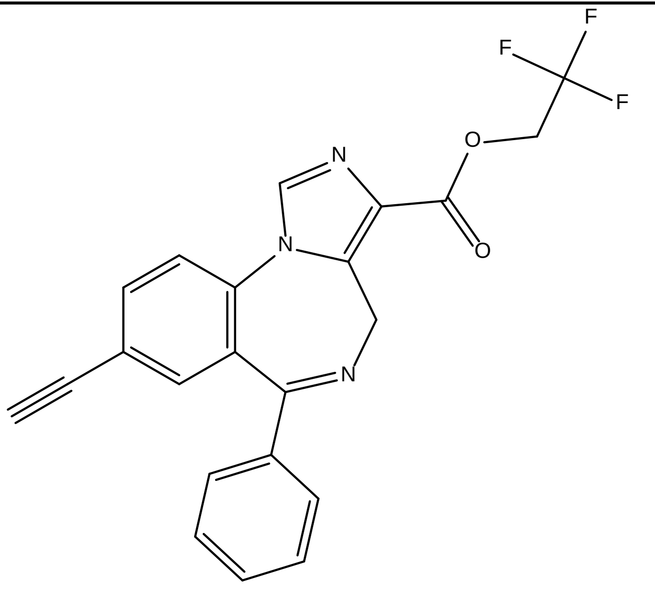 $C_{22}H_{14}F_3N_3O_2$	DM-II-20 (C22H14N3O2F3)	54.3	27.14	35.68		15.35	5000
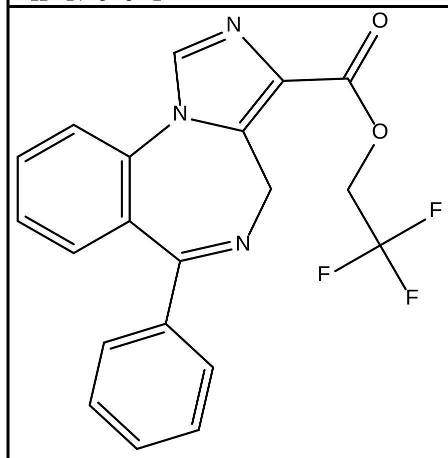 $C_{20}H_{14}F_3N_3O_2$	DM-II-30 C20H13N3O2BrF3)	17.6	13.4	28.51		7.8	5000
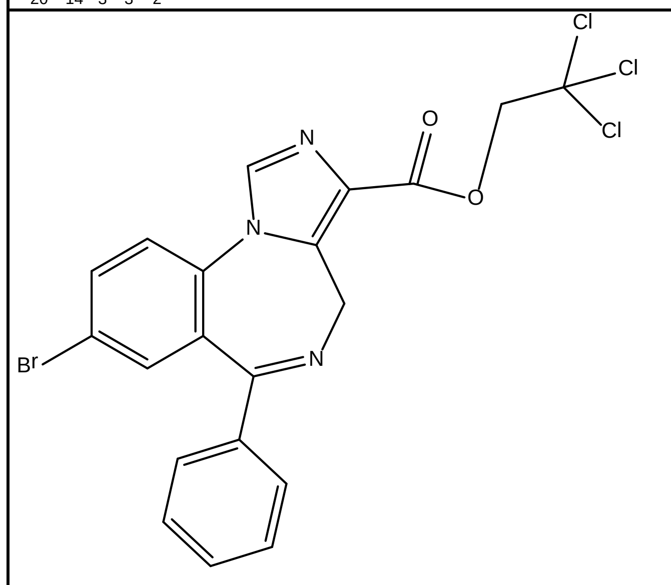 $C_{20}H_{13}BrCl_3N_3O_2$	DM-II-33 (C20H13N3O2BrCl3)	88.6	85	11.6		26.2	5000
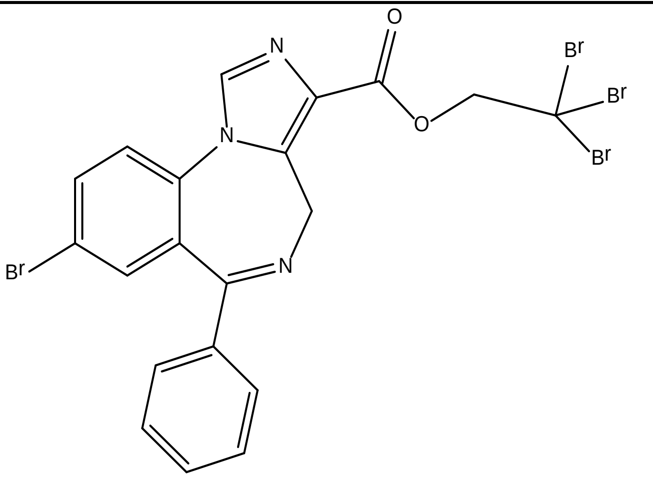 $C_{20}H_{13}Br_4N_3O_2$	DM-II-34						

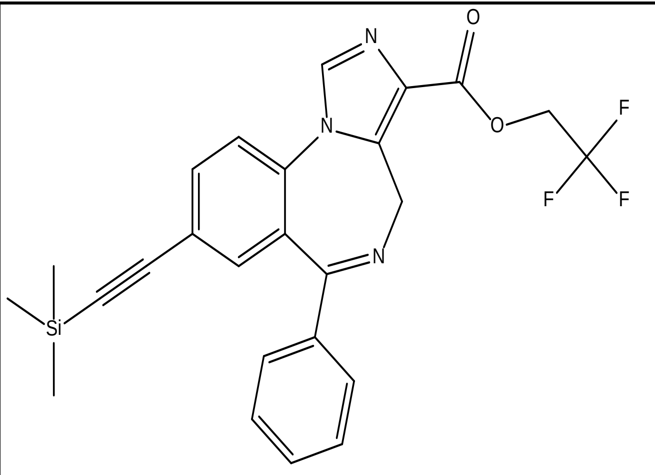 $C_{25}H_{22}F_3N_3O_2Si$	DM-II-35 (C25H22N3O2SiF3)	11380	327			380	5000
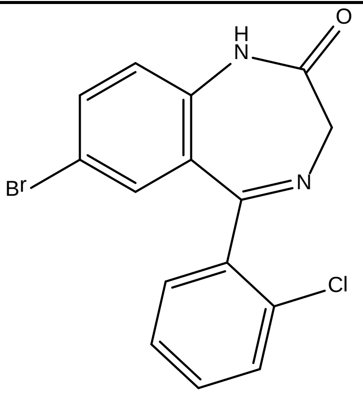 $C_{15}H_{10}BrClN_2O$	DM-II-72 (C15H10N20BrCl)	5000	1.37			2.02	5000
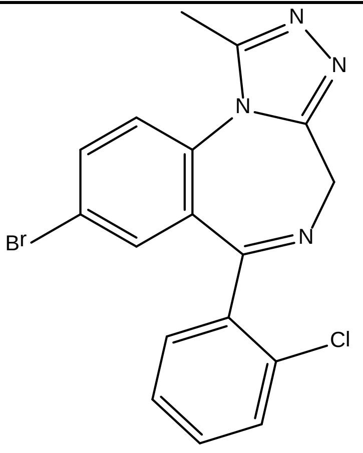 $C_{17}H_{12}BrClN_4$	DM-II-90 (C17H12N4BrCl)	0.505	1	0.63		0.37	5000
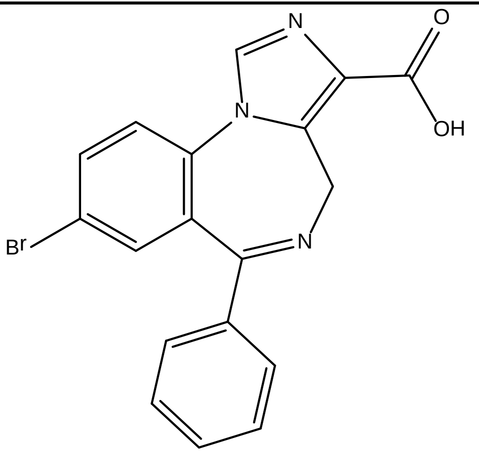 $C_{18}H_{12}BrN_3O_2$	DM-III-01 (C18H12N3O2Br)	5000	12			4.73	5000
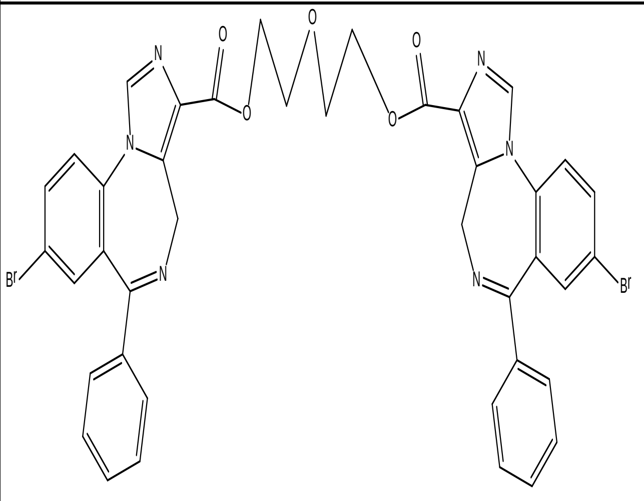 $C_{40}H_{30}Br_2N_6O_5$	DM-III-93 (C40H30N6O5Br2)	351	5000			179	5000
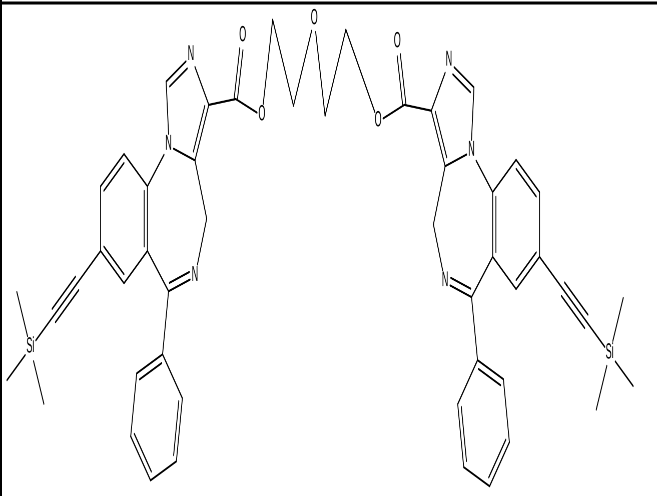 $C_{50}H_{48}N_6O_5Si_2$	DM-III-94 (C50H48N6O5Si2)	240	5000	5000		5000	5000

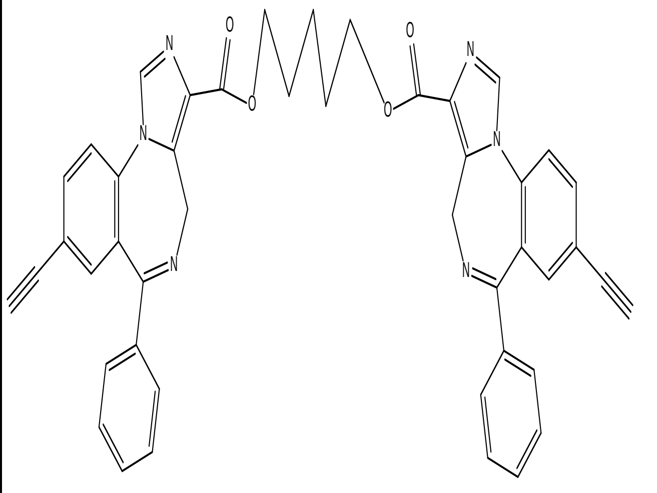 $C_{45}H_{34}N_6O_4$	DM-III-97 (C45H34N6O4)	280	5000			5000	5000
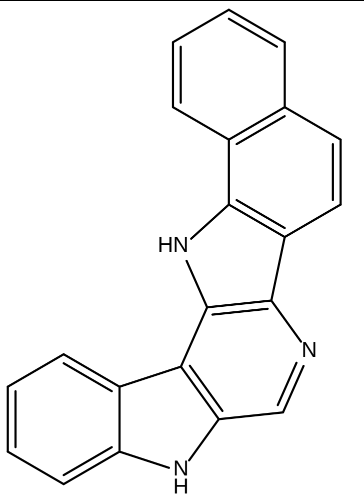 $C_{21}H_{13}N_3$	EDC-I	165		797		382.3	10000
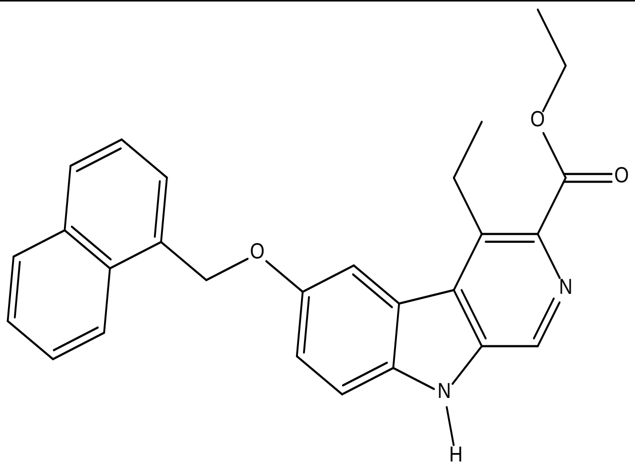 $C_{27}H_{24}N_2O_3$	EDC-I-042	183	20.5	300		270	10000
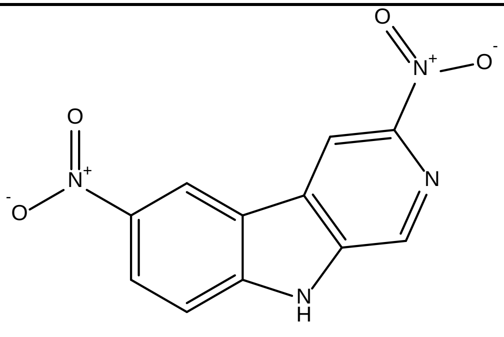 $C_{11}H_6N_4O_4$	EDC-I-071	12.9	83.1			314	5000
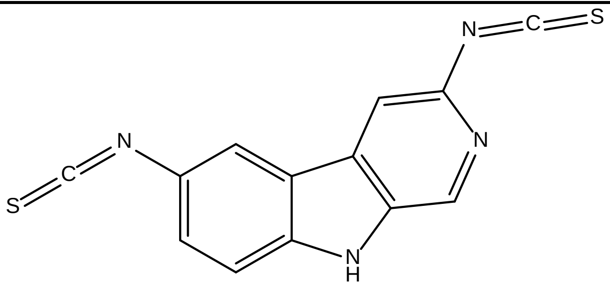 $C_{13}H_6N_4S_2$	EDC-I-093	13.6	423			2912	5000
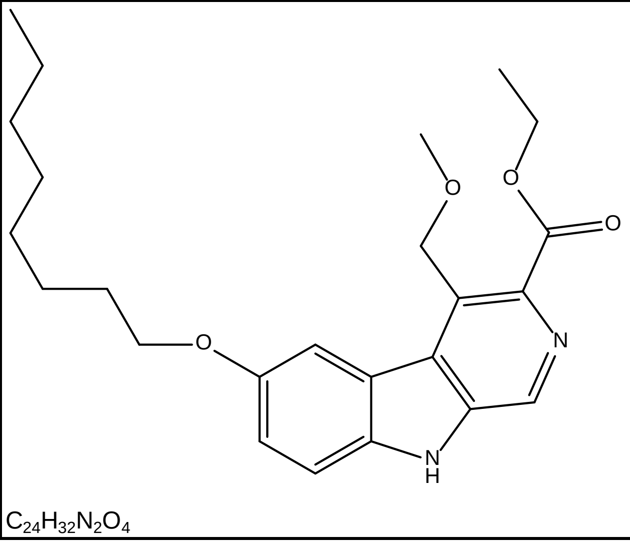 $C_{24}H_{32}N_2O_4$	EDC-II-012	1000	3000	3000		3000	3000

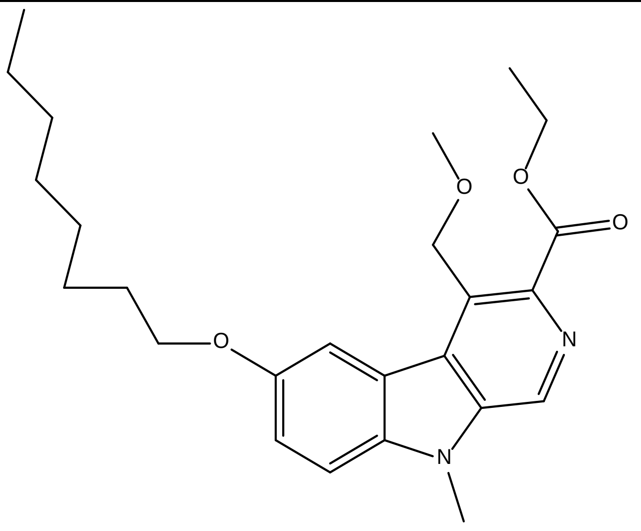 $C_{25}H_{34}N_2O_4$	EDC-II-015	3000	3000	3000		3000	3000
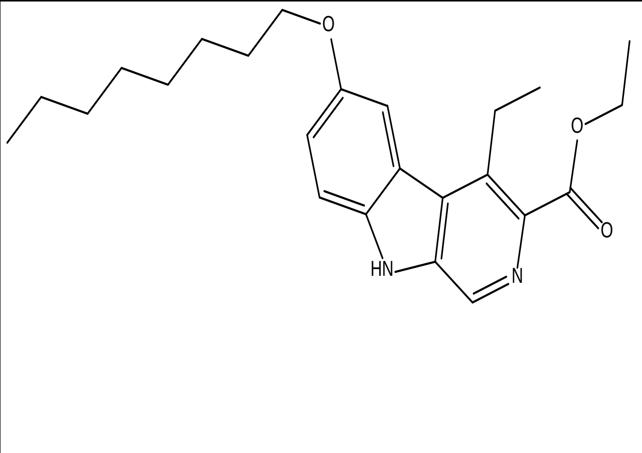 $C_{24}H_{32}N_2O_3$	EDC-II-024	3647	29749	35060		12683	30000
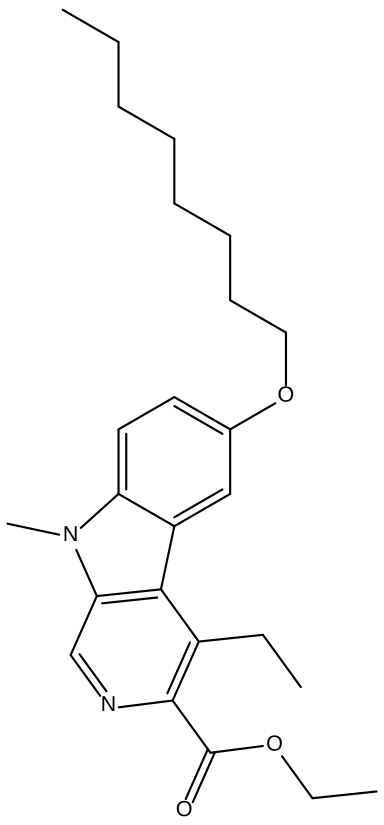 $C_{25}H_{34}N_2O_3$	EDC-II-031	1000	1000	1000		1000	1000
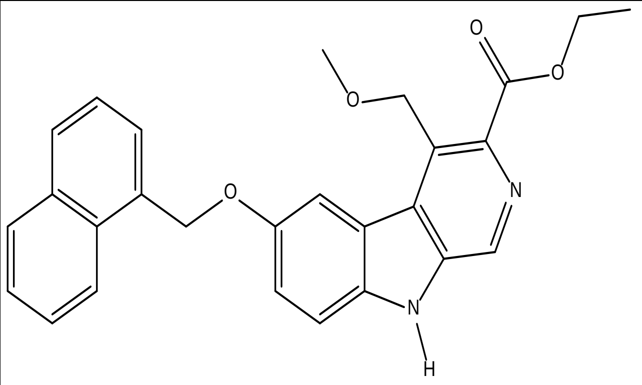 $C_{27}H_{24}N_2O_4$	EDC-II-044	15.4		293		323	1000
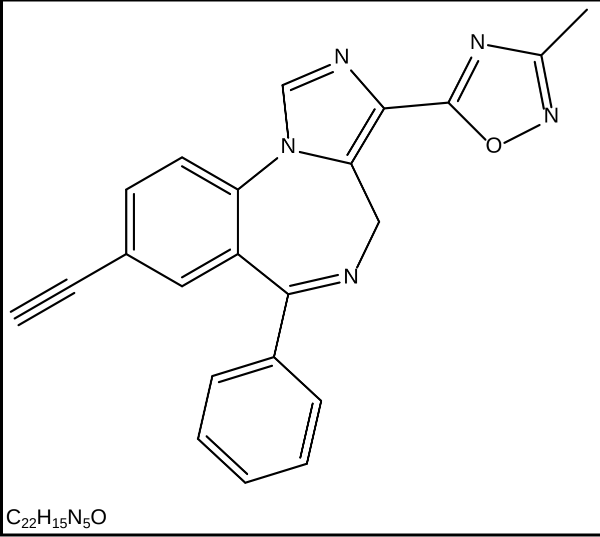 $C_{22}H_{15}N_5O$	EMJ-I-025	288	474	316		86	5000

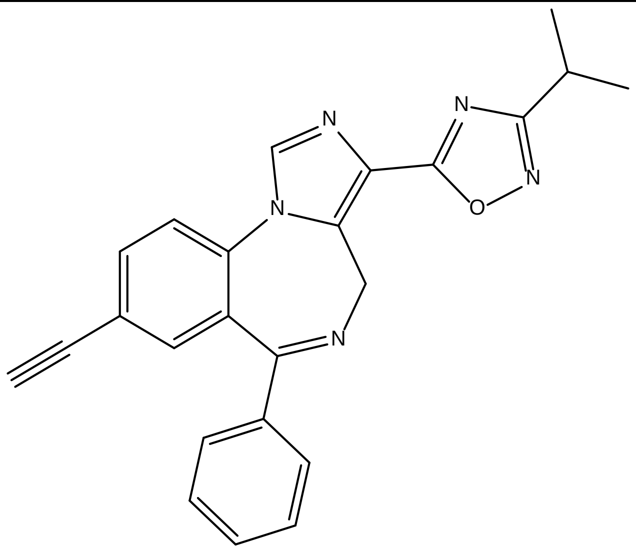 $C_{24}H_{19}N_5O$	EMJ-I-026	5000	135	1027		152	5000
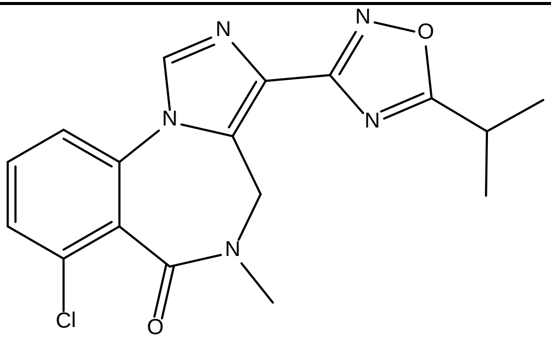 $C_{17}H_{16}ClN_5O_2$	FG8205	0.4	2.08	1.16		1.54	227
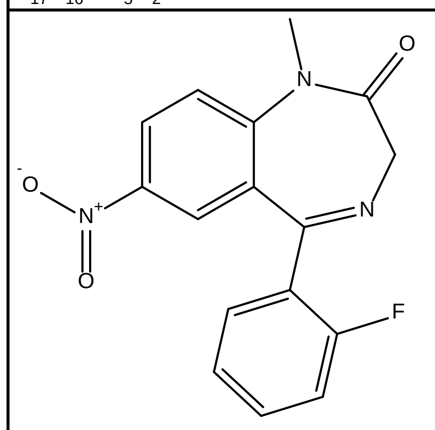 $C_{16}H_{12}FN_3O_3$	FLUNITRAZEPAM	2.2	2.5	4.5		2.1	2000
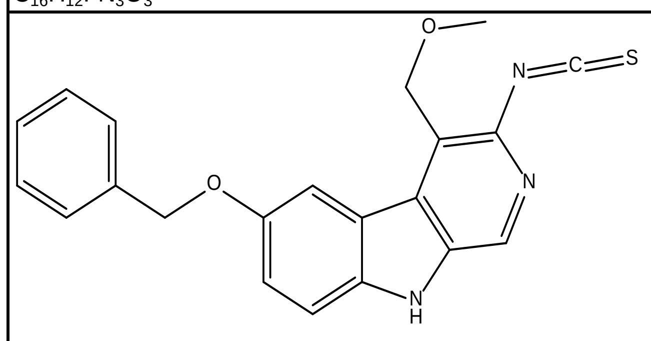 $C_{21}H_{17}N_3O_2S$	HDA-343	25	82.6	58.8		159	10000
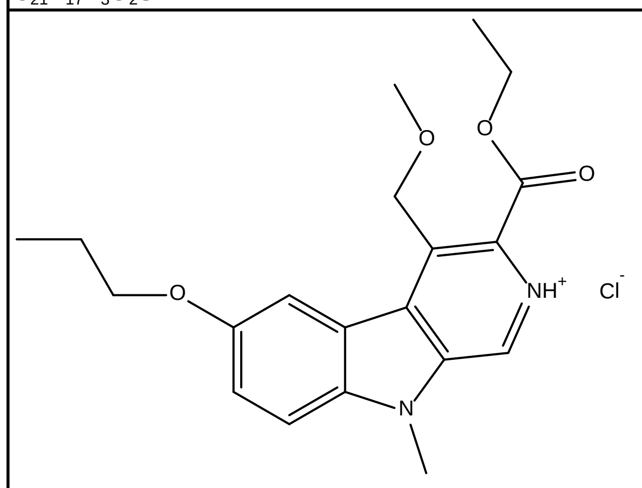 $C_{20}H_{25}ClN_2O_4$	HDA-90-III	25.6	105.4	163.6		160.6	10000
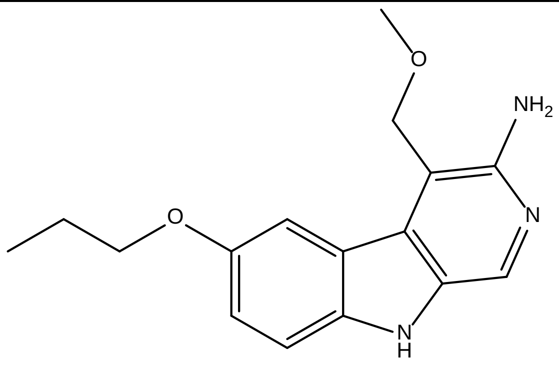 $C_{16}H_{19}N_3O_2$	HDA-II-96	236	3000	3000		3000	10000

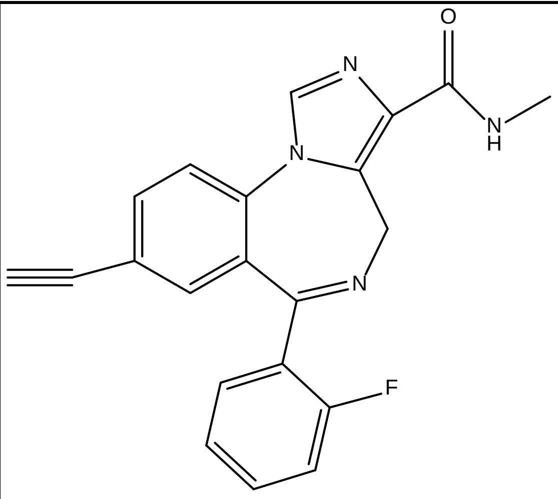 $C_{21}H_{15}FN_4O$	HJ-I-037	15.07	8.127	28.29		0.818	
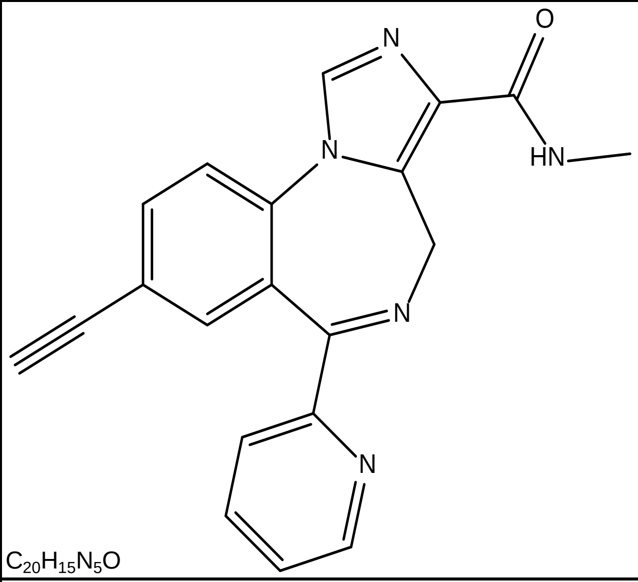 $C_{20}H_{15}N_5O$	HJ-I-040	1070	869.4	1123.6		1065	
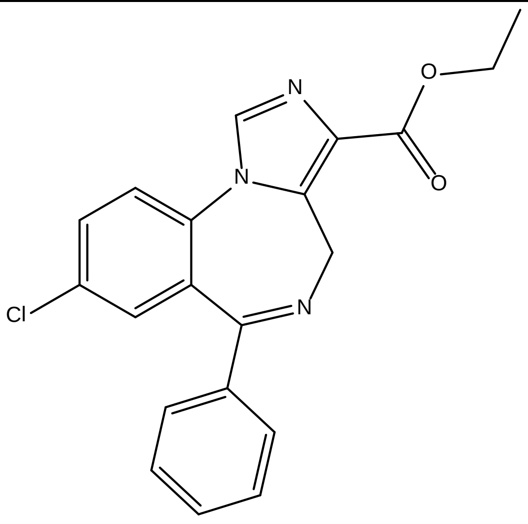 $C_{20}H_{16}ClN_3O_2$	Hz111 C20H16N3ClO2	46.65	34	77.6		24.6	842
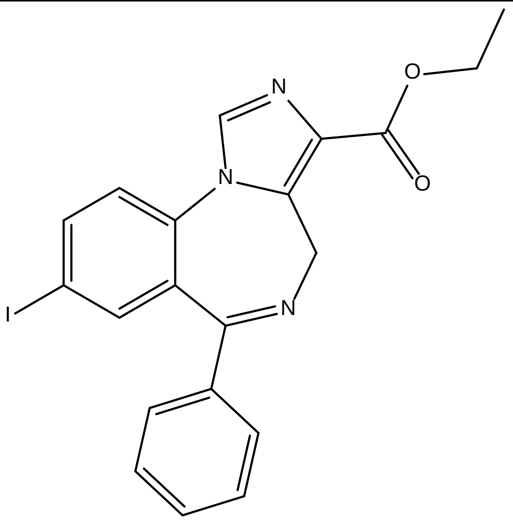 $C_{20}H_{16}IN_3O_2$	Hz120 ANX2	5000	35	78.5		20.6	32.2
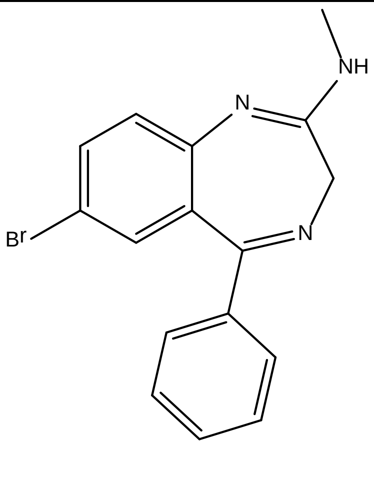 $C_{16}H_{14}BrN_3$	Hz135 C16H14N3Br	94.03	182			97	5000
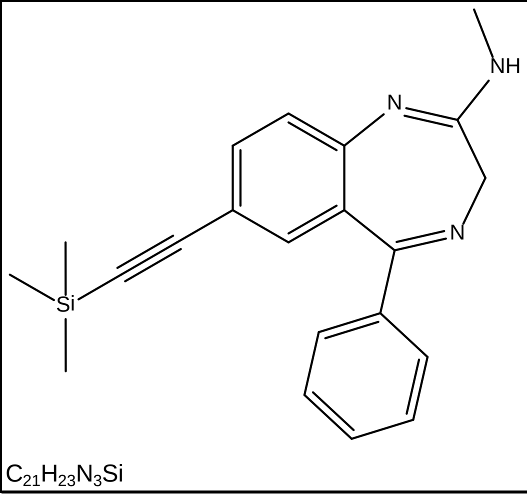 $C_{21}H_{23}N_3Si$	Hz141 C21H23N3Si	6480	5000	5000		737	5000

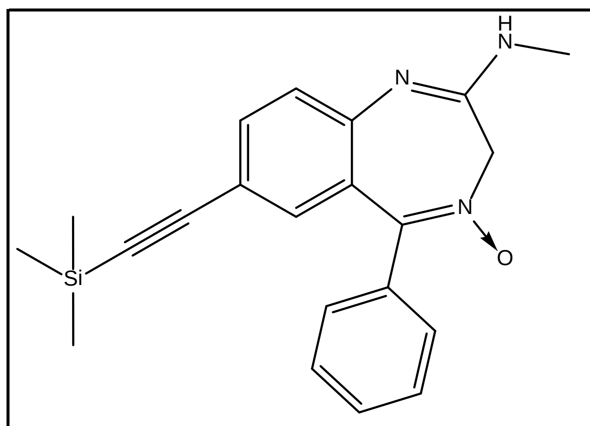 $C_{21}H_{23}N_3OSi$	Hz146 C21H23N3OSi	5000	5000			5000	5000
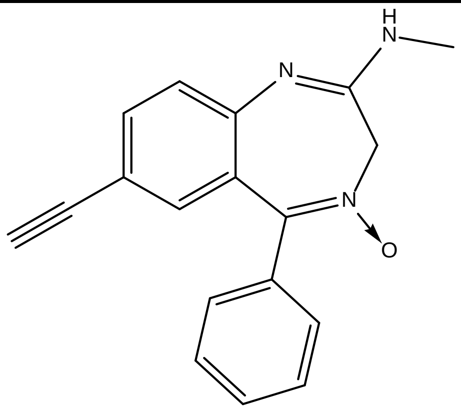 $C_{18}H_{15}N_3O$	Hz147 C18H15N3O	5000	5000			5000	5000
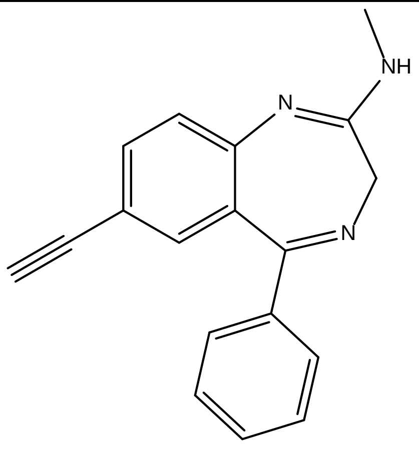 $C_{18}H_{15}N_3$	Hz148 C18H15N3	10.98	5000			256	5000
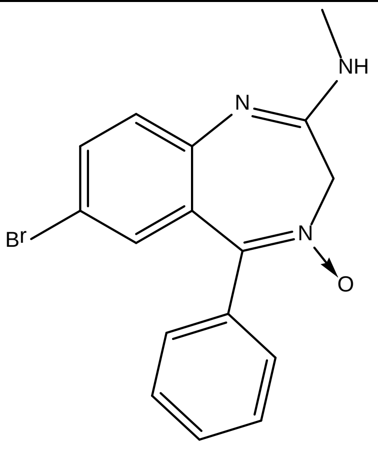 $C_{16}H_{14}BrN_3O$	Hz150 C16H14N3OBr	485	469	5000		419	5000
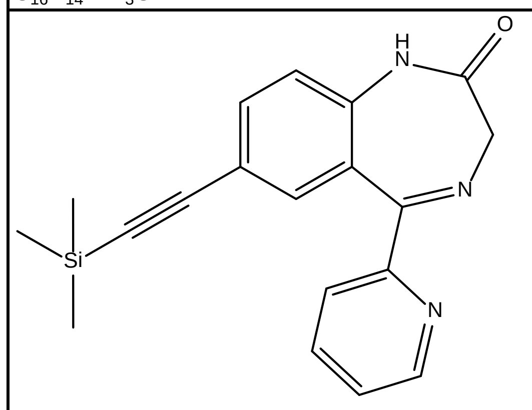 $C_{19}H_{19}N_3OSi$	Hz157 C19H19N3OSi	5000	5000	5000		5000	5000
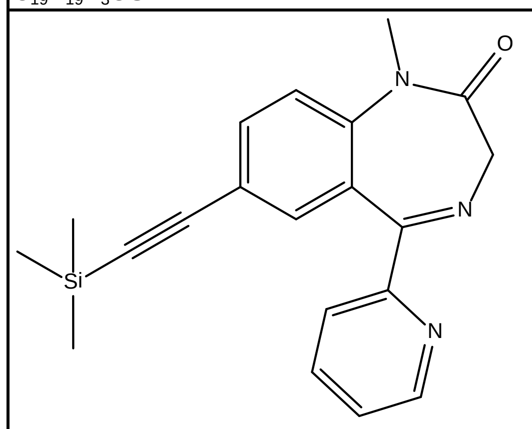 $C_{20}H_{21}N_3OSi$	Hz158 C20H21N3OSi	5000	5000	5000		5000	5000

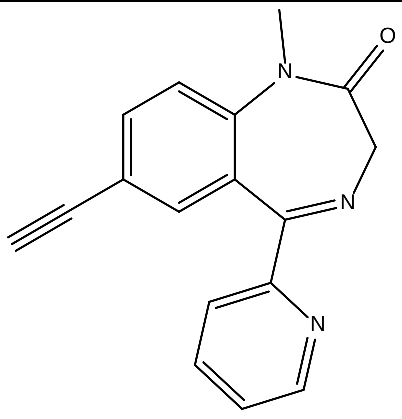 $C_{17}H_{13}N_3O$	Hz160 C17H13N3O	492	2633	334		87.3	5000
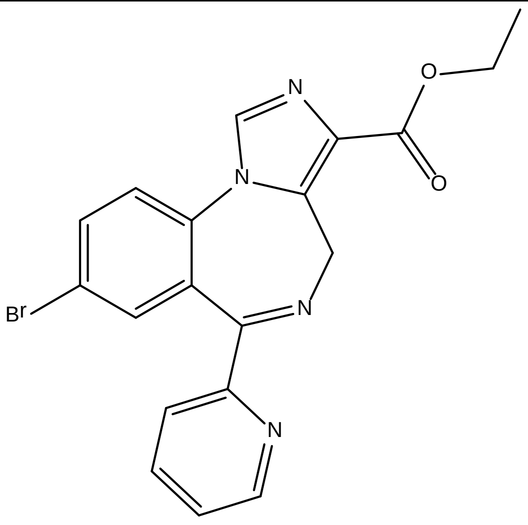 $C_{19}H_{15}BrN_4O_2$	Hz164 C19H15N4O2Br	99.18	150	459		108.2	494
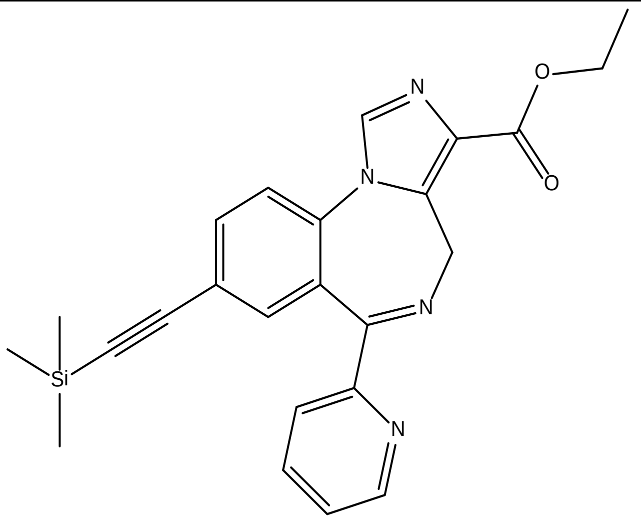 $C_{24}H_{24}N_4O_2Si$	Hz165 C24H24N4O2Si	5000	5000			5000	5000
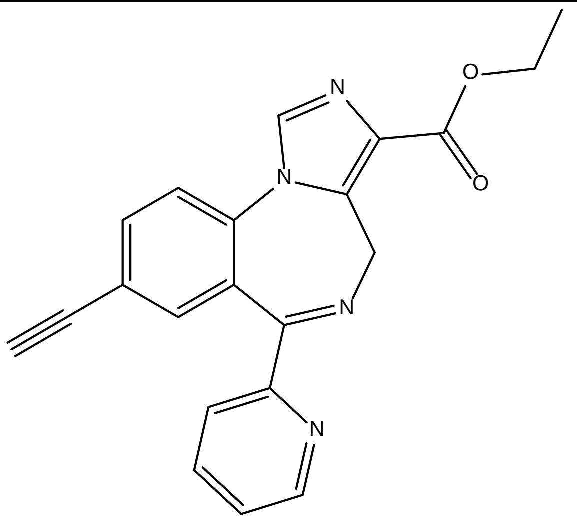 $C_{21}H_{16}N_4O_2$	Hz166 C21H16N4O2	118	148	365	5000	76.88	5000
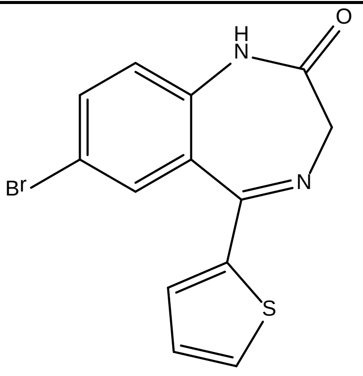 $C_{13}H_9BrN_2OS$	JC184 C13H9BrN2OS	9.606	10.5			6.709	
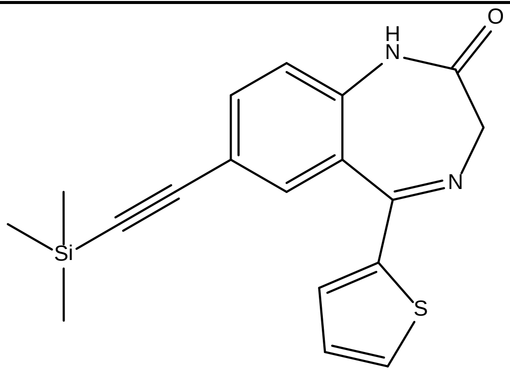 $C_{18}H_{18}N_2OSSi$	JC207 C18H18N2OSSi	104.8	169.7			68.16	
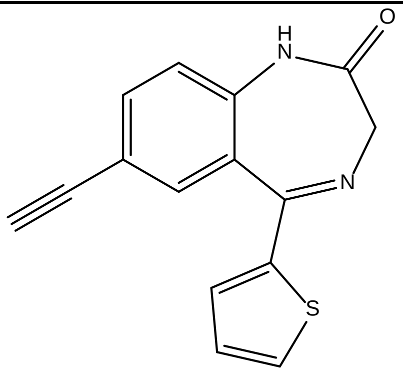 $C_{15}H_{10}N_2OS$	JC208 C15H10N2OS	22.42	18.89			5.039	

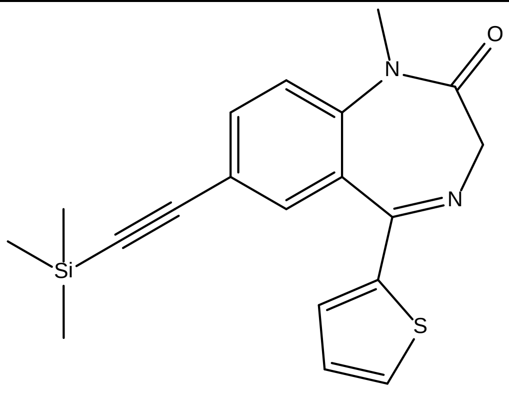 $C_{19}H_{20}N_2OSSi$	JC209 C19H20N2OSSi	294.6	334.7			249.5	
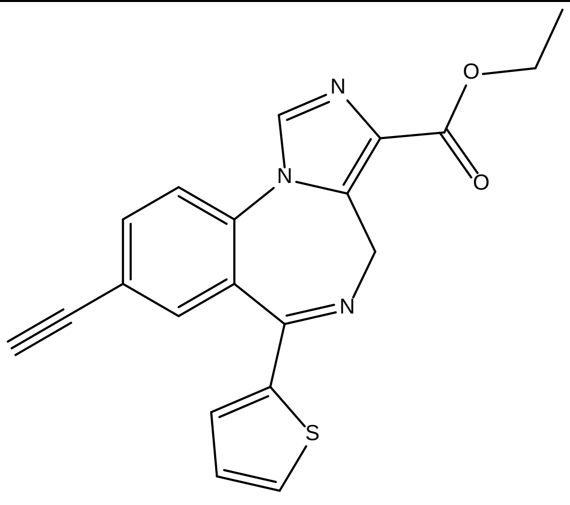 $C_{20}H_{15}N_3O_2S$	JC221 ANX1	106.2	49.4	182		7.75	362
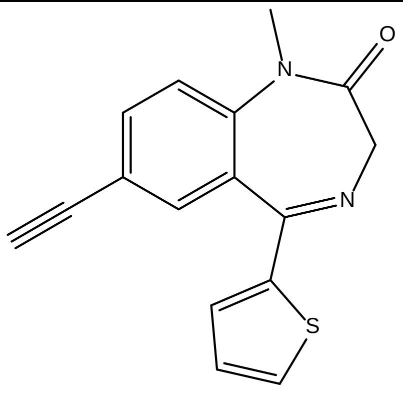 $C_{16}H_{12}N_2OS$	JC222 C16H12N2OS	86.7	45.11			17.63	
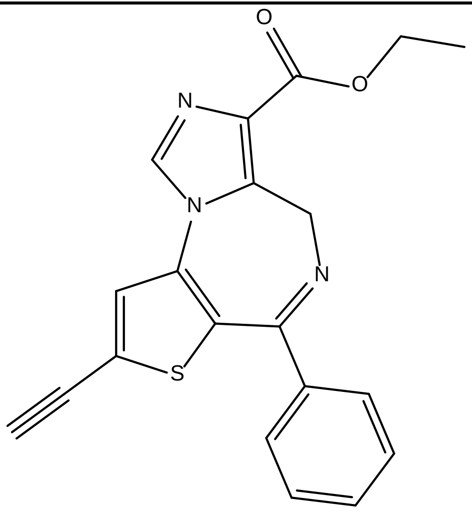 $C_{20}H_{15}N_3O_2S$	JIE-I-12	3576	454.8	768.3		4416	
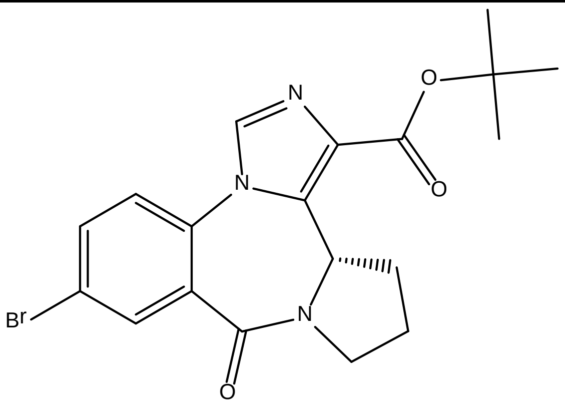 $C_{19}H_{20}BrN_3O_3$	JYI-01(C19H20N3O3Br)	59.2	159	96		10.6	2.88
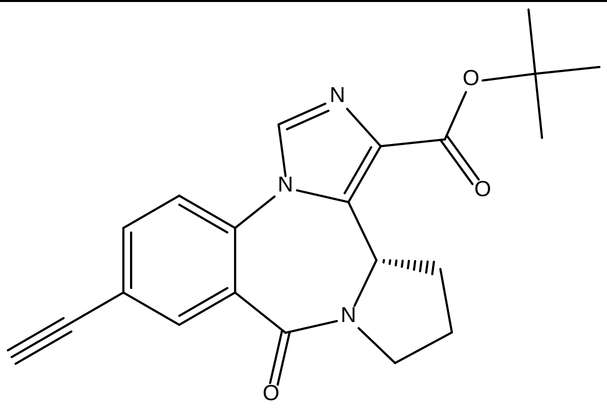 $C_{21}H_{21}N_3O_3$	JYI-03 (C21H21N3O3)	185.4	107			0.954	3.34

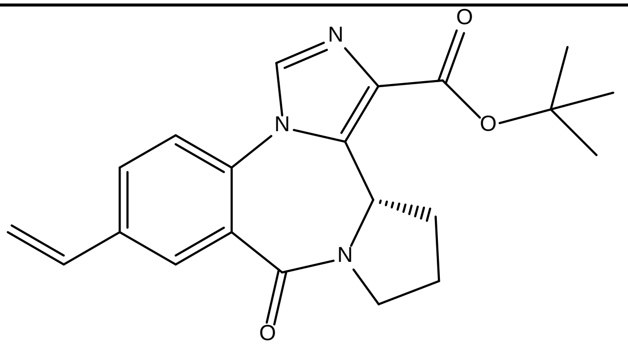 $C_{21}H_{23}N_3O_3$	JYI-04 (C21H23N3O3)	28.3	16			0.51	1.57
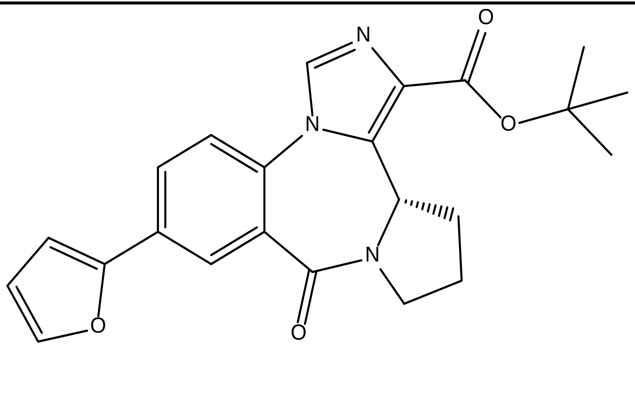 $C_{23}H_{23}N_3O_4$	JYI-06 (C23H23N3O4)	16.5	5.48	5000		12.6	5000
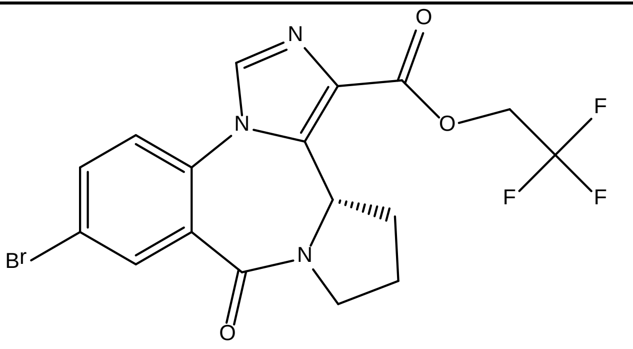 $C_{17}H_{13}BrF_3N_3O_3$	JYI-10 (C17H13N3O3F3Br)	5000	368			12.3	23
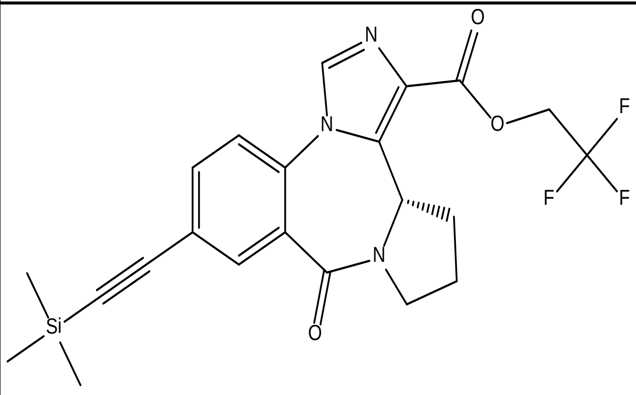 $C_{22}H_{22}F_3N_3O_3Si$	JYI-11 (C22H22N3O3F3Si)	5000	5000			648	3.97
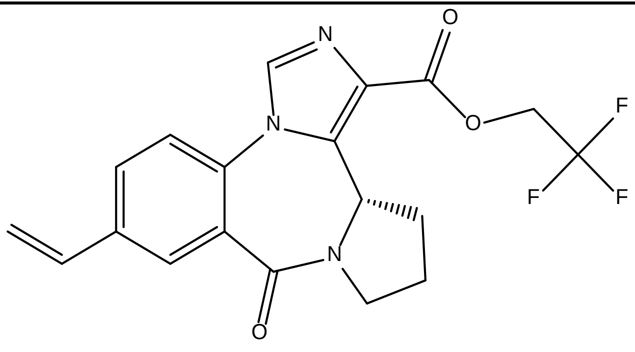 $C_{19}H_{16}F_3N_3O_3$	JYI-12 (C19H16N3O3F3)	91	39			4.5	6.8
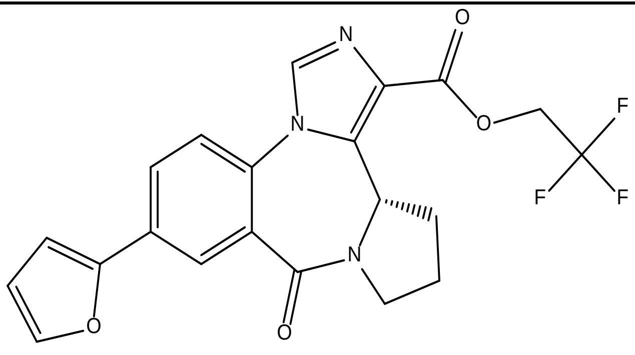 $C_{21}H_{16}F_3N_3O_4$	JYI-13 (C21H16N3O4F3)	5000	63.7			16	8.38
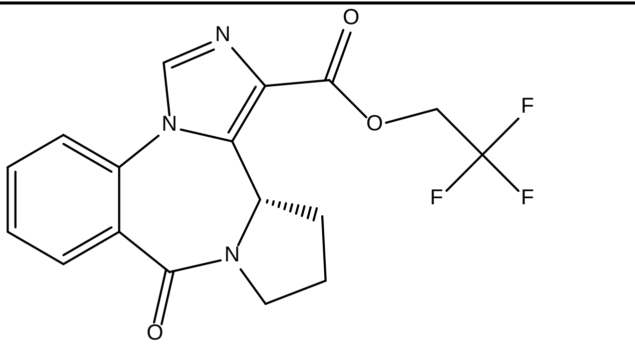 $C_{17}H_{14}F_3N_3O_3$	JYI-14 (C17H14N3O3F3)	32	25			13	565

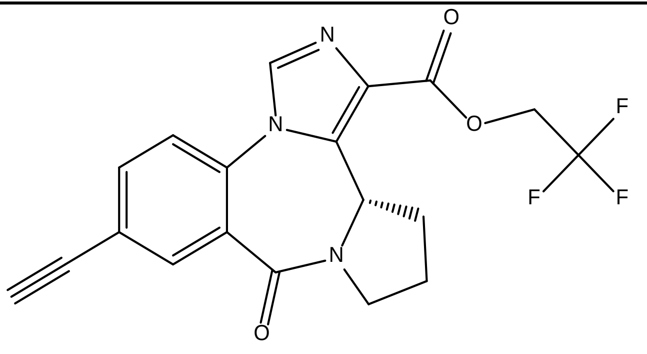 $C_{19}H_{14}F_3N_3O_3$	JYI-15 (C19H14N3O3F3)	205	812			4.8	22
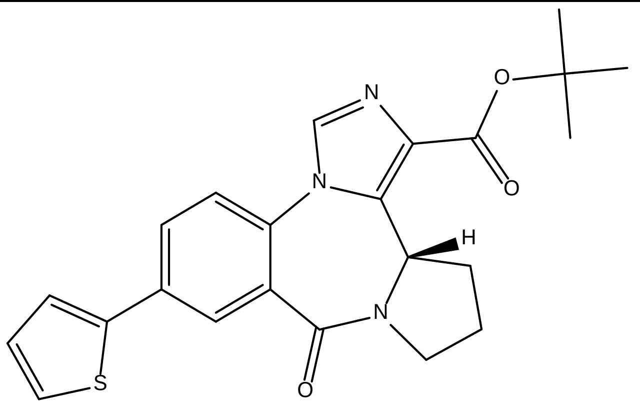 $C_{23}H_{23}N_3O_3S$	JYI-19 (C23H23N3O3S)	2.2	205			34	12.7
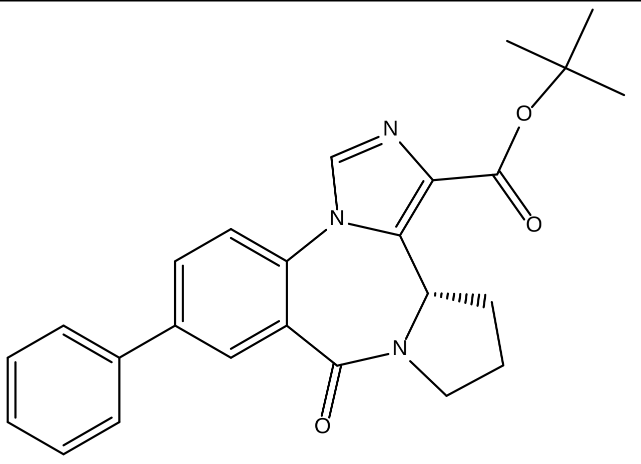 $C_{25}H_{25}N_3O_3$	JYI-20 (C25H25N3O3)	20.96	611			54	40.46
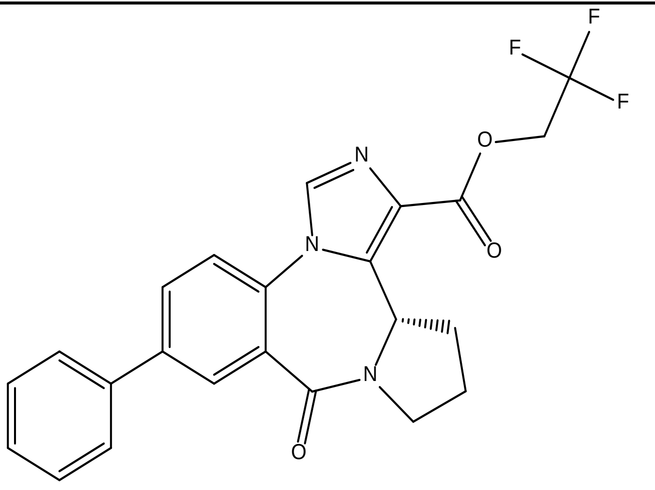 $C_{23}H_{18}F_3N_3O_3$	JYI-21 (C23H17N3O3F3)	113.7	5000			209	60.96
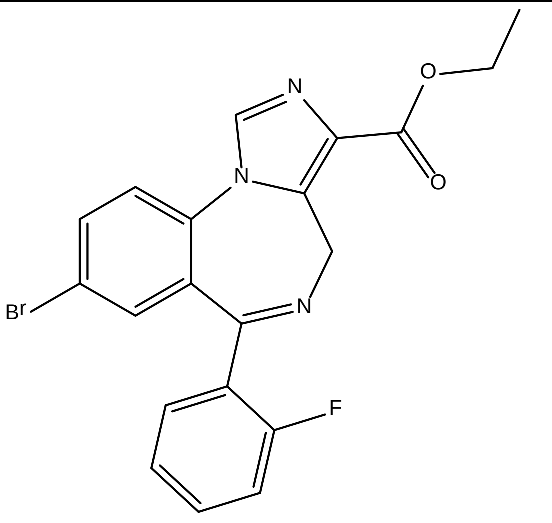 $C_{20}H_{15}BrFN_3O_2$	JYI-32 (C20H15N3O2BrF)	3.07	4.96			2.92	52.24
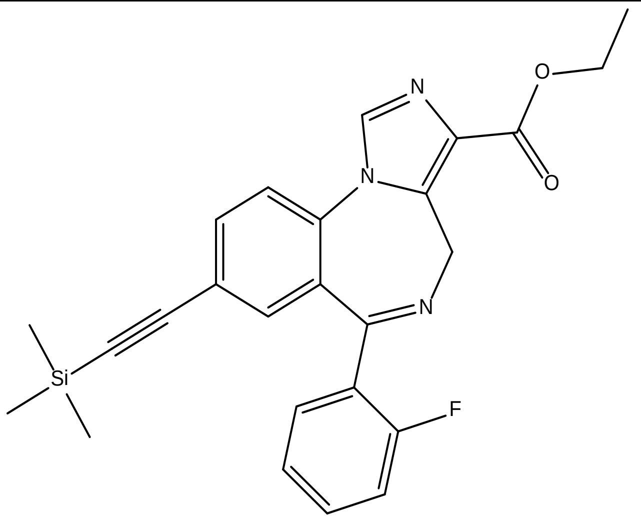 $C_{25}H_{24}FN_3O_2Si$	JYI-38 (C25H24N3O2FSi)	5000	314			55.4	5000

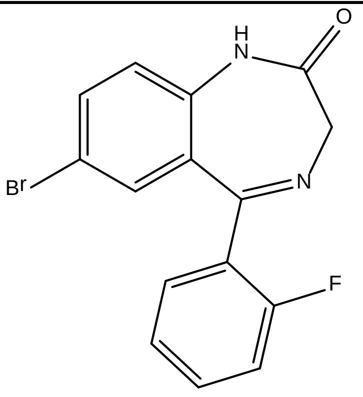 $C_{15}H_{10}BrFN_2O$	JYI-42	0.26	0.15	0.28		0.26	
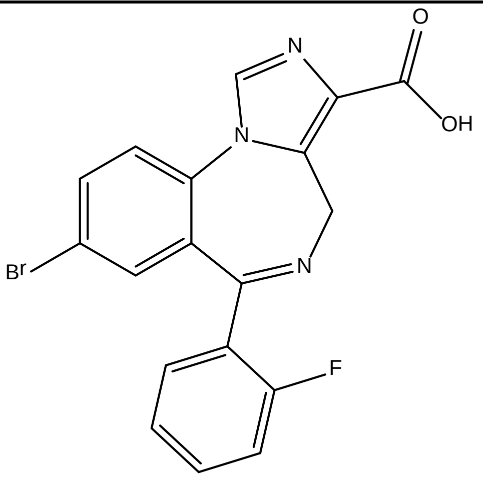 $C_{18}H_{11}BrFN_3O_2$	JYI-47	2.8	2.3	0.5		0.4	
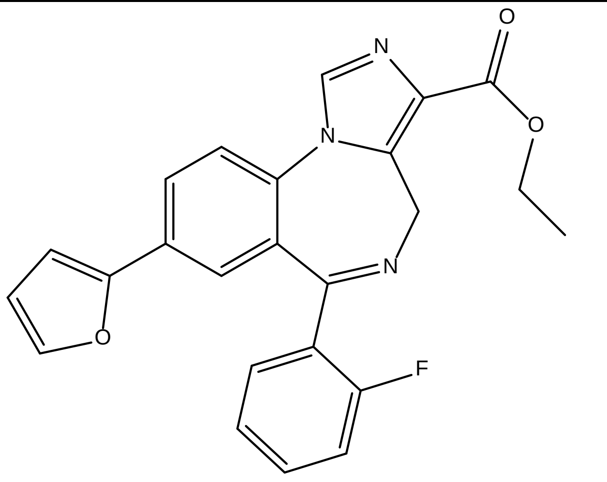 $C_{24}H_{18}FN_3O_3$	JYI-48	75.59	90.68	12.78		31.28	
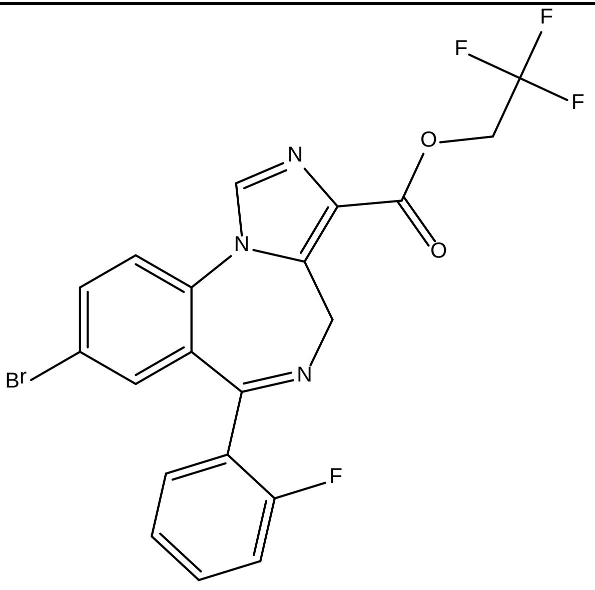 $C_{20}H_{12}BrF_4N_3O_2$	JYI-49 (C20H12N3O2F4Br)	1.87	2.38			6.7	3390
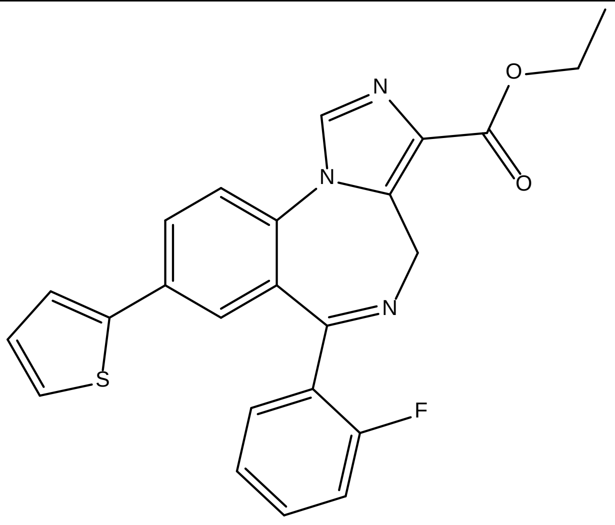 $C_{24}H_{18}FN_3O_2S$	JYI-50 (C24H18N3O2SF)	106.4	200			37.6	5040

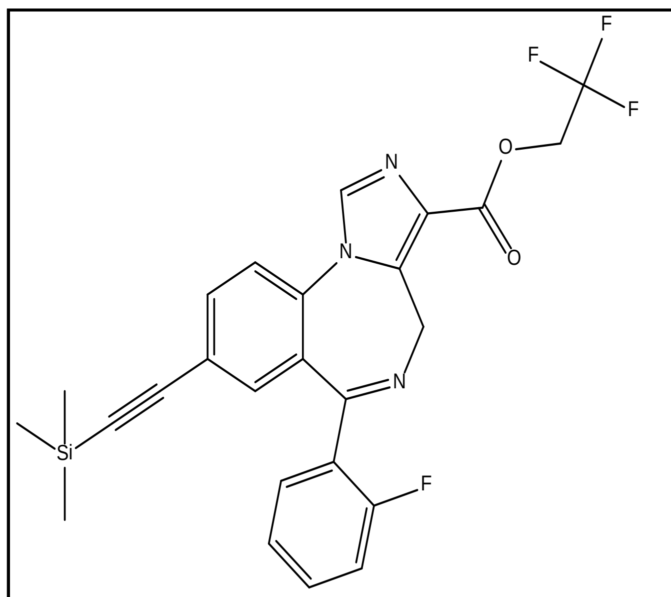 $C_{25}H_{21}F_4N_3O_2Si$	JYI-53 (C23H21N3O2F4Si)	77.4	40.4	183		133	3650
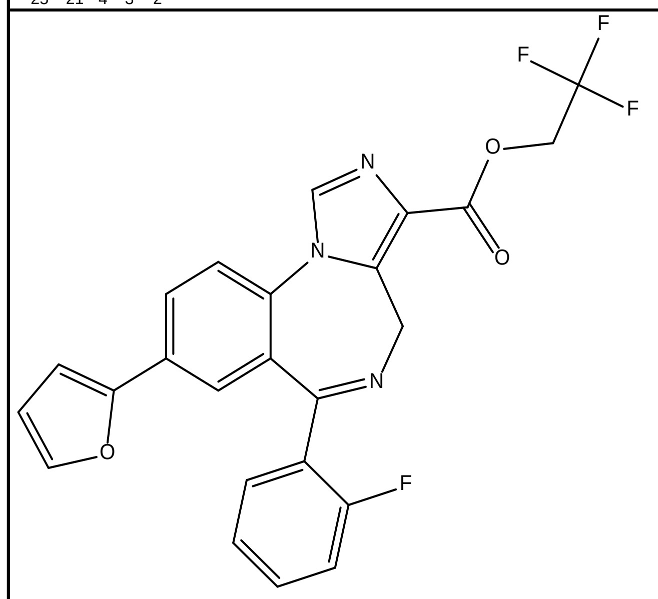 $C_{24}H_{15}F_4N_3O_3$	JYI-54 (C24H15N3O3F4)	2.89	172	6.7		57	1890
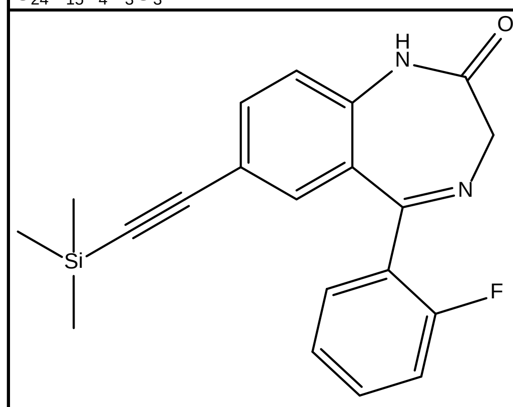 $C_{20}H_{19}FN_2OSi$	JYI-55	41.39		0.504		24.75	
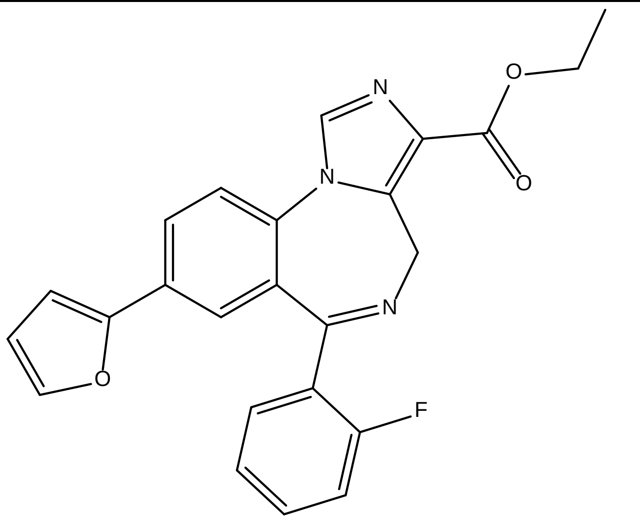 $C_{24}H_{18}FN_3O_3$	JYI-56 (C24H18N3O4F)	95.2	346	131		34.6	4140
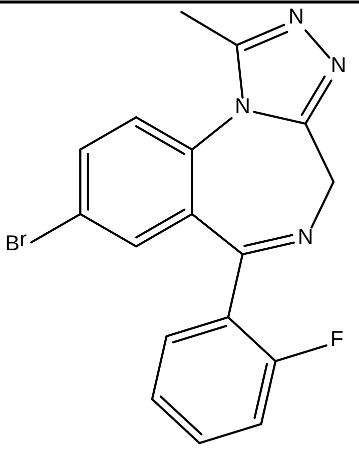 $C_{17}H_{12}BrFN_4$	JYI-57	0.08	0.08	0.13		0.04	
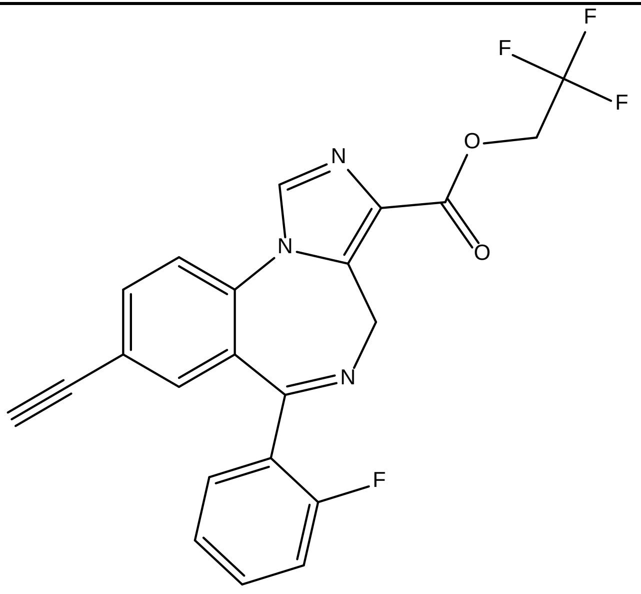 $C_{22}H_{13}F_4N_3O_2$	JYI-59 (C22H13N3O2F4)	1.08	2.6	11.82		11.5	5000

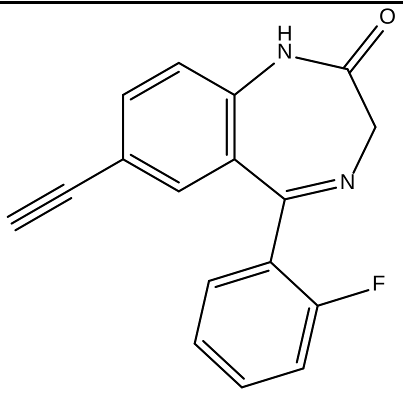 $C_{17}H_{11}FN_2O$	JYI-60 (C17H11N2OF)	3.7	1.6	4.3		1.7	5000
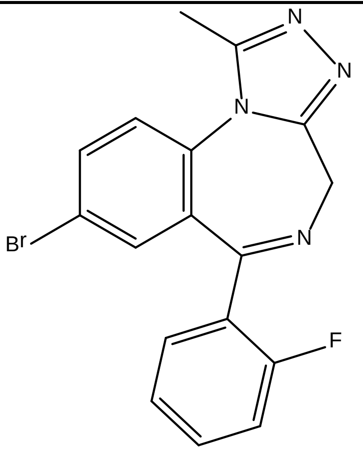 $C_{17}H_{12}BrFN_4$	JYI-64 (C17H12N4FBr)	0.3	1.11	0.62		0.9	5000
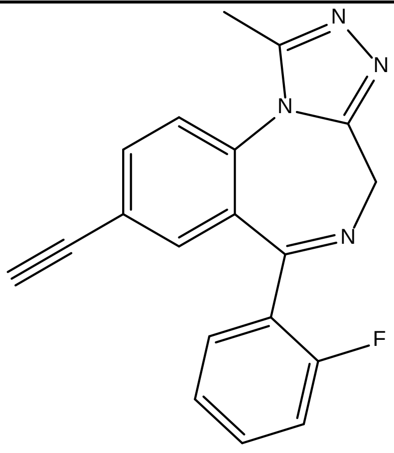 $C_{19}H_{13}FN_4$	JYI-70 (C19H13N4F)	6.3	2.1			0.56	5000
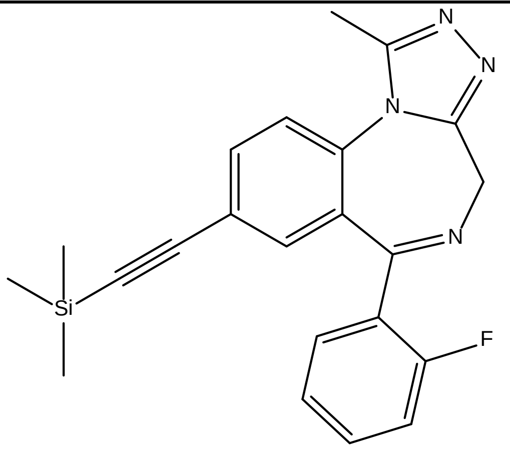 $C_{22}H_{21}FN_4Si$	JYI-72 (C22H21N4SiF)	48.5	18.5			11.5	5000
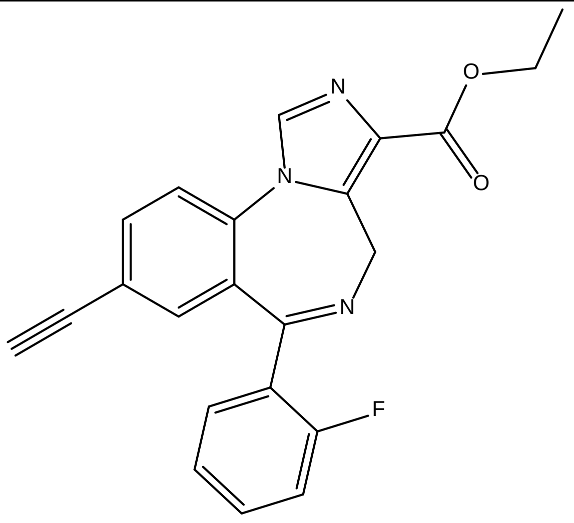 $C_{22}H_{16}FN_3O_2$	JY-XHE-053	22	12.3	34.9		0.7	
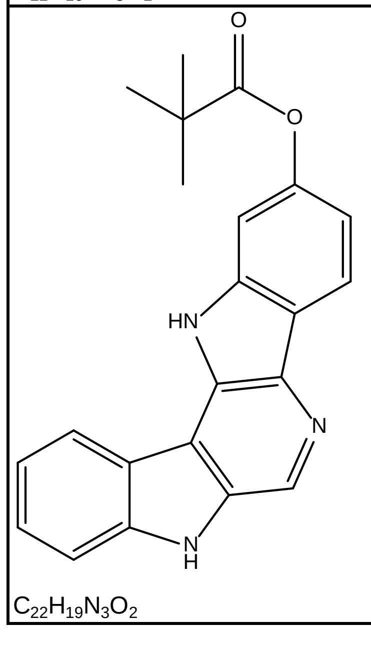 $C_{22}H_{19}N_3O_2$	LD-9	156				3000	10000

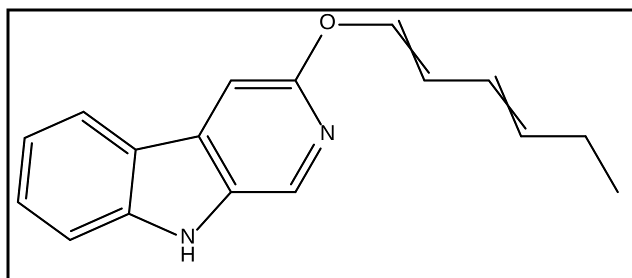 $C_{17}H_{16}N_2O$	LJD-I-0036A	245	818	869		10000	10000
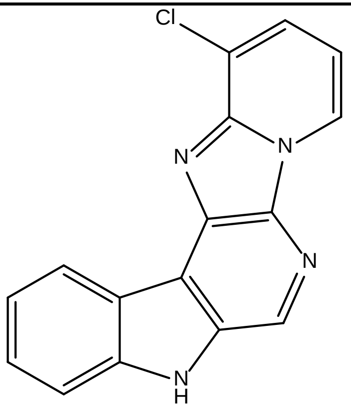 $C_{16}H_9ClN_4$	LJD-II-87	3000	3000	3000		10000	10000
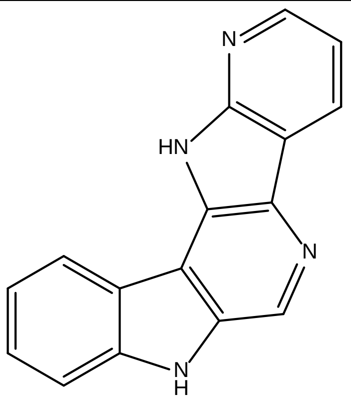 $C_{16}H_{10}N_4$	LJD-III-15E	1.93	14	19		70.8	1000
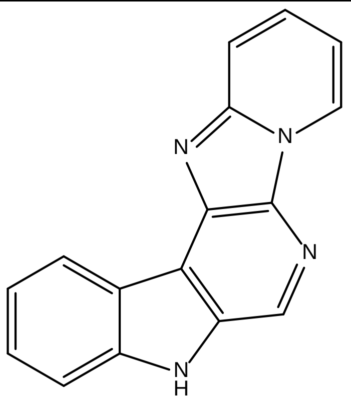 $C_{16}H_{10}N_4$	LJD-III-22A	105	227.5	284		3000	10000
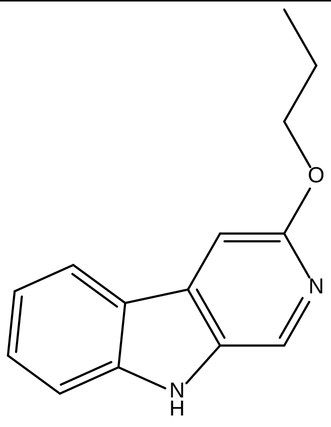 $C_{14}H_{14}N_2O$	MA-3-PROPOXYL	5.3	52.3	68.8		591	1000
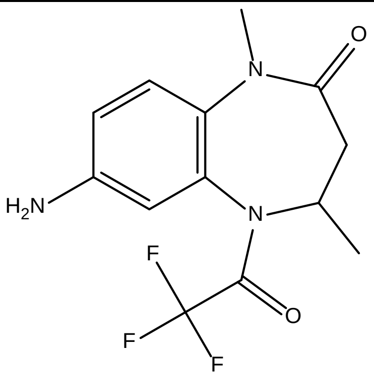 $C_{13}H_{14}F_3N_3O_2$	MH-1 (Parkistan)	3000	3000	3000	3000	3000	3000
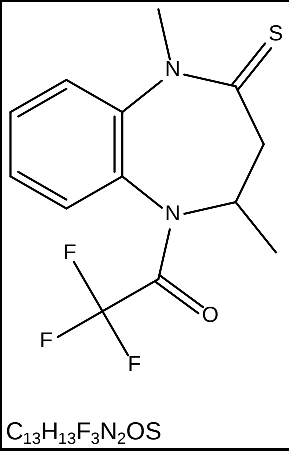 $C_{13}H_{13}F_3N_2OS$	MH-2 (Parkistan)	3000	3000	3000	3000	3000	3000

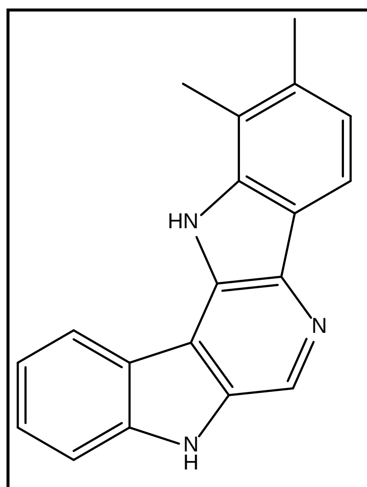 $C_{19}H_{15}N_3$	MJP-II-34	240		391		1360	10000
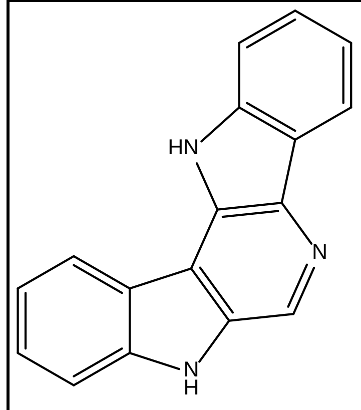 $C_{17}H_{11}N_3$	MLT-I-70	1.1	1.2	1.1		40.3	1000
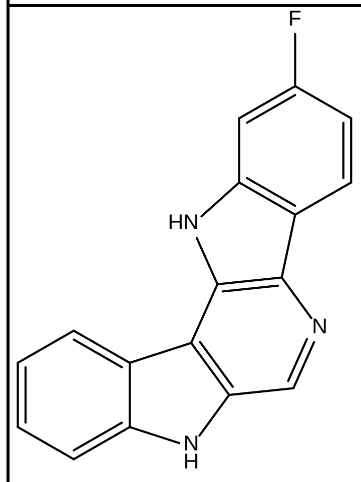 $C_{17}H_{10}FN_3$	MLT-II-16	5.05	10.41	18.4		260	10000
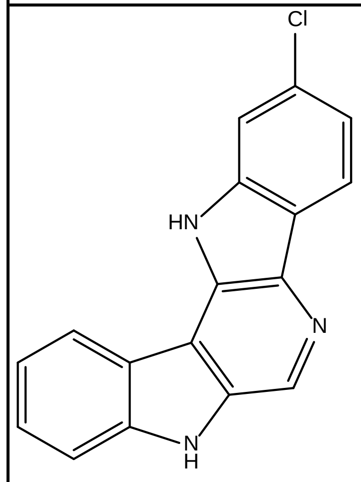 $C_{17}H_{10}ClN_3$	MLT-II-18	3.9	12.2	24.4		210	10000
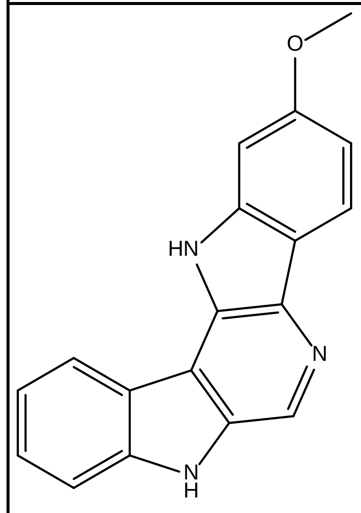 $C_{18}H_{13}N_3O$	MLT-II-18	3.4	11.7	11		225	10000
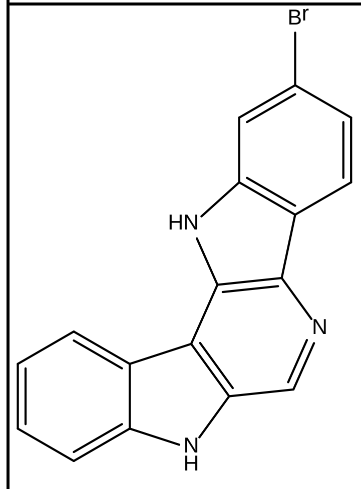 $C_{17}H_{10}BrN_3$	MLT-II-29						

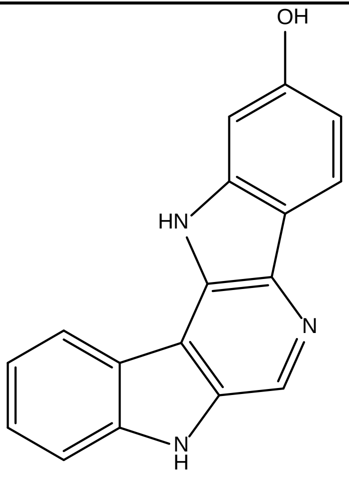 $C_{17}H_{11}N_3O$	MLT-II-34	7.04	15.95	22.3		158	1000
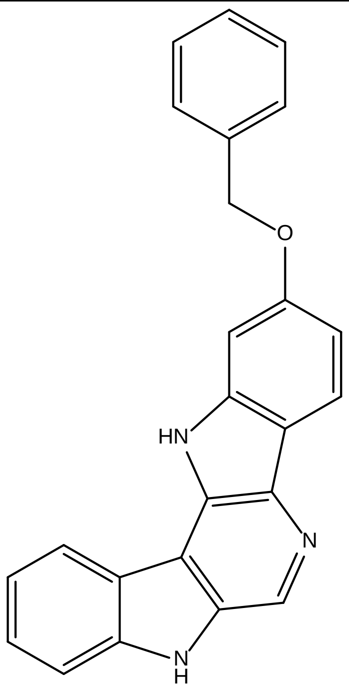 $C_{24}H_{17}N_3O$	MLT-II-87	432	3000	3000		10000	10000
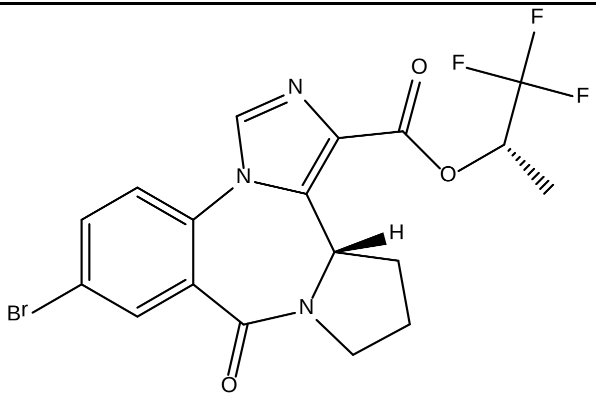 $C_{18}H_{15}BrF_3N_3O_3$	MMB-II-37	872	2602	1003	95	98	115
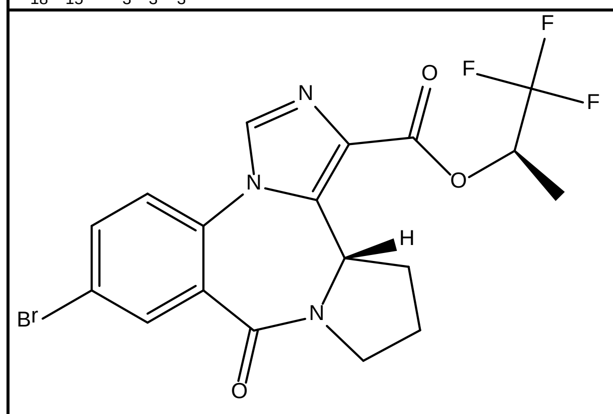 $C_{18}H_{15}BrF_3N_3O_3$	MMB-II-43	1353	5173	1535	98	132	78
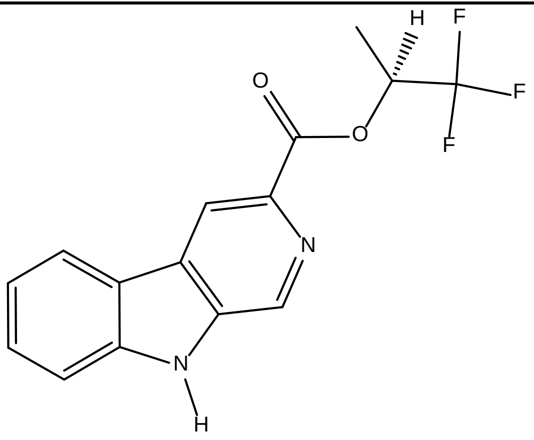 $C_{15}H_{11}F_3N_2O_2$	MMB-II-57	90	931	172	3000	1000	3000
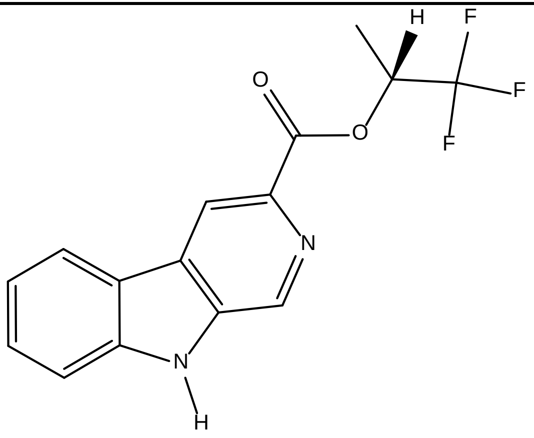 $C_{15}H_{11}F_3N_2O_2$	MMB-II-68A	27	343.3	453	3000	1847	3000

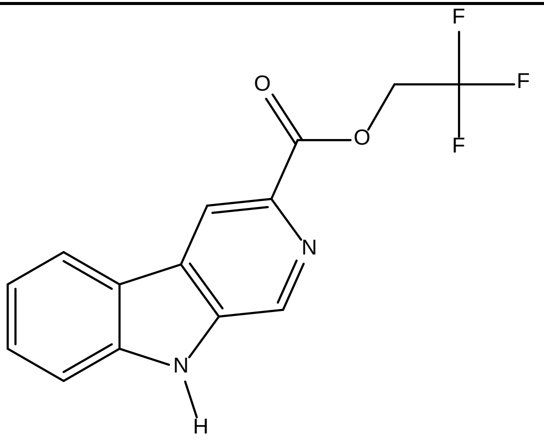 $C_{14}H_9F_3N_2O_2$	MMB-II-74	3	24.5	41.7	500	125.7	1000
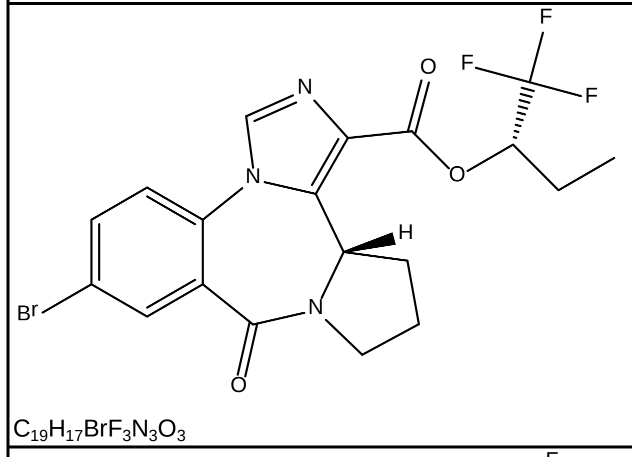 $C_{19}H_{17}BrF_3N_3O_3$	MMB-II-87	200	333	107	109	5.4	333
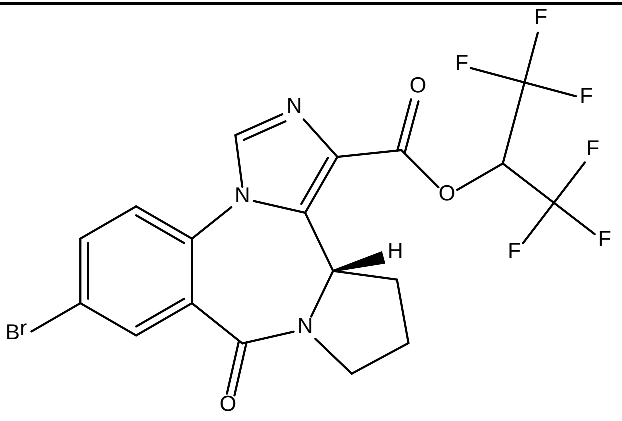 $C_{18}H_{12}BrF_6N_3O_3$	MMB-II-89	333	333	333	333	43	333
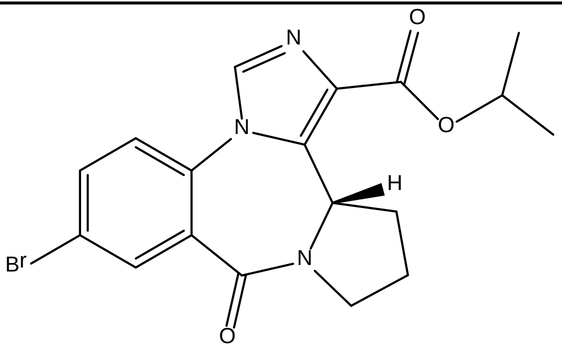 $C_{18}H_{18}BrN_3O_3$	MMB-II-90	20	24	5.7	9	0.25	36
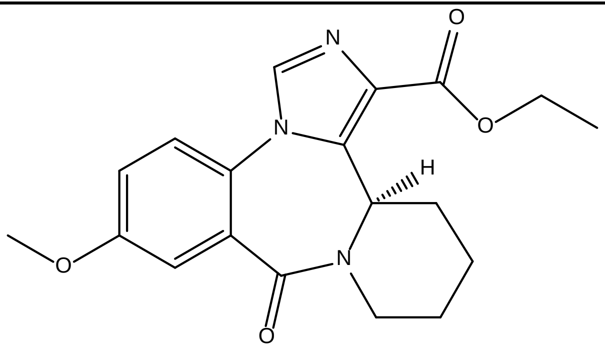 $C_{19}H_{21}N_3O_4$	MMB-II-99	>333	333	333	333	333	333
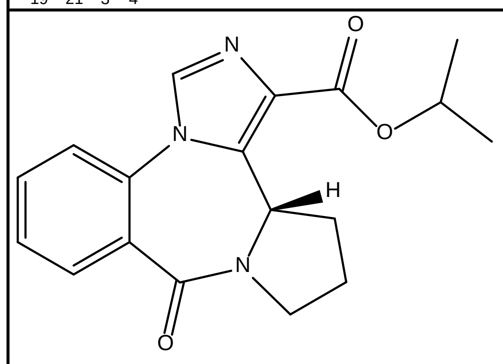 $C_{18}H_{19}N_3O_3$	MMB-III-016	3	1.97	2	1074	0.26	211
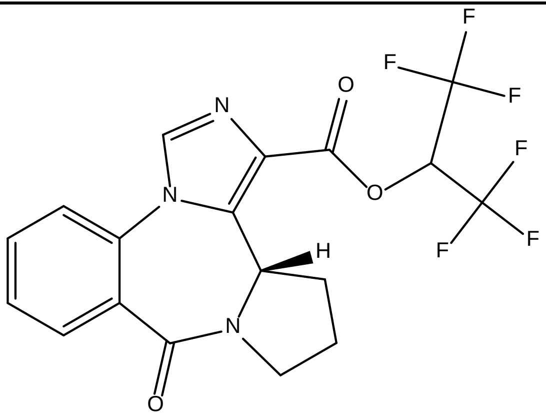 $C_{18}H_{13}F_6N_3O_3$	MMB-III-018	117	140	78	3500	14	976

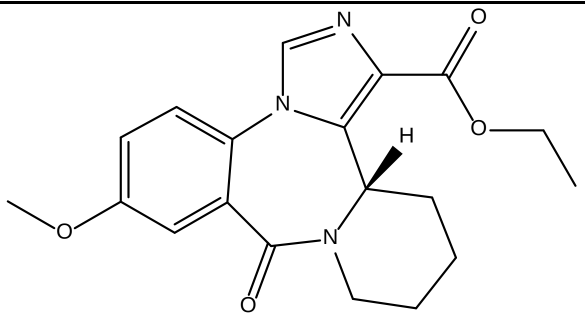 $C_{19}H_{21}N_3O_4$	MMB-III-019	3333	3333	3333	3500	889	3500
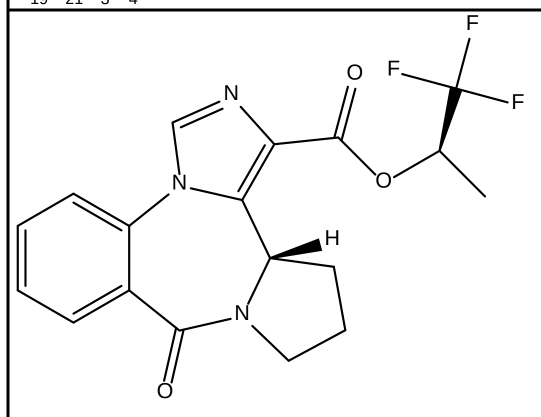 $C_{18}H_{16}F_3N_3O_3$	MMB-III-14	13	13	6.9	333	1.1	333
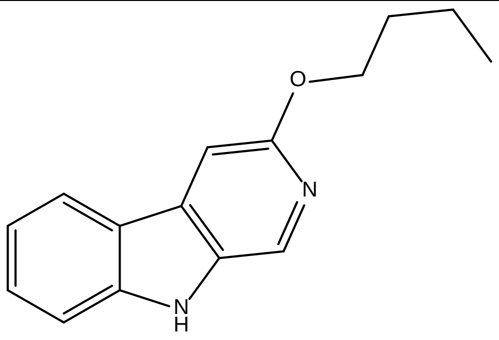 $C_{15}H_{16}N_2O$	MSA-0-28	36.9	194	245		1000	1000
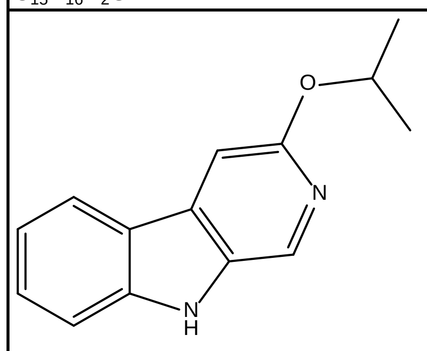 $C_{14}H_{14}N_2O$	MSA-3-IPBC	283	3000	3000		10000	10000
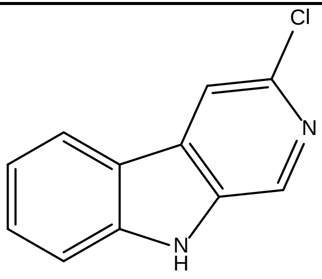 $C_{11}H_7ClN_2$	MSA-I-71	60.4	125.3	126		1000	10000
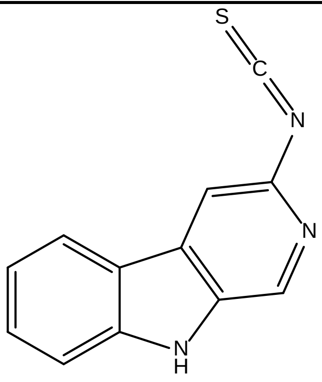 $C_{12}H_7N_3S$	MSA-II-35	67.2	120	141		3000	10000
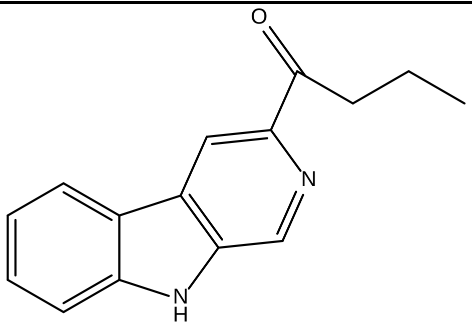 $C_{15}H_{14}N_2O$	MSA-IV-35	2.1	16	21		995	3000

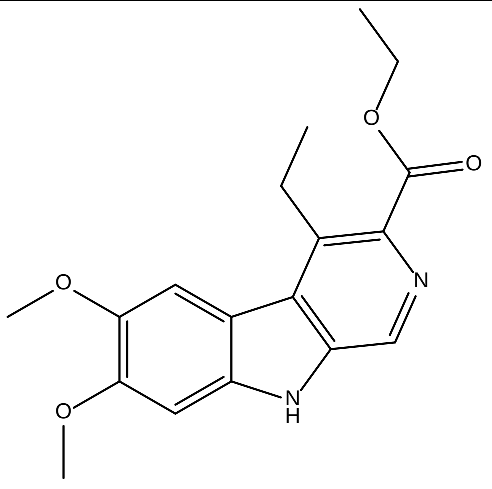 $C_{18}H_{20}N_2O_4$	MSR-I-032	6.2	18.7	4		3.3	74.9
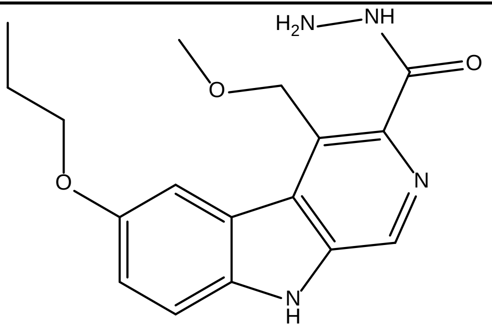 $C_{17}H_{20}N_4O_3$	MSR-I-036	46	422	400		107.5	300
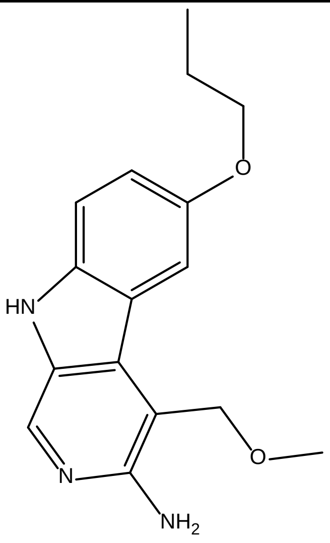 $C_{16}H_{19}N_3O_2$	MSR-I-046	45	540	700		380	3000
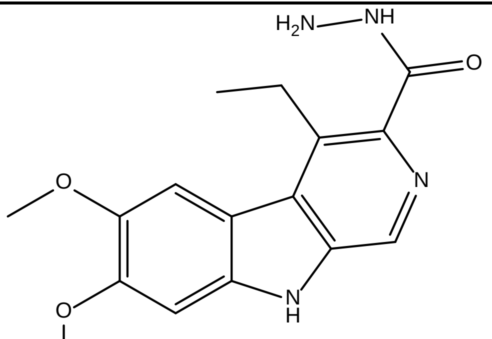 $C_{16}H_{18}N_4O_3$	MSR-I-049	896	1000	570		91.5	3000
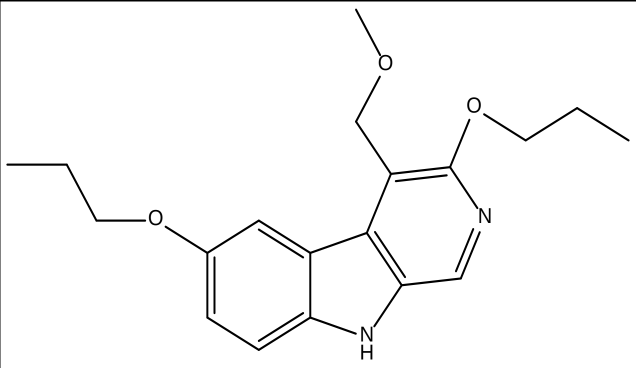 $C_{19}H_{24}N_2O_3$	MSR-I-056	24.6	214.4	270.4		331.9	1000
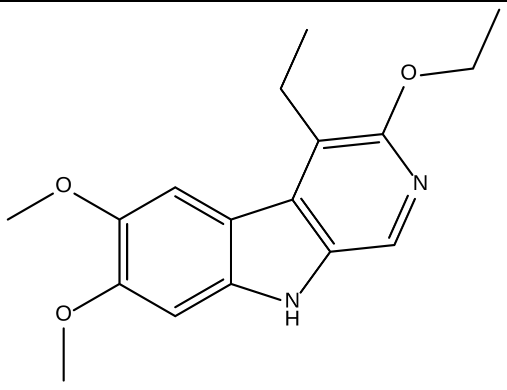 $C_{17}H_{20}N_2O_3$	MSR-I-057	>300	300	300		300	300

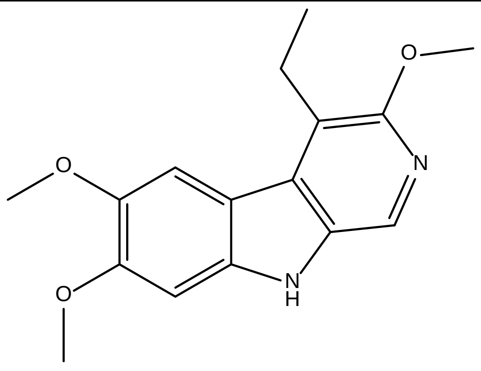 $C_{16}H_{18}N_2O_3$	MSR-I-100	300	300	300		300	300
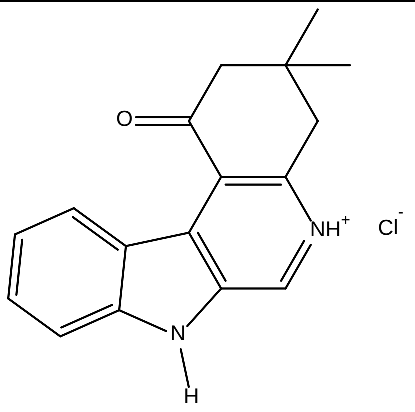 $C_{17}H_{17}ClN_2O$	N-111	691		4209	3000	3439	3000
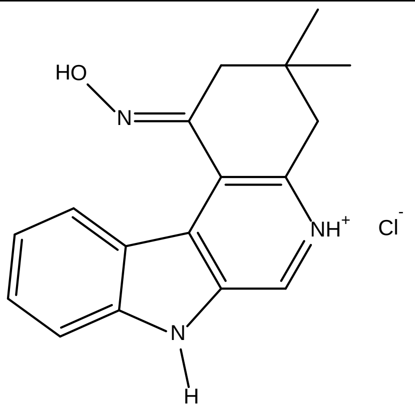 $C_{17}H_{18}ClN_3O$	N-112	296		5260	3000	3000	3000
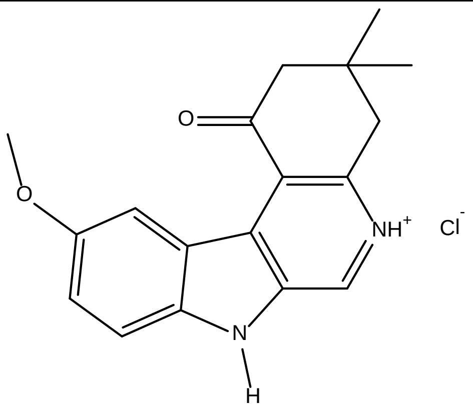 $C_{18}H_{19}ClN_2O_2$	N-120	422		1000	3000	932	3000
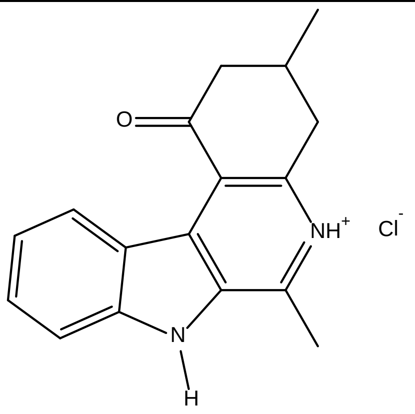 $C_{17}H_{17}ClN_2O$	N-26	1000		3000	3000	3000	3000
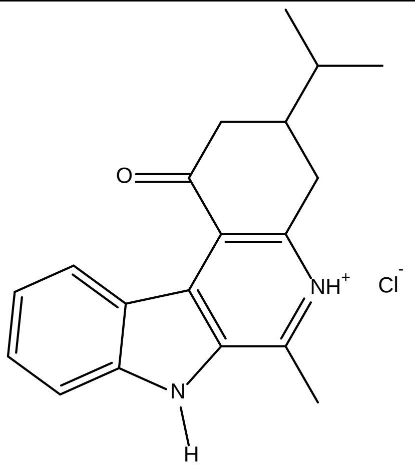 $C_{19}H_{21}ClN_2O$	N-30	1491		2000	2000	2000	2000
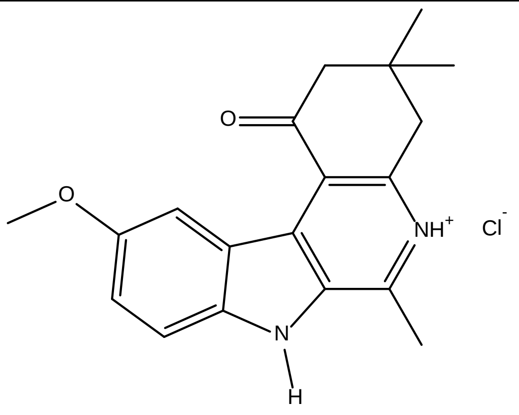 $C_{19}H_{21}ClN_2O_2$	N-80	1614.4		2000	2000	2000	2000

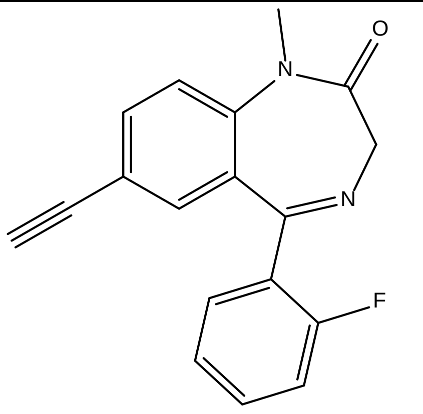 $C_{18}H_{13}FN_2O$	OMB-18	3.9	1.2	3.4	1733	0.8	5
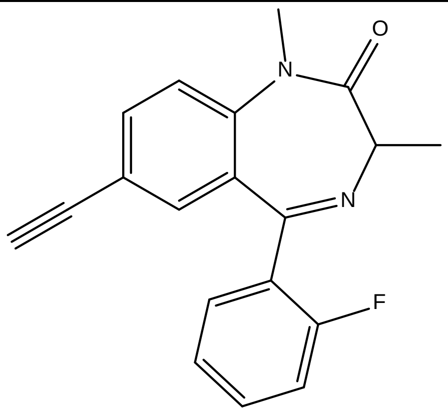 $C_{19}H_{15}FN_2O$	OMB-19	22	4.6	20	3333	3.5	40
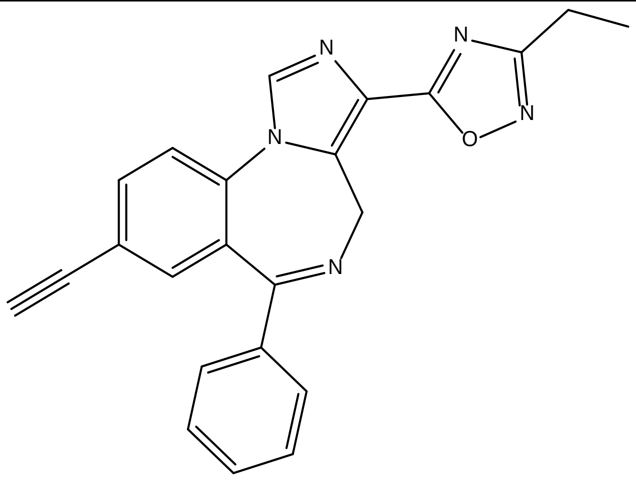 $C_{23}H_{17}N_5O$	PS-1-26 C23H17N5O	933.5	366	260		393.5	5000
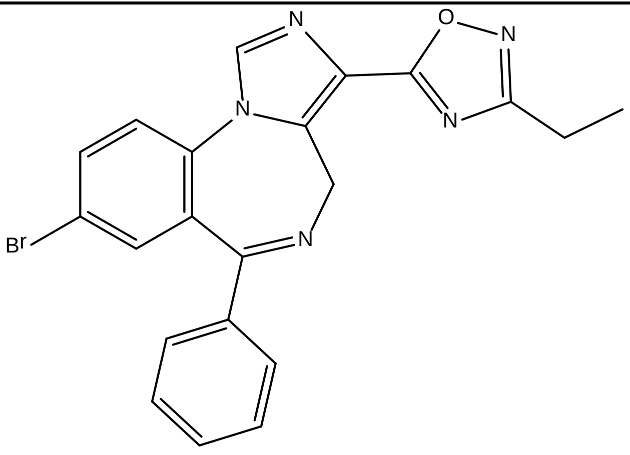 $C_{21}H_{16}BrN_5O$	PS-1-27 C21H16N5BrO	182	294			86	5000
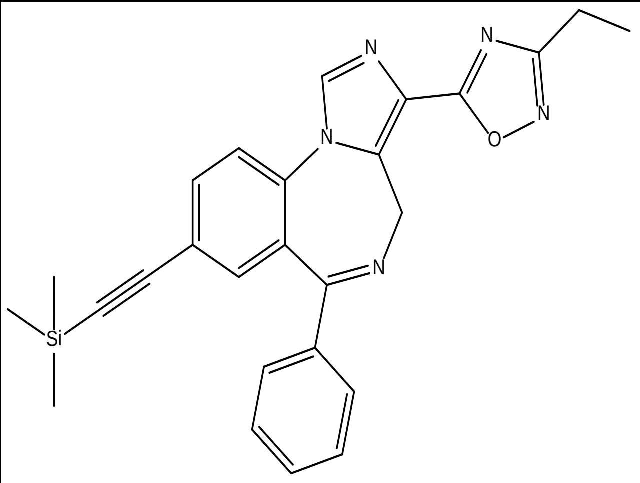 $C_{26}H_{25}N_5OSi$	PS-1-28 C26H25N5OSi	5000	5000			5000	5000
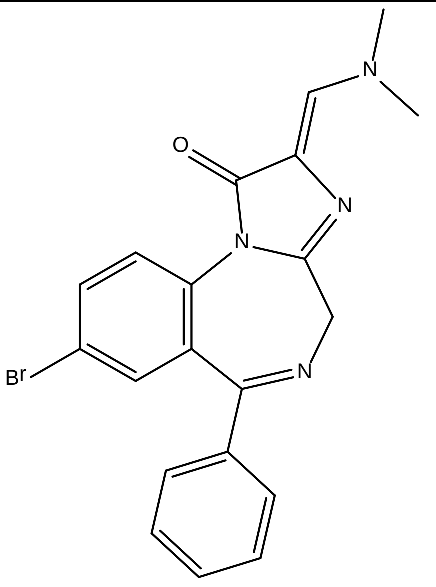 $C_{20}H_{17}BrN_4O$	PS-1-34B		4.198	3.928			

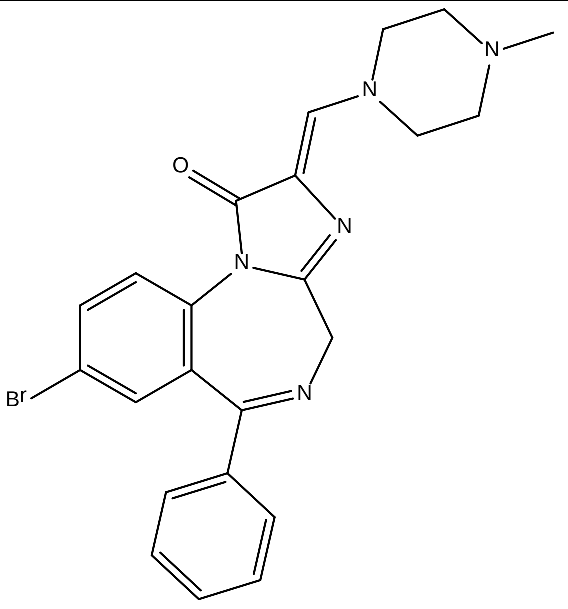 $C_{23}H_{22}BrN_5O$	PS-1-35		16.03	24.41			
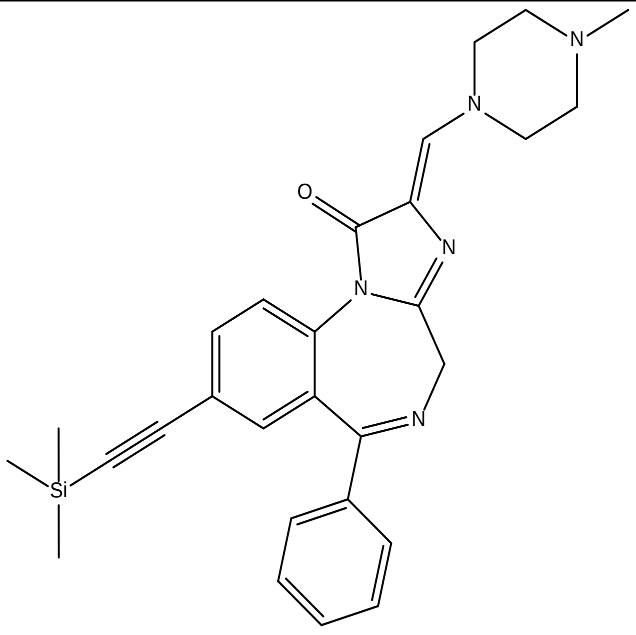 $C_{28}H_{31}N_5OSi$	PS-1-36			939.1			
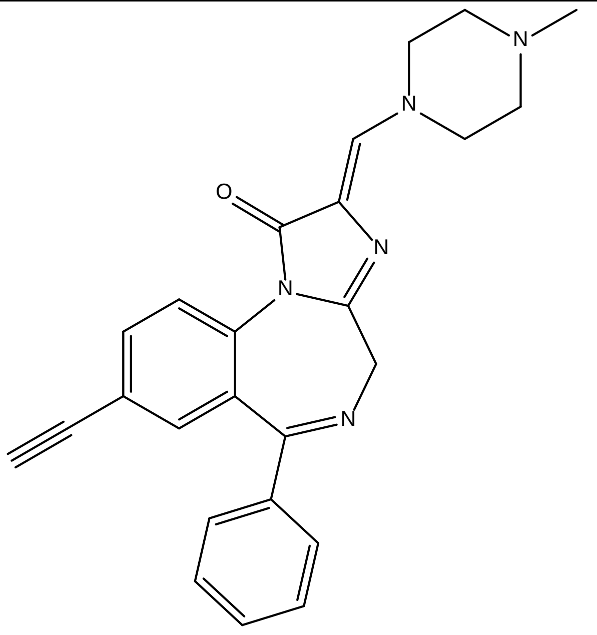 $C_{25}H_{23}N_5O$	PS-1-37	193	35	435		22	5000
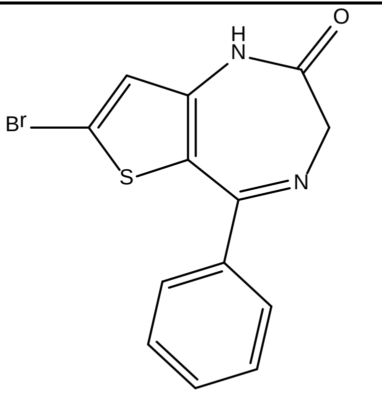 $C_{13}H_9BrN_2OS$	PS-I-68	3134	481.3	679.5		101.3	
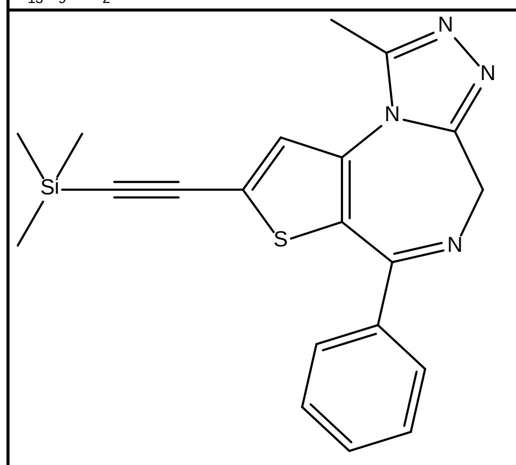 $C_{20}H_{20}N_4SSi$	PS-I-71	145.8	122.8	214.2		119.2	

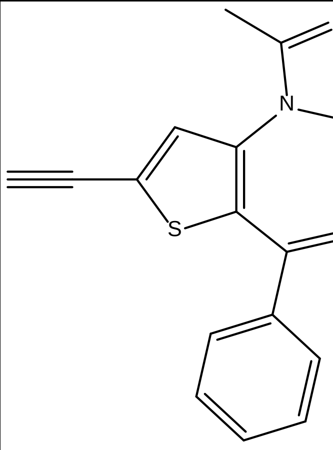 $C_{17}H_{12}N_4S$	PS-I-72	123	31	386		34	5000
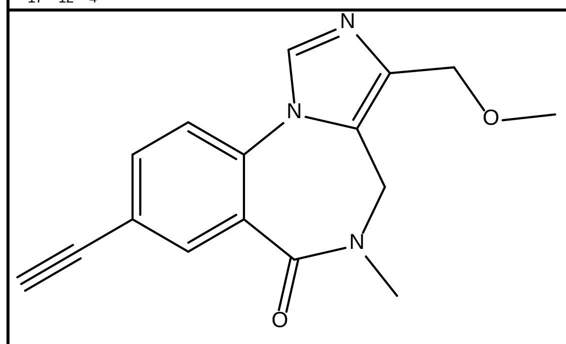 $C_{16}H_{15}N_3O_2$	PWIII-012	1000	1000	1000		166	1000
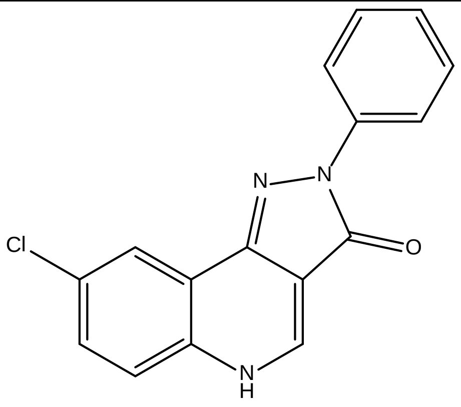 $C_{16}H_{10}ClN_3O$	PWZ-0071	0.23	0.17	0.12		0.44	17.31
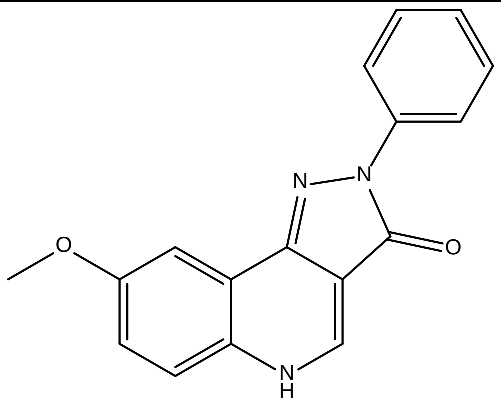 $C_{17}H_{13}N_3O_2$	PWZ-007A	0.11	0.1	0.09		0.2	10
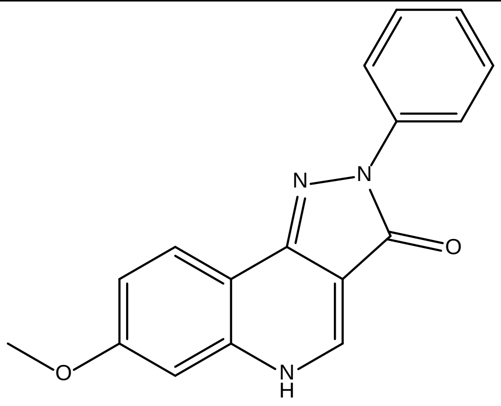 $C_{17}H_{13}N_3O_2$	PWZ-009A1	1.34	1.31	1.26		0.84	2.03
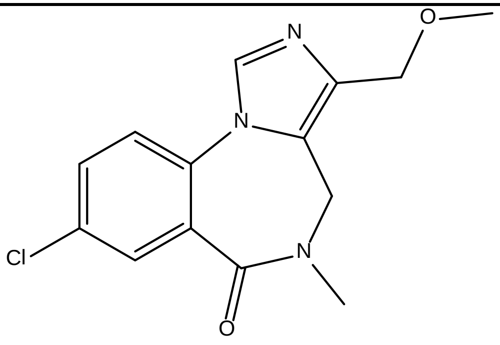 $C_{14}H_{14}ClN_3O_2$	PWZ-029	300	300	300		38.8	300

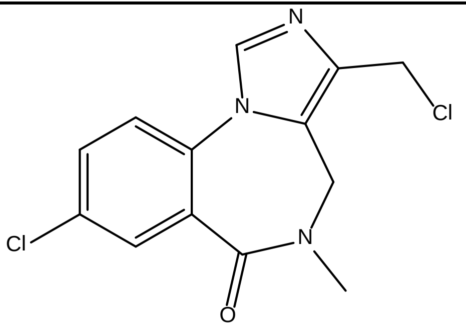 $C_{13}H_{11}Cl_2N_3O$	PWZ-031A	300	300	300		28.5	300
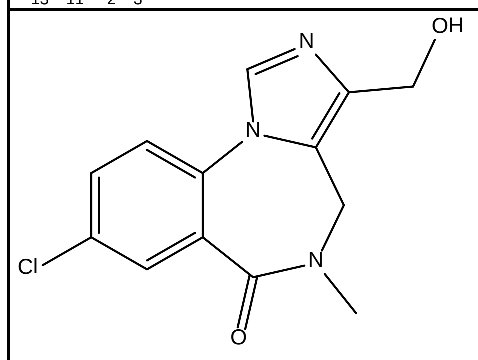 $C_{13}H_{12}ClN_3O_2$	PWZ-034A	300	300	300		300	300
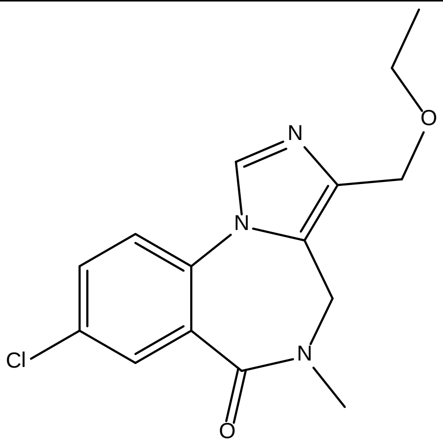 $C_{15}H_{16}ClN_3O_2$	PWZ-035A	300	300	300		82.7	300
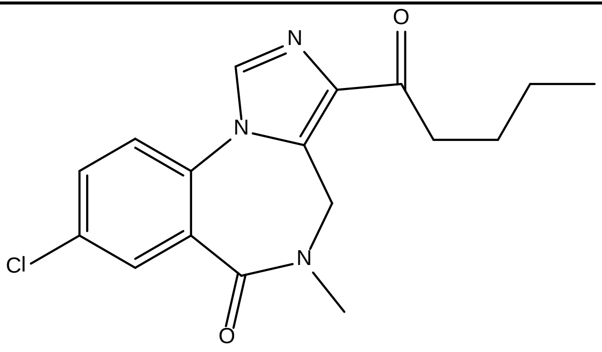 $C_{17}H_{18}ClN_3O_2$	PWZ-049	1581	2865	2739		166	2930
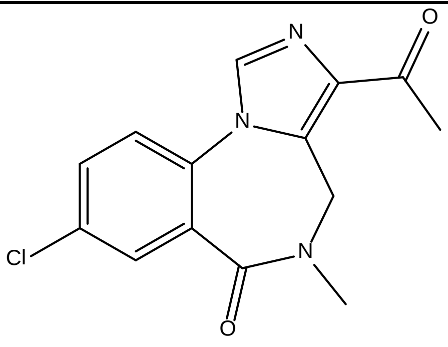 $C_{14}H_{12}ClN_3O_2$	PWZ-051	17535	33834	22125		2612	29500
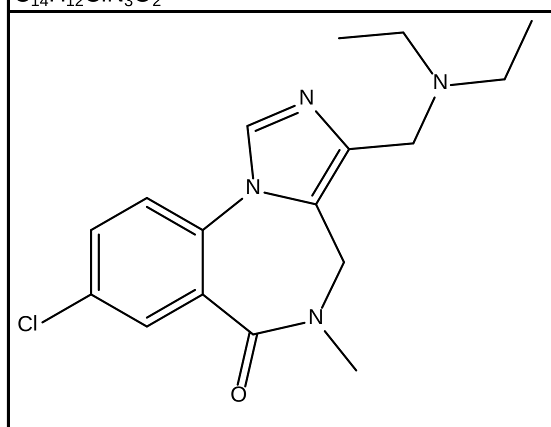 $C_{17}H_{21}ClN_4O$	PWZ-057	9483	30000	15409		2583	30160

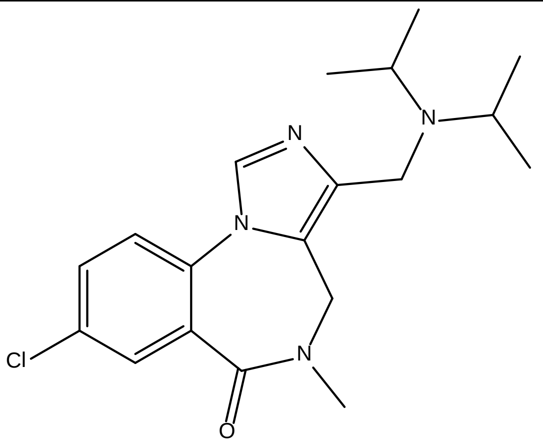 $C_{19}H_{25}ClN_4O$	PWZ-058	4201	12590	6266		1346	8600
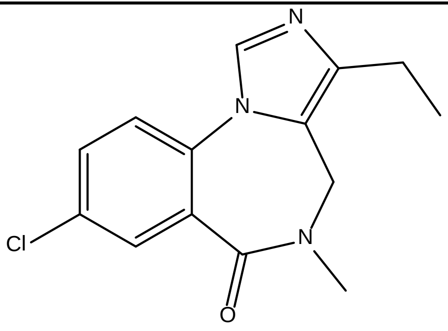 $C_{14}H_{14}ClN_3O$	PWZ-066	408	1527	1125		182	3648
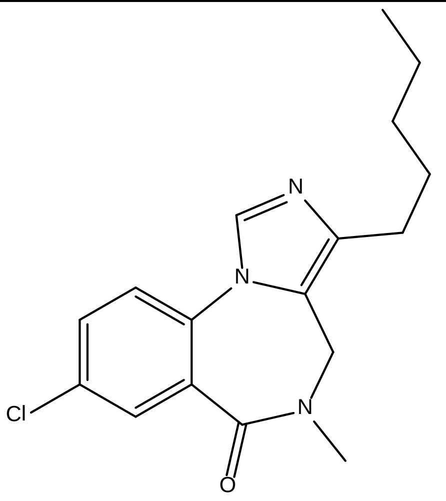 $C_{17}H_{20}ClN_3O$	PWZ-068	2050	2900	2907		369	960
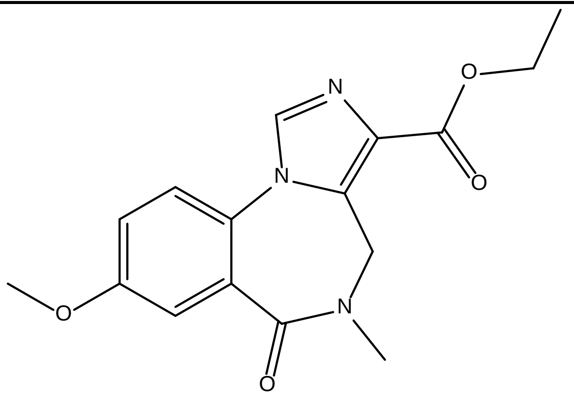 $C_{16}H_{17}N_3O_4$	PWZ-085	4.86	13	8.5		0.55	40
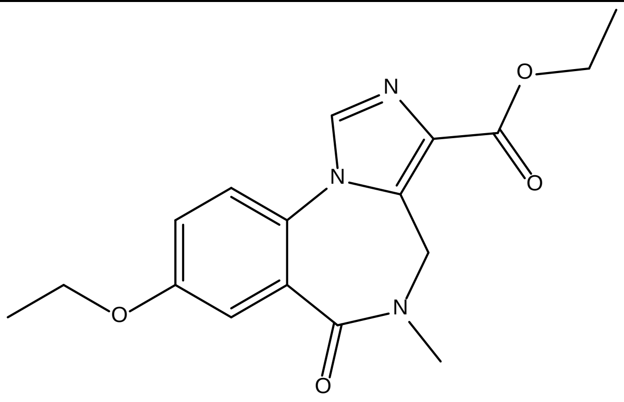 $C_{17}H_{19}N_3O_4$	PWZ-096	11.1	36	16.9		1.07	51.5
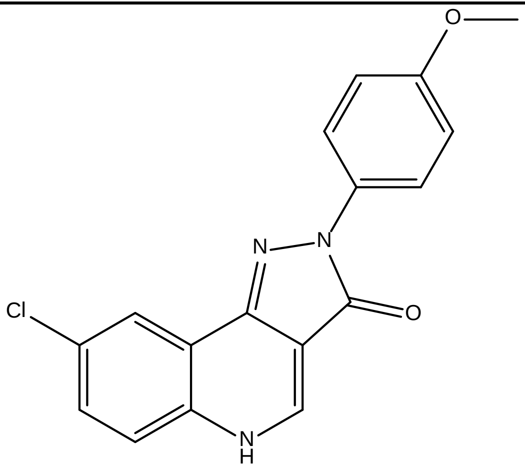 $C_{17}H_{12}ClN_3O_2$	PZII-028	0.2		0.2		0.32	1.9

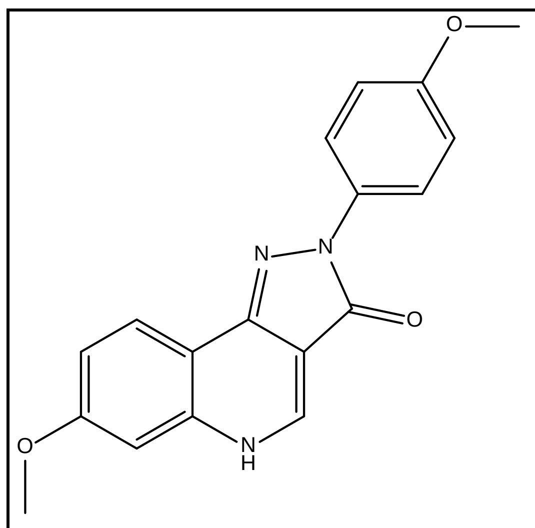 $C_{18}H_{15}N_3O_3$	PZII-029	0.34		0.79		0.52	10
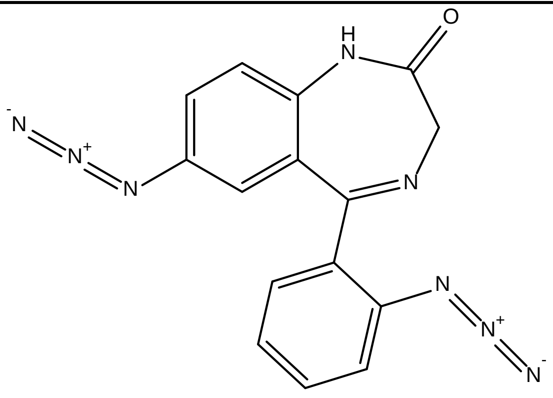 $C_{15}H_{10}N_8O$	QH-133	2164		4620		1635	1000
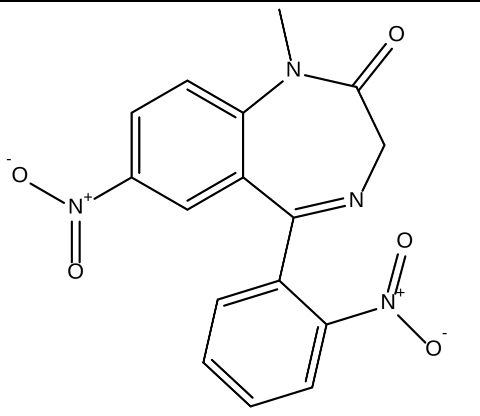 $C_{16}H_{12}N_4O_5$	QH-146	0.49		0.76		7.7	10000
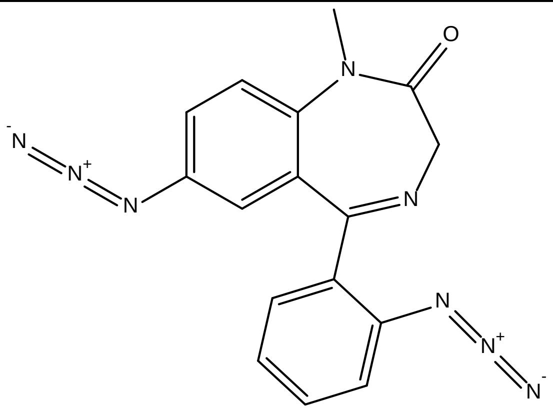 $C_{16}H_{12}N_8O$	QH-149						
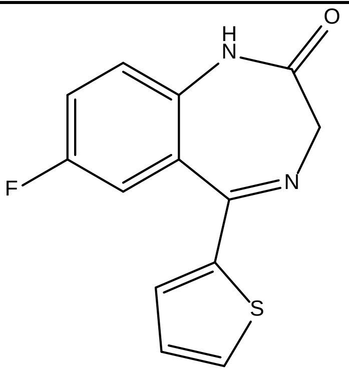 $C_{13}H_9FN_2OS$	QH-27	175	335	405		150	300
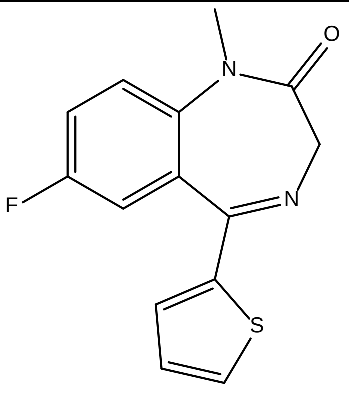 $C_{14}H_{11}FN_2OS$	QH-28	141	215	205		65.5	300
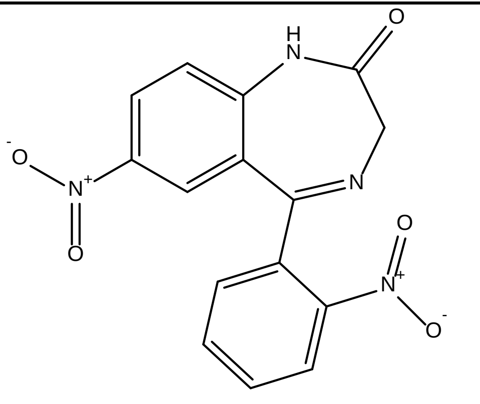 $C_{15}H_{10}N_4O_5$	QH-65						

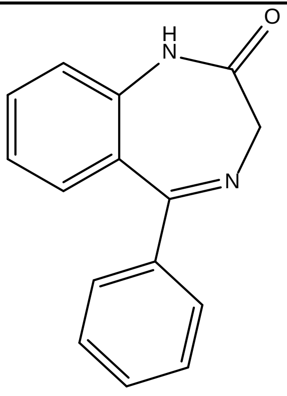 $C_{15}H_{12}N_2O$	QH-II-058b	288	233	350		140	3000
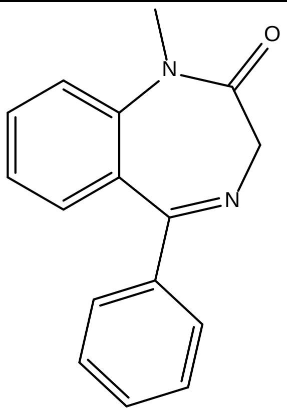 $C_{16}H_{14}N_2O$	QH-II-059	81	138	318		98	3000
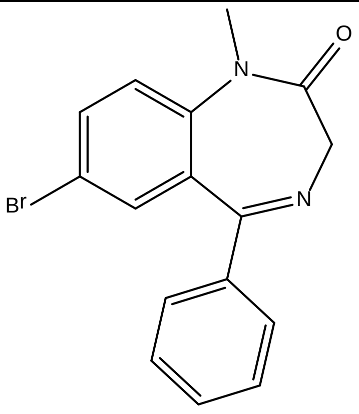 $C_{16}H_{13}BrN_2O$	QH-II-063	9.4	9.3	31		7.7	3000
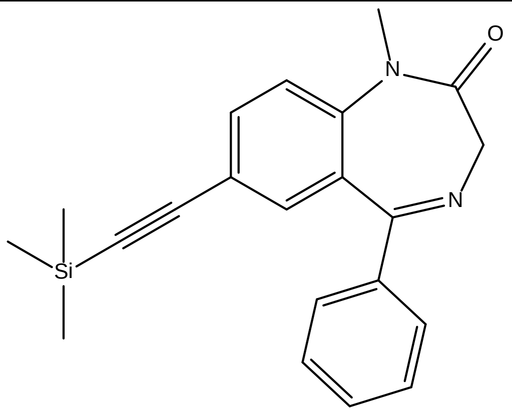 $C_{21}H_{22}N_2OSi$	QH-II-065	94	73	203		63	3000
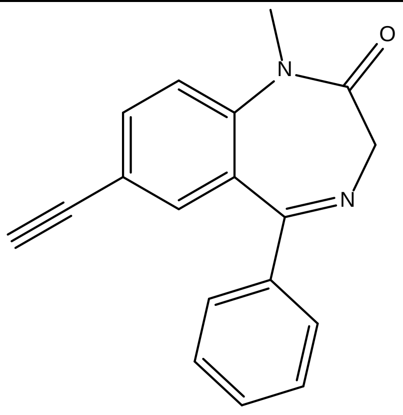 $C_{18}H_{14}N_2O$	QH-II-066	76.3	42.1	47.4	2000	6.8	3000
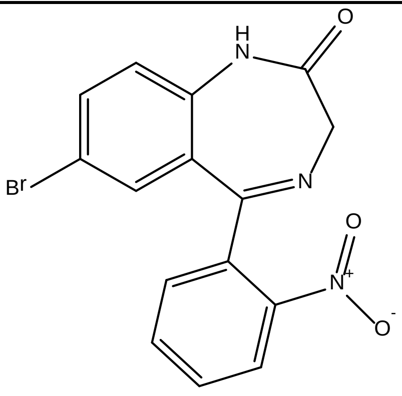 $C_{15}H_{10}BrN_3O_3$	QH-II-067a	16	31	52		199	3000

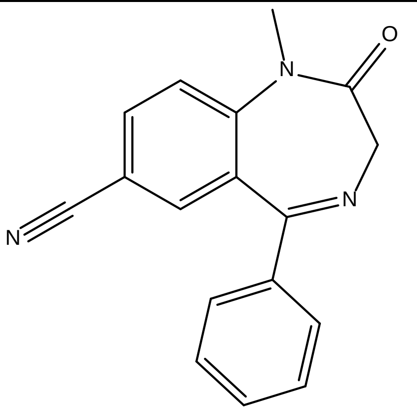 $C_{17}H_{13}N_3O$	QH-II-072	320	310	350		265	3000
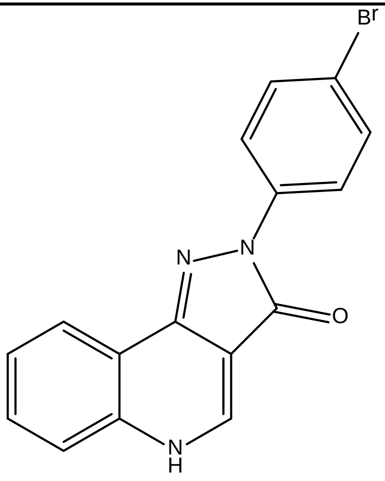 $C_{16}H_{10}BrN_3O$	QH-II-075	0.18	0.21	0.25		1.3	40
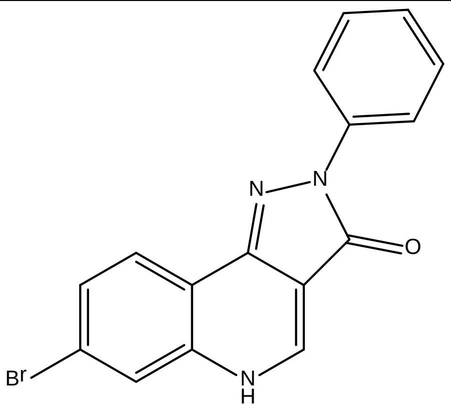 $C_{16}H_{10}BrN_3O$	QH-II-077	0.06	0.08	0.05		0.12	4
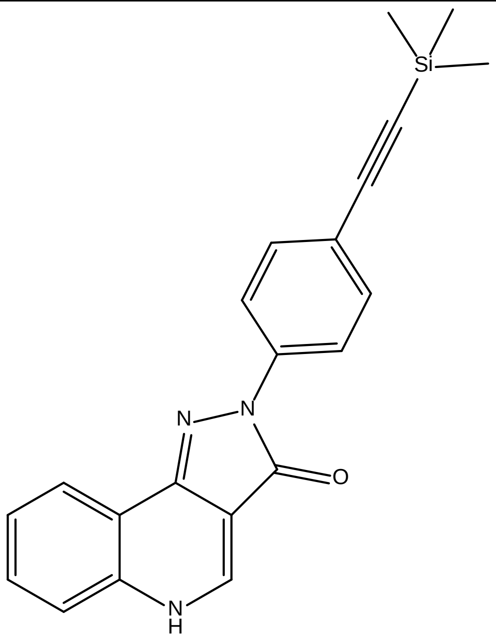 $C_{21}H_{19}N_3OSi$	QH-II-080b	3	3.7	4.7		24	1000
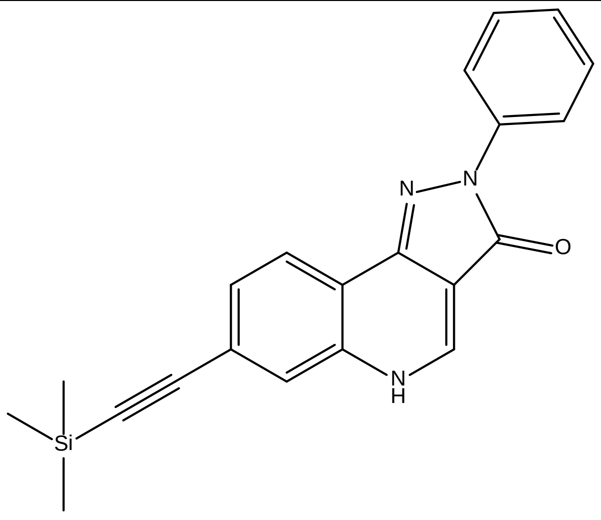 $C_{21}H_{19}N_3OSi$	QH-II-082	1.7	1.8	1.6		6.1	100
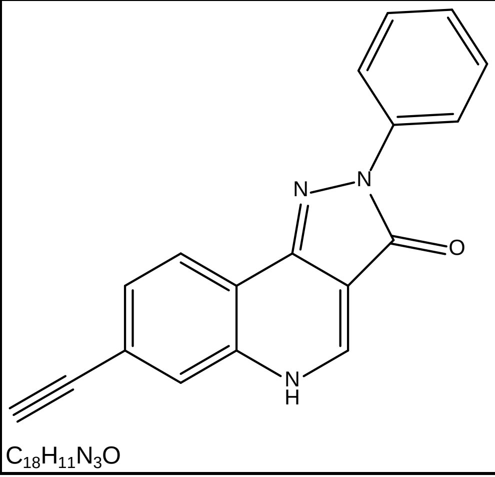 $C_{18}H_{11}N_3O$	QH-II-085	0.08	0.06	0.02		0.08	

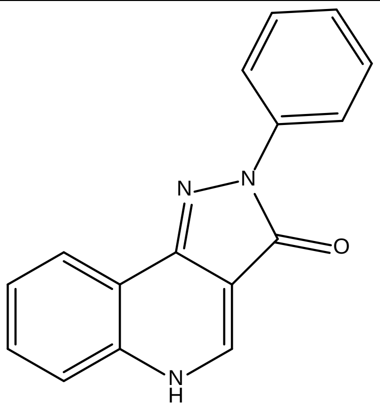 $C_{16}H_{11}N_3O$	QH-II-090(CGS-8216)	0.05	0.08	0.12		0.25	17
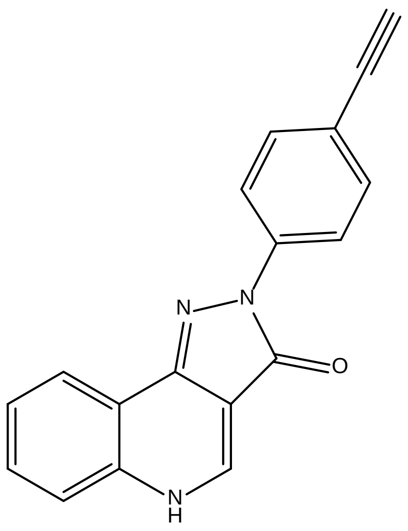 $C_{18}H_{11}N_3O$	QH-II-092	0.07	0.03	0.04		0.17	
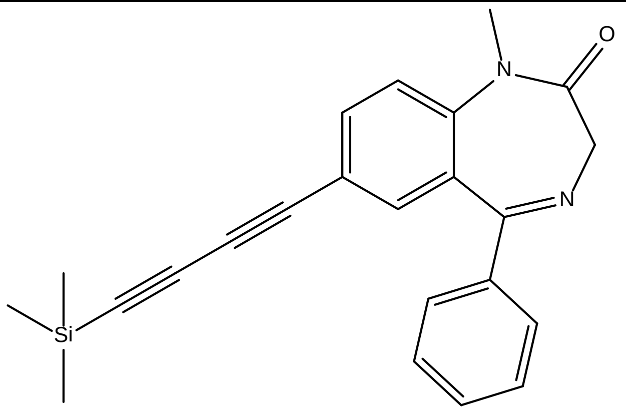 $C_{23}H_{22}N_2OSi$	QH-IV-014	3000	3000	3000		3000	3000
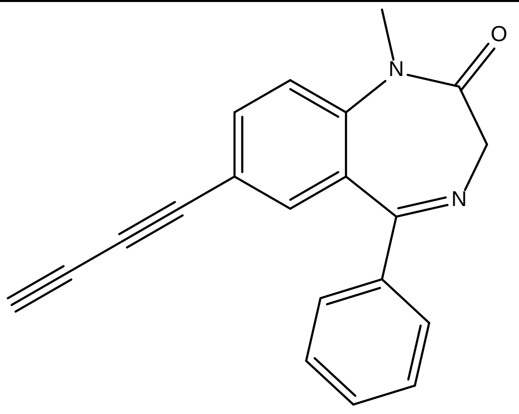 $C_{20}H_{14}N_2O$	QH-IV-016	3000	3000	3000		3000	3000
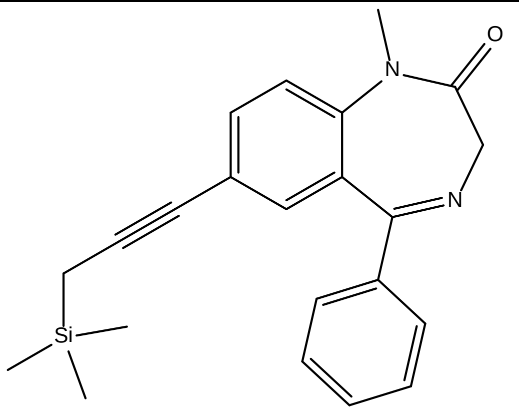 $C_{22}H_{24}N_2OSi$	QH-IV-019	3000	3000	3000		3000	3000
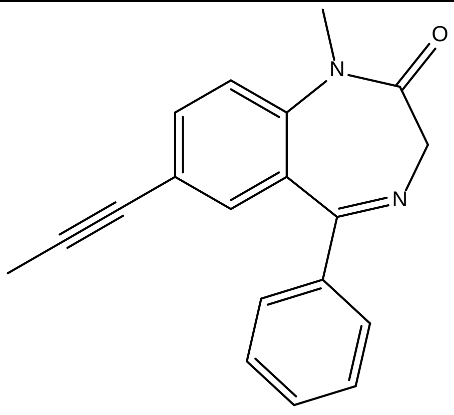 $C_{19}H_{16}N_2O$	QH-IV-021	3000	3000	3000		3000	3000

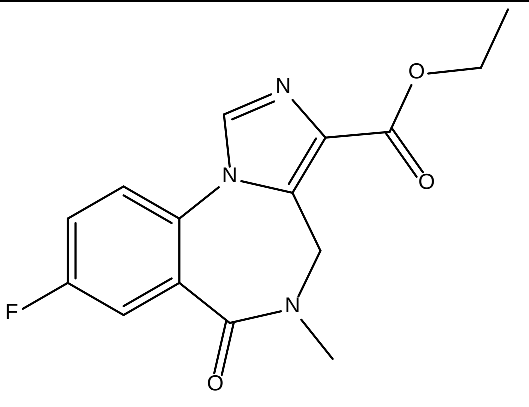 $C_{15}H_{14}FN_3O_3$	Ro15-1788	0.8	0.9	1.05		0.6	148
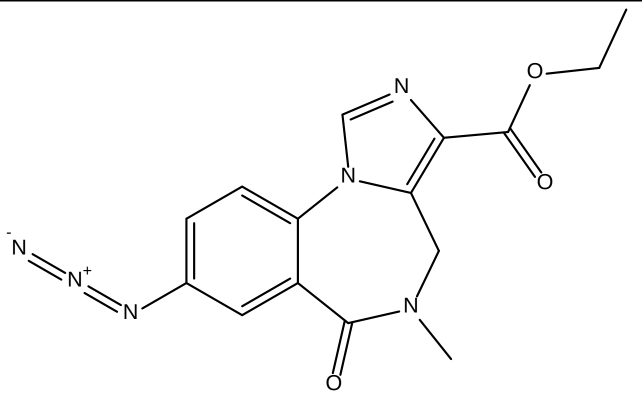 $C_{15}H_{14}N_6O_3$	Ro15-4513	3.3	2.6	2.5		0.26	3.8
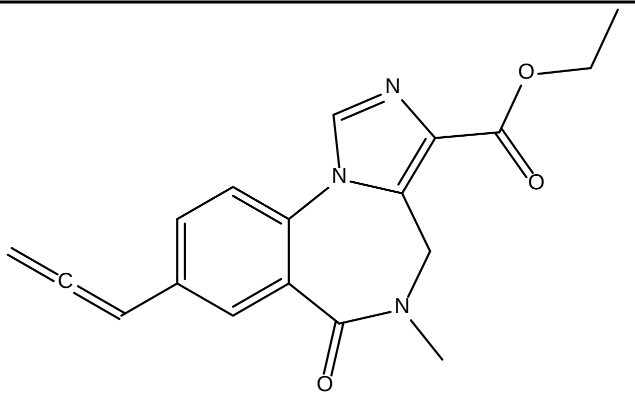 $C_{18}H_{17}N_3O_3$	RY-008	3.75	7.2	4.14		1.11	44.3
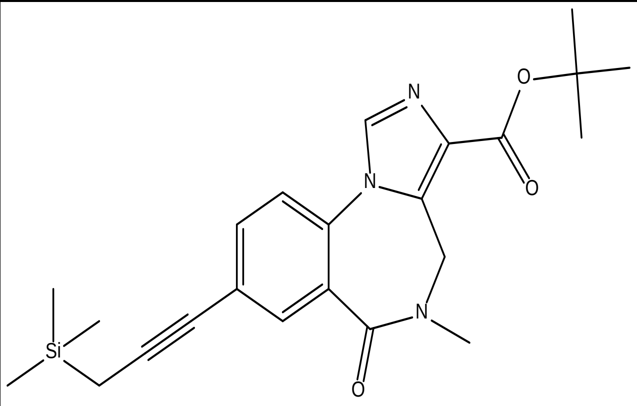 $C_{23}H_{29}N_3O_3Si$	RY-020	275	387	337		23	301
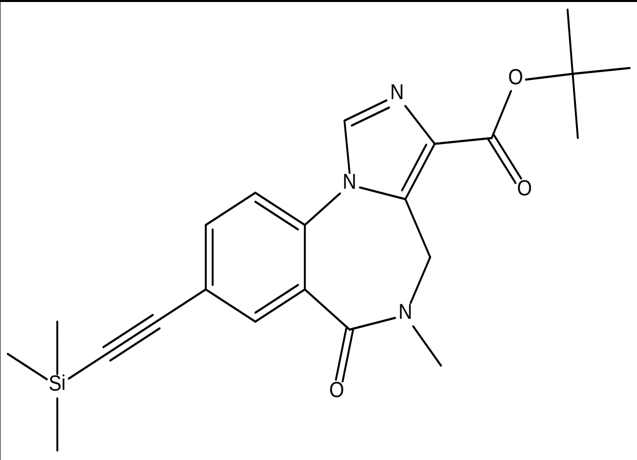 $C_{22}H_{27}N_3O_3Si$	RY-023 C22H27N3O3Si	197	142.6	255	7.8	2.61	58.6
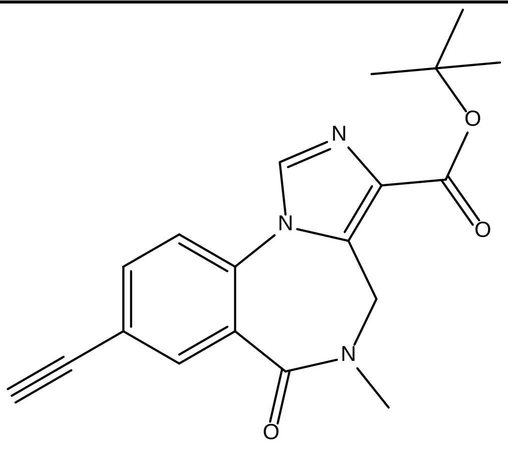 $C_{19}H_{19}N_3O_3$	RY-024 C19H19N3O3	26.9	26.3	18.7		0.4	5.1

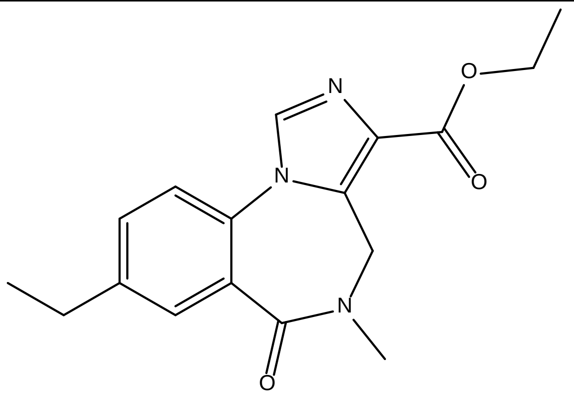 $C_{17}H_{19}N_3O_3$	RY-031 (RY-10)	20.4	27	26.1		1.5	176
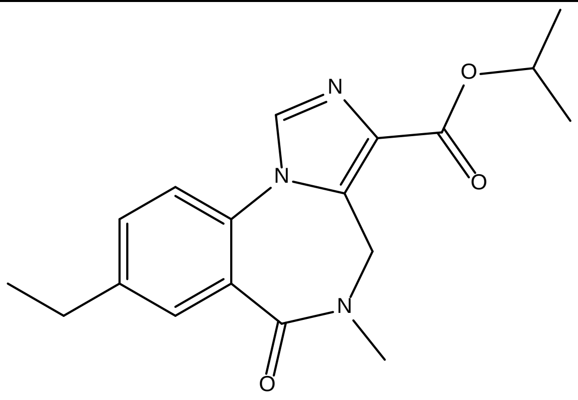 $C_{18}H_{21}N_3O_3$	RY-033	14.8	56	25.3		1.72	22.9
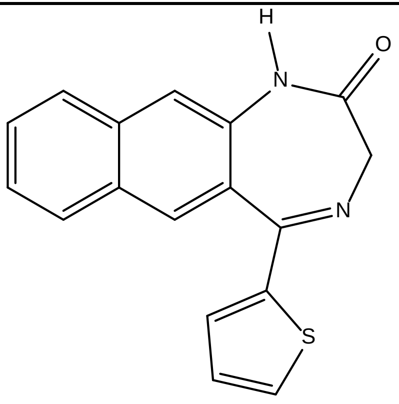 $C_{17}H_{12}N_2OS$	RY-035	980	590	775		92.5	3000
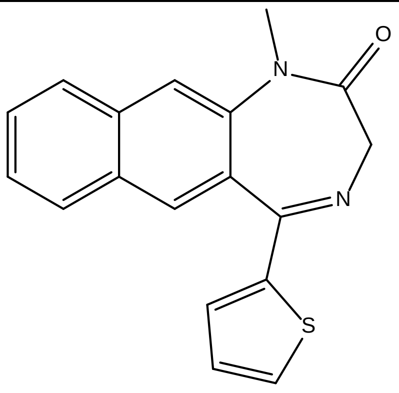 $C_{18}H_{14}N_2OS$	RY-037	300	300	300		300	300
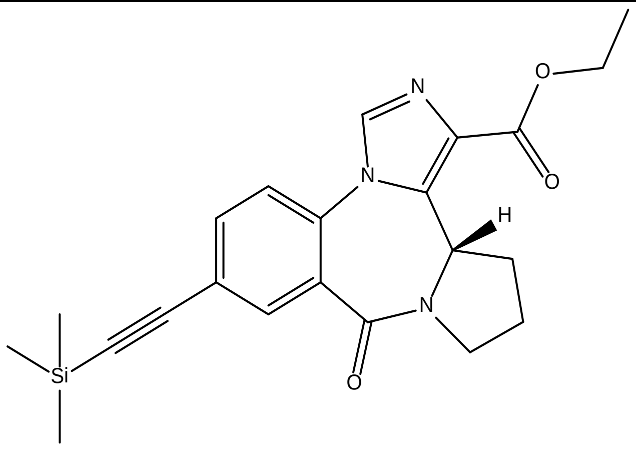 $C_{22}H_{25}N_3O_3Si$	RY-047	200	124	79		4	340
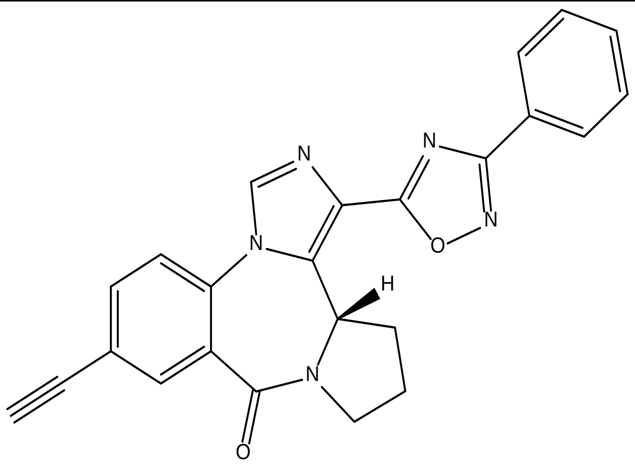 $C_{24}H_{17}N_5O_2$	RY-049						
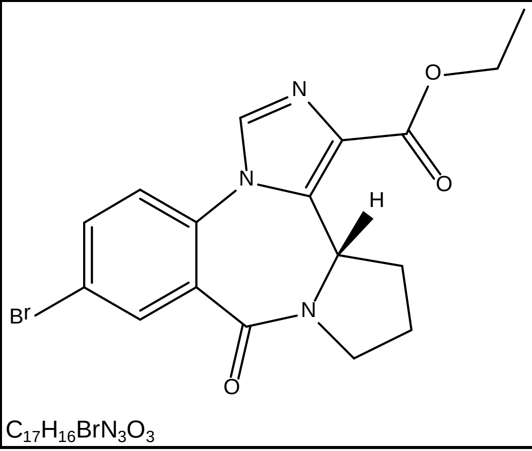 $C_{17}H_{16}BrN_3O_3$	RY-053	49	29	15		1	46

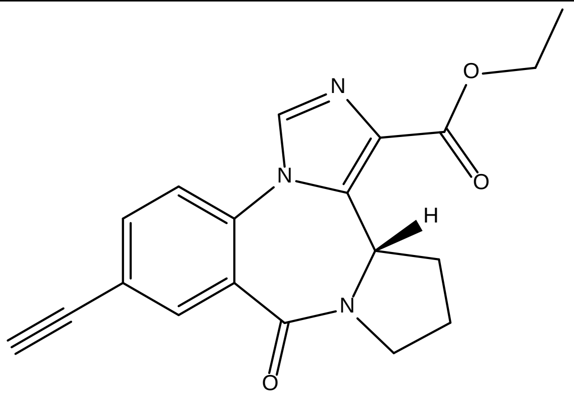 $C_{19}H_{17}N_3O_3$	RY-054	59	44	27		1.3	126
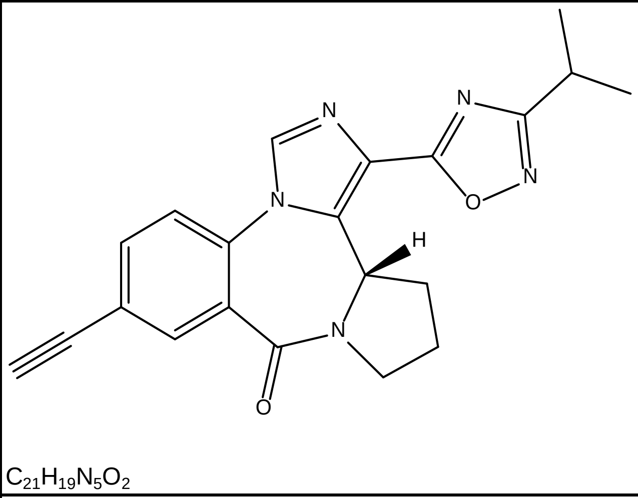 $C_{21}H_{19}N_5O_2$	RY-057	73	85	97		4.8	333
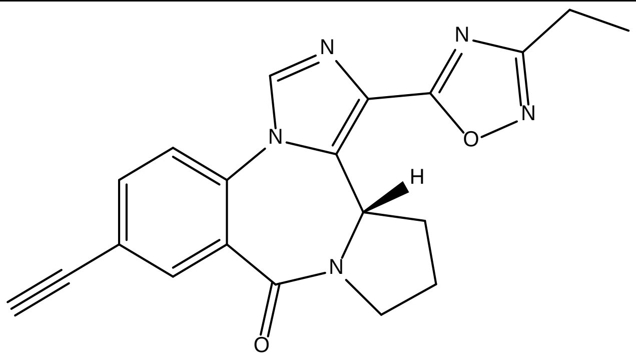 $C_{20}H_{17}N_5O_2$	RY-058	86	40	85		2.4	150
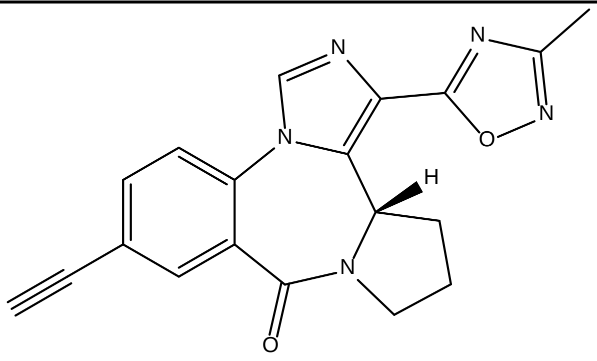 $C_{19}H_{15}N_5O_2$	RY-059	89	70	91		3.7	301
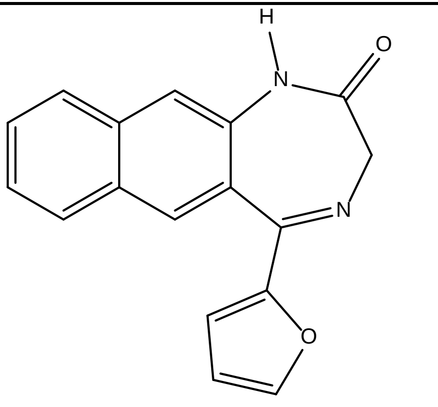 $C_{17}H_{12}N_2O_2$	RY-060	1000	1000	1000		1000	1000
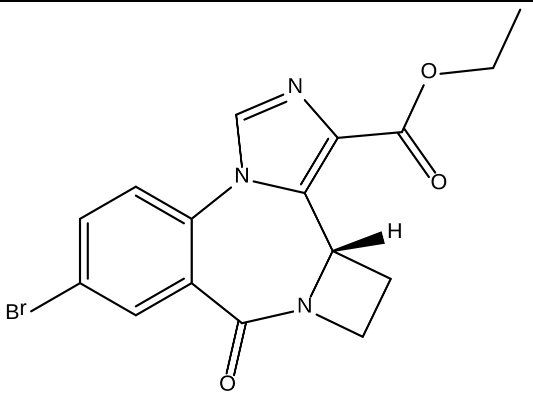 $C_{16}H_{14}BrN_3O_3$	RY-061	17	13	6.7		0.3	31

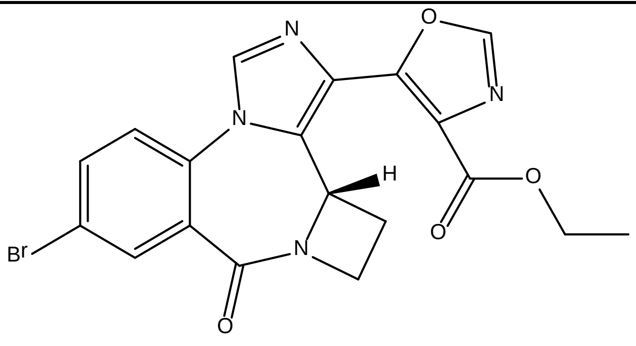 $C_{19}H_{15}BrN_4O_4$	RY-062	1000	1000	500		172	2000
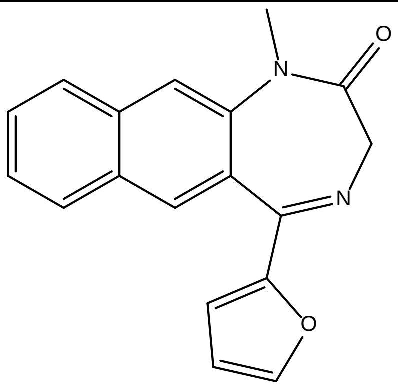 $C_{18}H_{14}N_2O_2$	RY-062	300	300	300		300	300
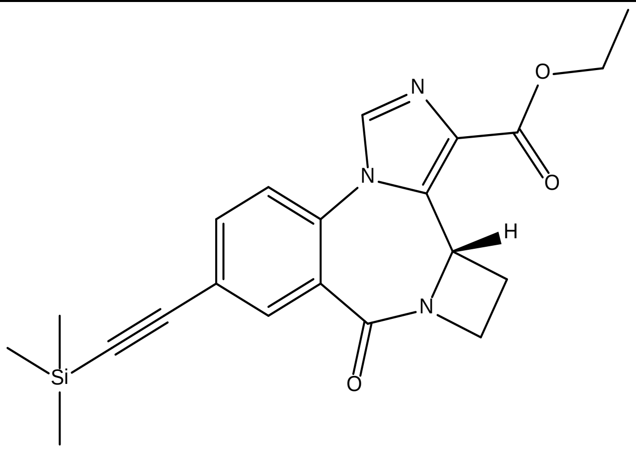 $C_{21}H_{23}N_3O_3Si$	RY-066	83	60	48		2.6	180
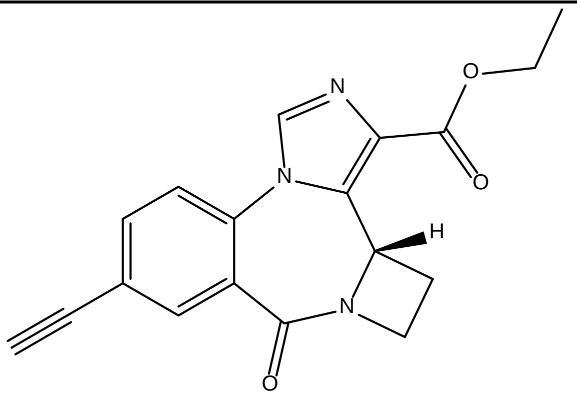 $C_{18}H_{15}N_3O_3$	RY-067	21	12	10		0.37	42
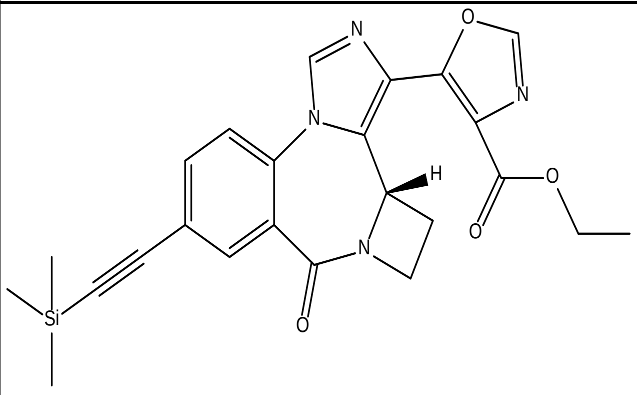 $C_{24}H_{24}N_4O_4Si$	RY-068	500	877	496		37	1000

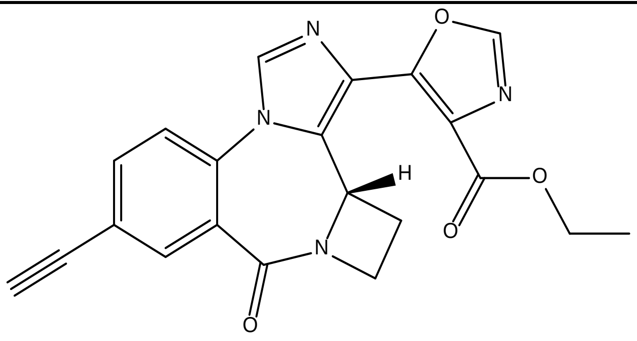 $C_{21}H_{16}N_4O_4$	RY-069	692	622	506		19	1000
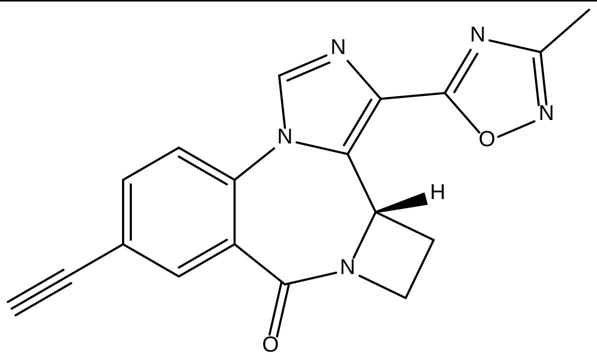 $C_{18}H_{13}N_5O_2$	RY-071	19	56	91		7.2	266
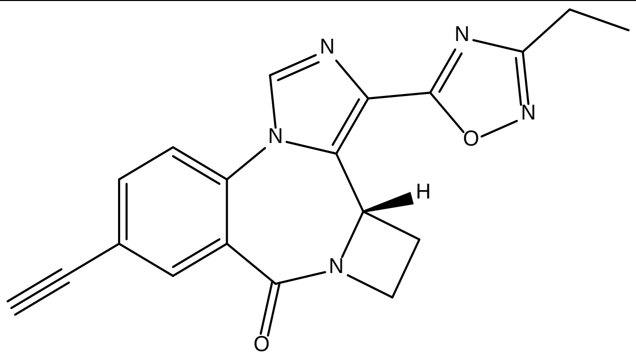 $C_{19}H_{15}N_5O_2$	RY-072	220	150	184		12.7	361
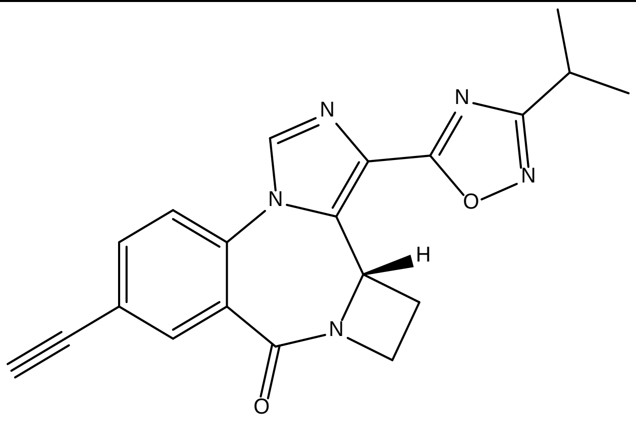 $C_{20}H_{17}N_5O_2$	RY-073	156	88	122		8.5	267
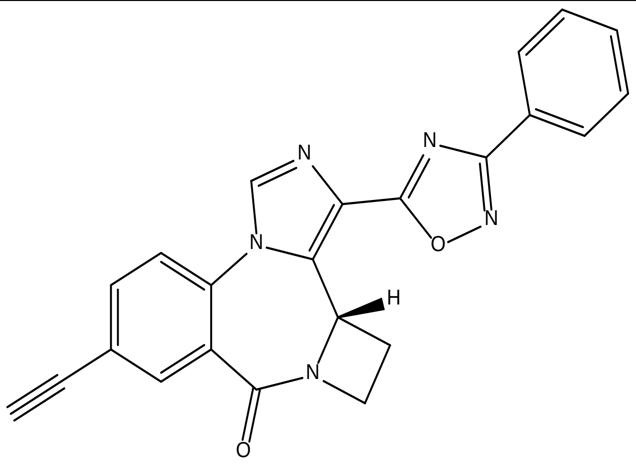 $C_{23}H_{15}N_5O_2$	RY-075						
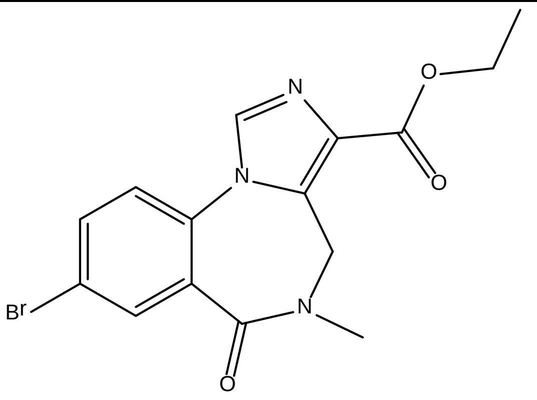 $C_{15}H_{14}BrN_3O_3$	RY-076	26	27	13		0.7	22

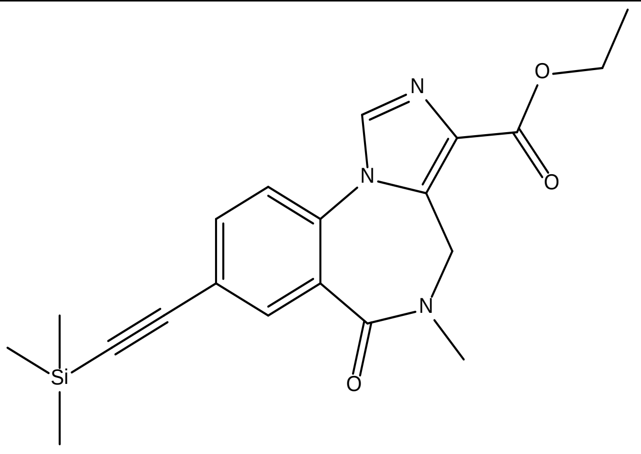 $C_{20}H_{23}N_3O_3Si$	RY-079	121.1	141.9	198.4	159	5	113.7
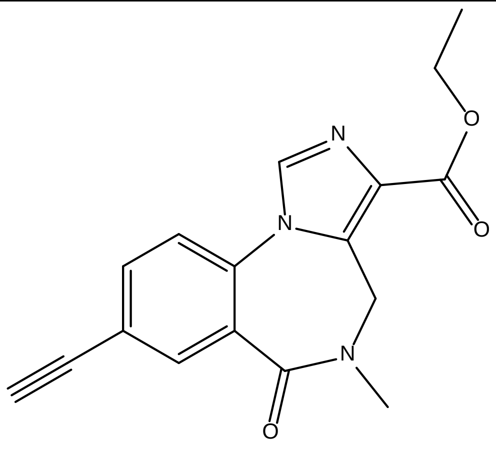 $C_{17}H_{15}N_3O_3$	RY-080 C17H15N3O3	28.4	21.4	25.8	5.3	0.49	28.8
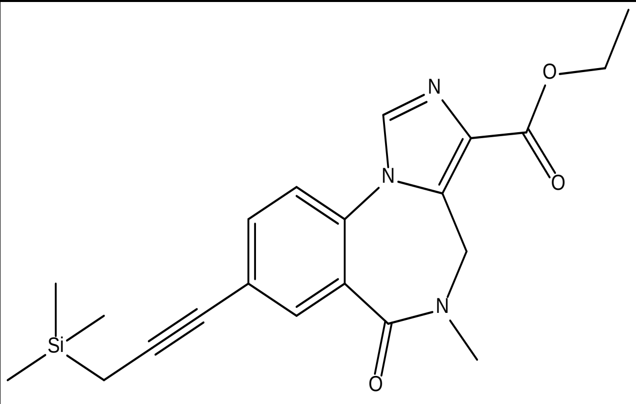 $C_{21}H_{25}N_3O_3Si$	RY-097	300	300	300		300	300
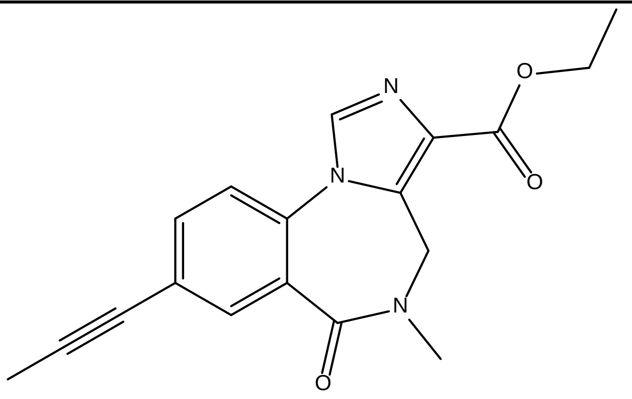 $C_{18}H_{17}N_3O_3$	RY-098	10.1	22.2	16.5		1.68	100
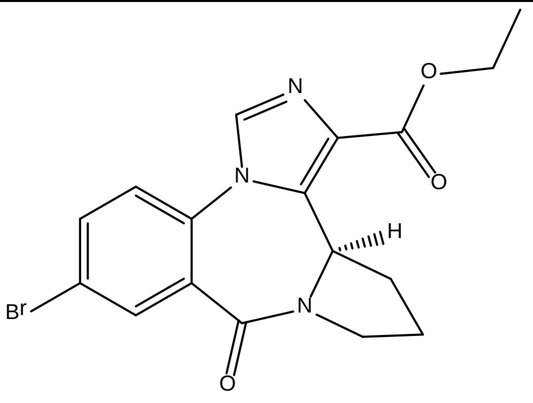 $C_{17}H_{16}BrN_3O_3$	RY-I-26	1000	1000	1000		1000	1000
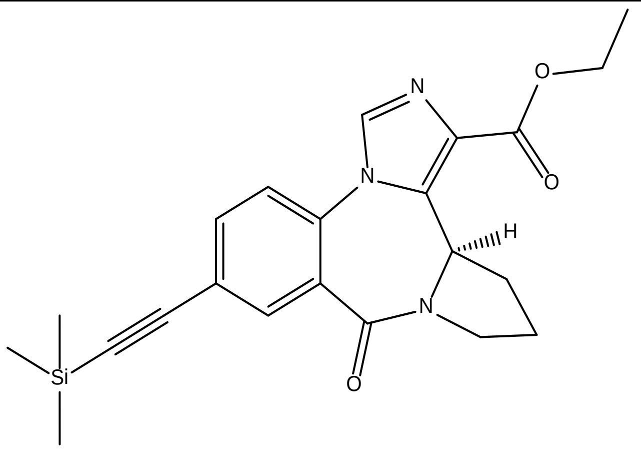 $C_{22}H_{25}N_3O_3Si$	RY-I-27	1000	1000	1000		1000	1000

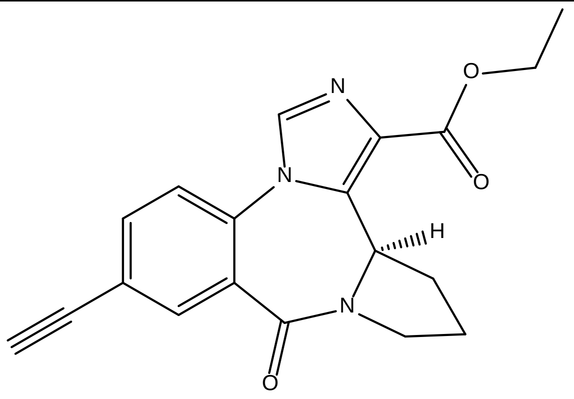 $C_{19}H_{17}N_3O_3$	RY-I-28	283	318	102		7.2	61
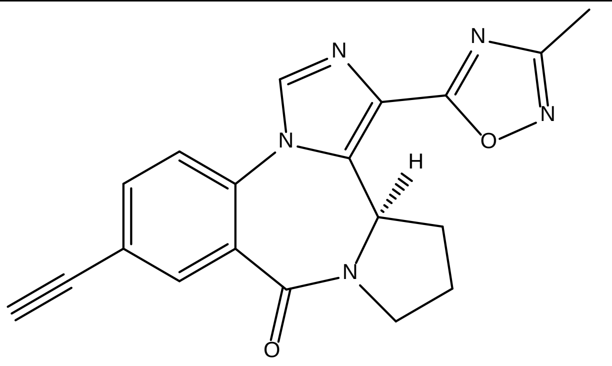 $C_{19}H_{15}N_5O_2$	RY-I-29	1000	1000	1000		157	1000
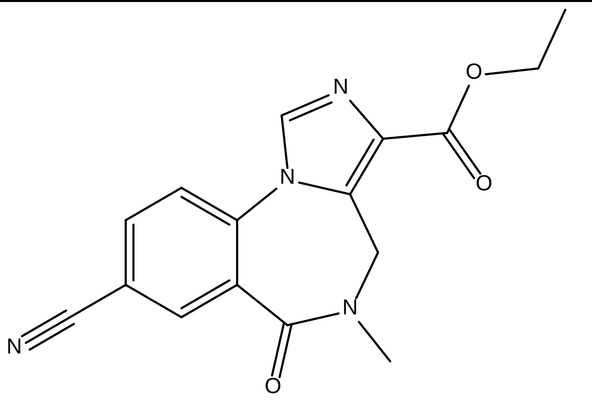 $C_{16}H_{14}N_4O_3$	RY-I-31	10	45	19		6	1000
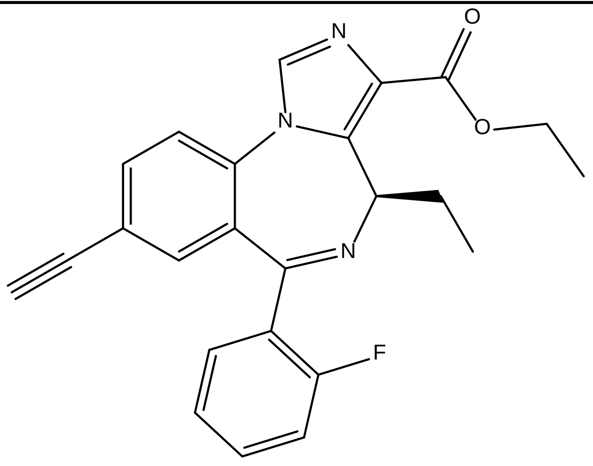 $C_{24}H_{20}FN_3O_2$	SA-01-031	2013	1887			609.3	
$C_{22}H_{18}N_4O_2$	SA-SH-053-2'N-R-CH3	406	73565	3392		16843	
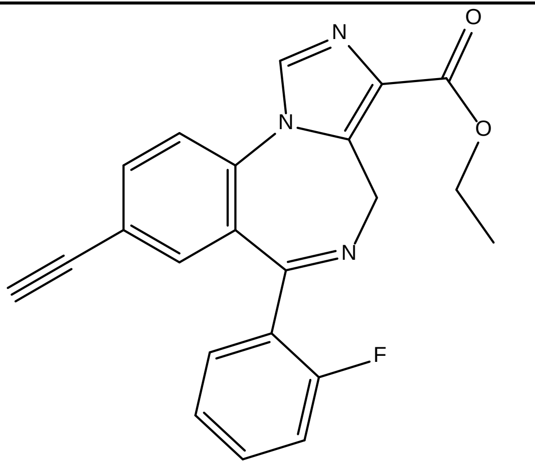 $C_{22}H_{16}FN_3O_2$	SH-053-2'F (JY-XHe-053)	21.99	12.34	34.9		0.671	
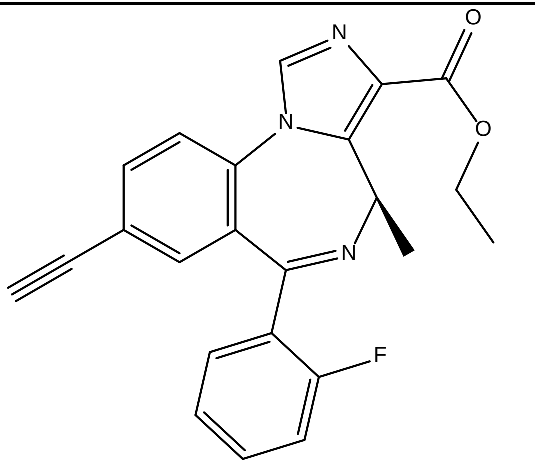 $C_{23}H_{18}FN_3O_2$	SH-053-2'F-R-CH3	759.1	948.2	768.8		95.17	

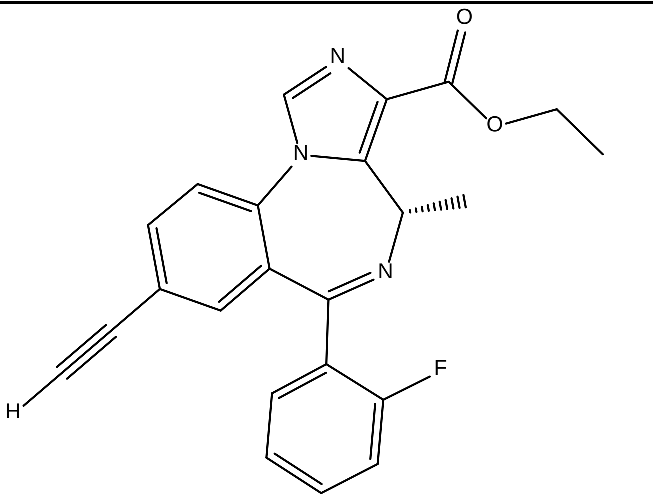 $C_{23}H_{18}FN_3O_2$	SH-053-2'F-S-CH3	468.2	33.27	291.5		19.2	
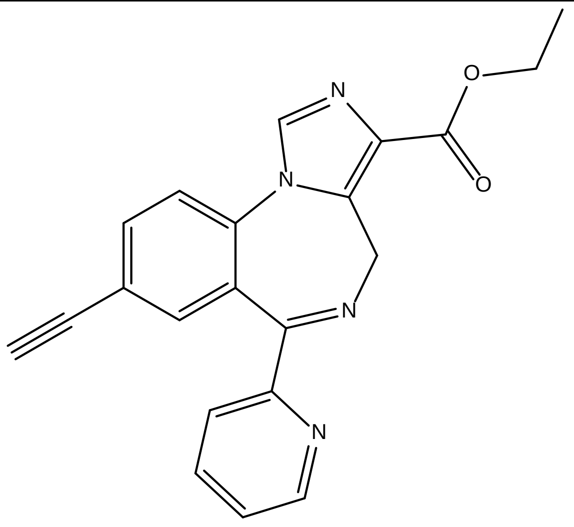 $C_{21}H_{16}N_4O_2$	SH-053-2'N	300	160	527		82	5000
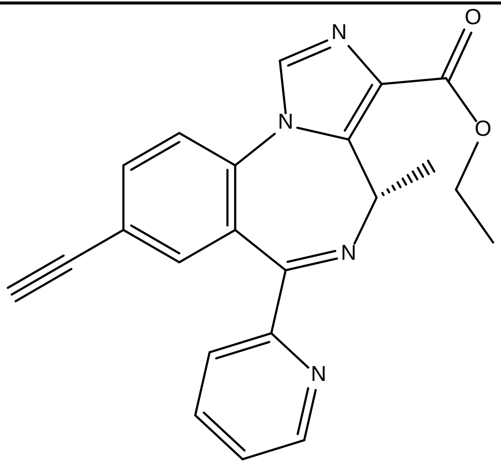 $C_{22}H_{18}N_4O_2$	SH-053-2'N-S-CH3	6951	2331	2655		744.1	
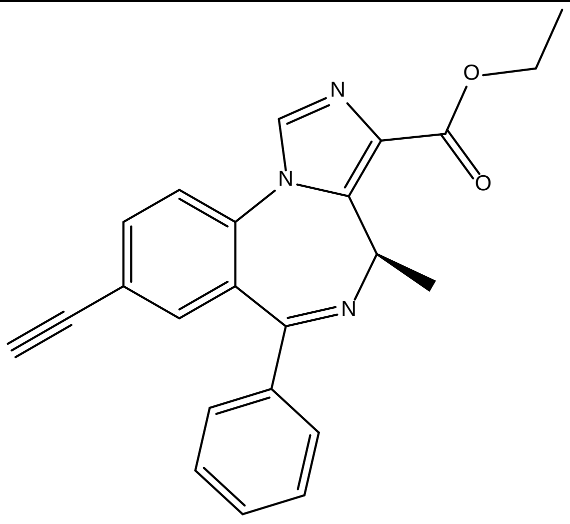 $C_{23}H_{19}N_3O_2$	SH-053-R-CH3	2026	2377	1183		949.1	
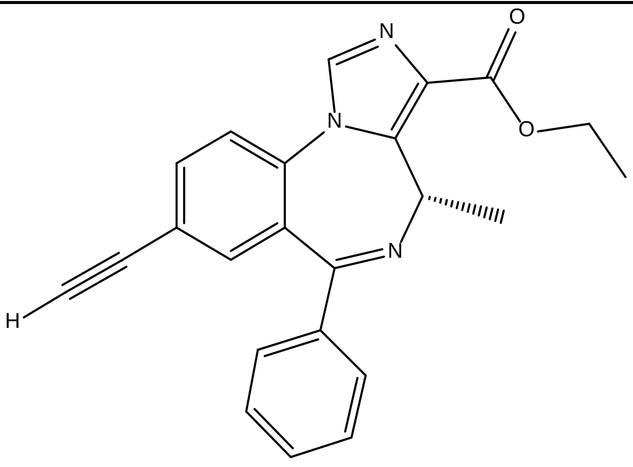 $C_{23}H_{19}N_3O_2$	SH-053-S-CH3	1666	1263	1249		206.4	
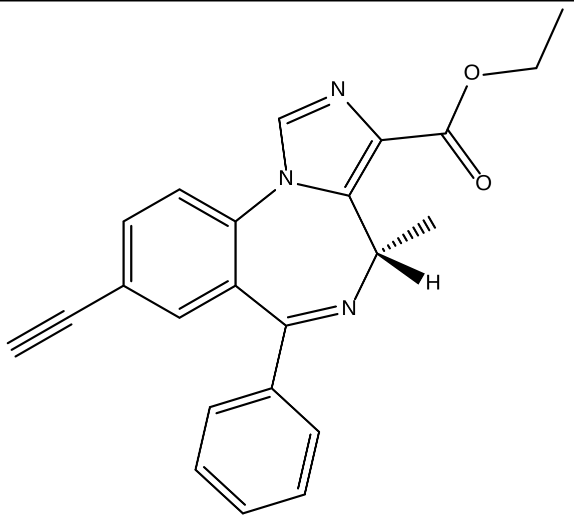 $C_{23}H_{19}N_3O_2$	SH-053-S-CH3	5000	551	540.1		201	5000

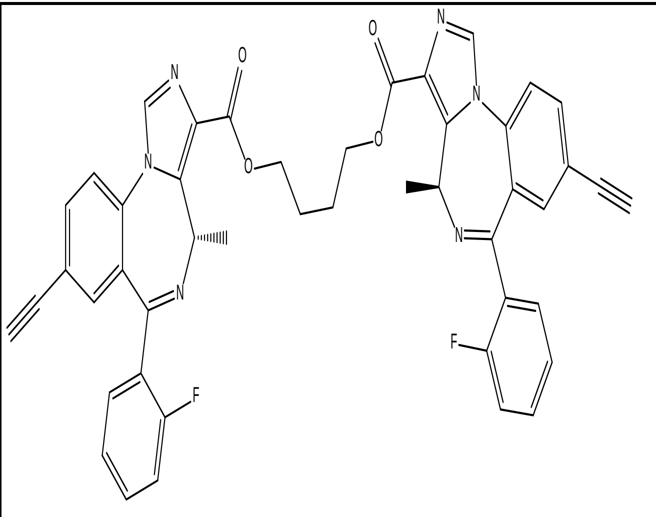 $C_{46}H_{34}F_2N_6O_4$	SH-222A	2058	292.3			51.24	
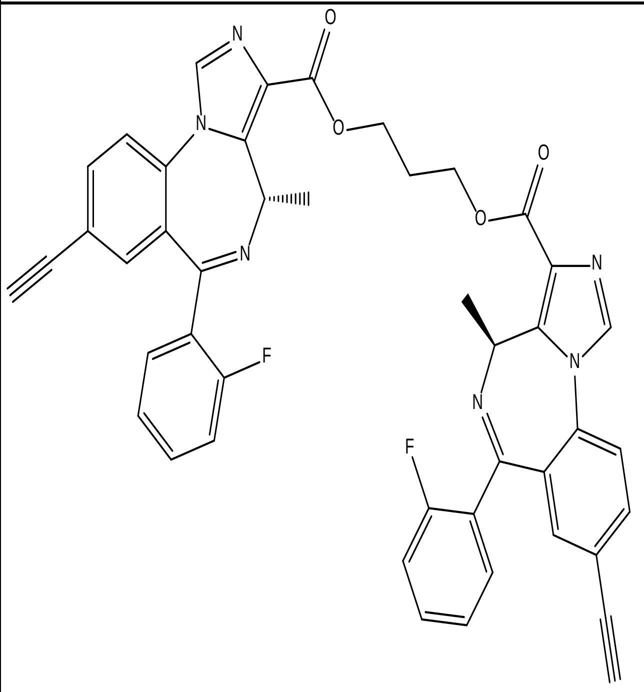 $C_{45}H_{32}F_2N_6O_4$	SH-223A	1231	2008	3609		261.3	
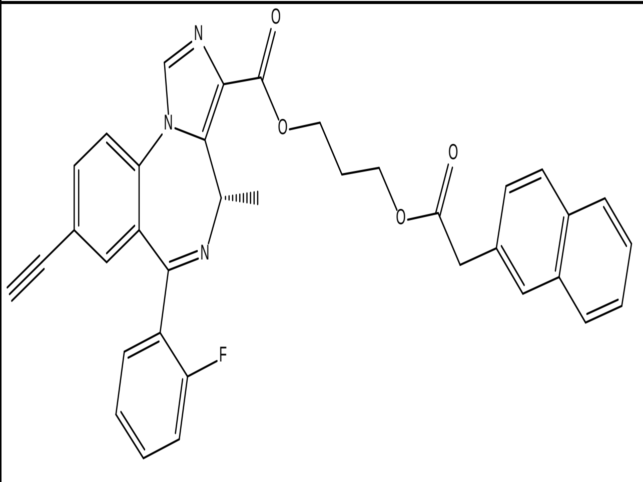 $C_{36}H_{28}FN_3O_4$	SH-223B	4117	4785	10661		911	
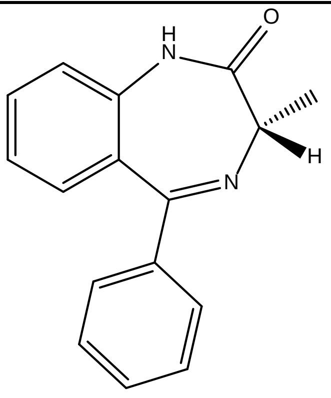 $C_{16}H_{14}N_2O$	SH-I-029B	489	576.5	706.1		208.8	
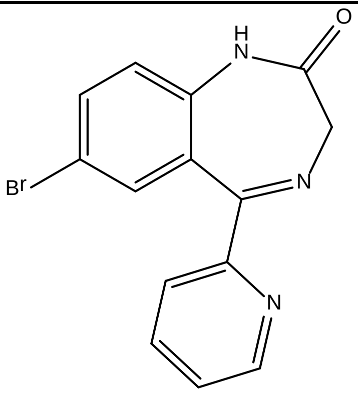 $C_{14}H_{10}BrN_3O$	SH-I-02B	29.82	1315	18		74.05	
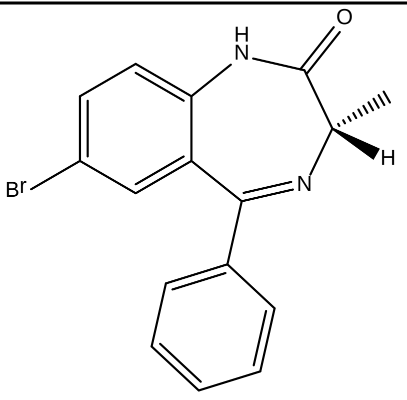 $C_{16}H_{13}BrN_2O$	SH-I-030	14.42	11.04	19.09		1.89	

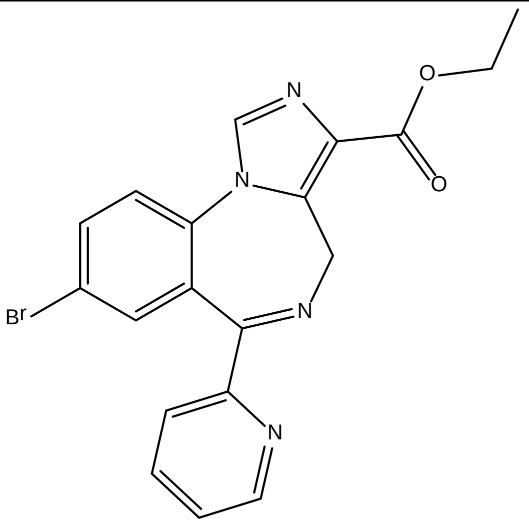 $C_{19}H_{15}BrN_4O_2$	SH-I-031A	84.05	141.3	150.5		151.2	
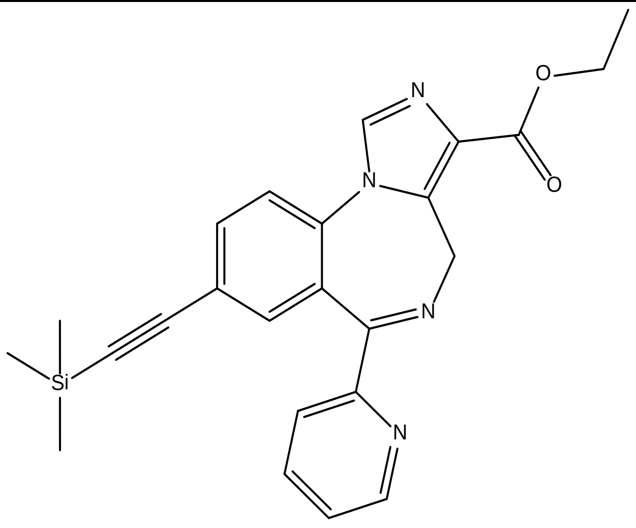 $C_{24}H_{24}N_4O_2Si$	SH-I-031B	981.9	1004	2046		356.2	
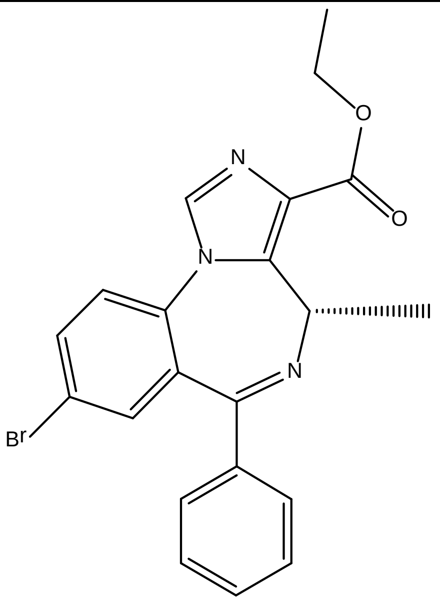 $C_{21}H_{18}BrN_3O_2$	SH-I-034(S)	793.4	746.8	544.6		1564	
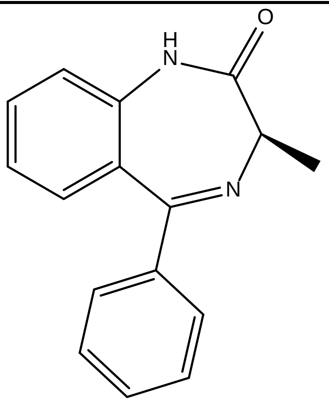 $C_{16}H_{14}N_2O$	SH-I-035A	7389	6049	16811		15793	
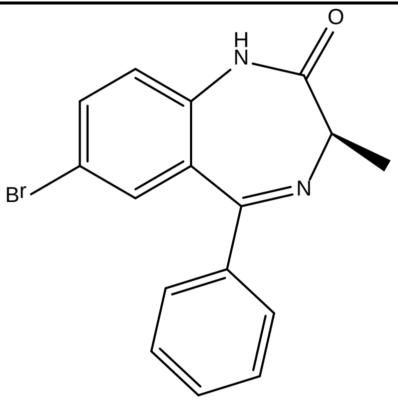 $C_{16}H_{13}BrN_2O$	SH-I-035B	7439	11084	3021		4043	
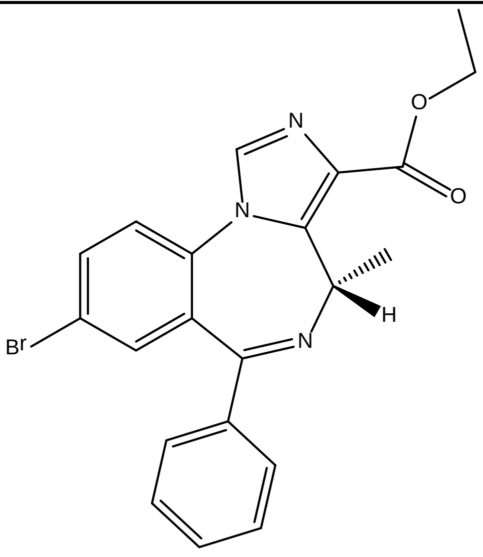 $C_{21}H_{18}BrN_3O_2$	SH-I-036	587	587.9	280.2		150.8	

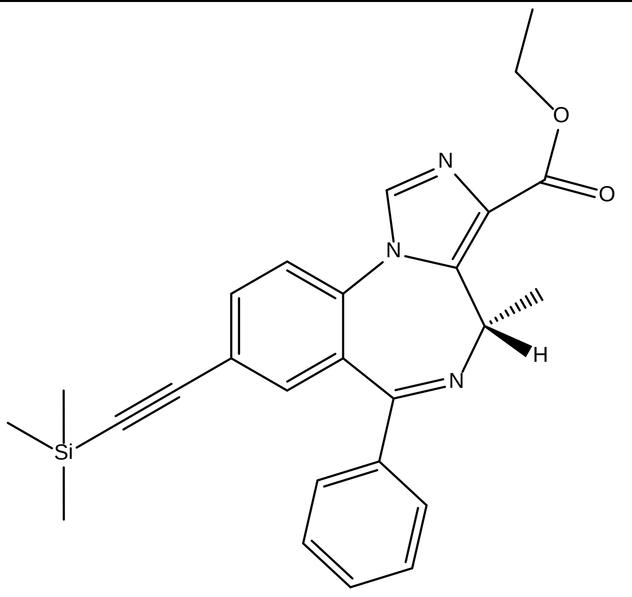 $C_{26}H_{27}N_3O_2Si$	SH-I-038						
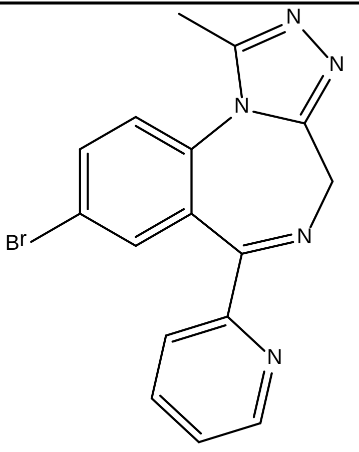 $C_{16}H_{12}BrN_5$	SH-I-04	7.3	6.1	5.1		7.7	
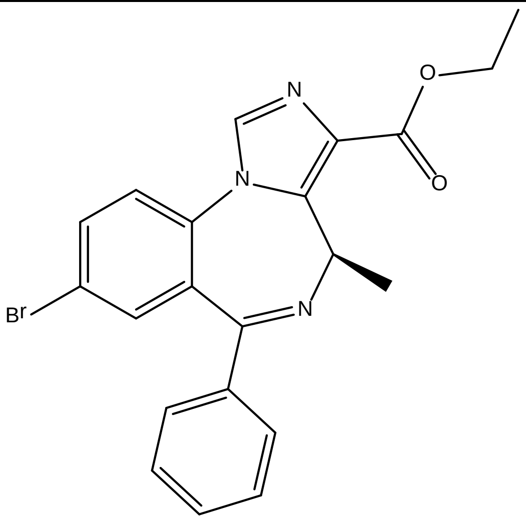 $C_{21}H_{18}BrN_3O_2$	SH-I-040 R	774.4	334.1	584		355.9	
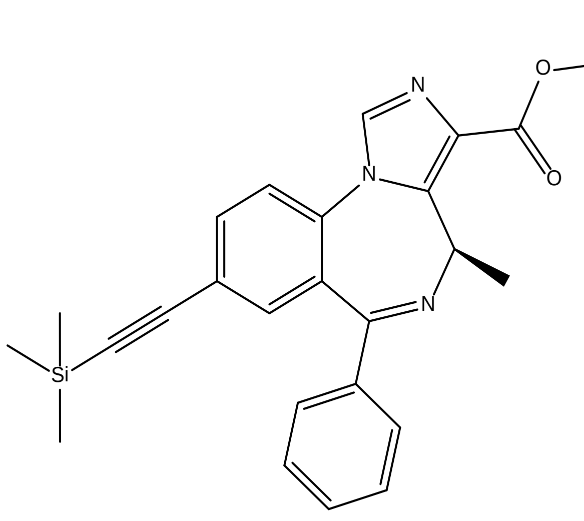 $C_{26}H_{27}N_3O_2Si$	SH-I-041						
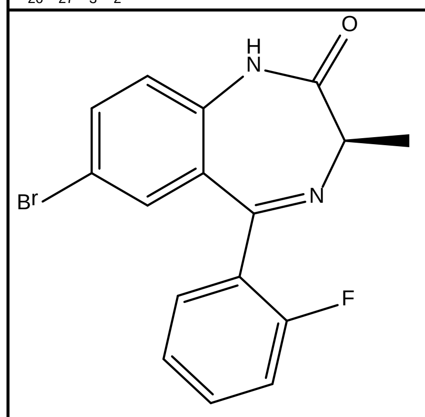 $C_{16}H_{12}BrFN_2O$	SH-I-044	5633	2034	6658		3521	
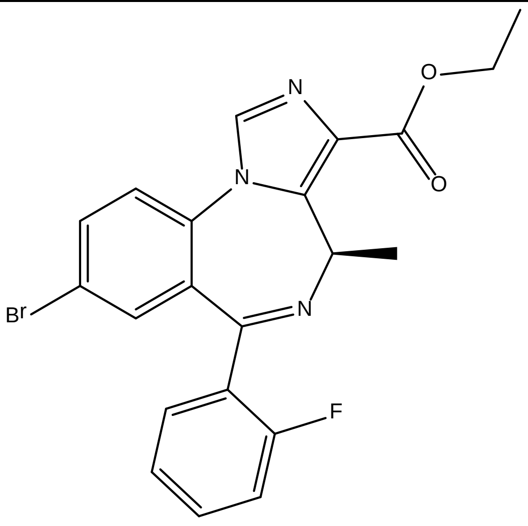 $C_{21}H_{17}BrFN_3O_2$	SH-I-047	1710	17.52	1222		1519	

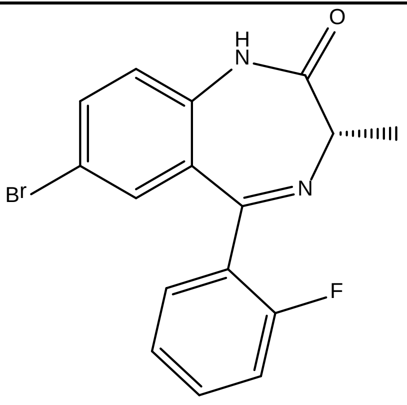 $C_{16}H_{12}BrFN_2O$	SH-I-048A	0.77	0.17	0.38		0.11	
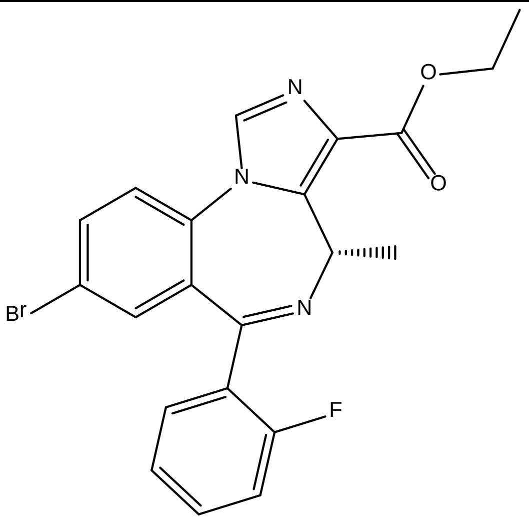 $C_{21}H_{17}BrFN_3O_2$	SH-I-048B						
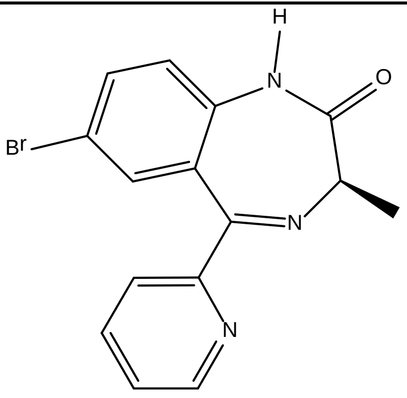 $C_{15}H_{12}BrN_3O$	SH-I-049R						
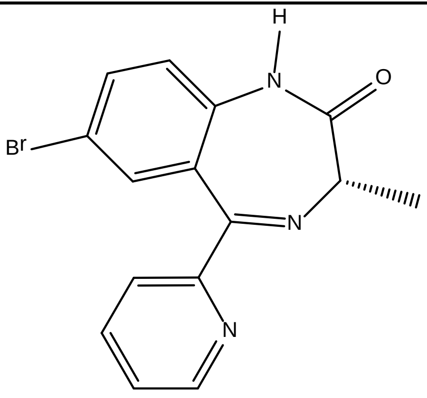 $C_{15}H_{12}BrN_3O$	SH-I-049S	155.6	137.2	88.5		11094	
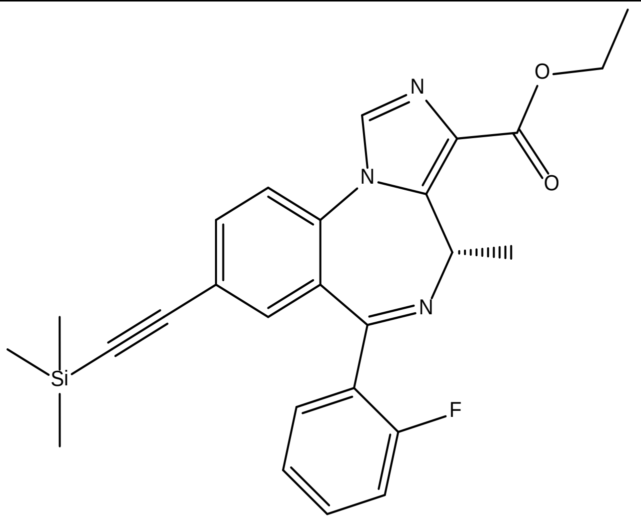 $C_{26}H_{26}FN_3O_2Si$	SH-I-055	5109	1638	1394		223.4	
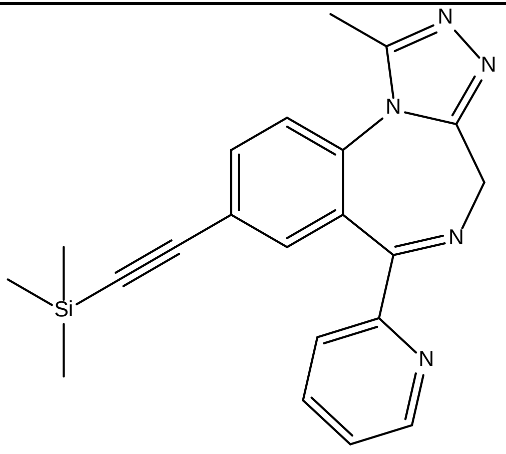 $C_{21}H_{21}N_5Si$	SH-I-06						

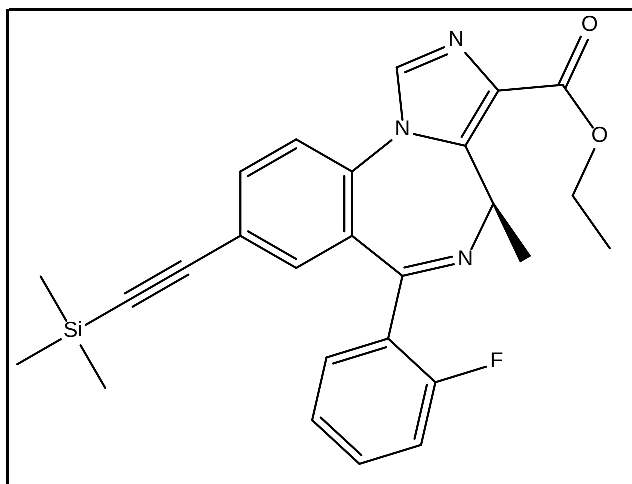 $C_{26}H_{26}FN_3O_2Si$	SH-I-064	4249	3970	3412		1215	
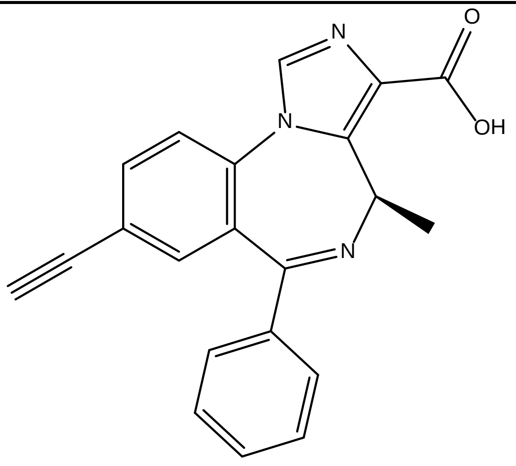 $C_{21}H_{15}N_3O_2$	SH-I-067						
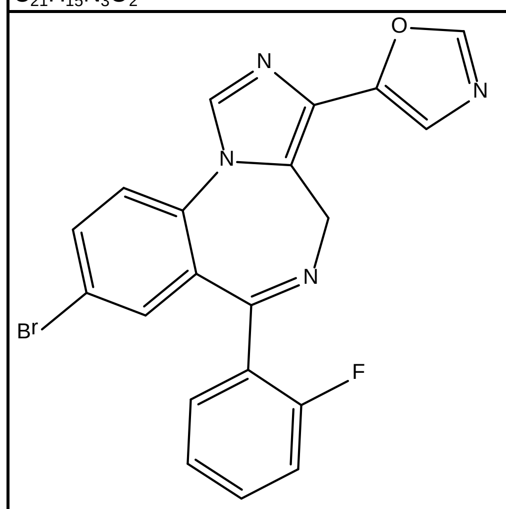 $C_{20}H_{12}BrFN_4O$	SH-I-085	11.08	4.866	13.75		0.24	
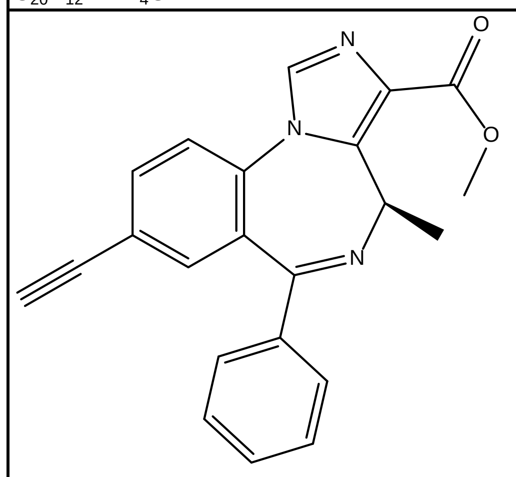 $C_{22}H_{17}N_3O_2$	SH-I-75	1487	989.9	773		0.1825	
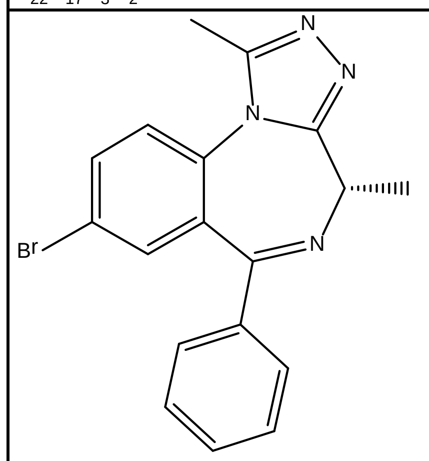 $C_{18}H_{15}BrN_4$	SH-I-89S	12.78	8.562	8.145		3.23	

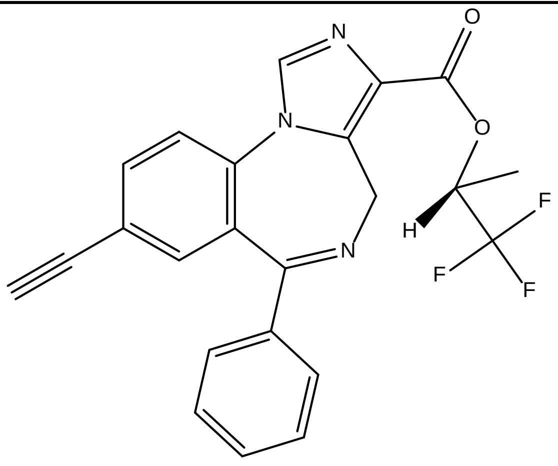 $C_{23}H_{16}F_3N_3O_2$	SH-I-R66	219.5	169.1	87.01		47.97	
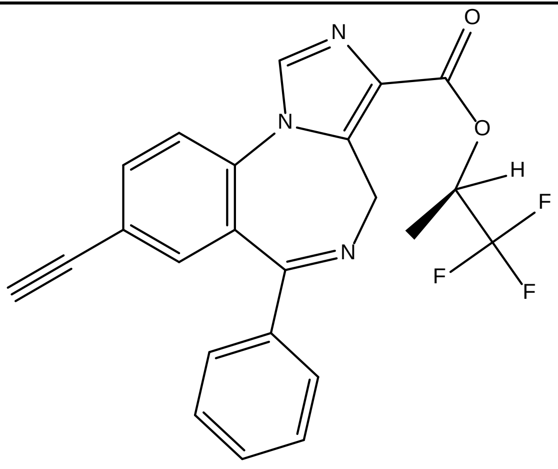 $C_{23}H_{16}F_3N_3O_2$	SH-I-S66	22.93	30.36	55.26		0.69	
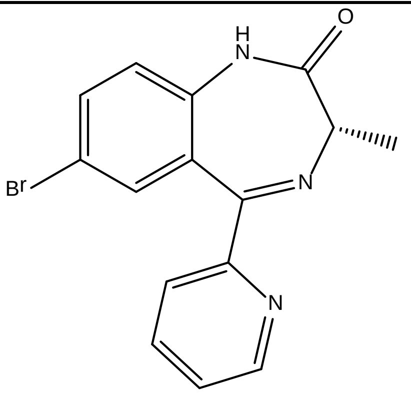 $C_{15}H_{12}BrN_3O$	SH-O53-2'N-S-CH3						
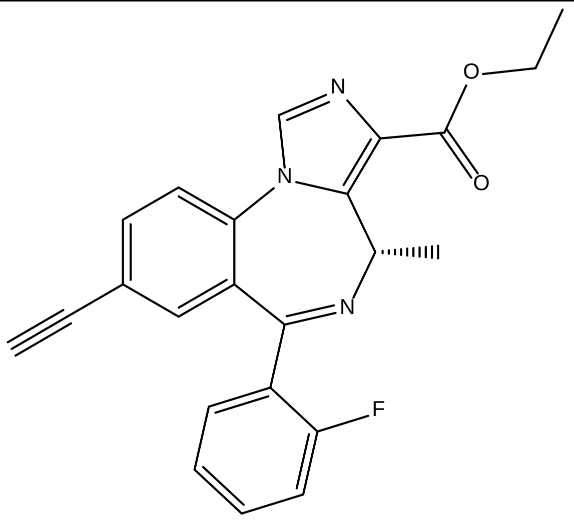 $C_{23}H_{18}FN_3O_2$	SH-O53-S-CH3-2'F	350	141	1237		16	5000
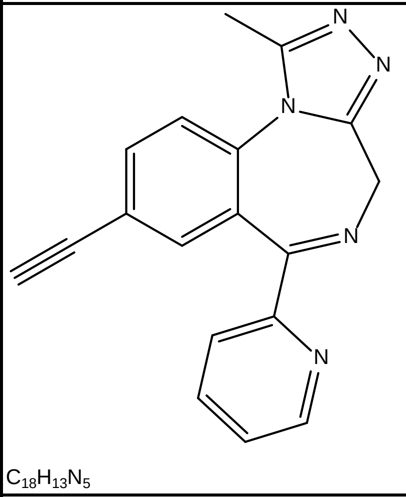 $C_{18}H_{13}N_5$	SH-TRI-108 ANX4	143.9	81.29	103.9		45.64	
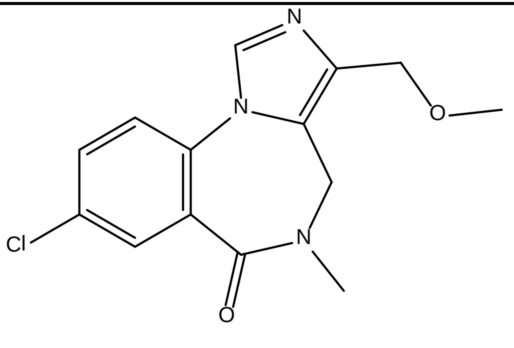 $C_{14}H_{14}ClN_3O_2$	SH-TSC-1(PWZ-029)	362.4	180.3	328.2		6.185	

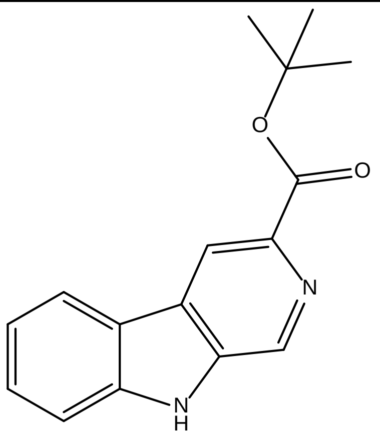 $C_{16}H_{16}N_2O_2$	SH-TSC-2(BCCT)	0.03	0.0419	0.035		69.32	
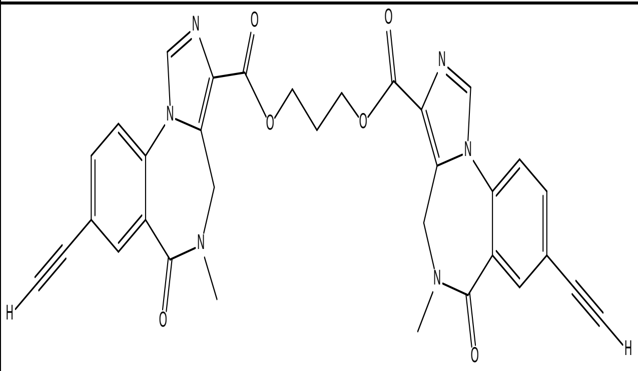 $C_{33}H_{26}N_6O_6$	SH-TSC-3(XLI-093)	1295	744.9	1814		33.99	
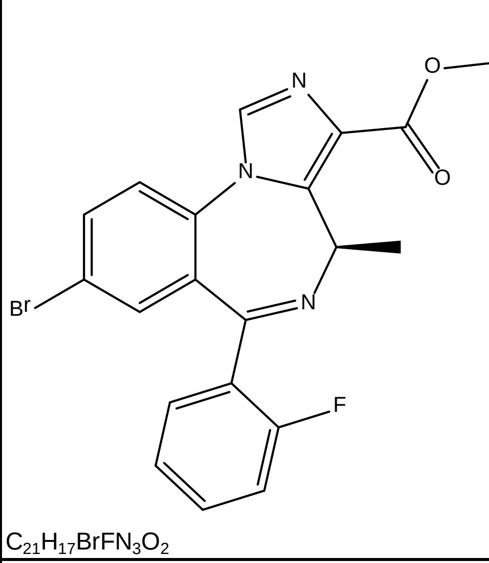 $C_{21}H_{17}BrFN_3O_2$	SH-TSC-5(SH-I-047)	356.7	169.4	285		75.31	
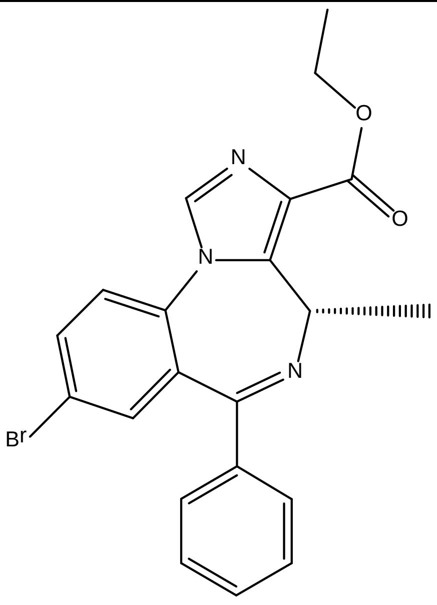 $C_{21}H_{18}BrN_3O_2$	SH-TSC-6(SH-I-034)	913.2	283	300		436.8	
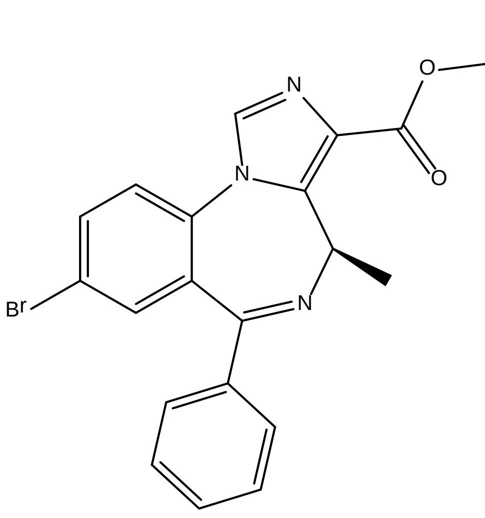 $C_{21}H_{18}BrN_3O_2$	SH-TSC-7(SH-I-040)	709	1997	1256		727.4	
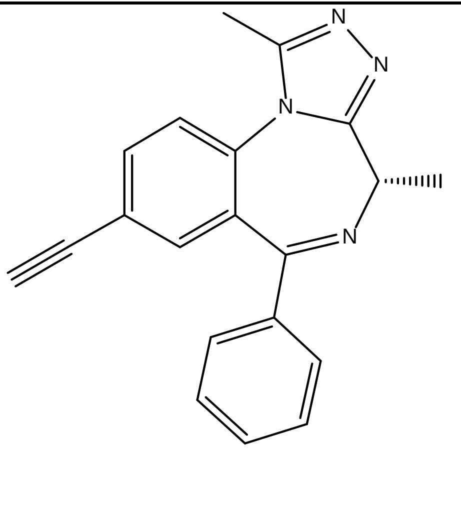 $C_{20}H_{16}N_4$	SH-TS-CH3	107.2	50.09	20.95		8.068	

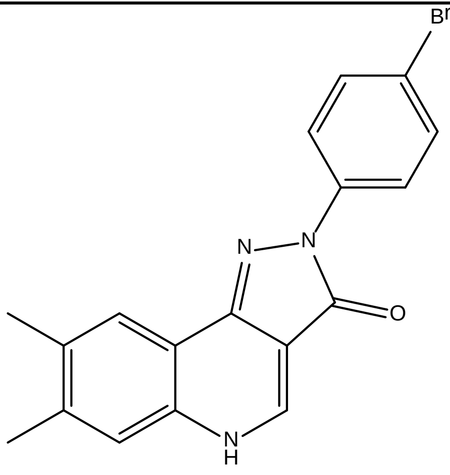 $C_{18}H_{14}BrN_3O$	SHU-1-07	108	260	175	940	155	2535
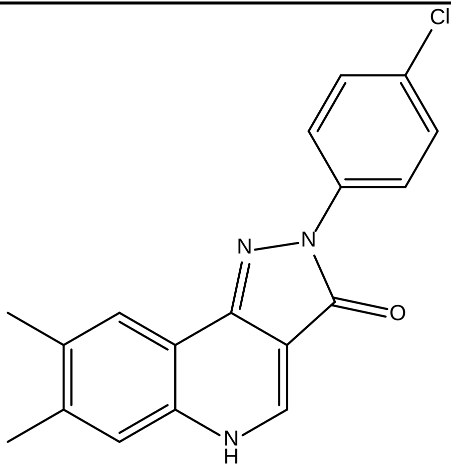 $C_{18}H_{14}ClN_3O$	SHU-1-15	81	63	86	2175	174	2167
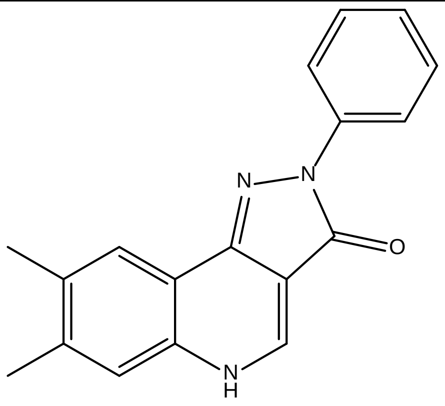 $C_{18}H_{15}N_3O$	SHU-1-19	4	12	7	48	14	84
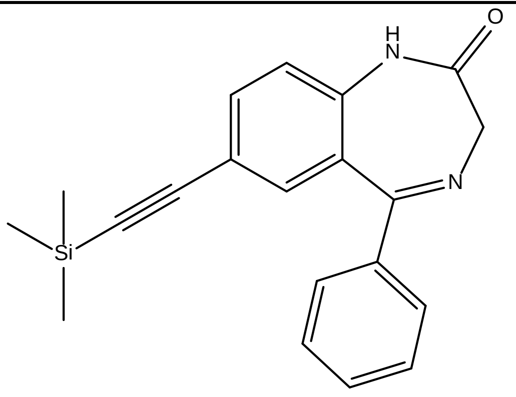 $C_{20}H_{20}N_2OSi$	SHU-221 (XLI-250)	492	439	339	3000	118	3000
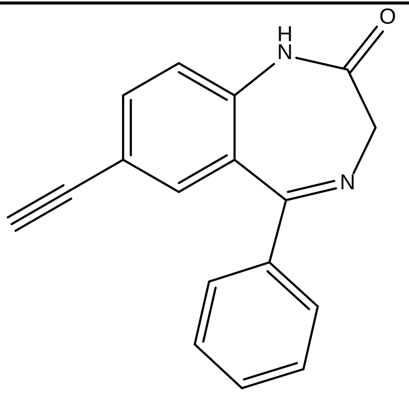 $C_{17}H_{12}N_2O$	SHU-221-1	66	41	43	3000	9	3000
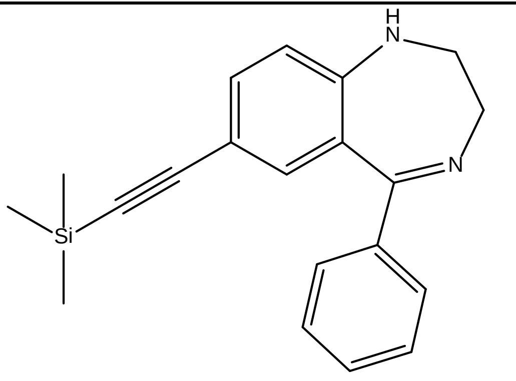 $C_{20}H_{22}N_2Si$	SHU-223	1000	1000	1000	1000	1000	1000

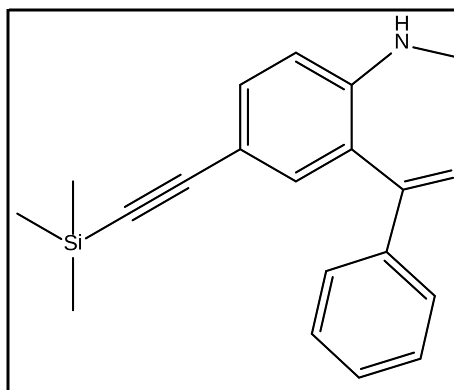<br>$C_{20}H_{22}N_2Si$	SHU-224	1000	1000	1000	1000	1000	1000
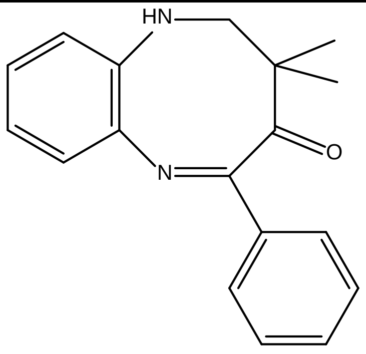<br>$C_{18}H_{18}N_2O$	SHU-525	667	667	667	667	667	429
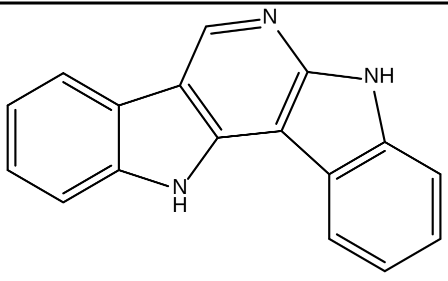<br>$C_{17}H_{11}N_3$	SHU-528	667	667	667	667	667	429
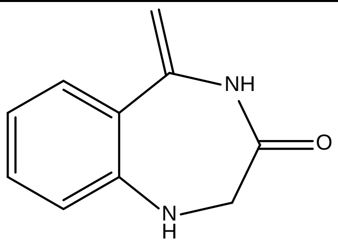<br>$C_{10}H_{10}N_2O$	SHU-530	667	667	667	667	667	429
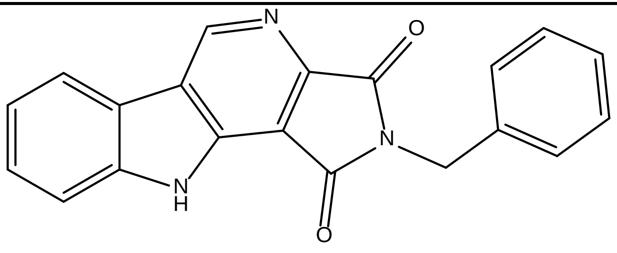<br>$C_{20}H_{13}N_3O_2$	SHU-537	333	333	333	333	667	429
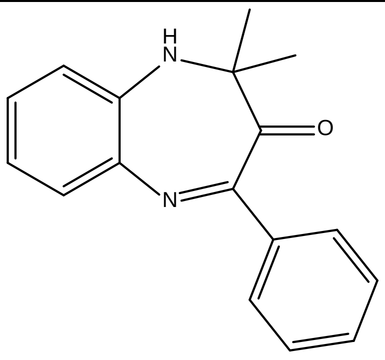<br>$C_{17}H_{16}N_2O$	SHU-547	667	667	667	667	667	429
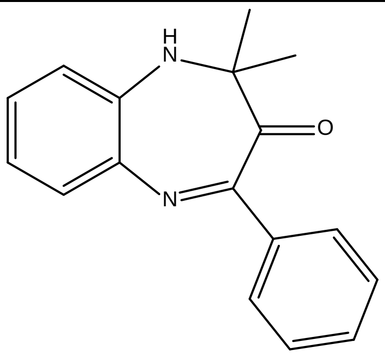<br>$C_{17}H_{16}N_2O$	SHU-550	333	333	333	333	333	429

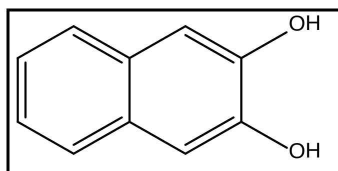 $C_{10}H_8O_2$	SHU-III-032	1000	1000	1000	1000	1000	1000
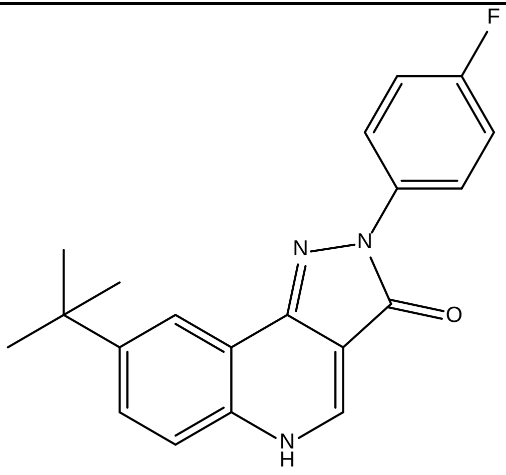 $C_{20}H_{18}FN_3O$	SHU-III-24	1000	1000	1000	1000	1000	1000
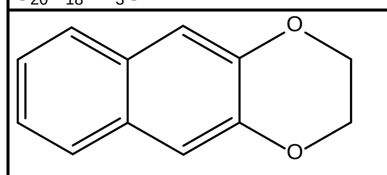 $C_{12}H_{10}O_2$	SHU-III-32	1000	1000	1000	1000	1000	1000
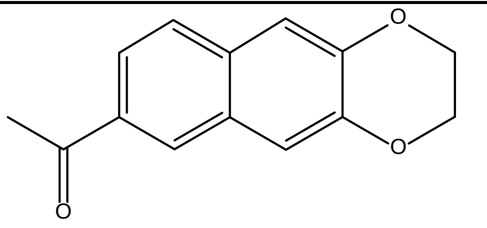 $C_{14}H_{12}O_3$	SHU-III-33	1000	1000	1000	1000	1000	1000
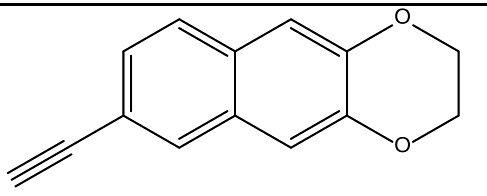 $C_{14}H_{10}O_2$	SHU-III-34	1000	1000	1000	1000	1000	1000
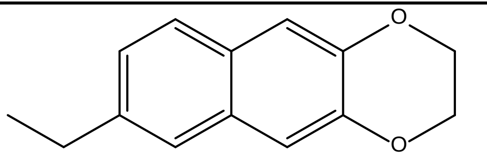 $C_{14}H_{14}O_2$	SHU-III-36	1000	1000	1000	1000	1000	1000
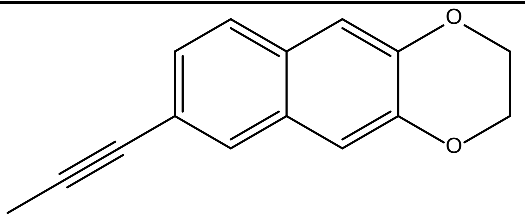 $C_{15}H_{12}O_2$	SHU-III-37	1000	1000	1000	1000	1000	1000

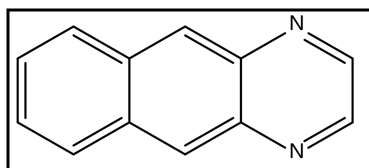 $C_{12}H_8N_2$	SHU-III-40	1000	1000	1000	1000	1000	1000
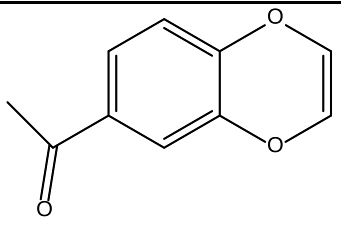 $C_{10}H_8O_3$	SHU-III-42	1000	1000	1000	1000	1000	1000
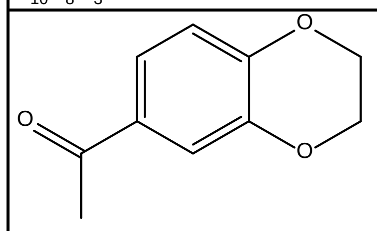 $C_{10}H_{10}O_3$	SHU-III-42A	1000	1000	1000	1000	1000	1000
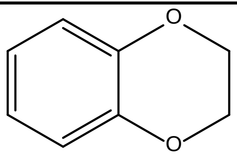 $C_8H_8O_2$	SHU-III-42B	1000	1000	1000	1000	1000	1000
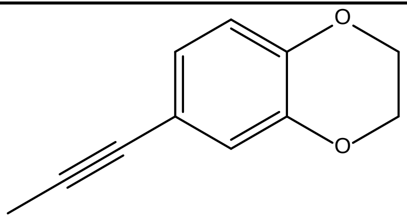 $C_{11}H_{10}O_2$	SHU-III-44	1000	1000	1000	1000	1000	1000
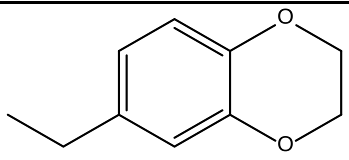 $C_{10}H_{12}O_2$	SHU-III-45	1000	1000	1000	1000	1000	1000

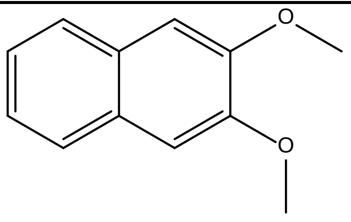 $C_{12}H_{12}O_2$	SHU-III-66	1000	1000	1000	1000	1000	1000
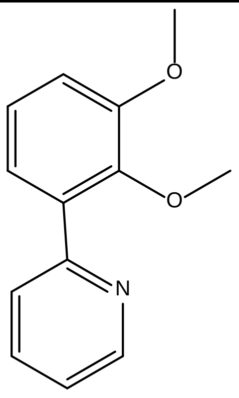 $C_{13}H_{13}NO_2$	SHU-III-87	1000	1000	1000	1000	1000	1000
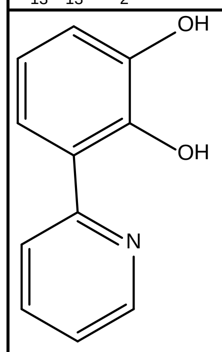 $C_{11}H_9NO_2$	SHU-III-88	1000	1000	1000	1000	1000	1000
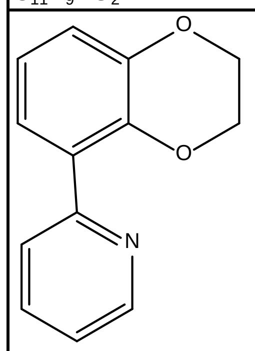 $C_{13}H_{11}NO_2$	SHU-III-90	1000	1000	1000	1000	1000	1000
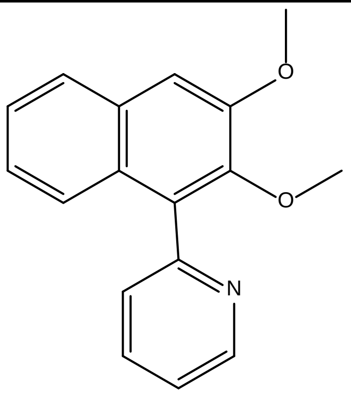 $C_{17}H_{15}NO_2$	SHU-III-93	1000	1000	1000	1000	1000	1000
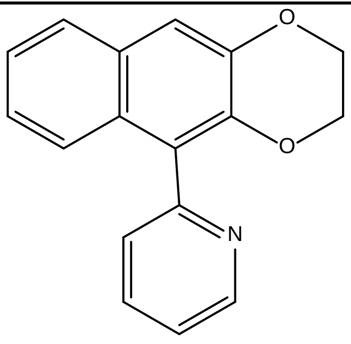 $C_{17}H_{13}NO_2$	SHU-III-94	1000	1000	1000	1000	1000	1000

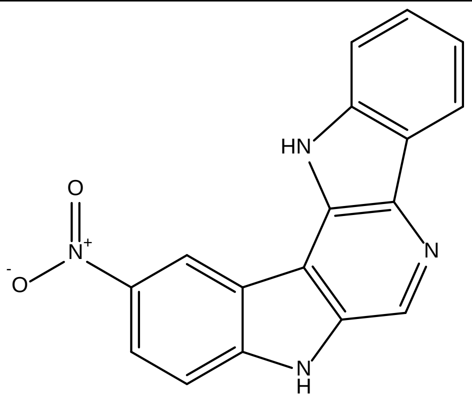 $C_{17}H_{10}N_4O_2$	SLL-II-25	244	500	532		1560	10000
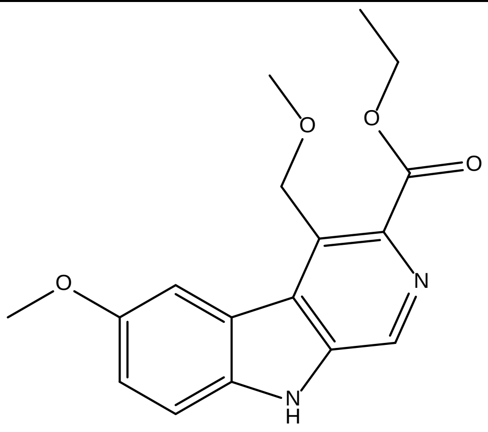 $C_{17}H_{18}N_2O_4$	SPH-121	0.14	1.19	1.72		4	479
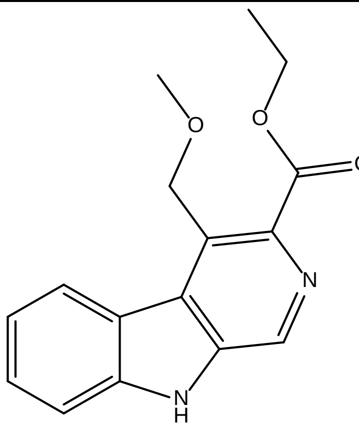 $C_{16}H_{16}N_2O_3$	SPH-165	0.63	2.79	4.85		10.4	1150
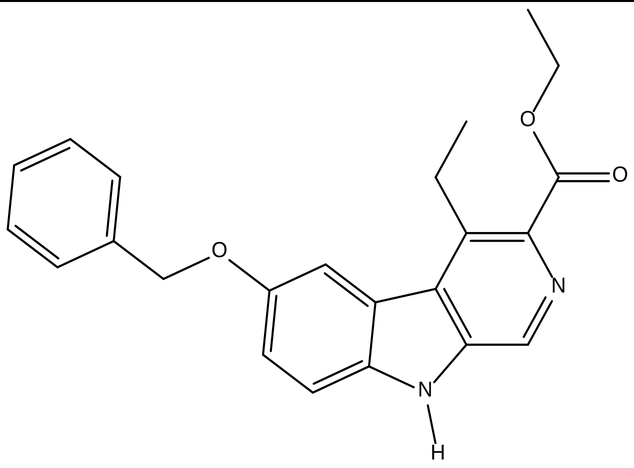 $C_{23}H_{22}N_2O_3$	SPH-166	20.8	78.3	58.7		67.3	10000
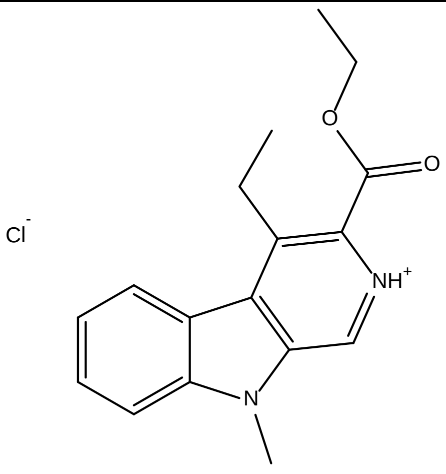 $C_{17}H_{19}ClN_2O_2$	SPH-178	1000	1000	1000		1000	1000
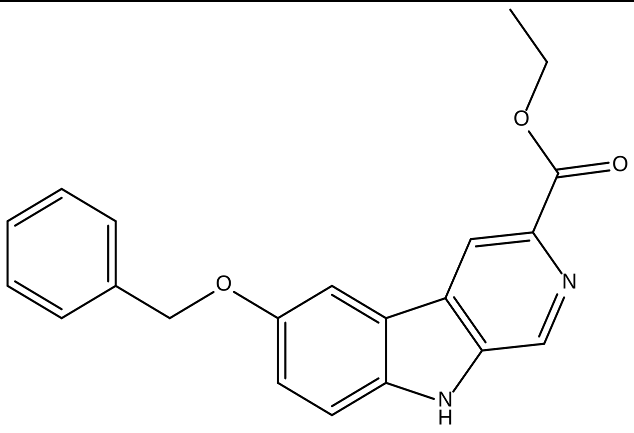 $C_{21}H_{18}N_2O_3$	SPH-195	7.2	168.5	283.5		271	10000

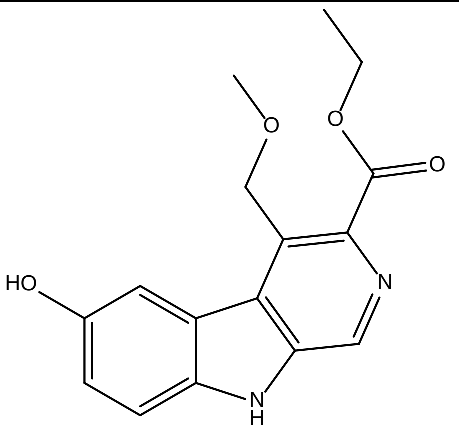 $C_{16}H_{16}N_2O_4$	SPH-38	2	5.4	10.8		18.5	3000
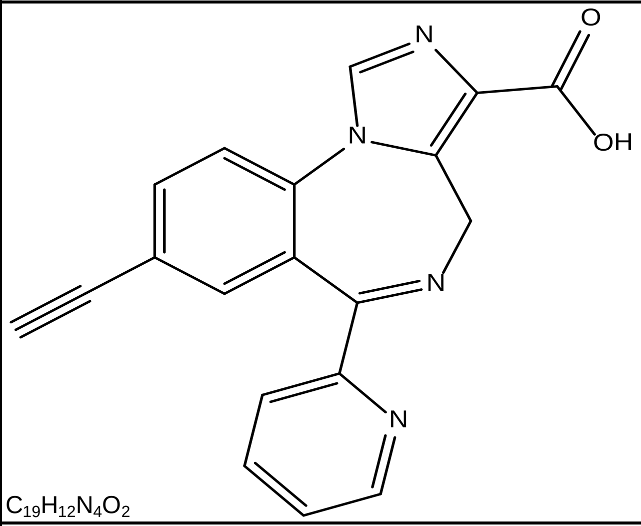 $C_{19}H_{12}N_4O_2$	SR-II-54	174.1	363.2	946.4		966.6	
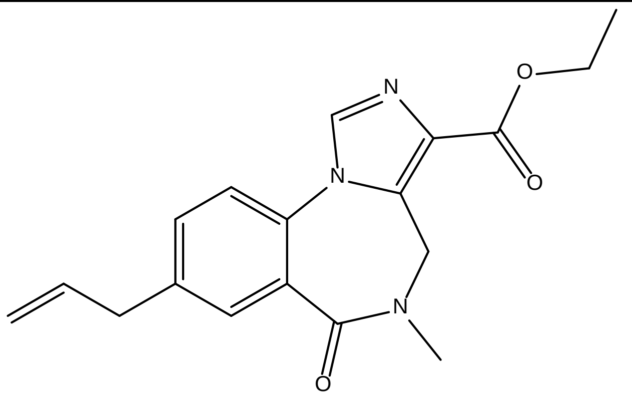 $C_{18}H_{19}N_3O_3$	SVO-8-14	8	25	8	6.9	0.9	14
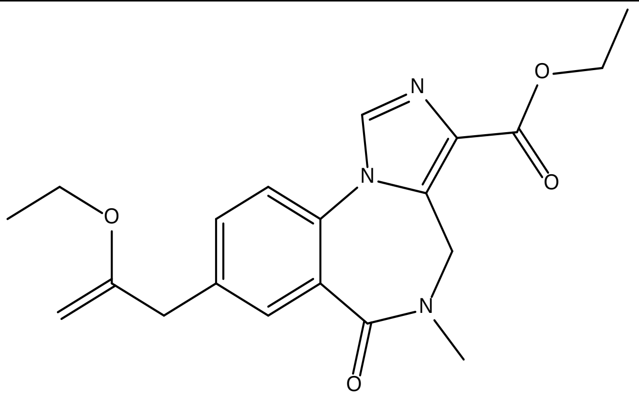 $C_{20}H_{23}N_3O_4$	SVO-8-20	11	40	28	19	8.6	138
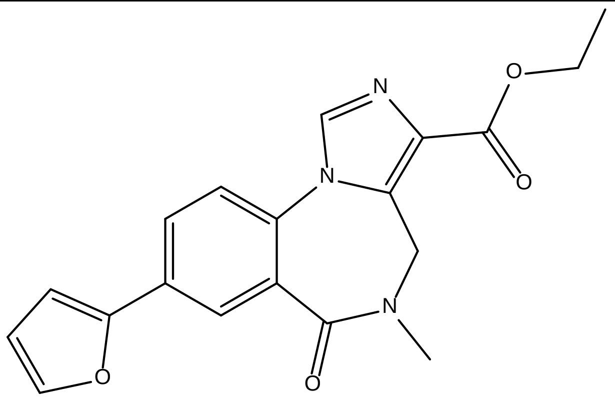 $C_{19}H_{17}N_3O_4$	SVO-8-30	1.1	5.3	5.3	2.8	0.6	15
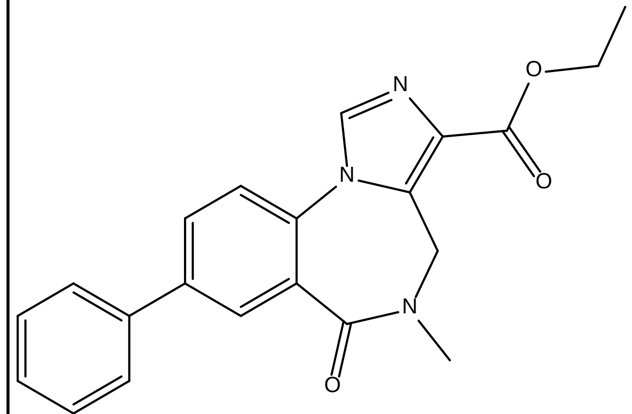 $C_{21}H_{19}N_3O_3$	SVO-8-67	7	41	26	15	2.3	191

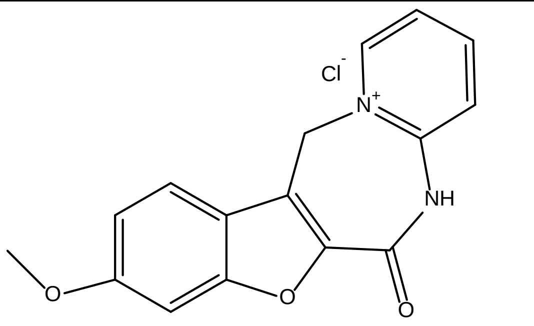 $C_{16}H_{13}ClN_2O_3$	TC-T-JG-24	746.5	8081	1543		2364	
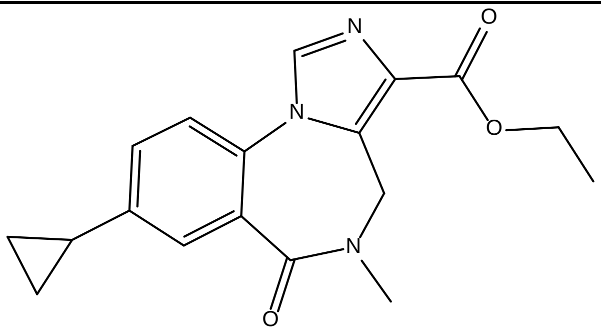 $C_{18}H_{19}N_3O_3$	TC-YT-II-76	101.1	1.897	5.816		11.99	
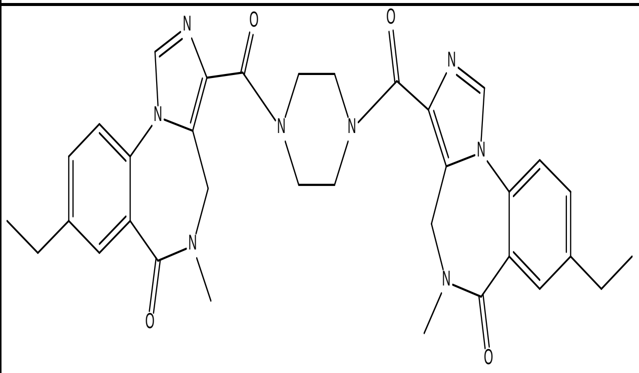 $C_{34}H_{36}N_8O_4$	TC-YT-III-19						
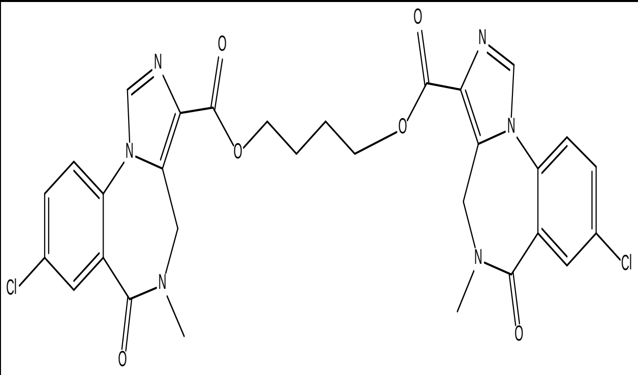 $C_{30}H_{26}Cl_2N_6O_6$	TC-YT-TC-2	2657	737.8	643		1973	
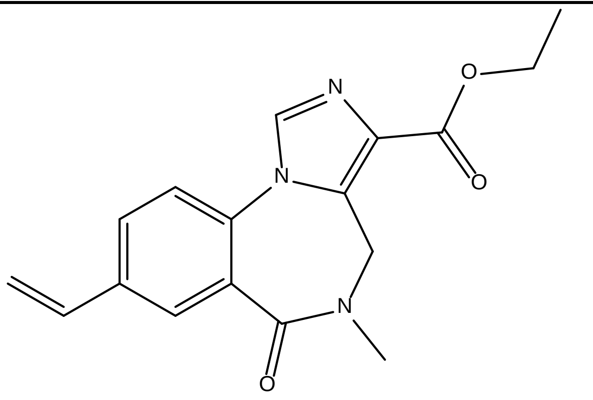 $C_{17}H_{17}N_3O_3$	TG-4-29	8.3	10.2	6.9		0.4	7.61
	TG-4-29	2.8	3.9	2.7	2.1	0.18	3.9

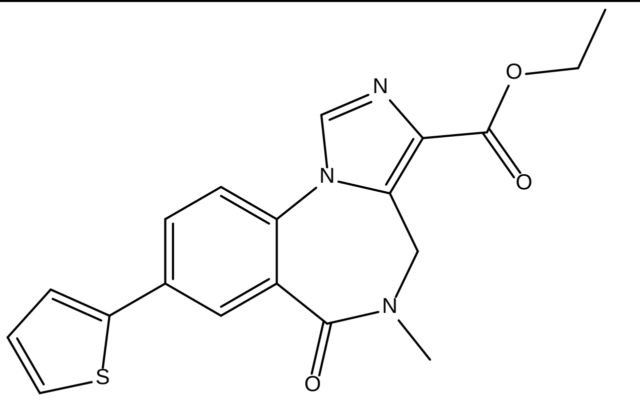 $C_{19}H_{17}N_3O_3S$	TG-4-39	1.6	34	24	5.6	1.4	23
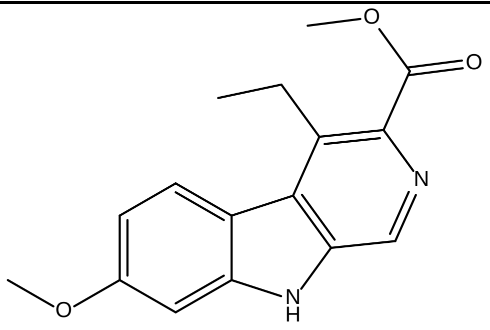 $C_{16}H_{16}N_2O_3$	TG-I-97						
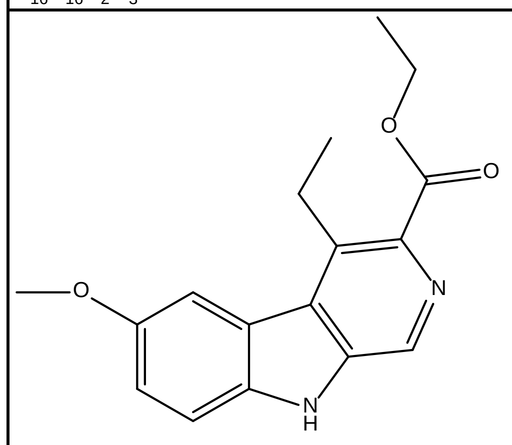 $C_{17}H_{18}N_2O_3$	TG-II-82	1.6	2.9	2.8		1	1000
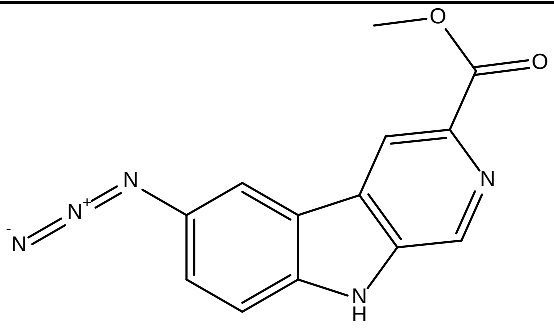 $C_{13}H_9N_5O_2$	TJH-6AZ						
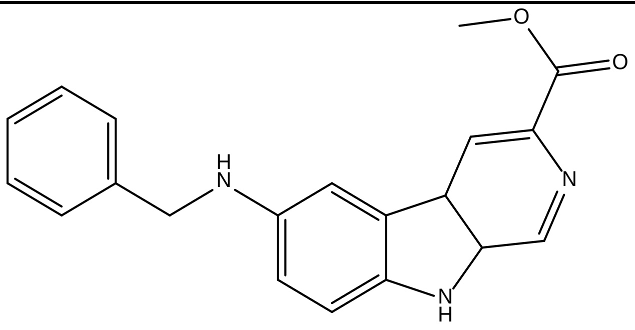 $C_{20}H_{19}N_3O_2$	TJH-IV-43	5.42	30.19	48.9		475	10000

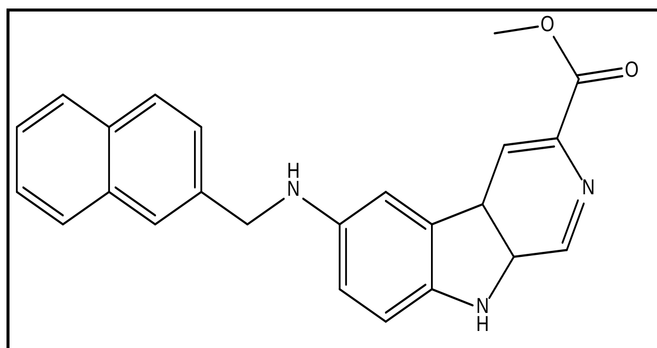 $C_{24}H_{21}N_3O_2$	TJH-IV-50	30.7	205	271		814	10000
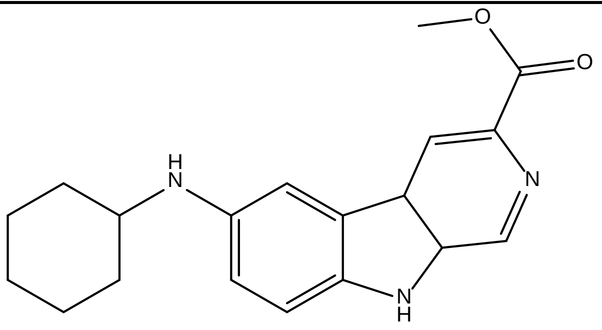 $C_{19}H_{23}N_3O_2$	TJH-IV-51	2.39	17.4	14.5		316	10000
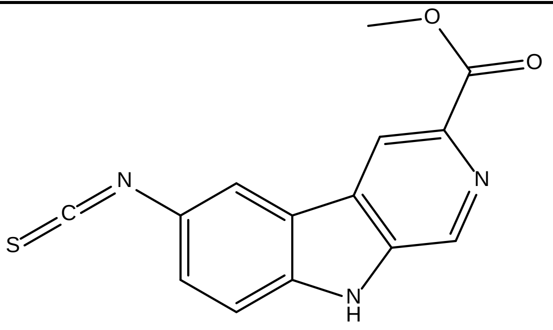 $C_{14}H_9N_3O_2S$	TJH-V-88	3.41		30		140.9	10000
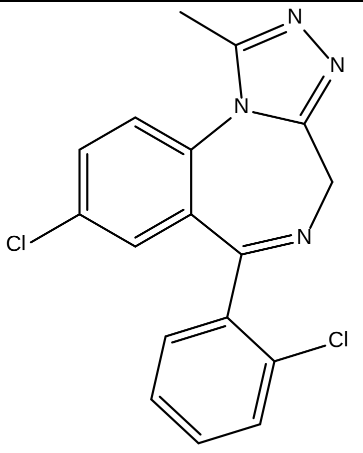 $C_{17}H_{12}Cl_2N_4$	triazolam						
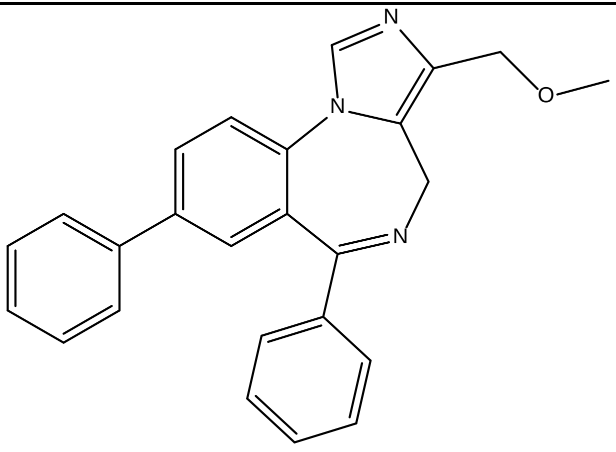 $C_{25}H_{21}N_3O$	TSC-I-93	5000	5000	5000		304	5000
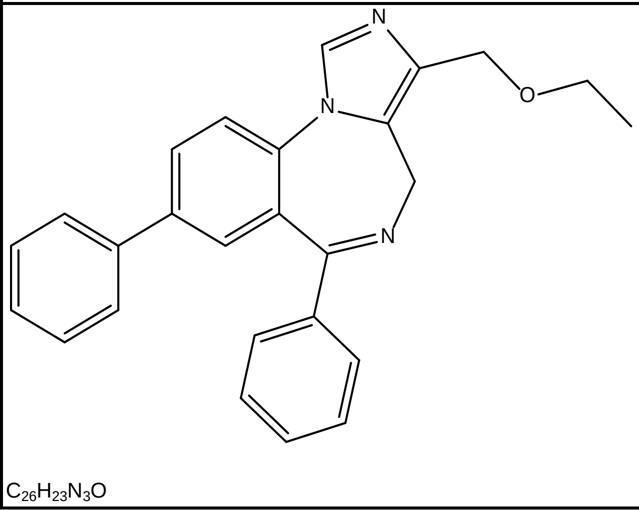 $C_{26}H_{23}N_3O$	TSC-I-94						

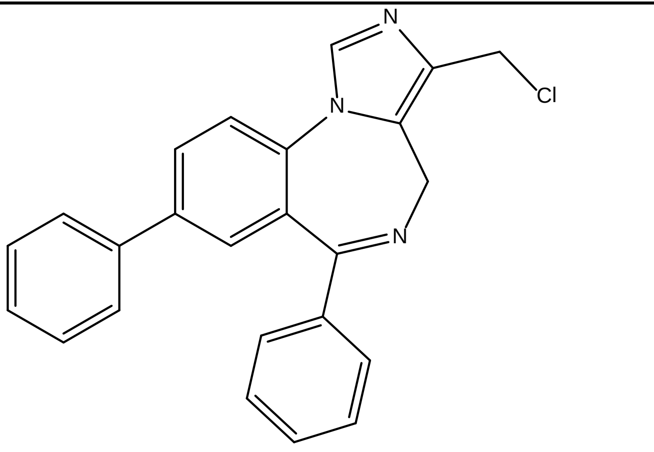 $C_{24}H_{18}ClN_3$	TSC-I-95						
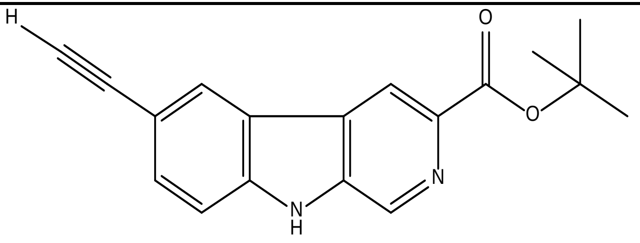 $C_{18}H_{16}N_2O_2$	WY-A-99-2(WYS8)	0.972	111	102	2000	208	1980
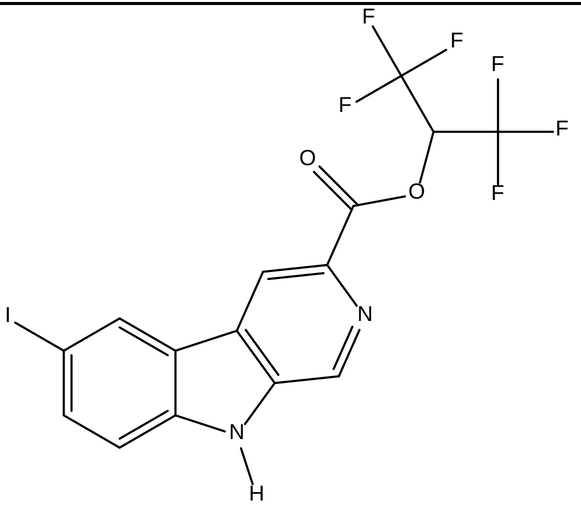 $C_{15}H_7F_6IN_2O_2$	WY-B-08	78	301	131	3000	681	3000
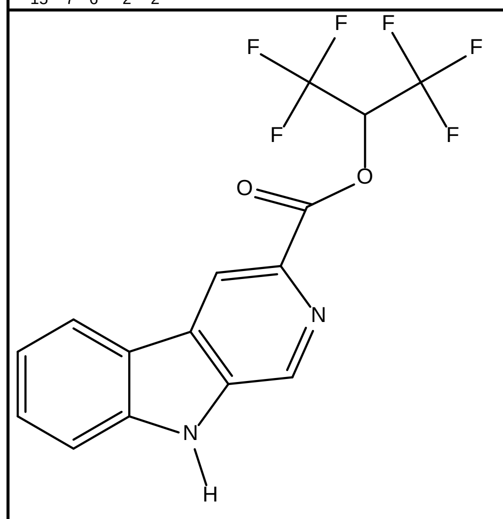 $C_{15}H_8F_6N_2O_2$	WY-B-09-1	3.99	8	32	1000	461	2000
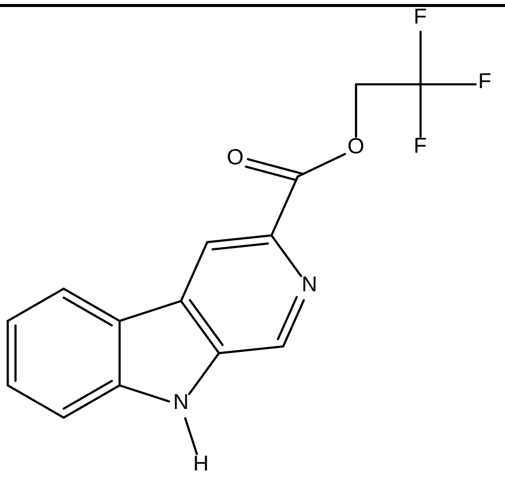 $C_{14}H_9F_3N_2O_2$	WY-B-09-2	1000	2000	2000	3000	1000	3000
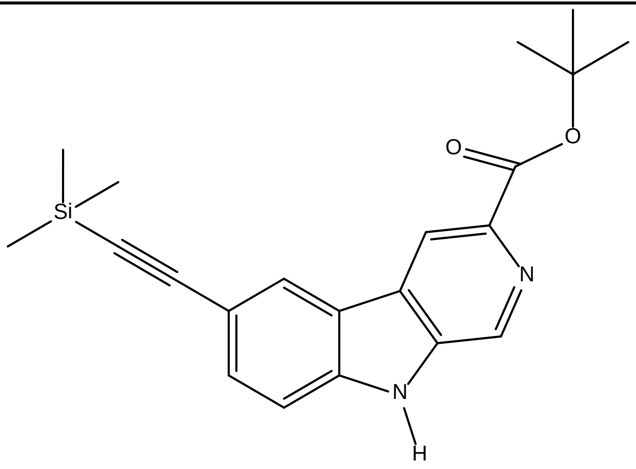 $C_{21}H_{24}N_2O_2Si$	WY-B-14(WYS7)	6.84	30	36	2000	108	1000

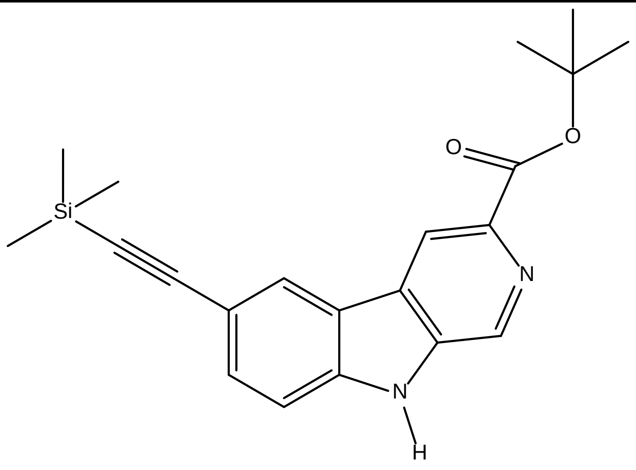 $C_{21}H_{24}N_2O_2Si$	WY-B-14(WYS7)	8.5	165	245		1786	5000
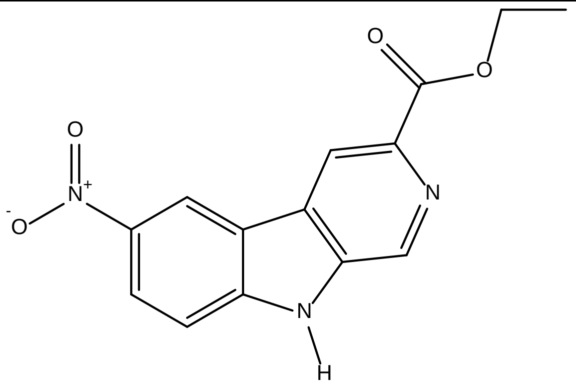 $C_{14}H_{11}N_3O_4$	WY-B-15	0.92	0.83	0.58	2080	4.42	646
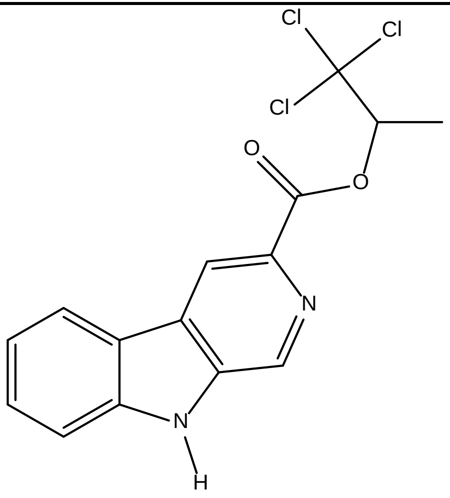 $C_{15}H_{11}Cl_3N_2O_2$	WY-B-17	2000	2000	2000	3000	2000	5000
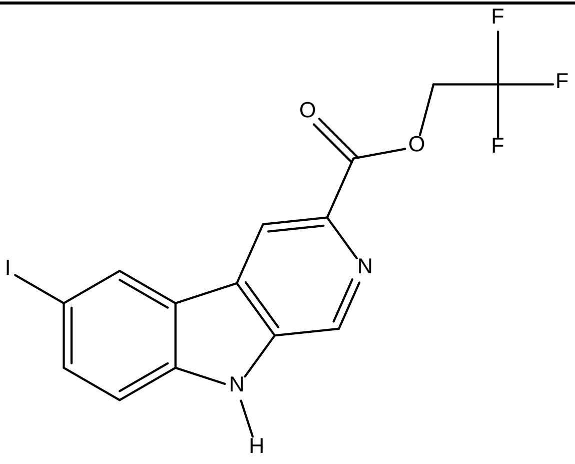 $C_{14}H_8F_3IN_2O_2$	WY-B-20	12	39	47	2000	122	3000
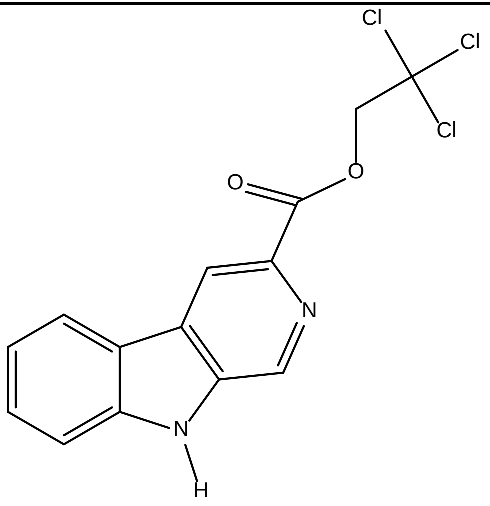 $C_{14}H_9Cl_3N_2O_2$	WY-B-23-1	10	33	43	1000	189	2000
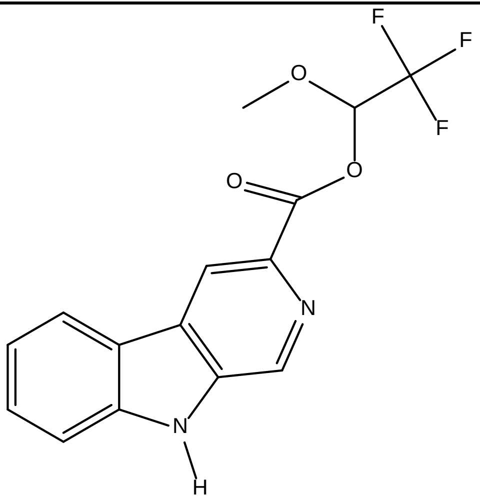 $C_{15}H_{11}F_3N_2O_3$	WY-B-23-2(WYS11)	4.2	37.7	39	2000	176	69.4

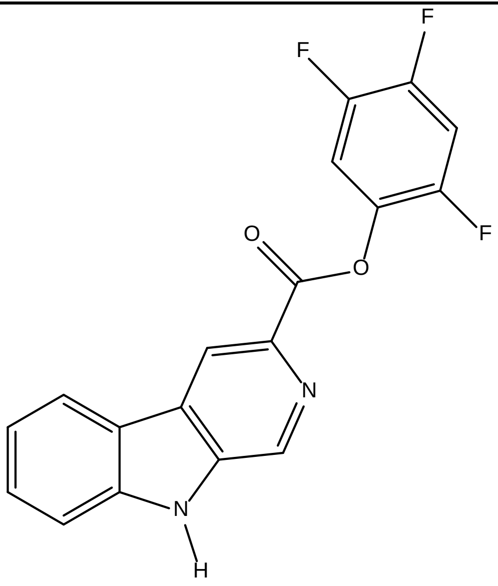 $C_{18}H_{9}F_{3}N_{2}O_{2}$	WY-B-24	22.02	177	44.78	3000	422	3000
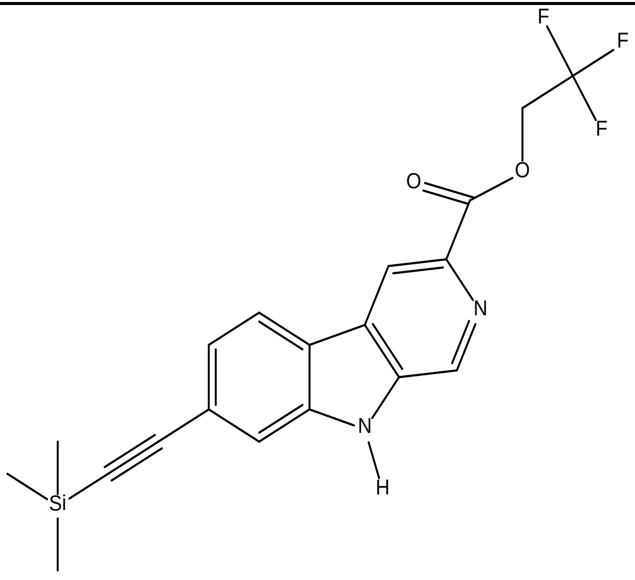 $C_{19}H_{17}F_{3}N_{2}O_{2}Si$	WY-B-25	7.6	40	66	2000	263	2000
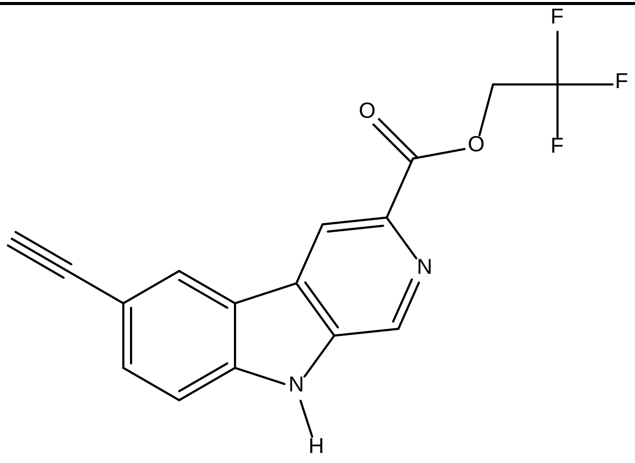 $C_{16}H_{9}F_{3}N_{2}O_{2}$	WY-B-26-2	4.45	44.57	42.66	2000	124	2000
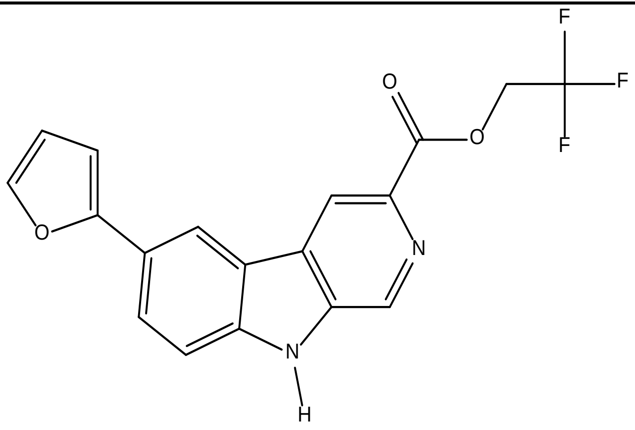 $C_{18}H_{11}F_{3}N_{2}O_{3}$	WY-B-27-1	26	143	117	3000	127	2000
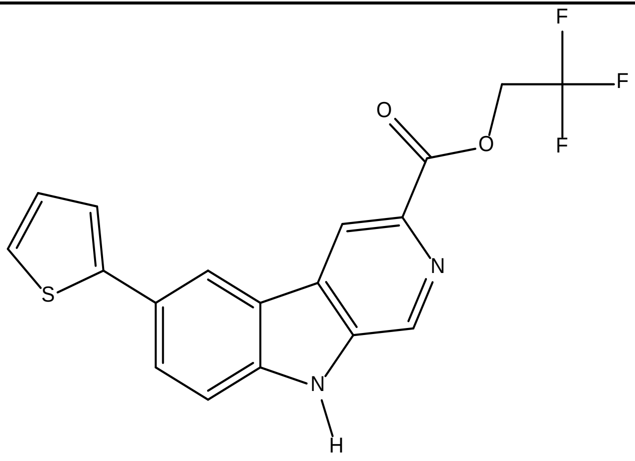 $C_{18}H_{11}F_{3}N_{2}O_{2}S$	WY-B-27-2	9.19	111	72	2000	449	2000
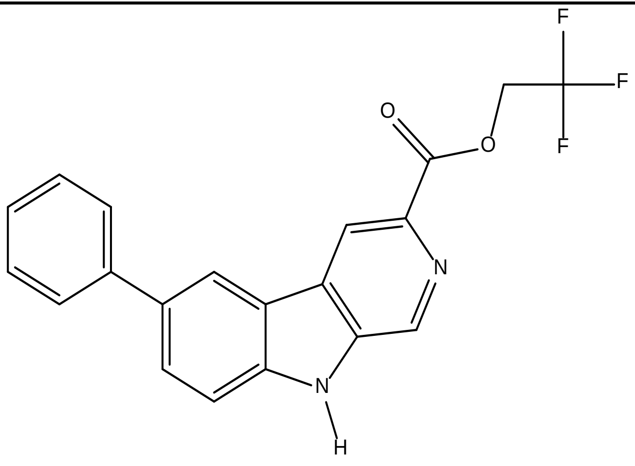 $C_{20}H_{13}F_{3}N_{2}O_{2}$	WY-B-29-2	25	137	125	2000	299	2000

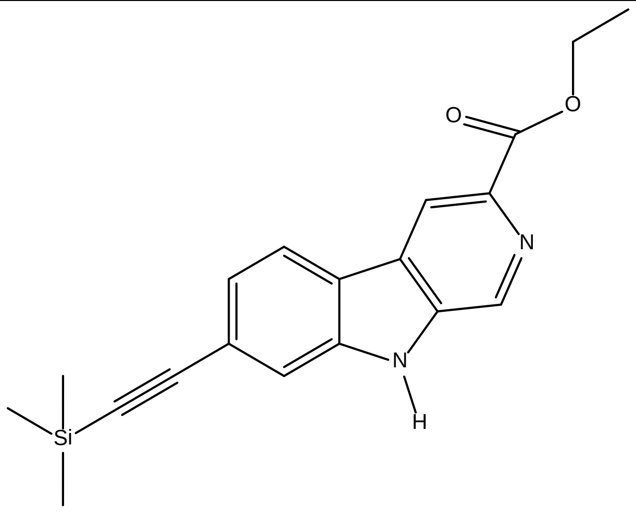 $C_{19}H_{20}N_2O_2Si$	WY-B-99-1	4.4	4.5	5.58	2000	47	2000
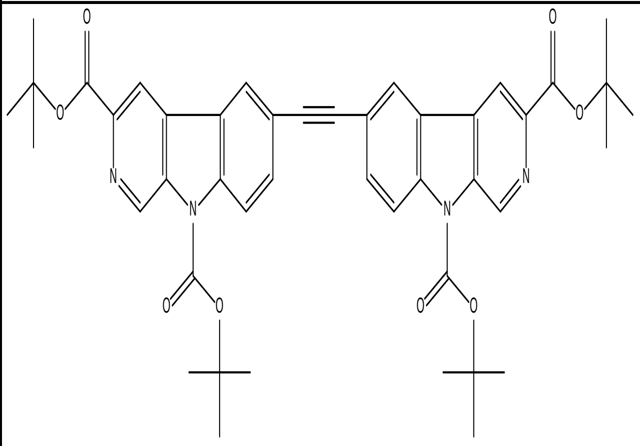 $C_{44}H_{46}N_4O_8$	WYS1 C45H50N4O6	missing					
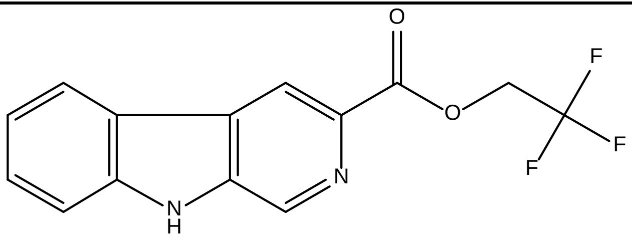 $C_{14}H_9F_3N_2O_2$	WYS10 C14H9F3N2O2	0.88	36	25.6		548.7	15.3
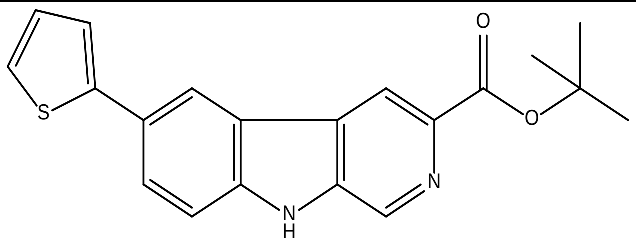 $C_{20}H_{18}N_2O_2S$	WYS12 C20H18N2O2S	37	166	314		2861	5000
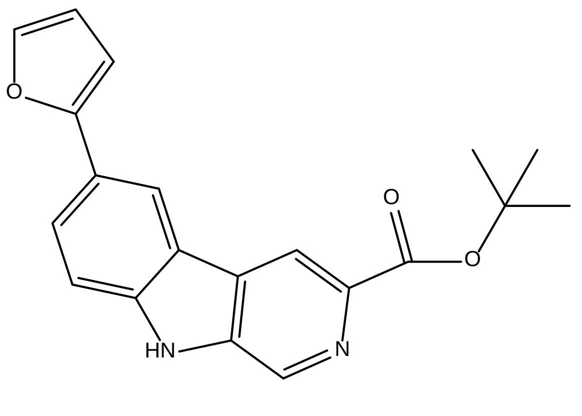 $C_{20}H_{18}N_2O_3$	WYS13 C20H18N2O3	2.442	13	27.5		163	5000
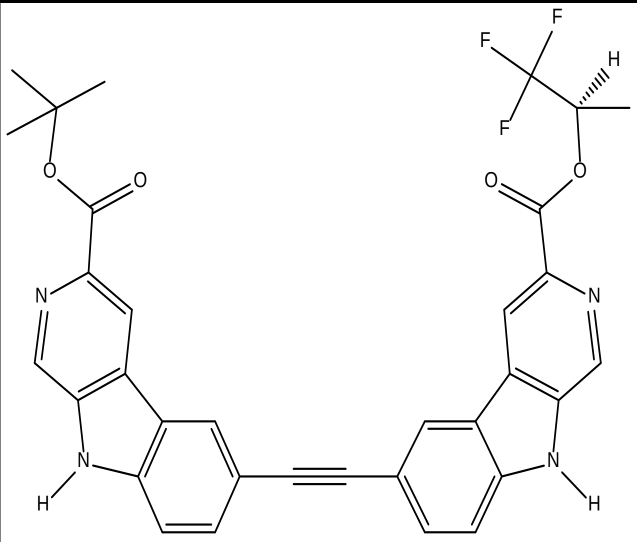 $C_{33}H_{25}F_3N_4O_4$	WYS14 C34H29F3N4O4	798	5000			5000	5000

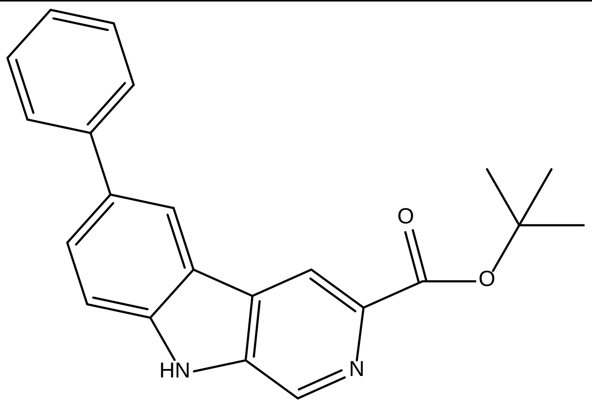 $C_{22}H_{20}N_2O_2$	WYS15 C22H20N2O2	3.63	2.02	44.3		76.5	5000
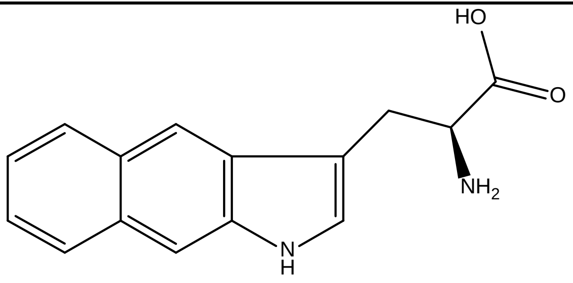 $C_{15}H_{14}N_2O_2$	WYS16 C18H22N2O2	5000	5000			5000	5000
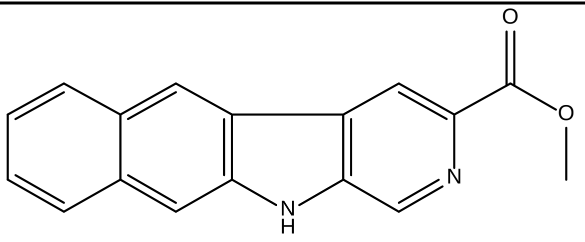 $C_{17}H_{12}N_2O_2$	WYS17 C17H18N2O2	missing					
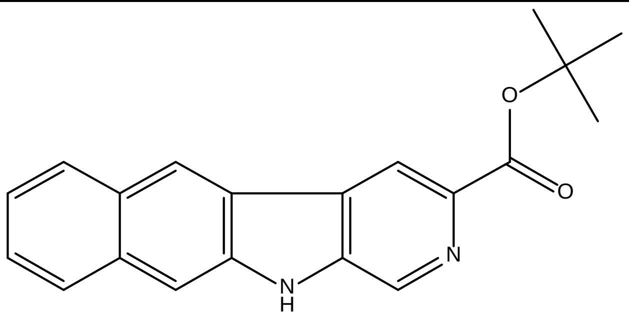 $C_{20}H_{18}N_2O_2$	WYS18 C21H22N2O2	missing					
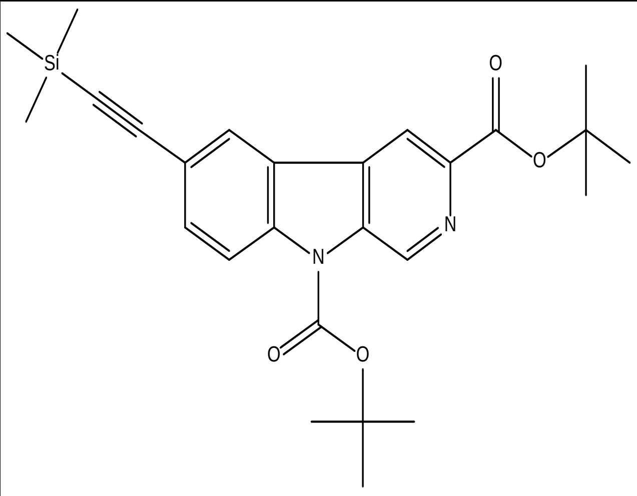 $C_{26}H_{32}N_2O_4Si$	WYS19 C26H32N2O4Si						
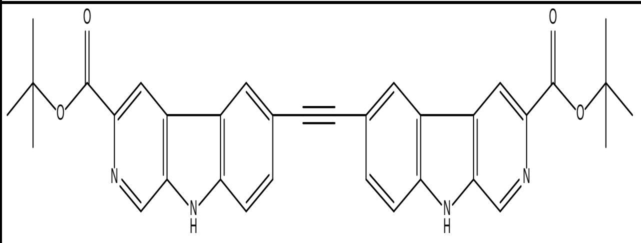 $C_{34}H_{30}N_4O_4$	WYS2 C35H34N4O4						
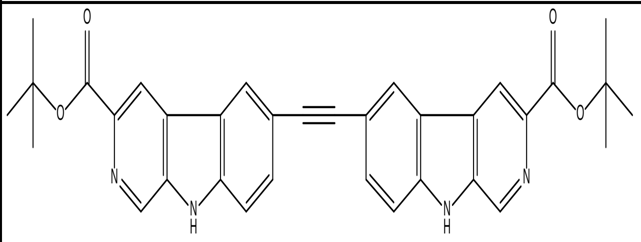 $C_{34}H_{30}N_4O_4$	WYS2 C35H34N4O4	30	124	100	300	300	4000

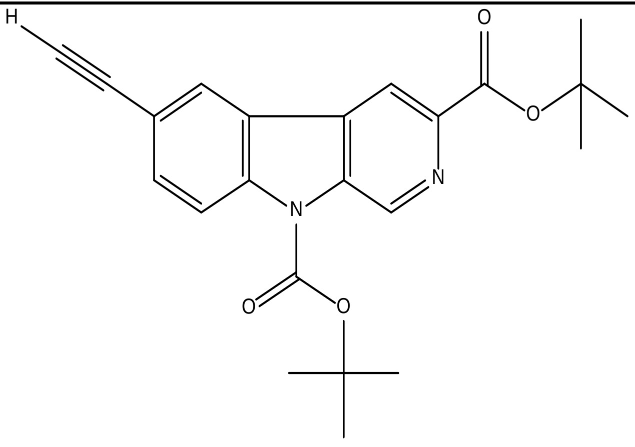 $C_{23}H_{24}N_2O_4$	WYS20 C23H24N2O4	450	5000			5000	5000
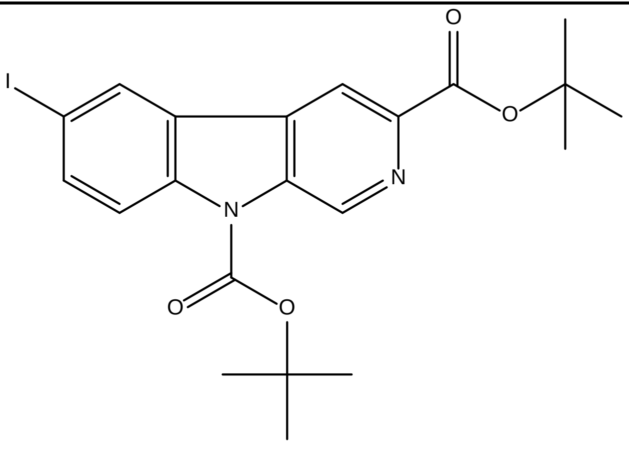 $C_{21}H_{23}IN_2O_4$	WYS21 C21H23IN2O4	missing					
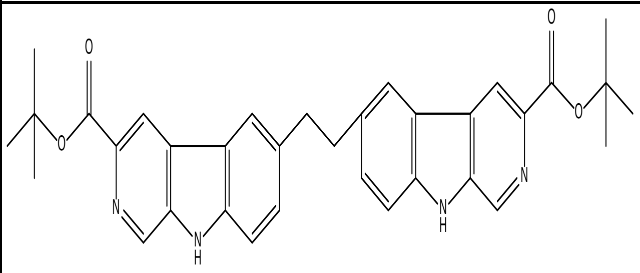 $C_{34}H_{34}N_4O_4$	WYS3 C35H38N4O4	241	5000	1841		5000	5000
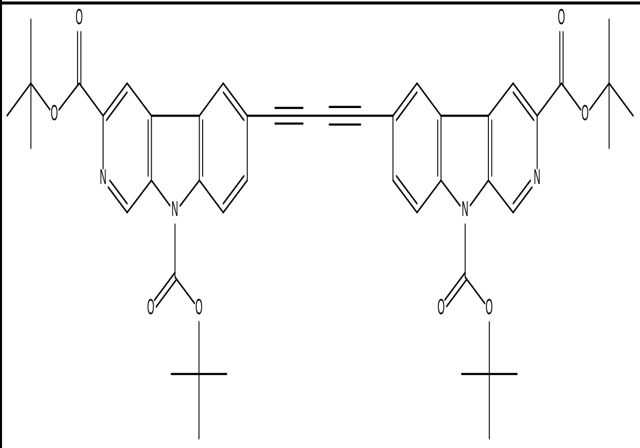 $C_{46}H_{46}N_4O_8$	WYS4 C48H56N4O8	missing					
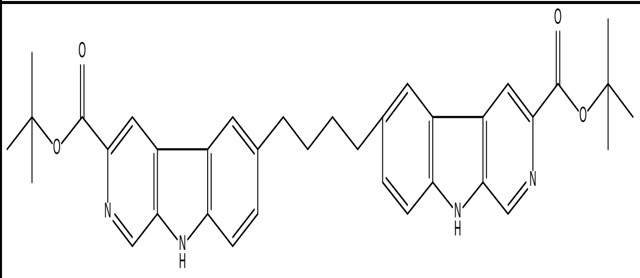 $C_{37}H_{42}N_4O_4$	WYS5 C37H42N4O4	5000	5000			5000	5000
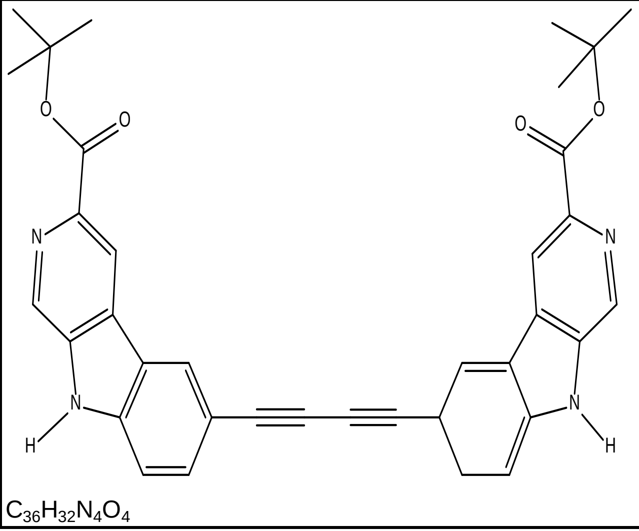 $C_{36}H_{32}N_4O_4$	WYS6 C37H35N4O4	120	1059	3942		5000	5000

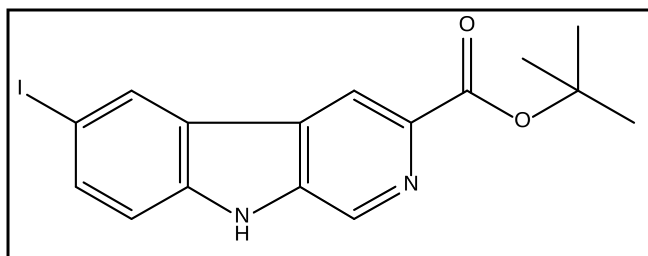 $C_{16}H_{15}IN_2O_2$	WYS9 C16H15IN2O2	2.72	22.2	23.1		562	122
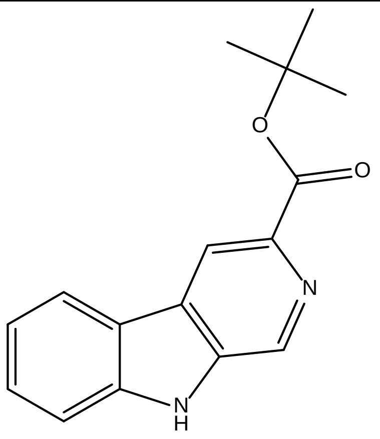 $C_{16}H_{16}N_2O_2$	WYSC1 (BCCt)	1.094	5.44	12.3		69.8	21.2
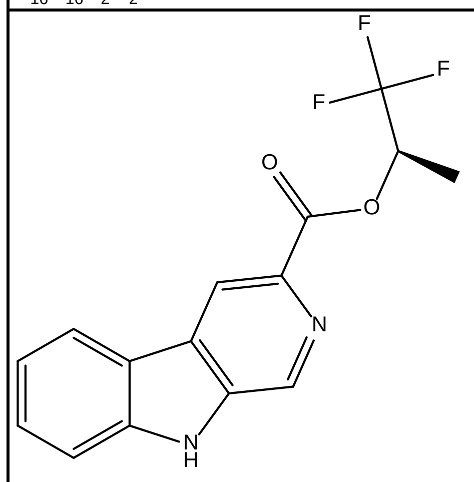 $C_{15}H_{11}F_3N_2O_2$	WYSC2 C15H11F3N2O2	14.14	113	170		518	61.2
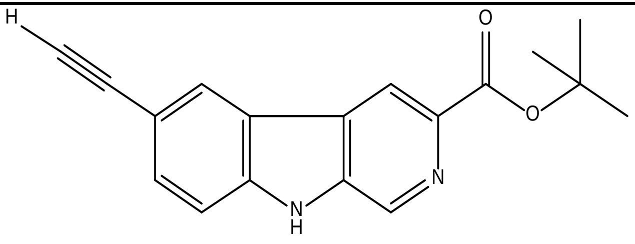 $C_{18}H_{16}N_2O_2$	WY-TSC-4(WYS8)	109.431	2374.5	1656.4		2663.5	
	WY-TSC-4(WYS8)	0.007	0.99	1.63		51.04	
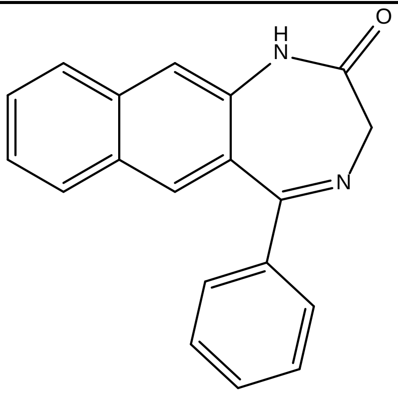 $C_{19}H_{14}N_2O$	WZ-030	279	210	200		45.15	1000

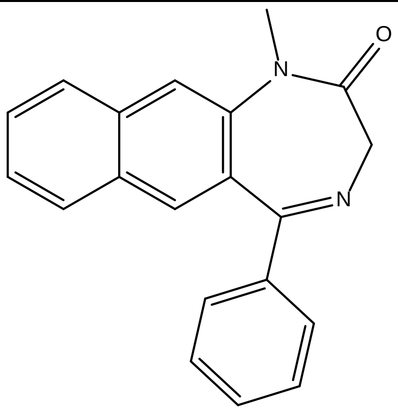 $C_{20}H_{16}N_2O$	WZ-032	965	345	590		150	3000
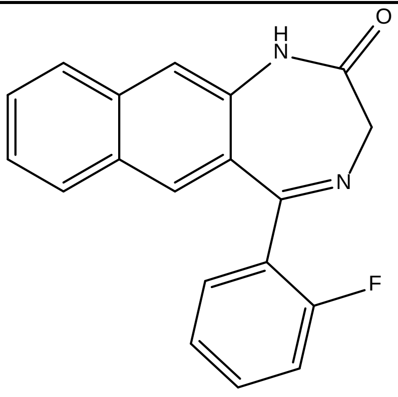 $C_{19}H_{13}FN_2O$	WZ-069	40	30.5	38.5		12.6	1000
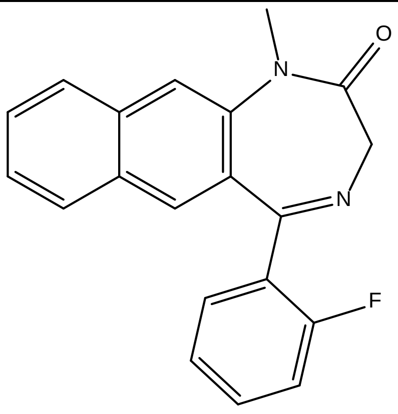 $C_{20}H_{15}FN_2O$	WZ-070	72.7	30.7	53.2		18.6	300
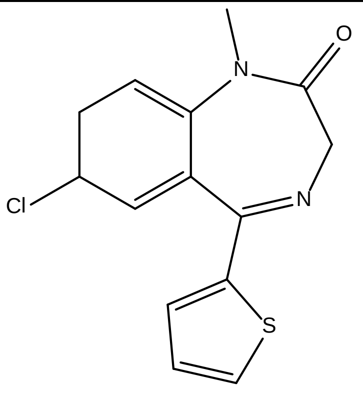 $C_{14}H_{13}ClN_2OS$	WZ-113	19.2	13.2	13.4		11.5	300
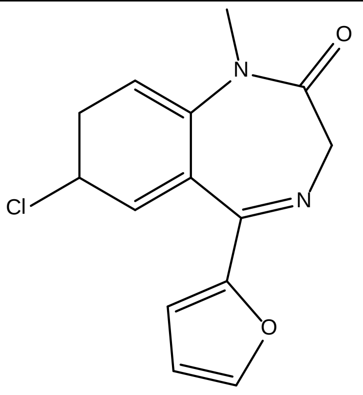 $C_{14}H_{13}ClN_2O_2$	WZ-148	300	300	300		300	300
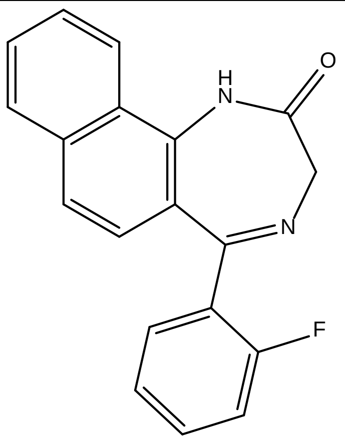 $C_{19}H_{13}FN_2O$	WZ-153	199	151.3	191.6		73.5	1000
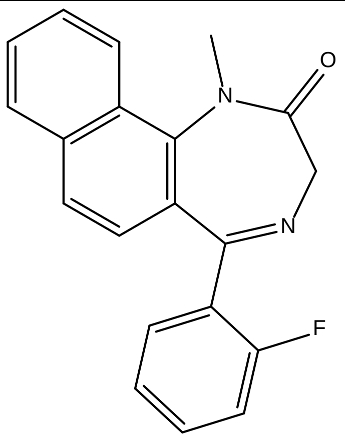 $C_{20}H_{15}FN_2O$	WZ-154	>300	>300	>300		>300	>300

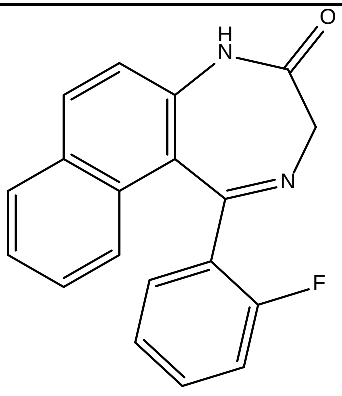 $C_{19}H_{13}FN_2O$	WZ-175	1000	1000	1000		1000	1000
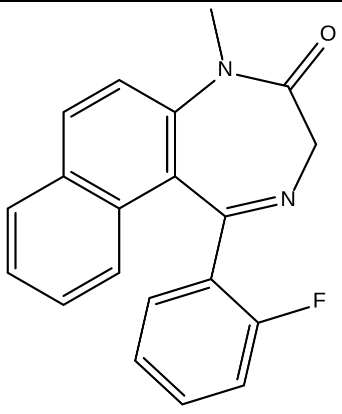 $C_{20}H_{15}FN_2O$	WZ-176	>300	>300	>300		>300	>300
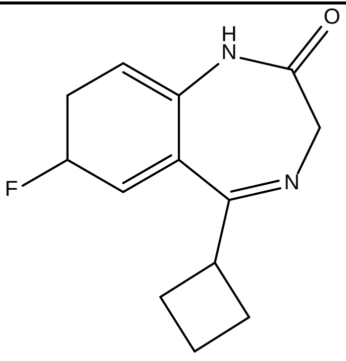 $C_{13}H_{15}FN_2O$	WZ-185	>300	>300	>300		>300	>300
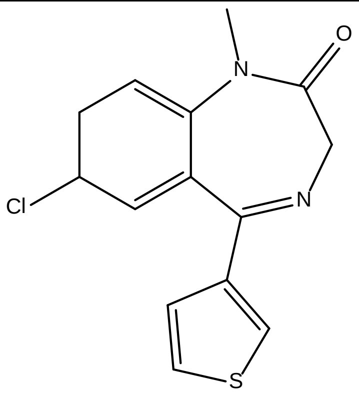 $C_{14}H_{13}ClN_2OS$	WZ-198	>300	>300	>300		>300	>300
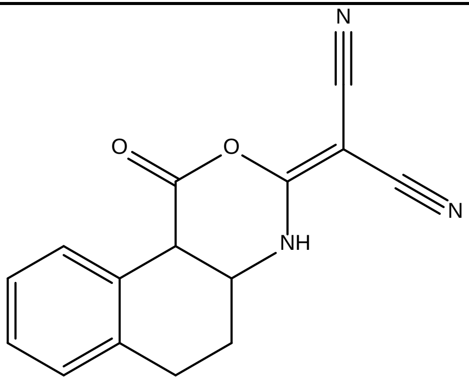 $C_{15}H_{11}N_3O_2$	WZ-201						
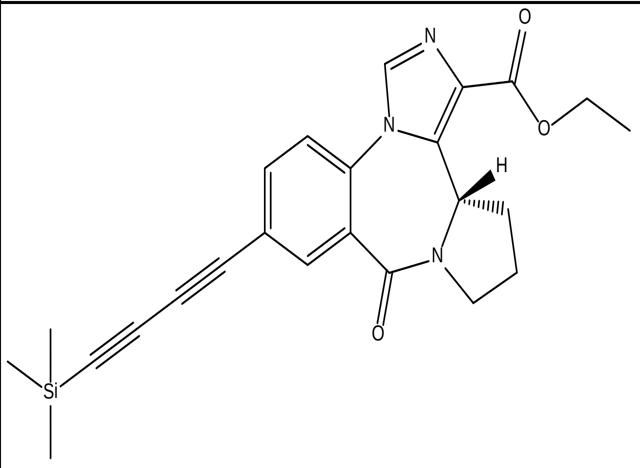 $C_{24}H_{25}N_3O_3Si$	XHE-I-027	1000	1000	1000		180	

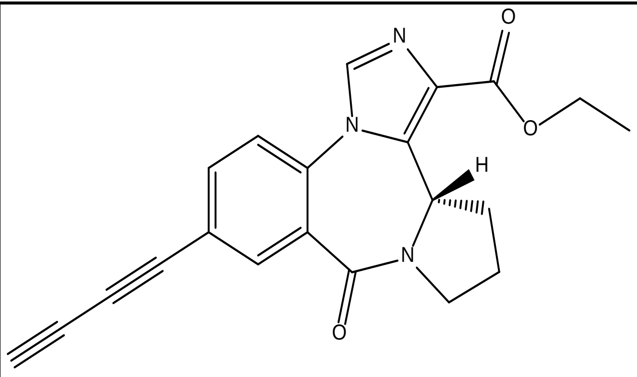 $C_{21}H_{17}N_3O_3$	XHE-I-030	1000	1000	1000	1000	53	1000
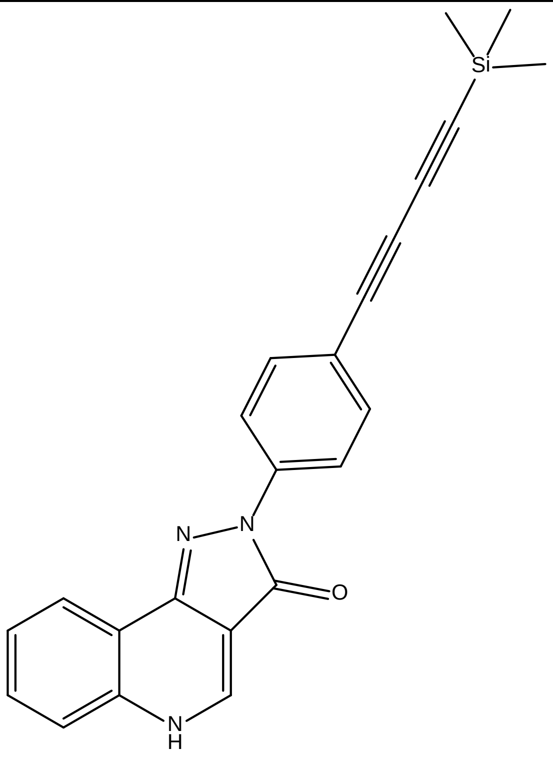 $C_{23}H_{19}N_3OSi$	XHE-I-038	7.3	5	34		132	1000
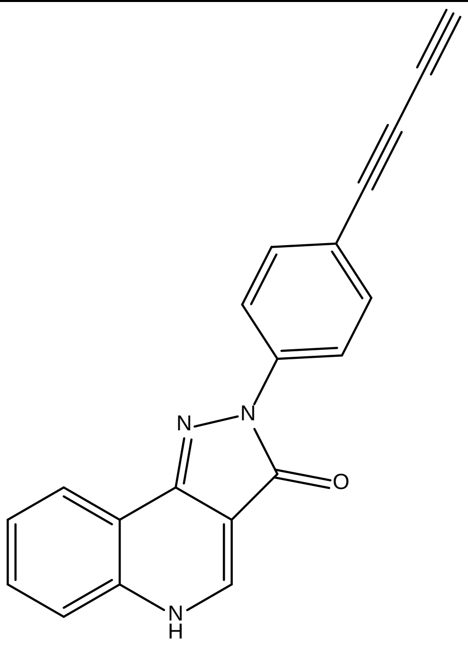 $C_{20}H_{11}N_3O$	XHE-I-043	31	95	272		81	1000
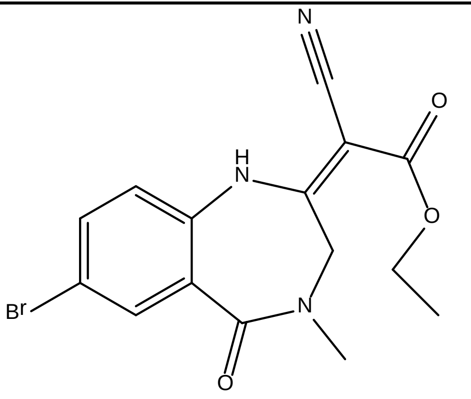 $C_{15}H_{14}BrN_3O_3$	XHE-I-046	1000	1000	1000		1000	1000
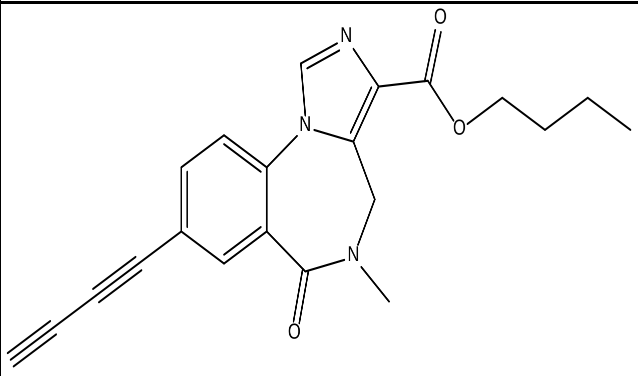 $C_{21}H_{19}N_3O_3$	XHE-I-048	220	440	481		50	160
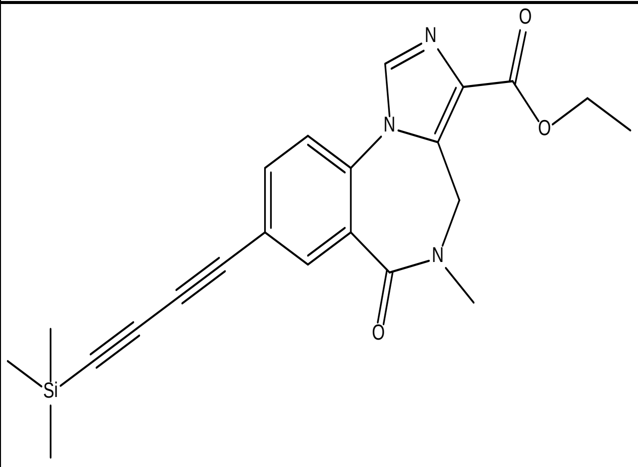 $C_{22}H_{23}N_3O_3Si$	XHE-I-050	254	300	300		195	3000

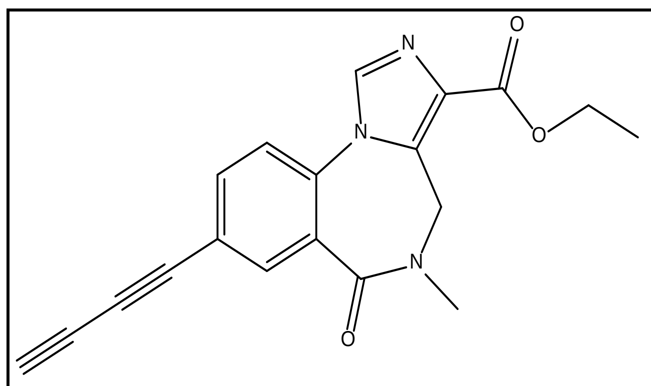 $C_{19}H_{15}N_3O_3$	XHE-I-051	35	39	42		5.3	979
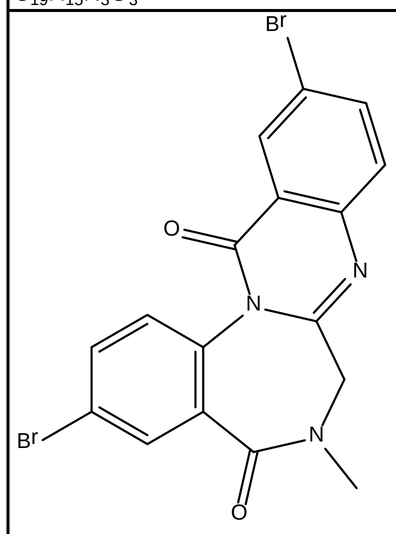 $C_{17}H_{11}Br_2N_3O_2$	XHE-I-055b	615	1000	1000	2213	268	1524
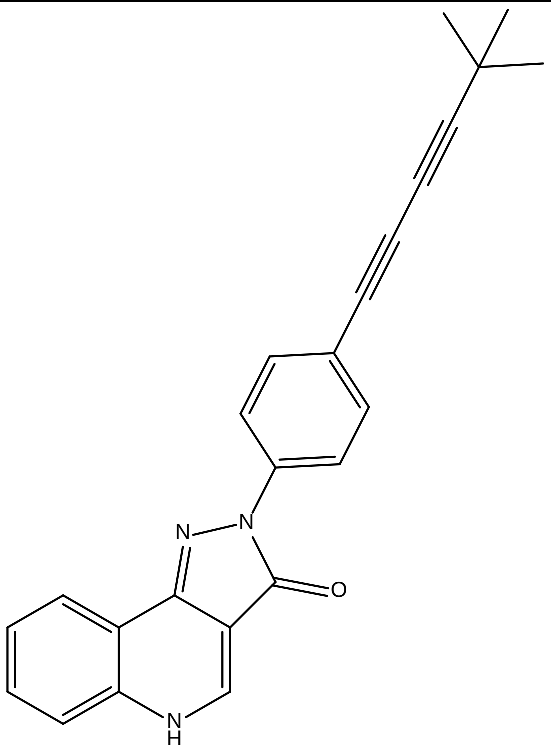 $C_{24}H_{19}N_3O$	XHE-I-065	7.2	17	18	500	57	500
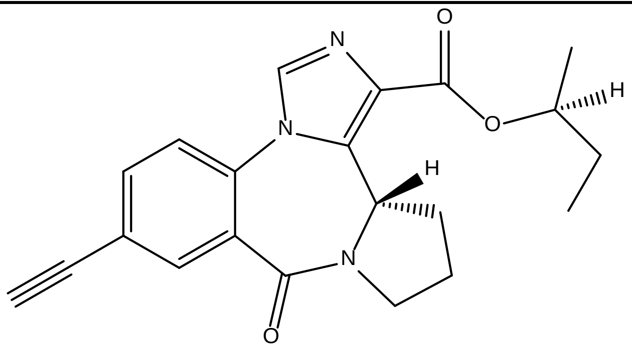 $C_{21}H_{21}N_3O_3$	XHE-I-066A(SS)	196.66	176.14	95.4	74.26	125.06	186.25
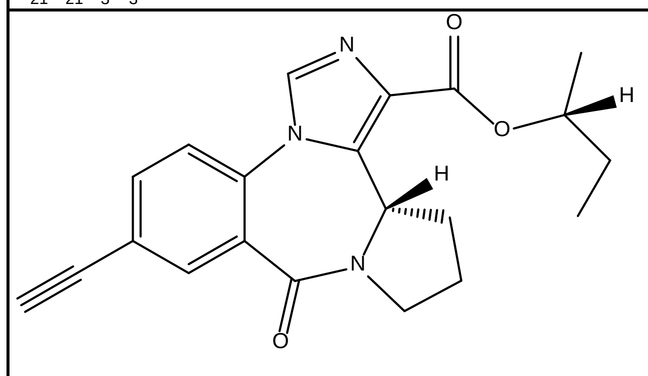 $C_{21}H_{21}N_3O_3$	XHE-I-066B(SR)	76.04	73.56	45.05	22.72	53.42	51.32

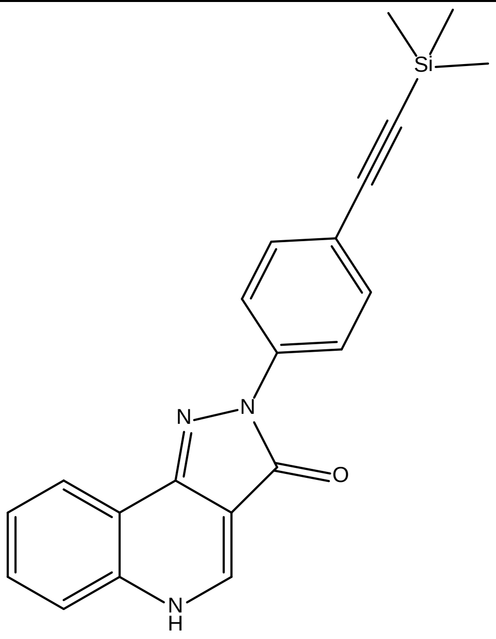 $C_{21}H_{19}N_3OSi$	XHE-I-073	62	155	217	1000	393	1000
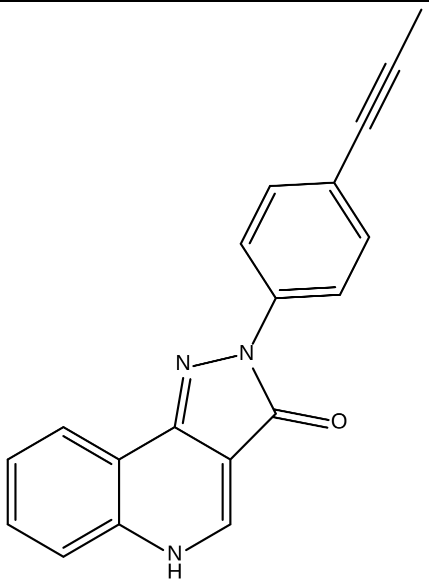 $C_{19}H_{13}N_3O$	XHE-I-093	2	7.1	8.9	1107	20	1162
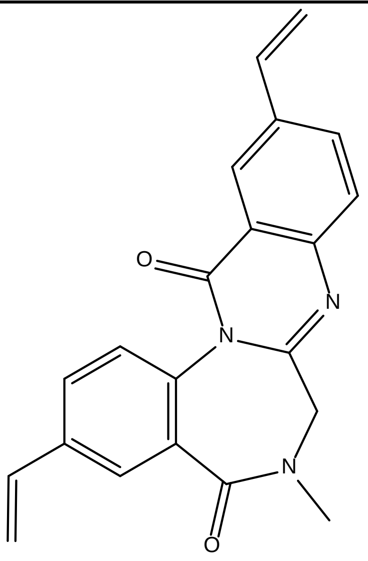 $C_{21}H_{17}N_3O_2$	XHE-I-096	3079	1000	4636	1601	567	2712
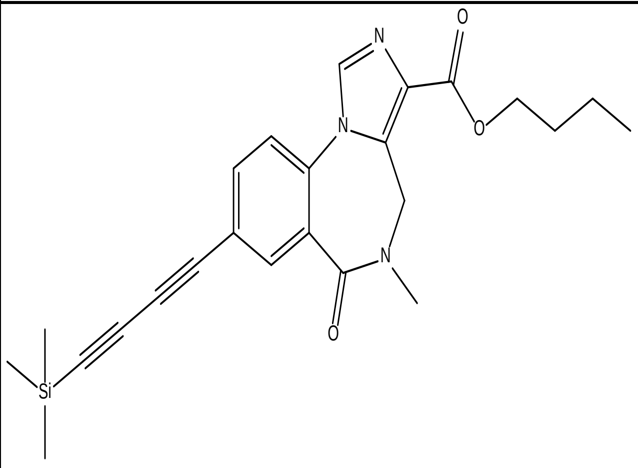 $C_{24}H_{27}N_3O_3Si$	XHE-I-47	705	2502	2460		480	10000
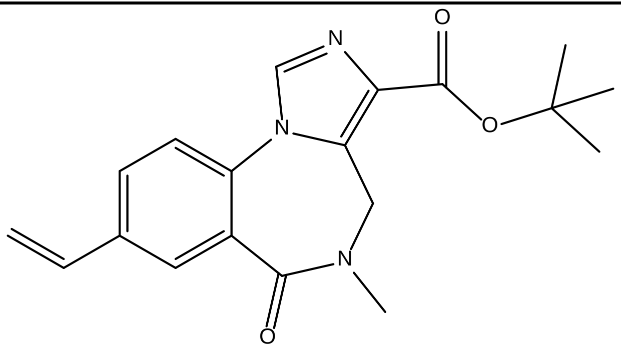 $C_{19}H_{21}N_3O_3$	XHE-II-002	8.3	18	13	3.9	1.5	11

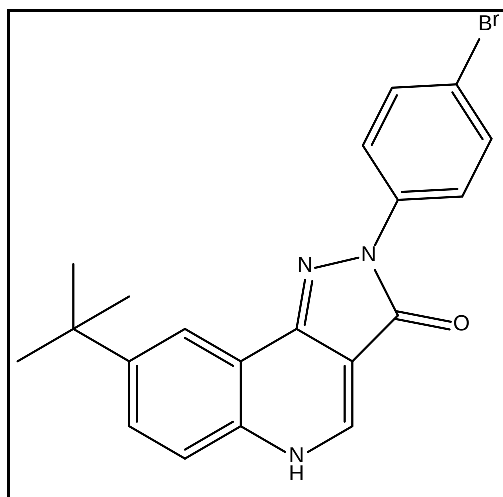 $C_{20}H_{18}BrN_3O$	XHE-II-006a	4.7	4.4	20	1876	89	3531
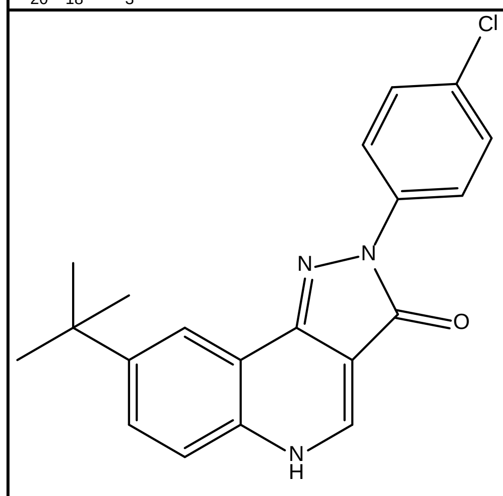 $C_{20}H_{18}ClN_3O$	XHE-II-006b	3.7	15	12	1897	144	1000
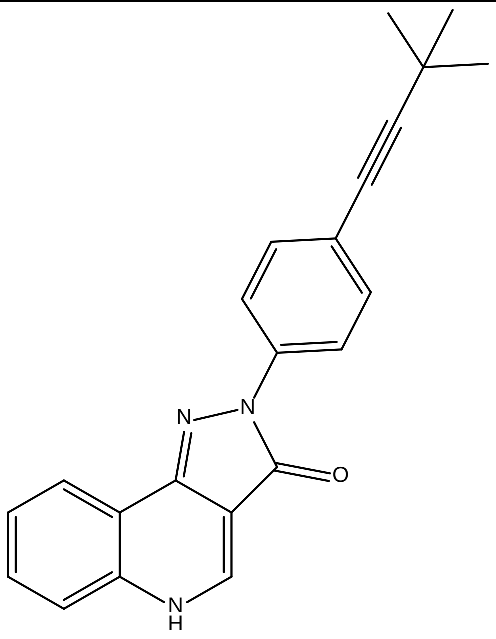 $C_{22}H_{19}N_3O$	XHE-II-011	9	60	39	3233	90	1000
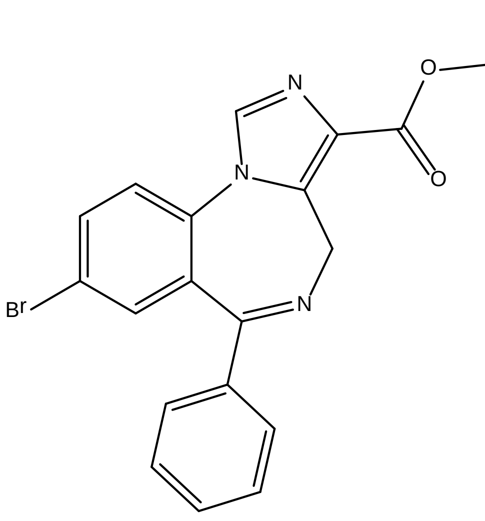 $C_{20}H_{16}BrN_3O_2$	XHE-II-012	49	24	31	1042	14	2038
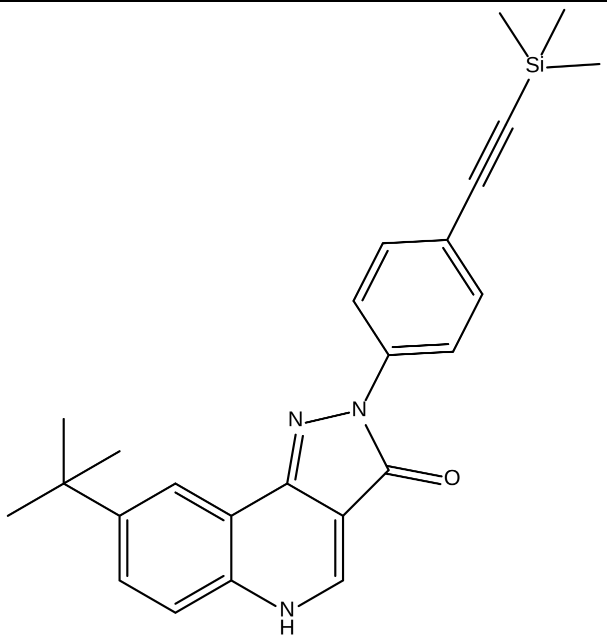 $C_{25}H_{27}N_3OSi$	XHE-II-014	329	1098	1156	1000	2462	1000

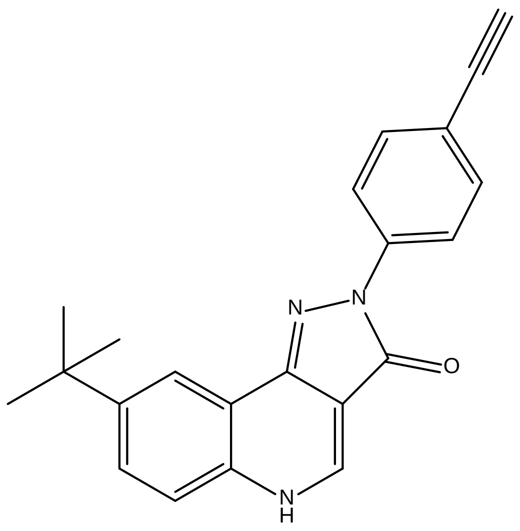 $C_{22}H_{19}N_3O$	XHE-II-017	3.3	10	7	258	17	294
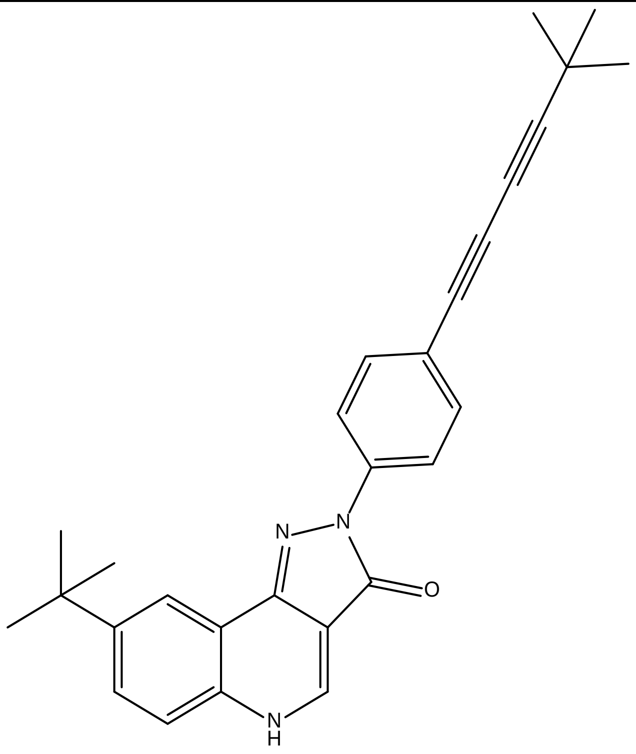 $C_{28}H_{27}N_3O$	XHE-II-019	273	428	762	1000	1464	1000
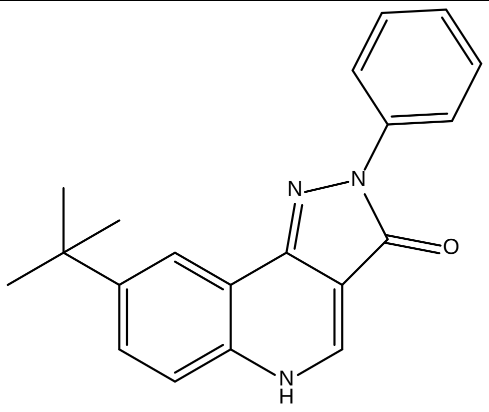 $C_{20}H_{19}N_3O$	XHE-II-024	0.09	0.18	0.32	14	0.24	11
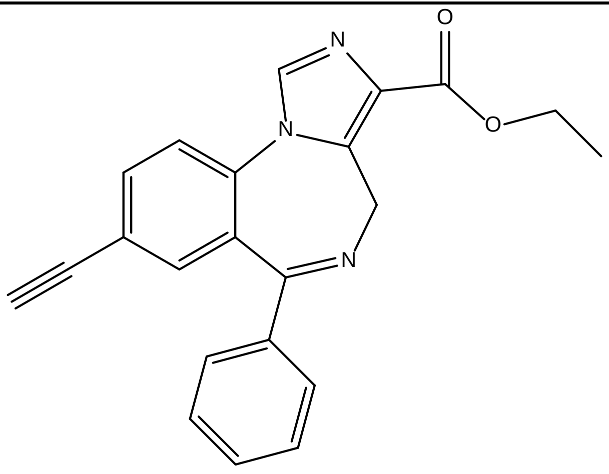 $C_{22}H_{17}N_3O_2$	XHE-II-053	287	45	96	1504	13.8	1000
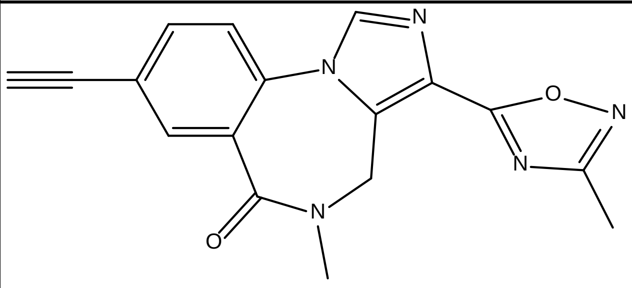 $C_{17}H_{13}N_5O_2$	XHE-II-065	1000	409	216	37	6.4	175
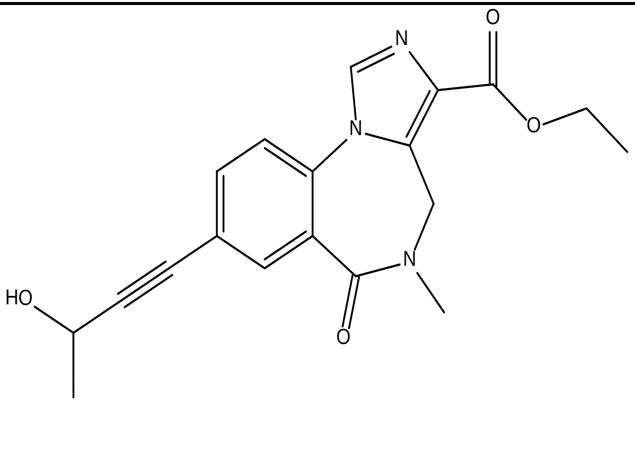 $C_{19}H_{19}N_3O_4$	XHE-II-070	352	532	454	45%	55	1000

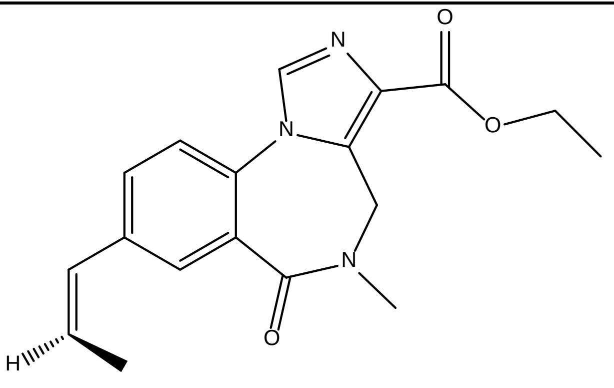 $C_{18}H_{19}N_3O_3$	XHE-II-073A (R ENRICHED)	5.9	11	10	15	1.18	140
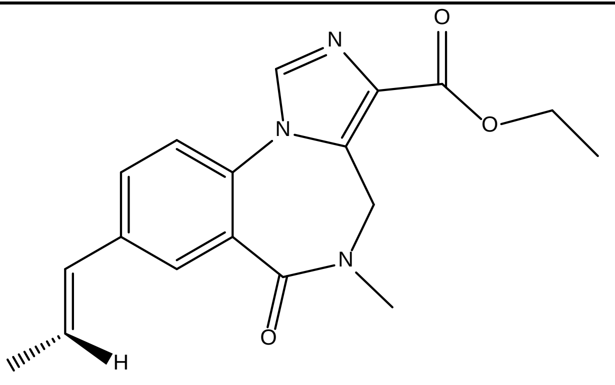 $C_{18}H_{19}N_3O_3$	XHE-II-073B (S-ENRICHED)	11	17	12	33	2.1	269
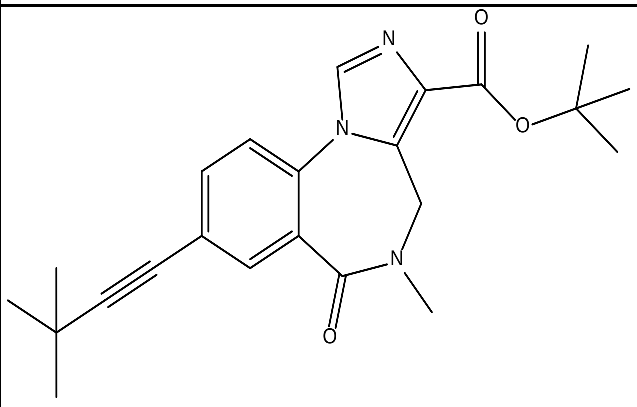 $C_{23}H_{27}N_3O_3$	XHE-II-077	1363	1000	3403	3561	1000	1000
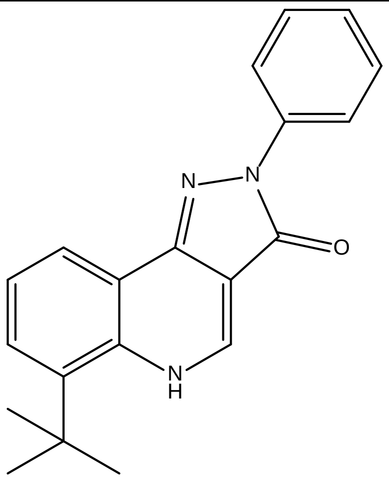 $C_{20}H_{19}N_3O$	XHE-II-087a	1604	1000	1000	1000	1000	1000
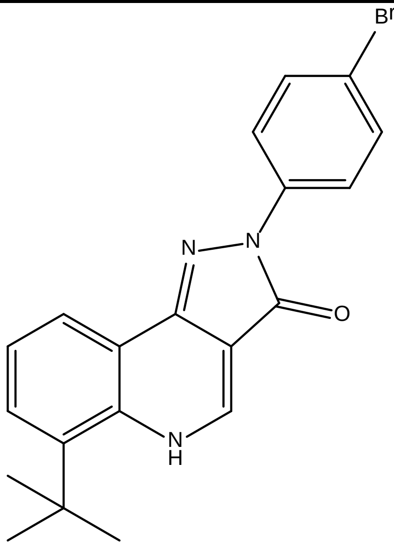 $C_{20}H_{18}BrN_3O$	XHE-II-087c	1000	1000	1000	1000	1000	1000
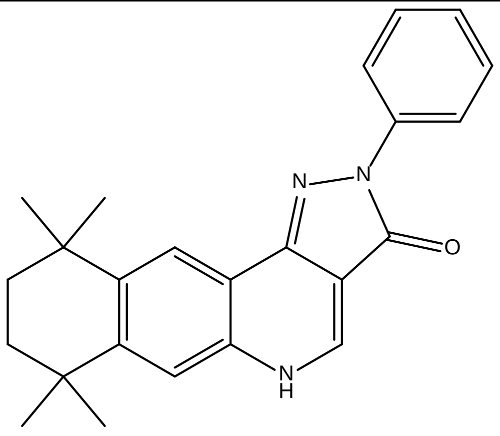 $C_{24}H_{25}N_3O$	XHE-II-098a	1000	1000	1000	1000	1000	1000

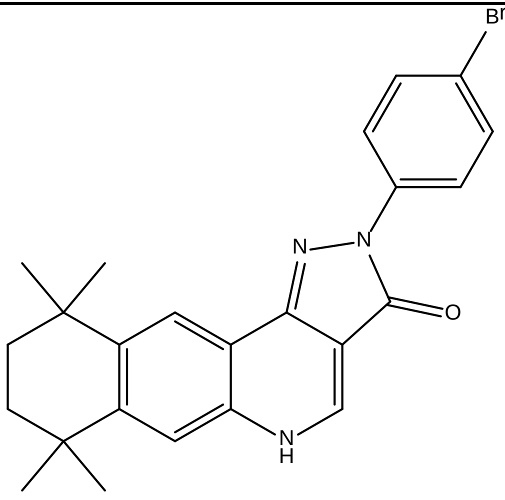 $C_{24}H_{24}BrN_3O$	XHE-II-098b	1000	1000	1000	1000	1000	1000
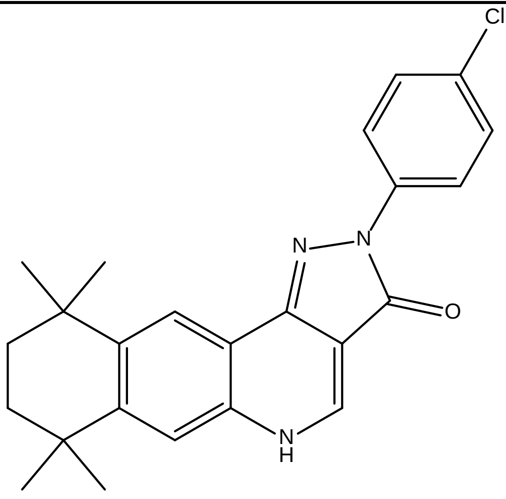 $C_{24}H_{24}ClN_3O$	XHE-II-098c	1000	1000	1000	1000	1000	1000
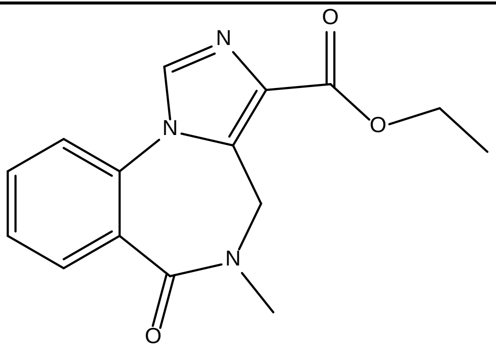 $C_{15}H_{15}N_3O_3$	XHE-III-04	1.2	2	1.1	219	0.4	500
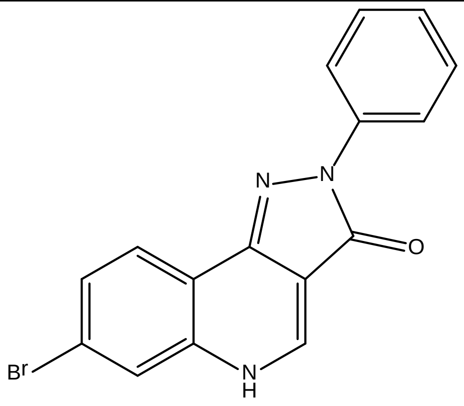 $C_{16}H_{10}BrN_3O$	XHE-III-06a	1	2	1	5	1.8	37
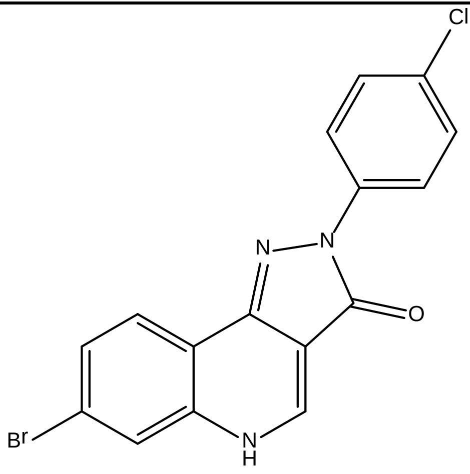 $C_{16}H_9BrClN_3O$	XHE-III-06b	32	33	20	299	28.6	740
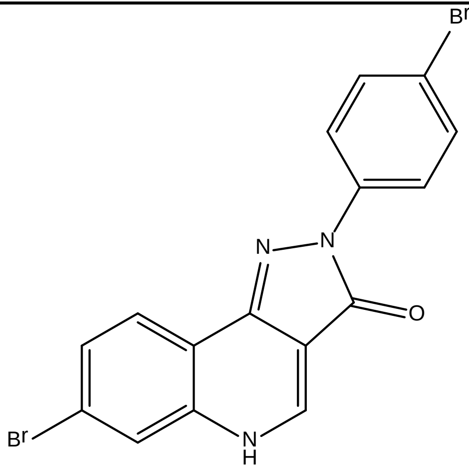 $C_{16}H_9Br_2N_3O$	XHE-III-06c	34	44	29	210	23	319

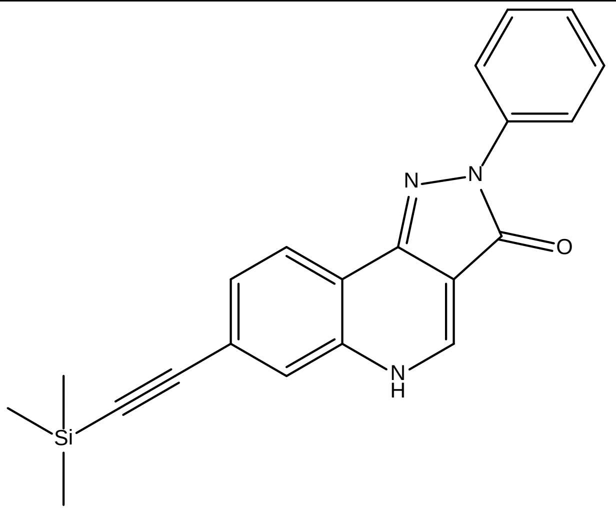 $C_{21}H_{19}N_3OSi$	XHE-III-12	240		1000	1000	181	536
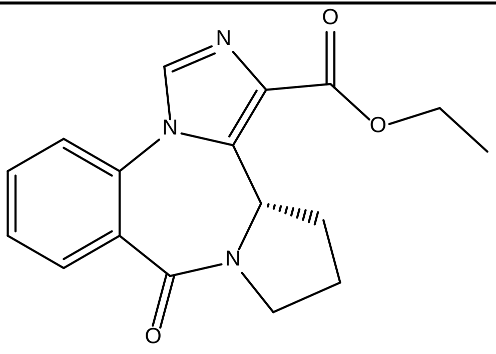 $C_{17}H_{17}N_3O_3$	XHE-III-13	7.3		7.1	880	1.6	311
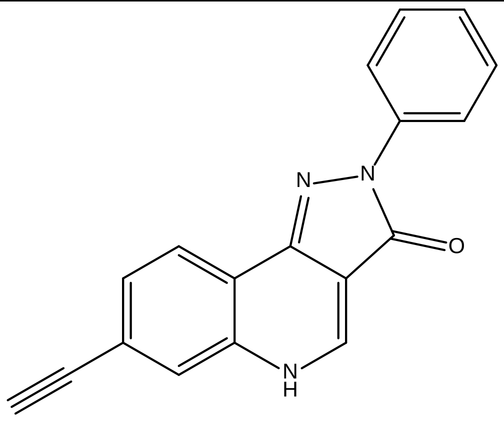 $C_{18}H_{11}N_3O$	XHE-III-14	2.6		10	13	2	7
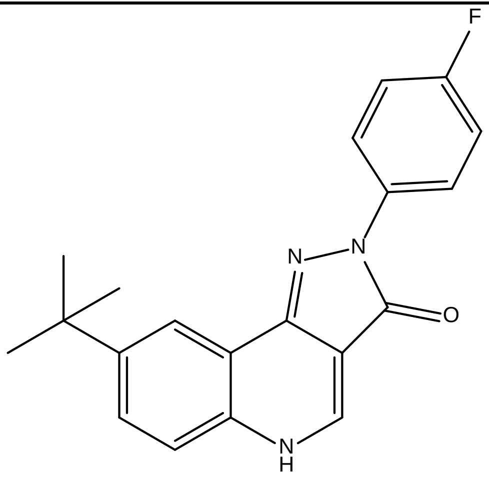 $C_{20}H_{18}FN_3O$	XHE-III-24	0.25		8	222	10	328
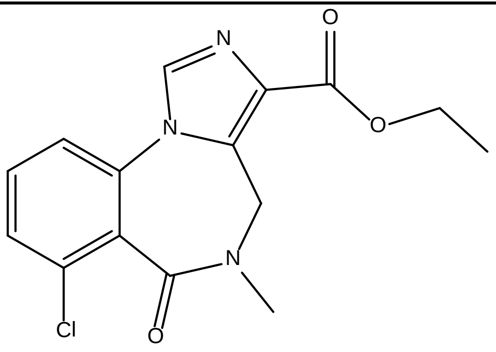 $C_{15}H_{14}ClN_3O_3$	XHE-III-49	1.3	5.5	4.2	38.7	11.3	85.1
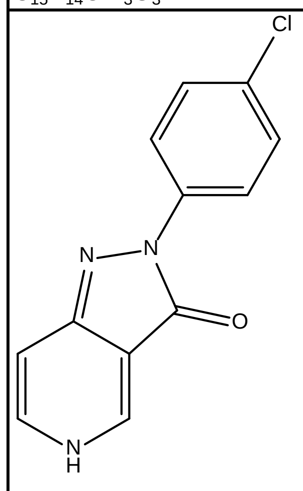 $C_{12}H_8ClN_3O$	XHE-III-54	1071	1624	2048	1000	1158	1000

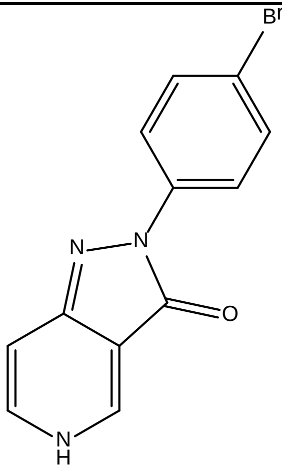 $C_{12}H_8BrN_3O$	XHE-III-56b	1005	1642	1928	1000	1300	1000
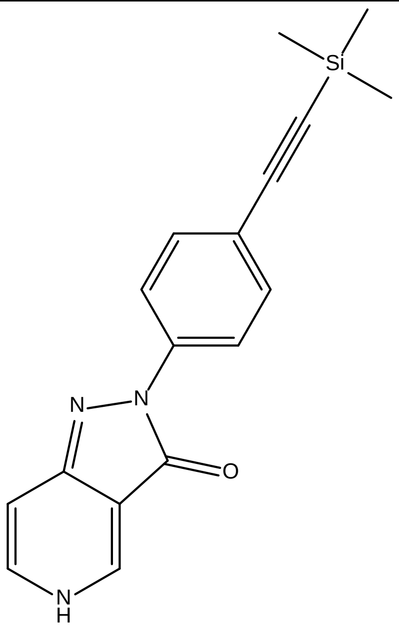 $C_{17}H_{17}N_3OSi$	XHE-III-67	1000	1000	1000	1000	1000	1000
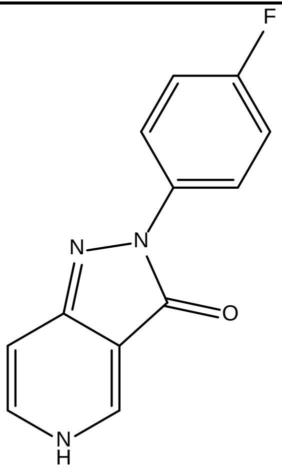 $C_{12}H_8FN_3O$	XHE-III-69	3433	1000	1000	1000	1000	1000
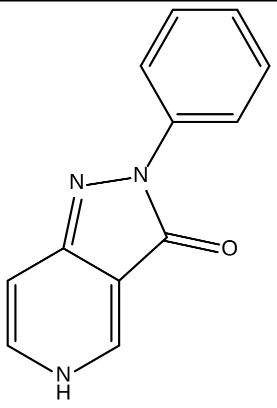 $C_{12}H_9N_3O$	XHE-III-70	997.5	2356	2484	711	500	1000
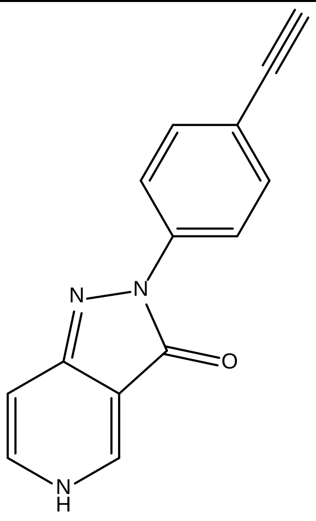 $C_{14}H_9N_3O$	XHE-III-73	1000	303	942	1000	492	1000
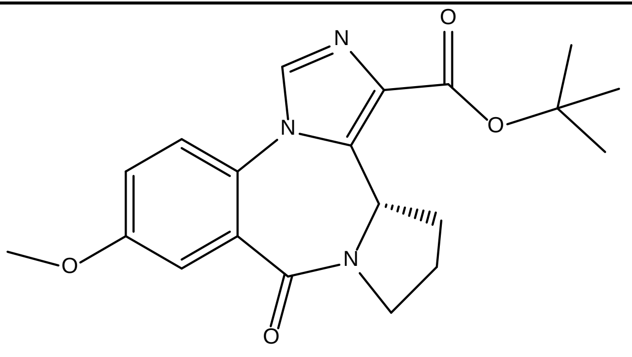 $C_{20}H_{23}N_3O_4$	XHE-III-74	77	105	38	0.42	2.2	5.8

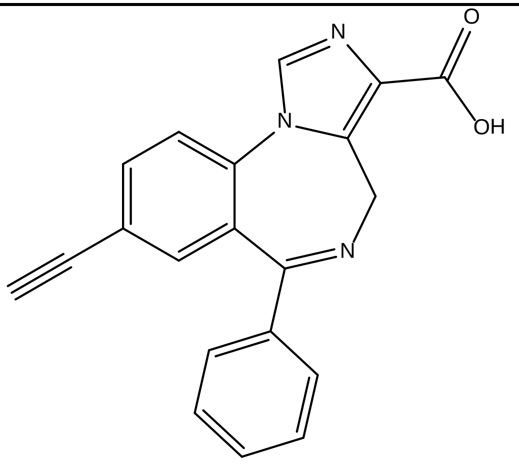 $C_{20}H_{13}N_3O_2$	XHE-II-O53-ACID	50.35	11.8	44		5.9	5000
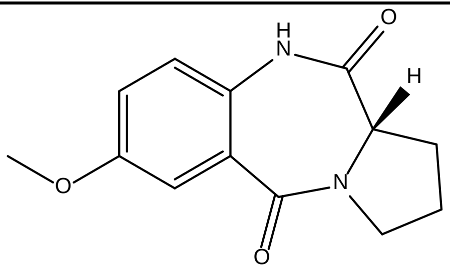 $C_{13}H_{14}N_2O_3$	XLI-?						
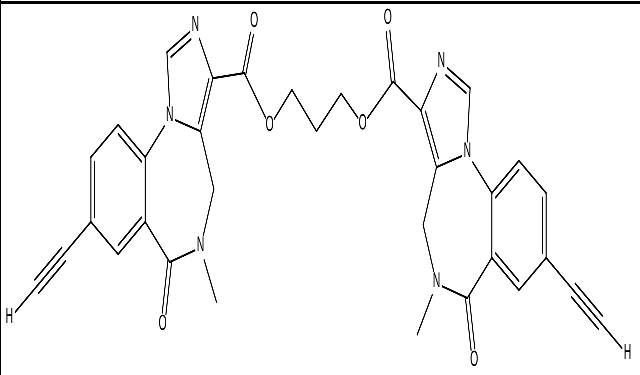 $C_{33}H_{26}N_6O_6$	XLI-093	1000	1000	858	1550	15	2000
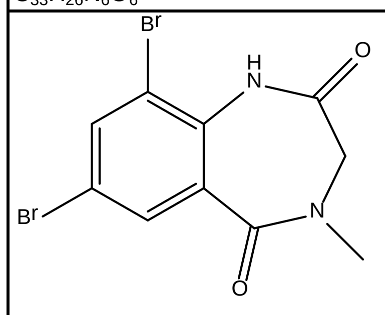 $C_{10}H_8Br_2N_2O_2$	XLI-1?						
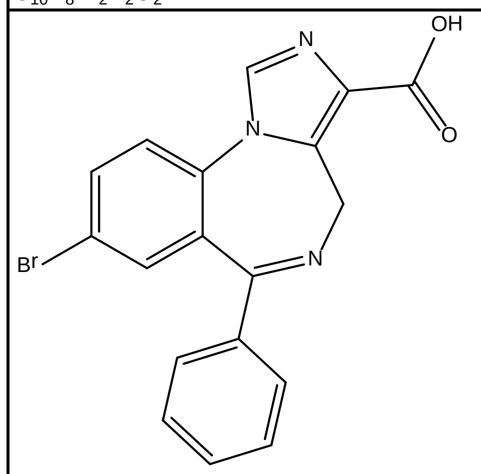 $C_{18}H_{12}BrN_3O_2$	XLI-12TC						
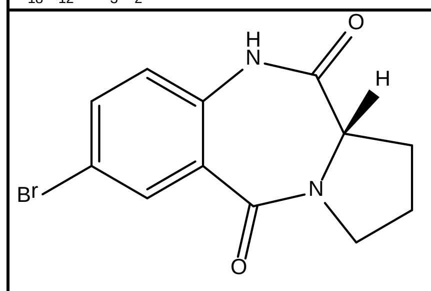 $C_{12}H_{11}BrN_2O_2$	XLI-13TC	2078	5289	618.7		8382	
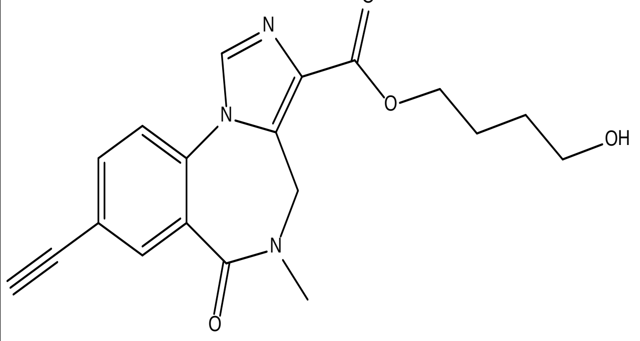 $C_{19}H_{19}N_3O_4$	XLI-14TC						

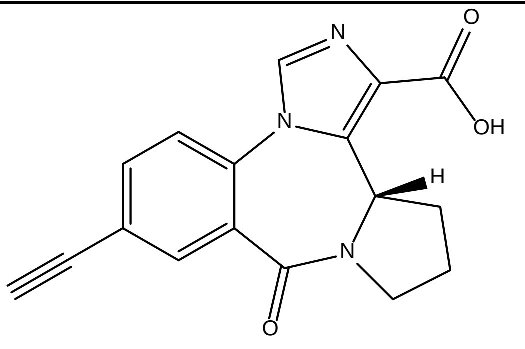 $C_{17}H_{13}N_3O_3$	XLI-15TC						
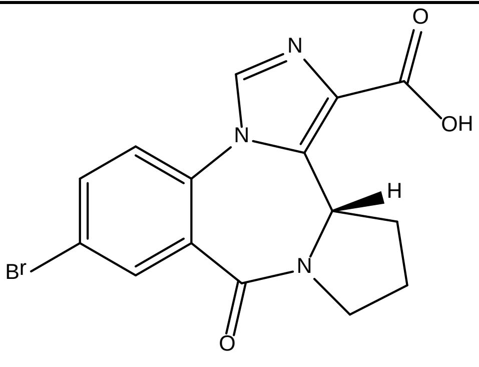 $C_{15}H_{12}BrN_3O_3$	XLI-16TC	7664	3515	787.4		1831	
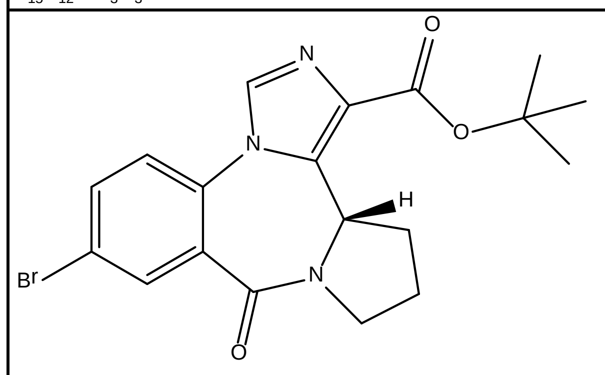 $C_{19}H_{20}BrN_3O_3$	XLI-17TC	224.8	106.6	120.6		92.43	
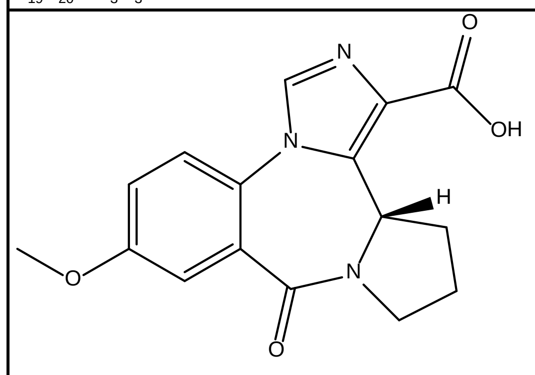 $C_{16}H_{15}N_3O_4$	XLI-18TC						
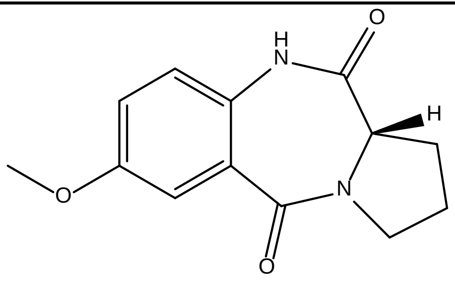 $C_{13}H_{14}N_2O_3$	XLI-19TC						
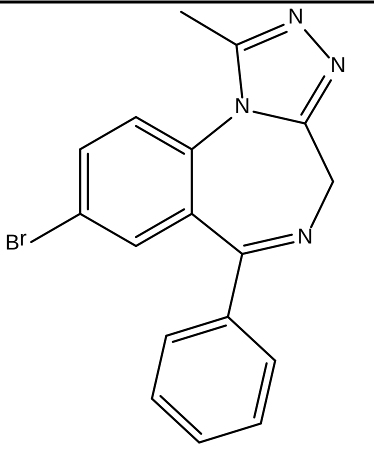 $C_{17}H_{13}BrN_4$	XLI-2?						
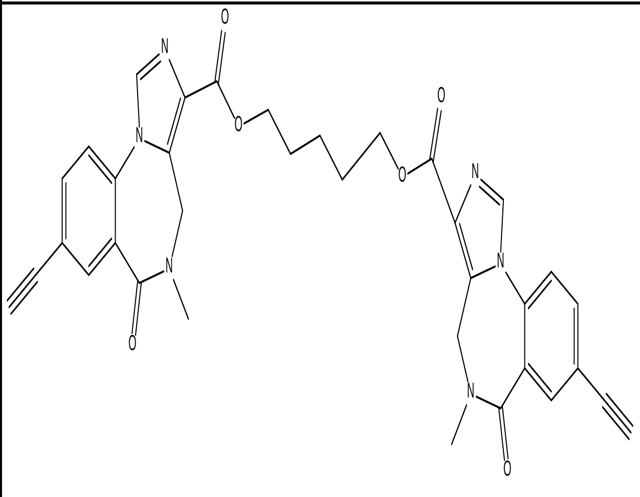 $C_{35}H_{30}N_6O_6$	XLI-210	231	661	2666		5.4	54.22

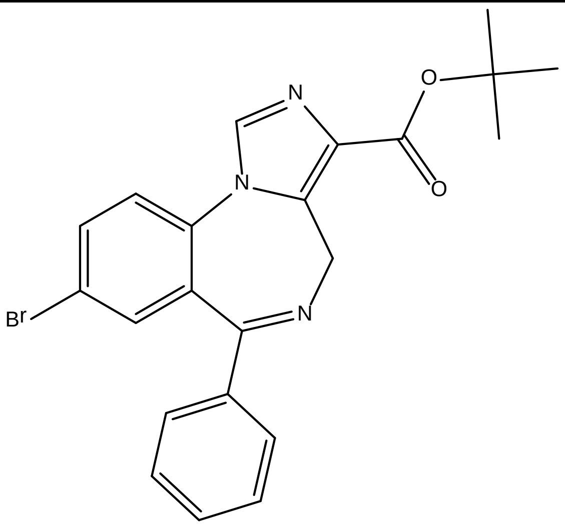 $C_{22}H_{20}BrN_3O_2$	XLi223	C22H20BrN3O2	14	8.7	18	1000	10	2000
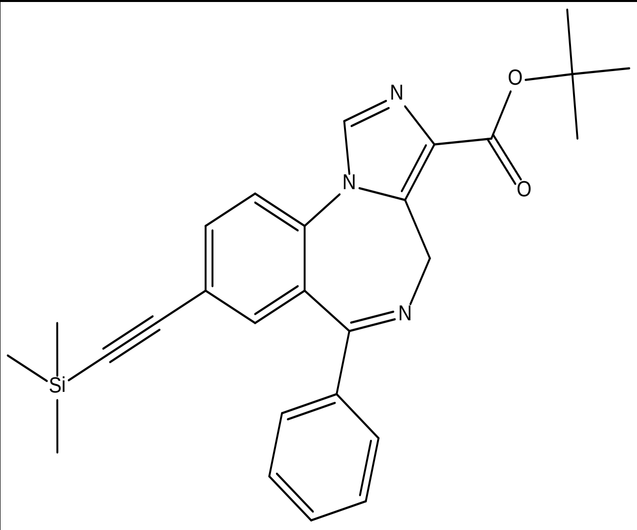 $C_{27}H_{29}N_3O_2Si$	XLi224	C27H29N3O2Si	333	333	333	3000	333	2000
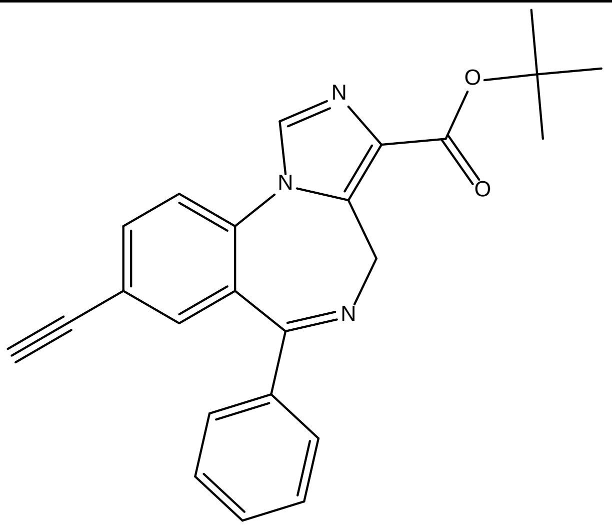 $C_{24}H_{21}N_3O_2$	XLi225	C24H21N3O2	333	225	174	3000	110	2000
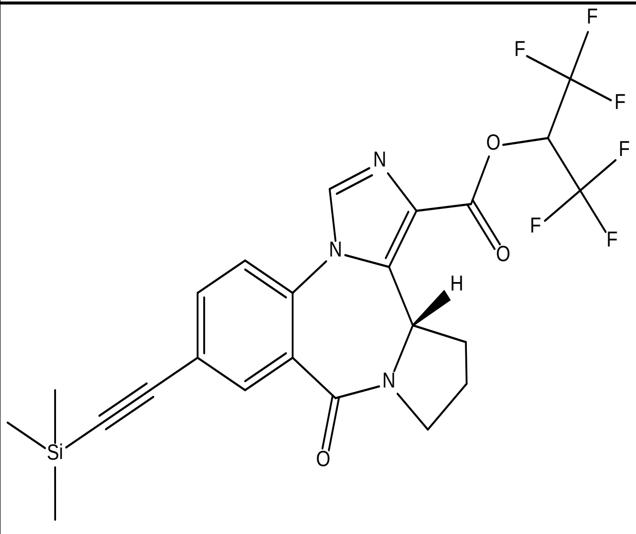 $C_{23}H_{21}F_6N_3O_3Si$	XLI-232							
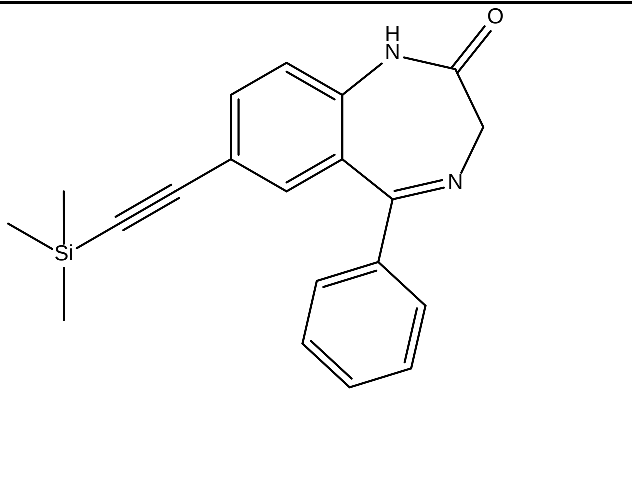 $C_{20}H_{20}N_2OSi$	XLI-250 (SHU-221)		1000	1000	339	1000	118	1000
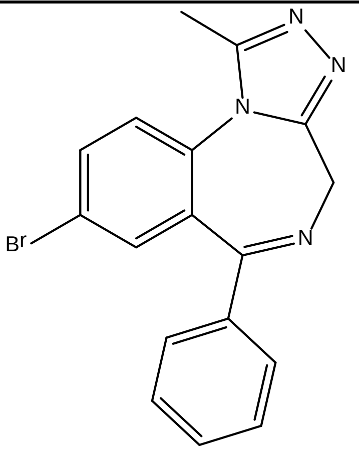 $C_{17}H_{13}BrN_4$	XLi268	C17H13BrN4	2.81	0.69			0.62	

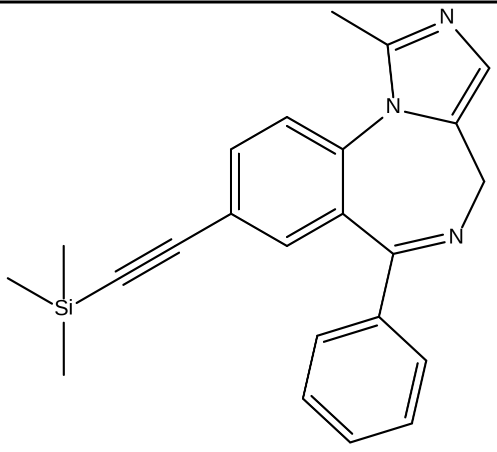 $C_{23}H_{23}N_3Si$	XLi269 C22H22N4Si	221.8	154.2			15.51	
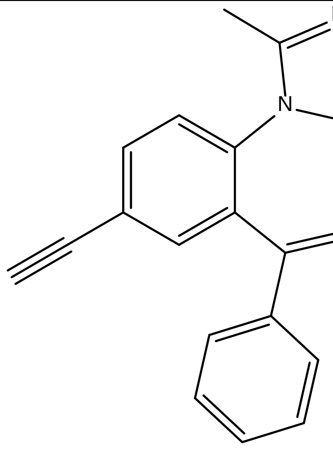 $C_{19}H_{14}N_4$	XLi270 C19H14N4	36.39	25.81			5.291	
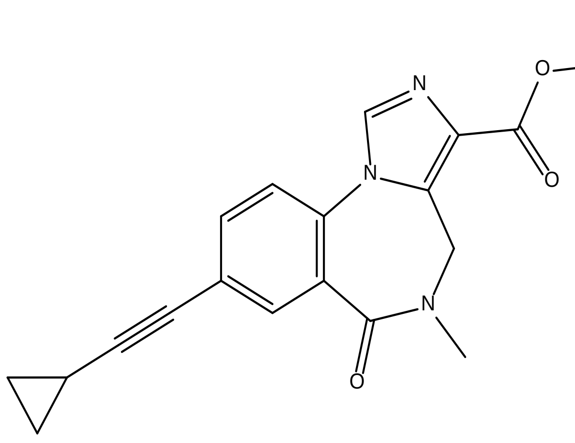 $C_{20}H_{19}N_3O_3$	XLi275 C20H19N3O3	35.61					
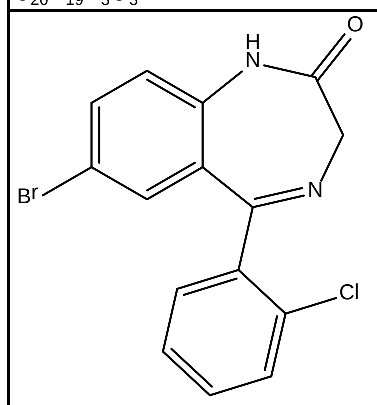 $C_{15}H_{10}BrClN_2O$	XLI-286	0.051	0.064	0.118		0.684	
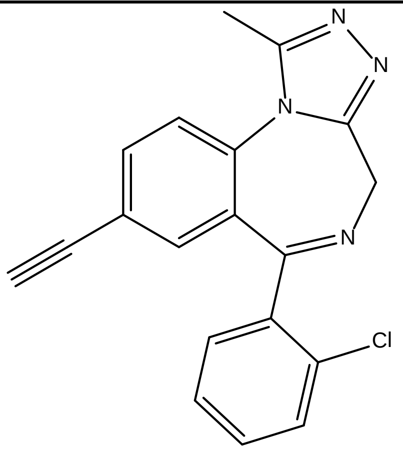 $C_{19}H_{13}ClN_4$	xli296						
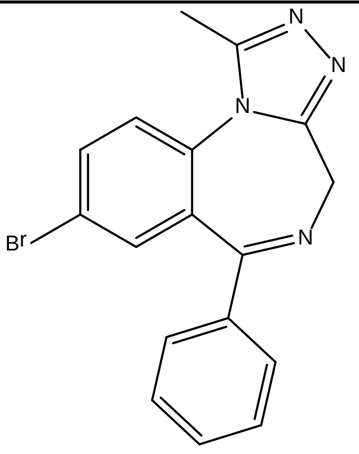 $C_{17}H_{13}BrN_4$	XLI-2TC	3.442	1.673	44.08		1.121	
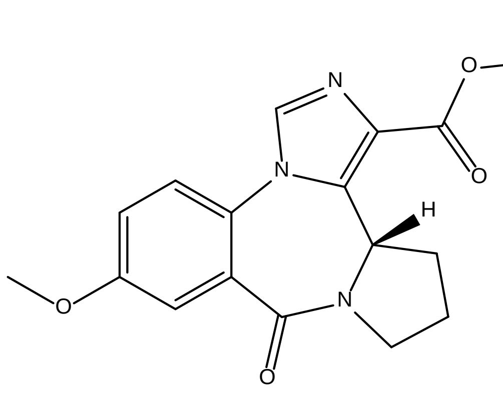 $C_{18}H_{19}N_3O_4$	XLI-317	60.24	24.05	4.562		0.295	

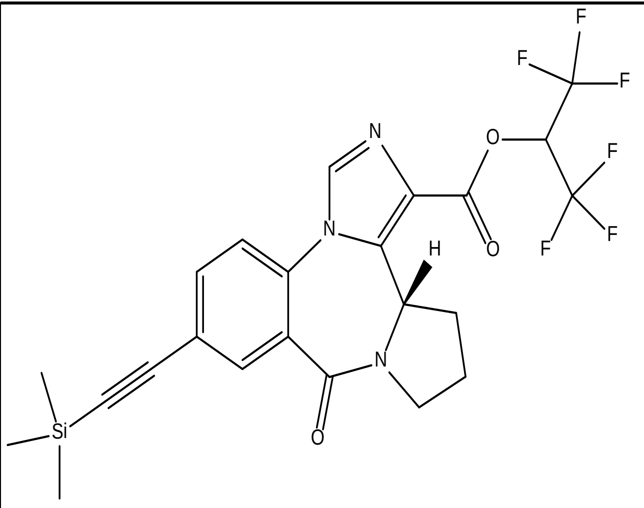 $C_{23}H_{21}F_6N_3O_3Si$	XLI-332	1390	1507	3405		132.1	
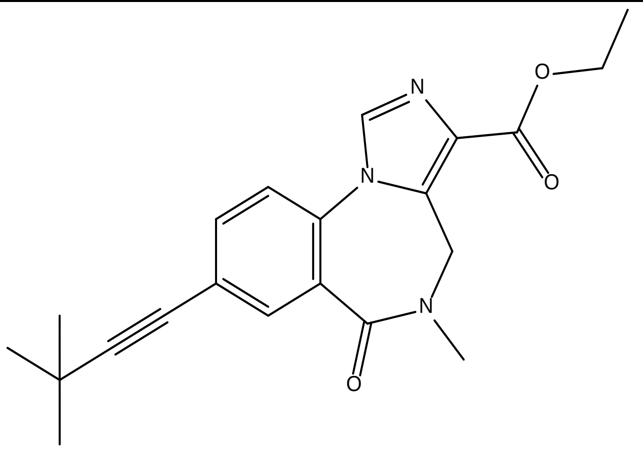 $C_{21}H_{23}N_3O_3$	XLi337 C21H23N3O3						
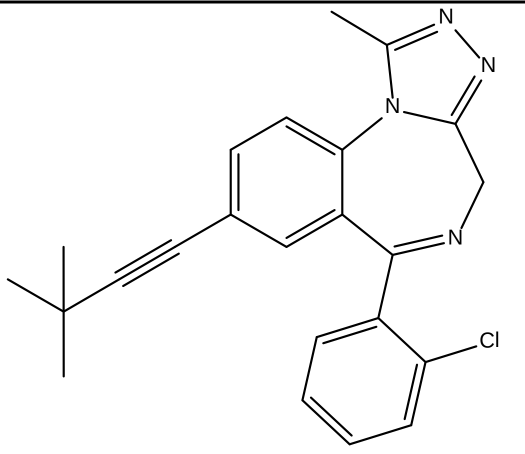 $C_{23}H_{21}ClN_4$	XLi338 C23H21ClN4	380	189	692		5000	5000
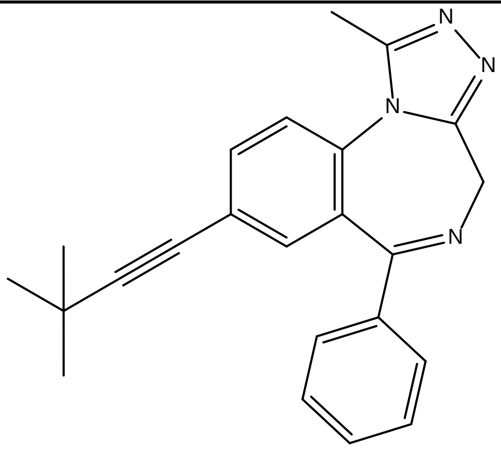 $C_{23}H_{22}N_4$	XLi339 C23H22N4	5000	625	5000		5000	5000
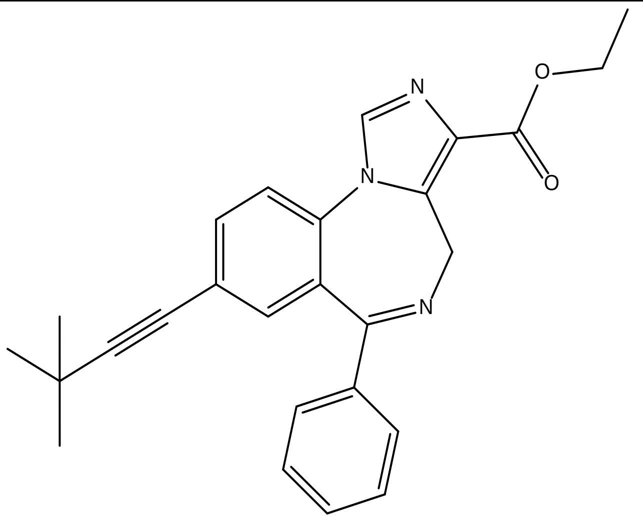 $C_{26}H_{25}N_3O_2$	XLi340 C26H25N3O2						
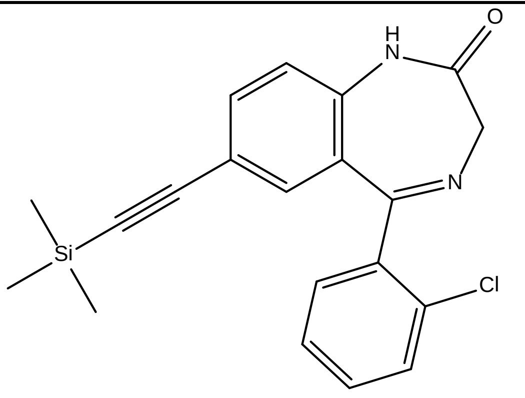 $C_{20}H_{19}ClN_2OSi$	XLi343 C20H19ClN2OSi	6.4	17.7			150.5	

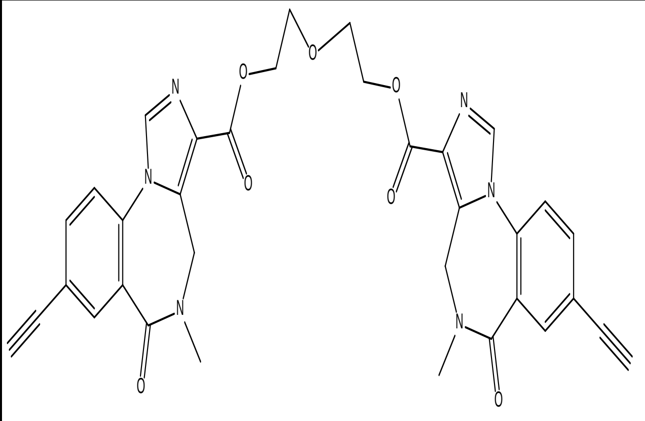 $C_{34}H_{28}N_6O_7$	Xli-347 C34H28N6O7	828.05	690.2			13.87	
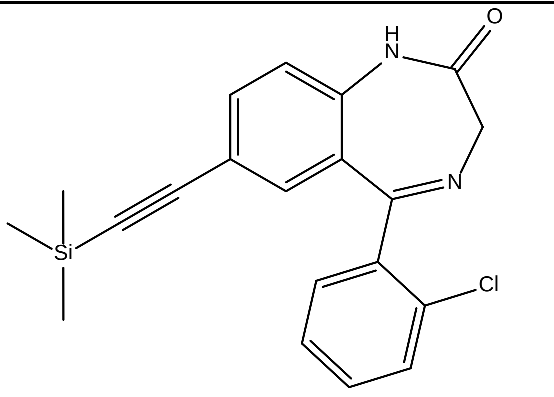 $C_{20}H_{19}ClN_2OSi$	XLI-348	13.56	11.17	1.578		82.05	
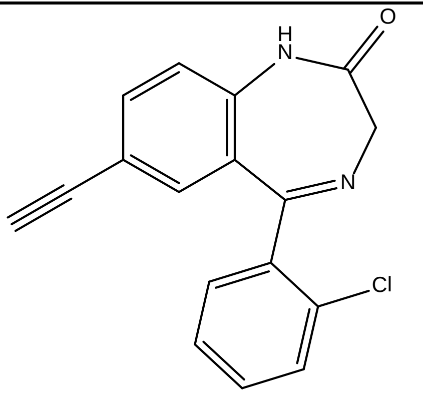 $C_{17}H_{11}ClN_2O$	XLi350 C17H11ClN2O	1.224	1.188			2.9	
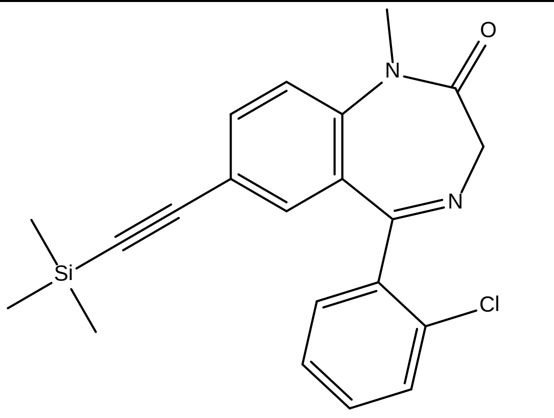 $C_{21}H_{21}ClN_2OSi$	XLi351 C21H21ClN2OSi	1.51	0.97			1.98	
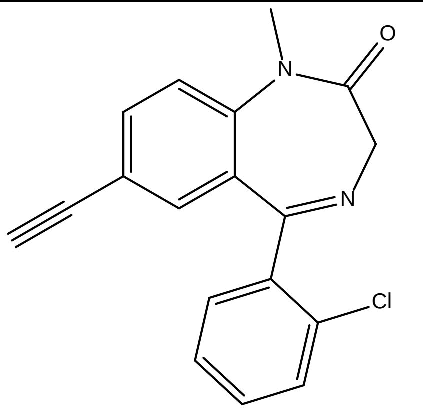 $C_{18}H_{13}ClN_2O$	XLi352 C18H13ClN2O	1.56	0.99			1.96	
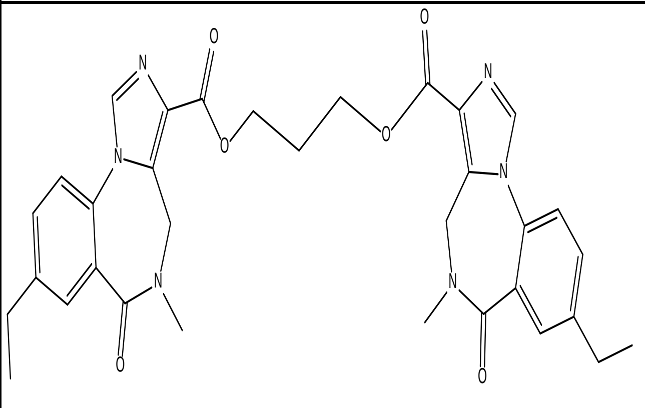 $C_{33}H_{34}N_6O_6$	XLi356 C33H34N6O6	1851.5	4702.5	8545		100.5	5000
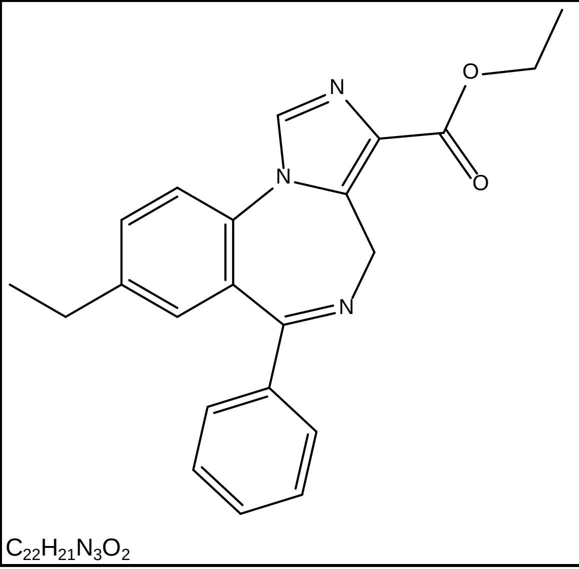 $C_{22}H_{21}N_3O_2$	Xli366 C22H21N3O2	1					

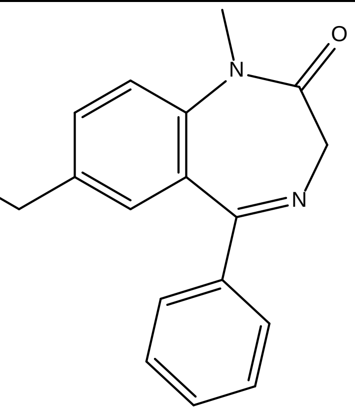 $C_{18}H_{18}N_2O$	XLI368 C24H25N302						
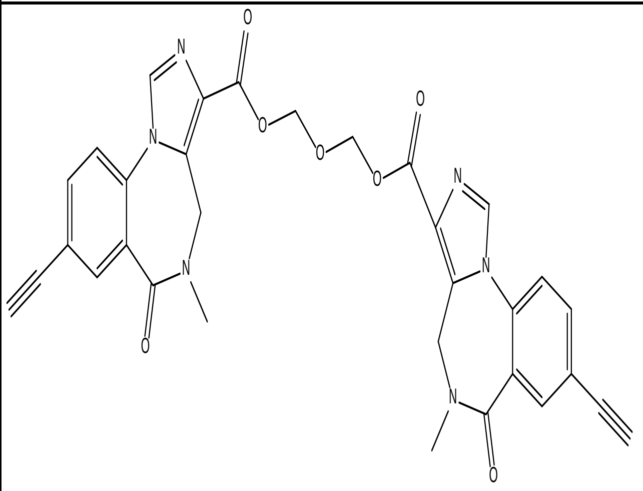 $C_{32}H_{24}N_6O_7$	XLI-374	3795	2694	1864		76.14	
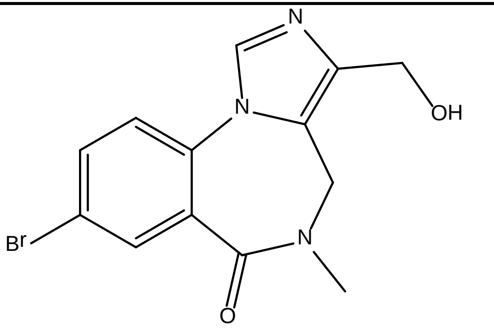 $C_{13}H_{12}BrN_3O_2$	XLI-379	1037	482.4	357.5		36.13	
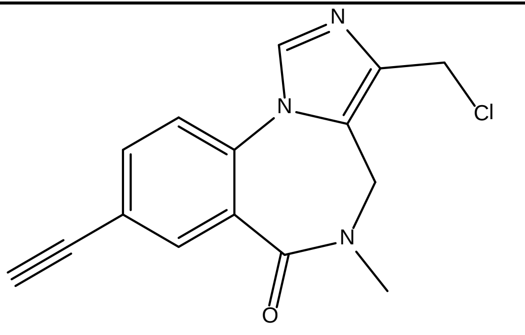 $C_{15}H_{12}ClN_3O$	XLI-381	619.9	285.6	3639		7.1	
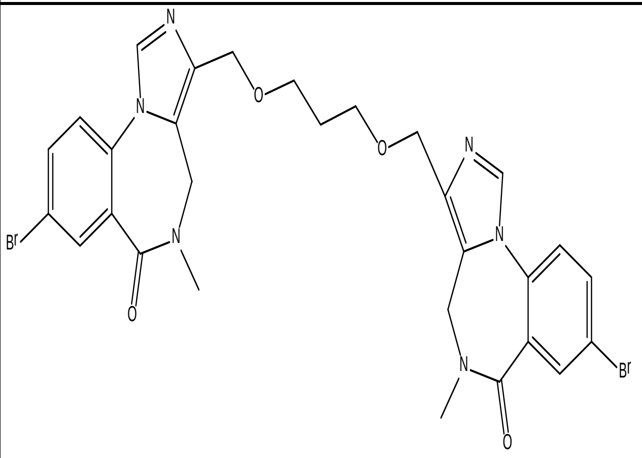 $C_{29}H_{28}Br_2N_6O_4$	XLI-382						
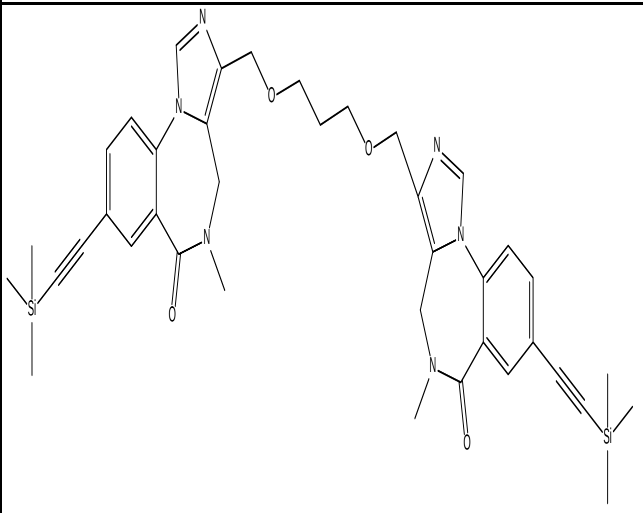 $C_{39}H_{46}N_6O_4Si_2$	XLI-385						

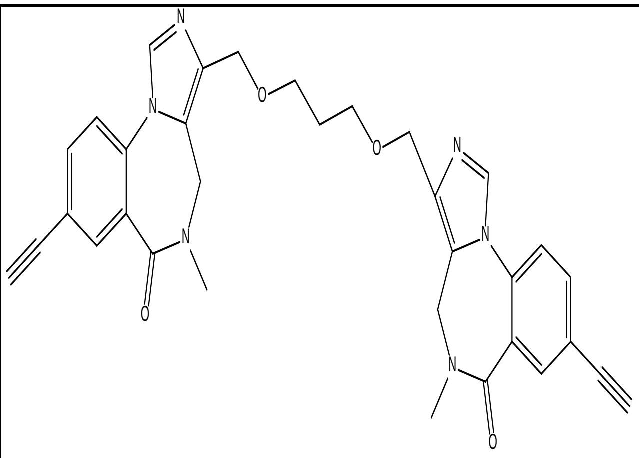 $C_{33}H_{30}N_6O_4$	XLI-387	5454	5000	5000		5000	5000
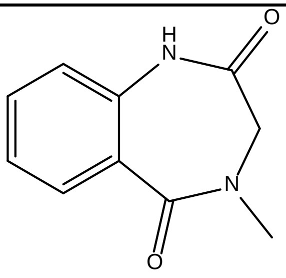 $C_{10}H_{10}N_2O_2$	XLI-4?						
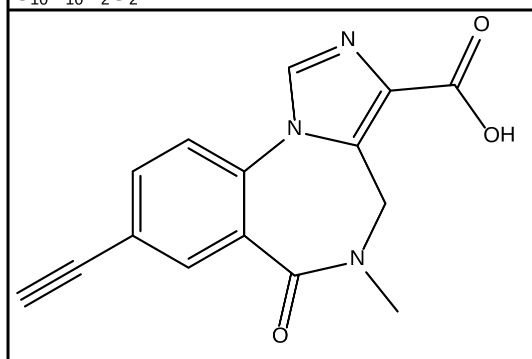 $C_{15}H_{11}N_3O_3$	XLI-6TC	2371	1421	1840		1169	
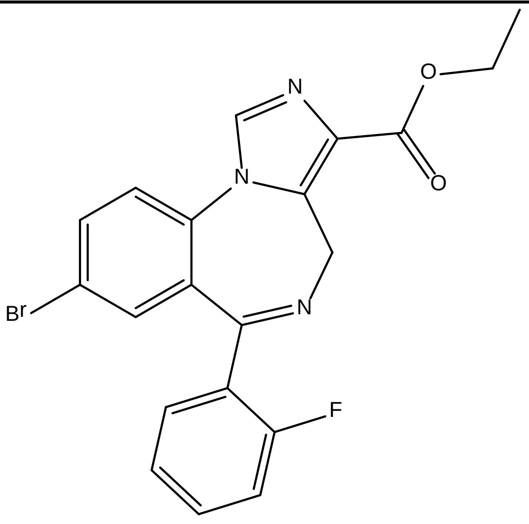 $C_{20}H_{15}BrFN_3O_2$	XLI-8TC	21.52	11.01	2.16		4.06	
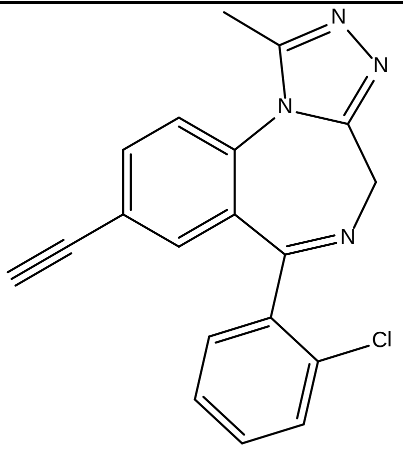 $C_{19}H_{13}ClN_4$	XLi-JY-DMH ANX3	3.3	0.6	1.9		4.4	5000
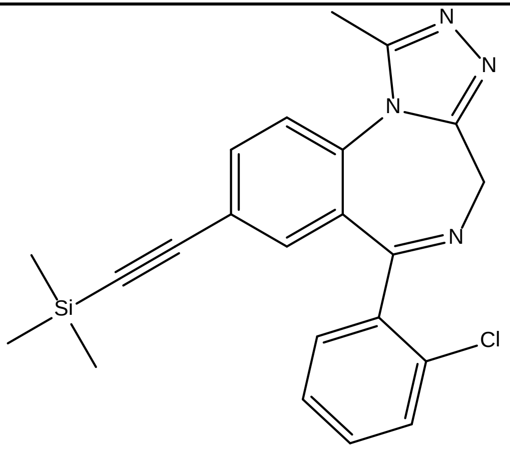 $C_{22}H_{21}ClN_4Si$	XLi-JY-DMH-TMS C22H21N4SiCl)	35	63	99.13		69.7	5000

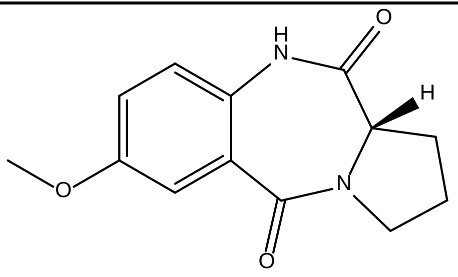 $C_{13}H_{14}N_2O_3$	XLI-TC	8155	7528	1036		508.9	
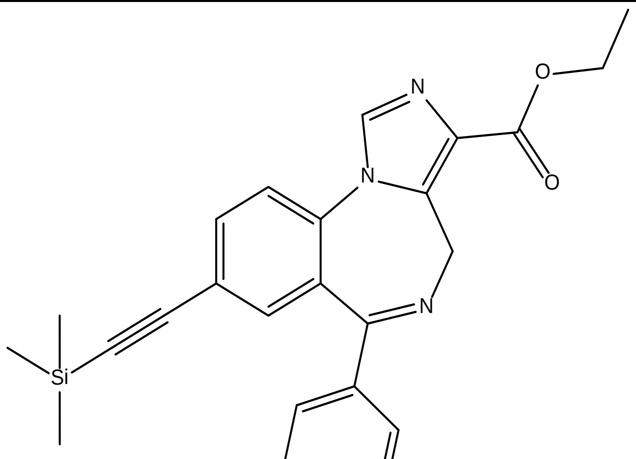 $C_{25}H_{25}N_3O_2Si$	XliXHe-II-048 C25H25N3O2Si	5000	5000	5000		5000	
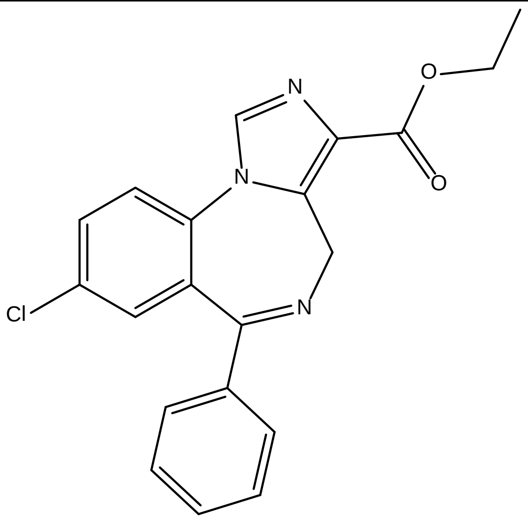 $C_{20}H_{16}ClN_3O_2$	XZ112						
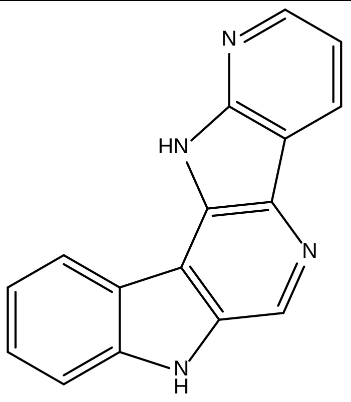 $C_{16}H_{10}N_4$	YCT-5	2.2	11.46	16.3		200	10000
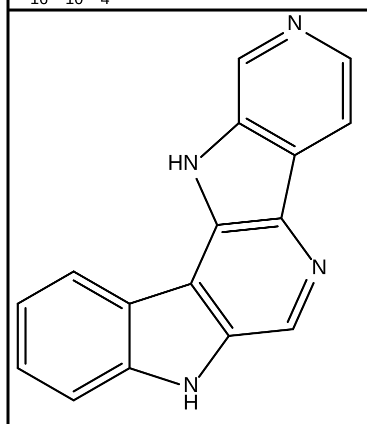 $C_{16}H_{10}N_4$	YCT-7A	3	23.8	30.5		240	10000
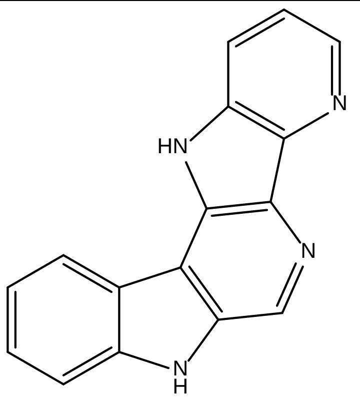 $C_{16}H_{10}N_4$	YCT-7B	28	118	156		1000	10000

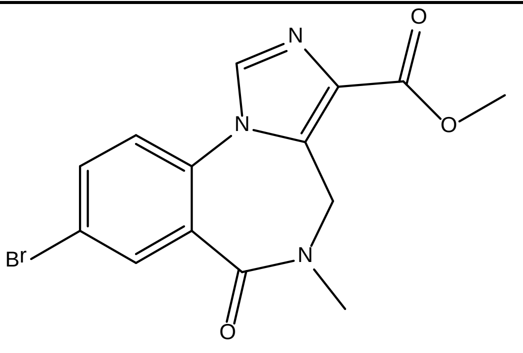 $C_{14}H_{12}BrN_3O_3$	YT-5	0.421	0.6034	36.06		1.695	
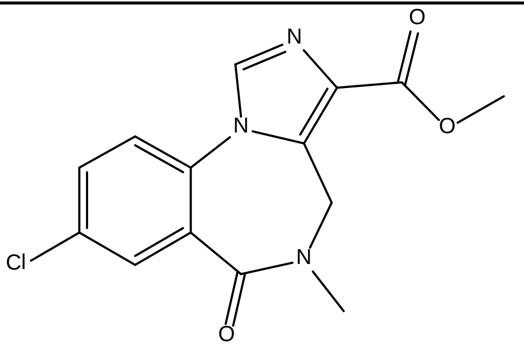 $C_{14}H_{12}ClN_3O_3$	YT-6	15.31	87.8	60.49		1.039	
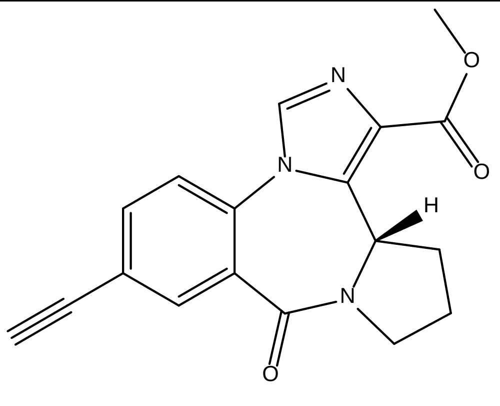 $C_{18}H_{15}N_3O_3$	YT-I-38	945.9	326.8	245.9		4.07	
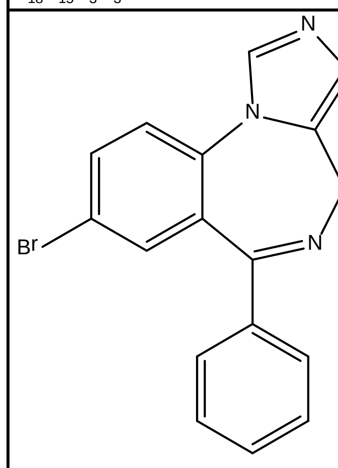 $C_{18}H_{14}BrN_3O$	YT-II	6.93	0.87	3.51		5.12	
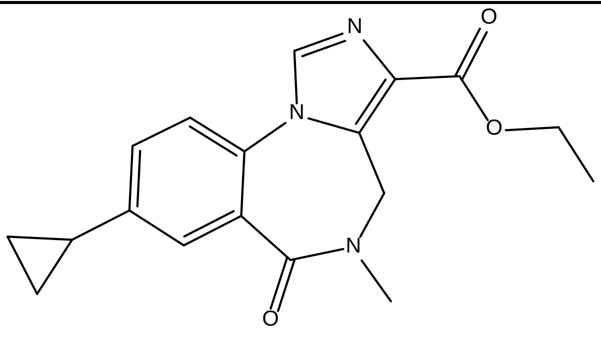 $C_{18}H_{19}N_3O_3$	YT-II-76	95.34	2.8	0.05		0.04	
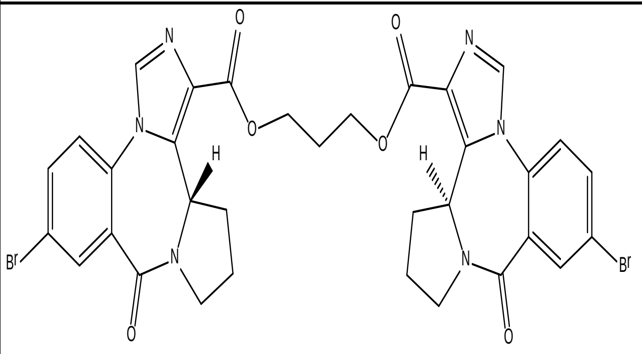 $C_{33}H_{28}Br_2N_6O_6$	YT-II-791	1495	2681	527.4		144	
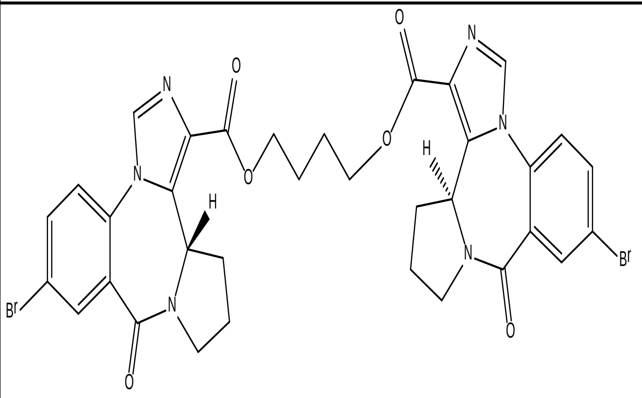 $C_{34}H_{30}Br_2N_6O_6$	YT-II-792	789.6	599.5	1698		83.18	

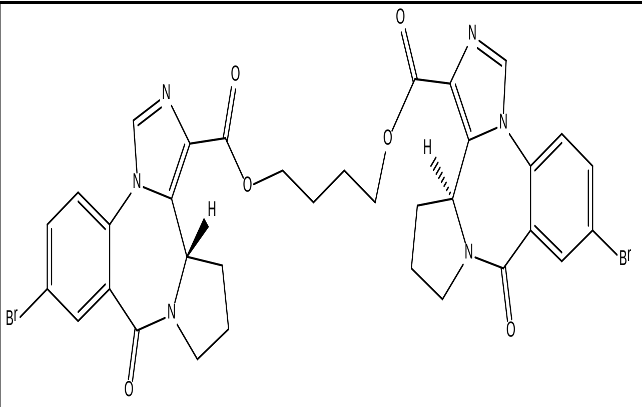 $C_{34}H_{30}Br_2N_6O_6$	YT-II-793	1383	2881	782.6		128.9	
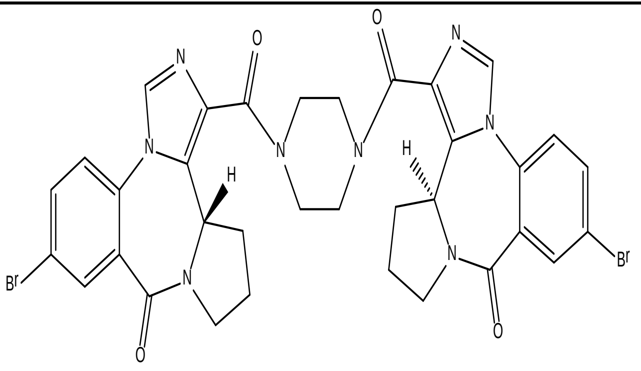 $C_{34}H_{30}Br_2N_8O_4$	YT-II-794	4536	126.2	4981		932.8	
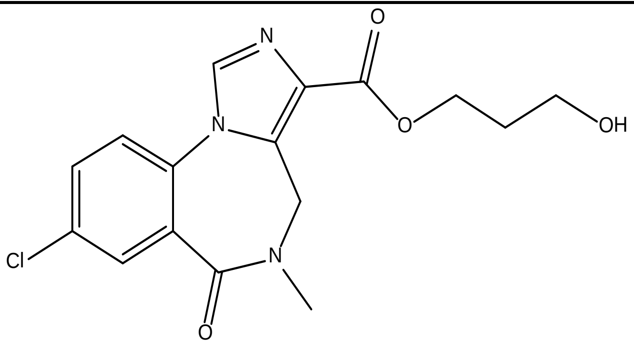 $C_{16}H_{16}ClN_3O_4$	YT-II-83	32.74	13.22	24.1		3.548	
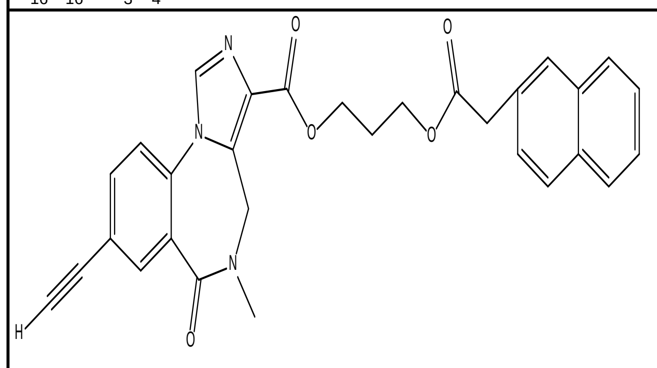 $C_{30}H_{25}N_3O_5$	YT-III-1						
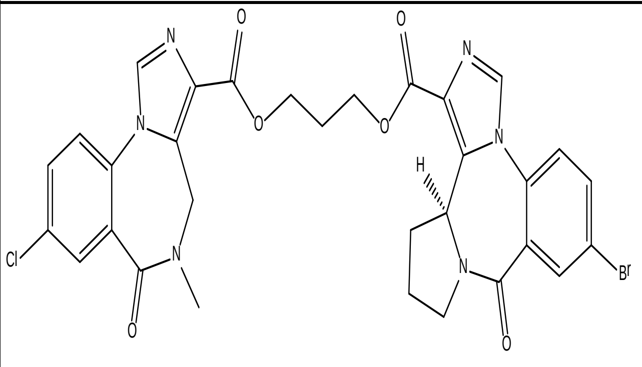 $C_{31}H_{26}BrClN_6O_6$	YT-III-10	1172	1915	1481		282.9	
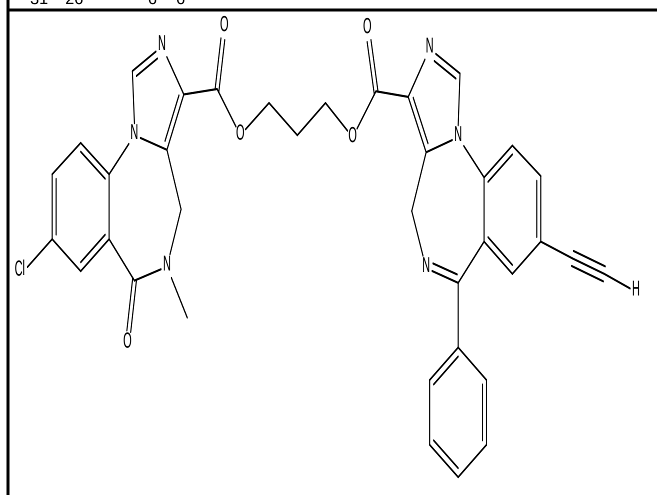 $C_{36}H_{27}ClN_6O_5$	YT-III-15	73.19	90.45	141.4		114	

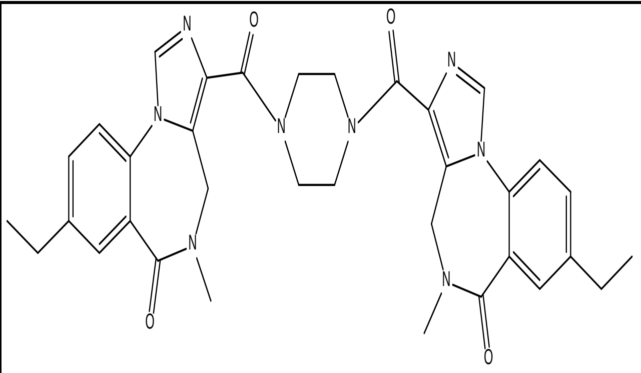 $C_{34}H_{36}N_8O_4$	YT-III-19	5329	182.4	3817		3313	
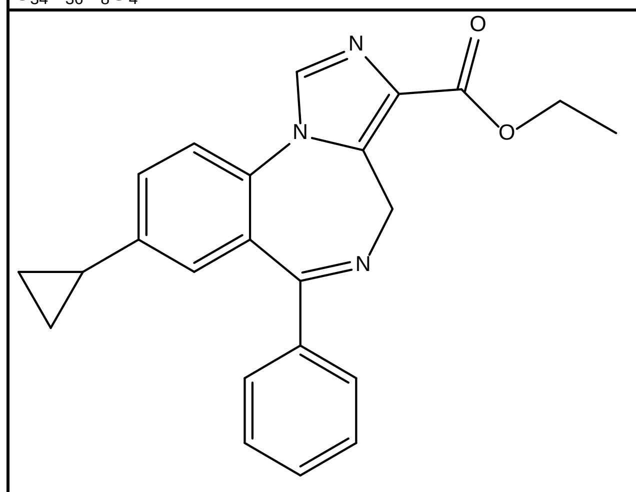 $C_{23}H_{21}N_3O_2$	YT-III-23	19.83	23.65	19.87		1.105	
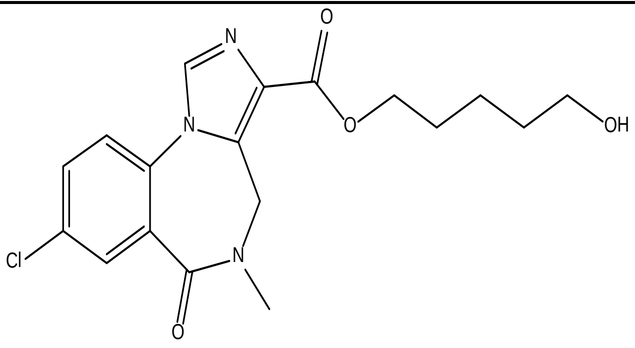 $C_{18}H_{20}ClN_3O_4$	YT-III-25	2.53	5.79	5.69		0.09	
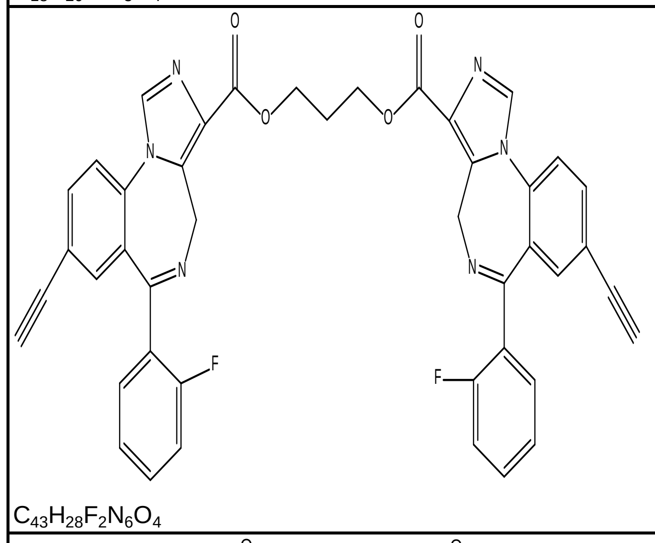 $C_{43}H_{28}F_2N_6O_4$	YT-III-271	32.54	1.26	2.35		103	
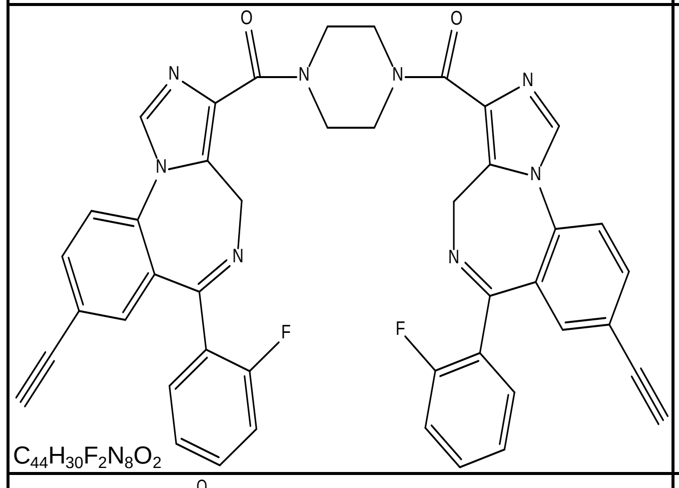 $C_{44}H_{30}F_2N_8O_2$	YT-III-272	295.9	14.98	10.77		103.3	
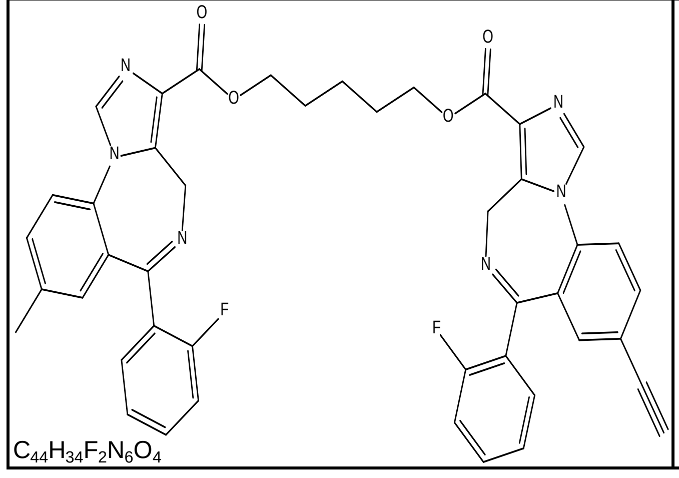 $C_{44}H_{34}F_2N_6O_4$	YT-III-273	245.1	366	389		879.4	

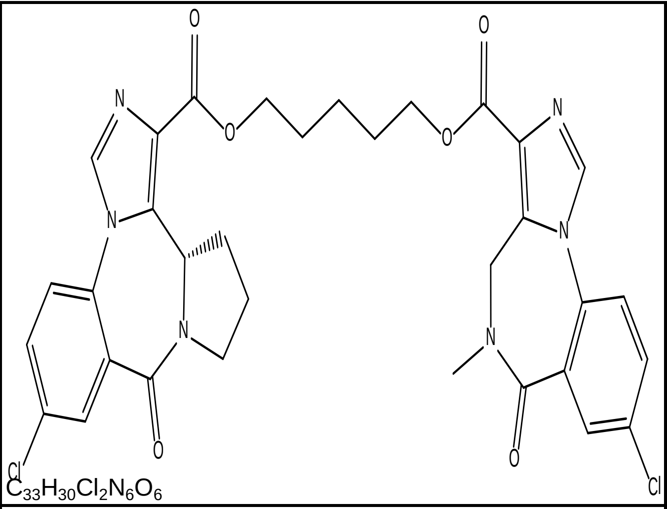 $C_{33}H_{30}Cl_2N_6O_6$	YT-III-28	130.7	80.03	82.9		192	
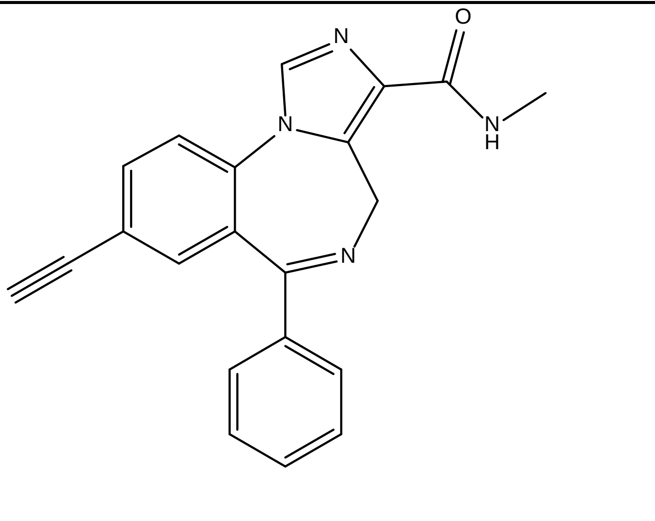 $C_{21}H_{16}N_4O$	YT-III-31	36.39	67.85	129.7		7.59	
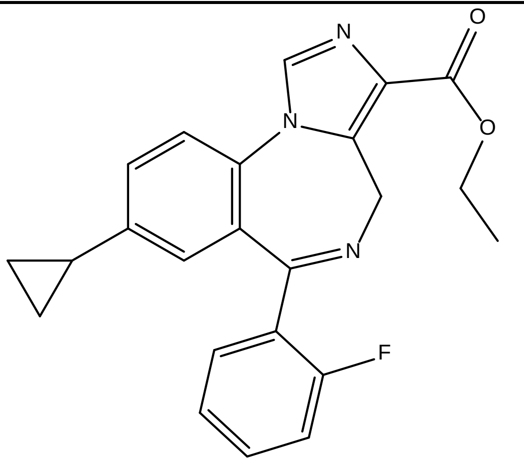 $C_{23}H_{20}FN_3O_2$	YT-III-38	1461	18.21	14.63		3999	
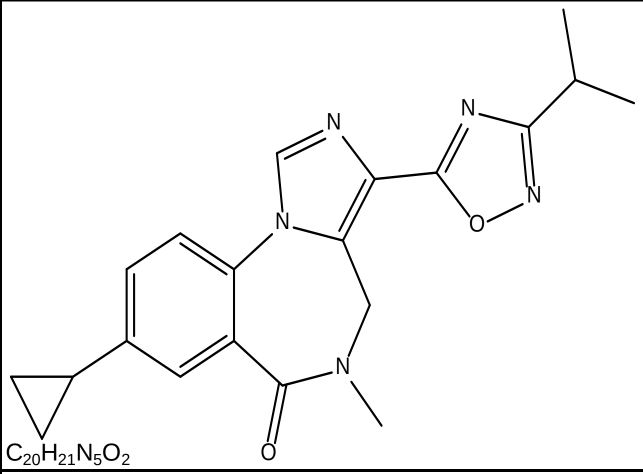 $C_{20}H_{21}N_5O_2$	YT-III-42	382.9	16.83	44.04		9.77	
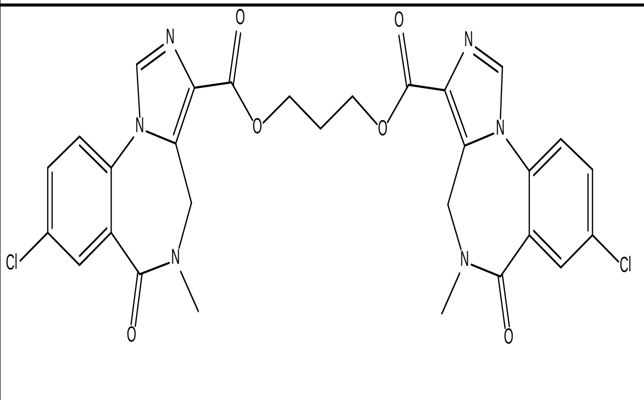 $C_{29}H_{24}Cl_2N_6O_6$	YT-TC-1	441.8	927.4	157.9		122.2	
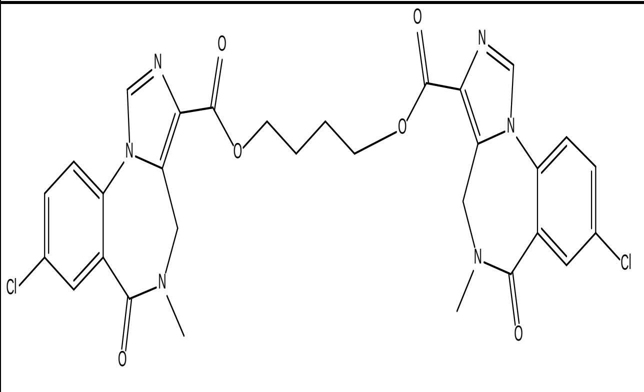 $C_{30}H_{26}Cl_2N_6O_6$	YT-TC-2	839.1	1647	568.1		421.9	

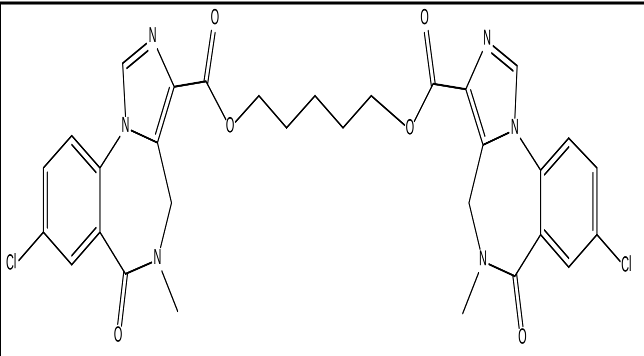 $C_{31}H_{28}Cl_2N_6O_6$	YT-TC-3	141.4	11.43	118.1		29.22	
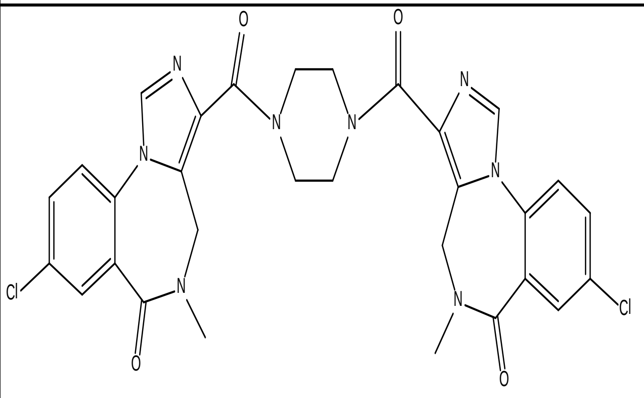 $C_{30}H_{26}Cl_2N_8O_4$	YT-TC-4	6291	3315	352.2		2881	
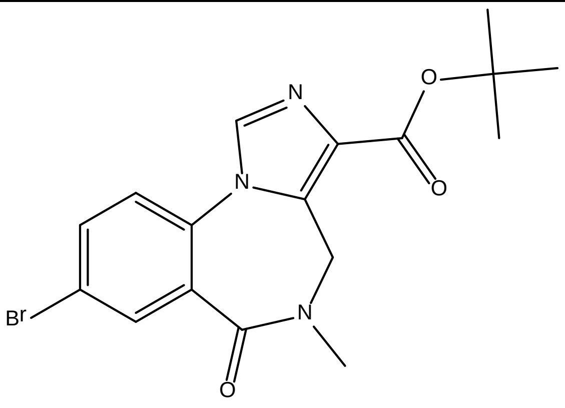 $C_{17}H_{18}BrN_3O_3$	ZG-168	11.2	10.7	9.2		0.47	9.4
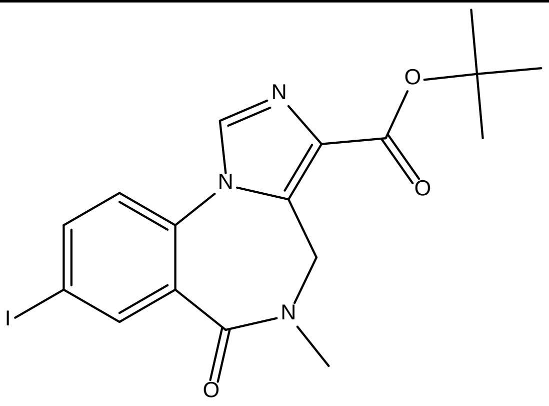 $C_{17}H_{18}IN_3O_3$	ZG-208	9.7	11.2	10.9		0.38	4.6
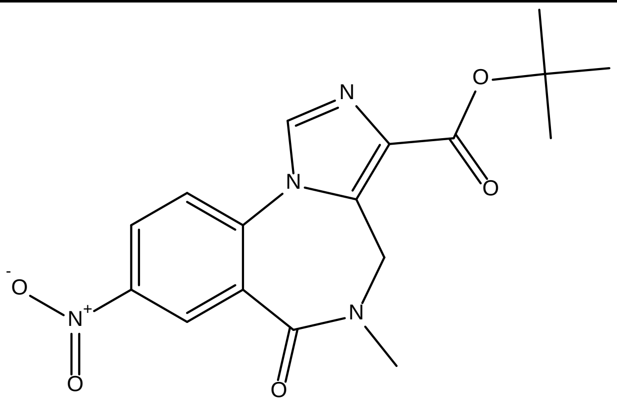 $C_{17}H_{18}N_4O_5$	ZG-213	12.8	49.8	30.2		3.5	22.5
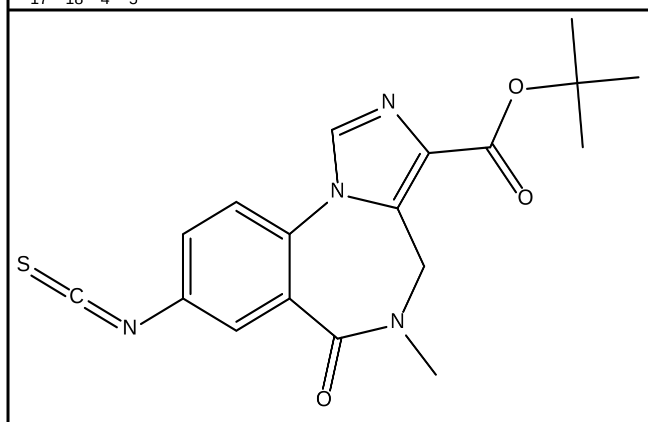 $C_{18}H_{18}N_4O_3S$	ZG-224	17.1	33.7	50		2.5	30.7

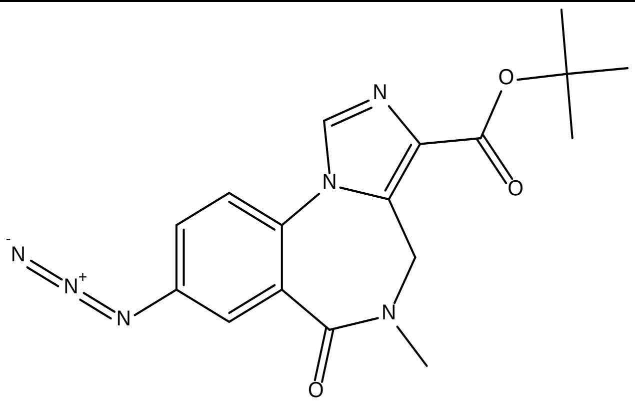 $C_{17}H_{18}N_6O_3$	ZG-234	7.25	22.14	9.84		0.3	5.25
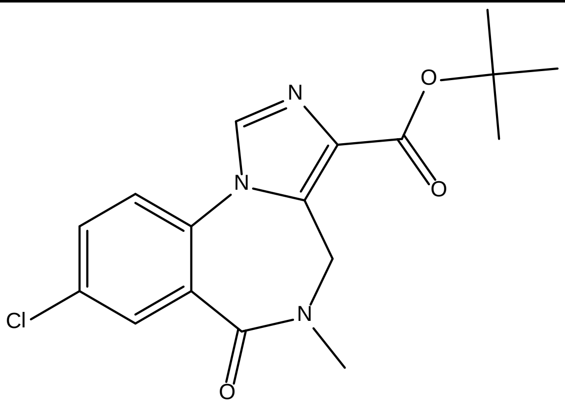 $C_{17}H_{18}ClN_3O_3$	ZG-63A	17.3	21.6	29.1		0.65	4
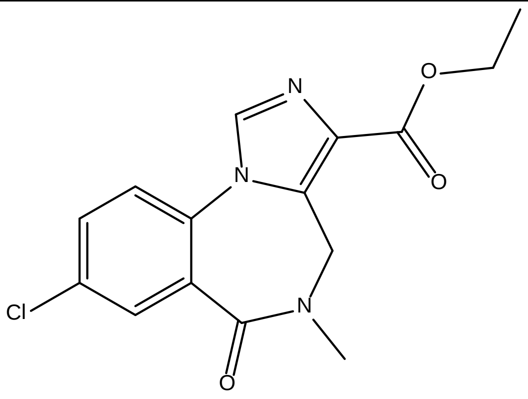 $C_{15}H_{14}ClN_3O_3$	ZG69A	6.8	16.3	9.2		0.85	54.6
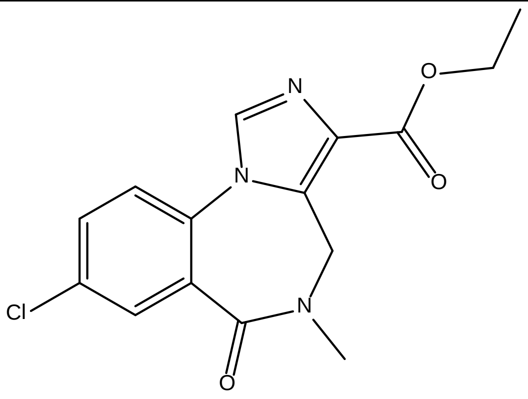 $C_{15}H_{14}ClN_3O_3$	ZG-69a(Ro15-1310)	6.8	16.3	9.2		0.85	54.6
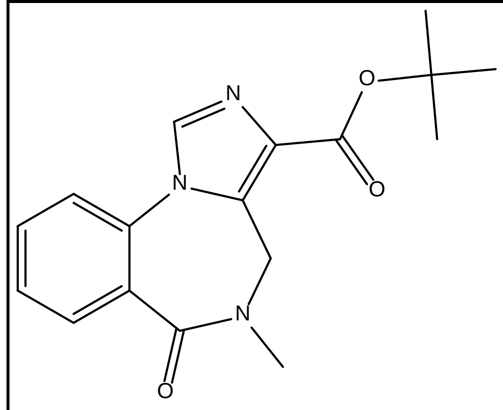 $C_{17}H_{19}N_3O_3$	ZG-88						
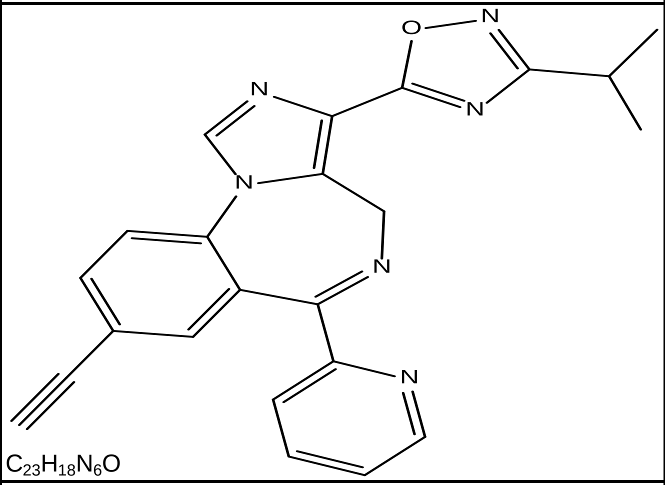 $C_{23}H_{18}N_6O$	ZJW-II-040						
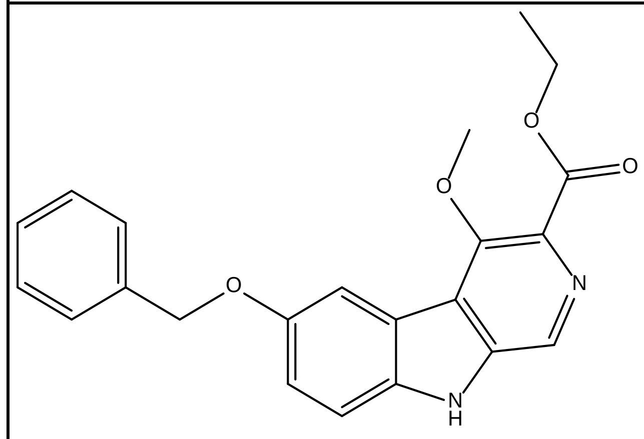 $C_{22}H_{20}N_2O_4$	ZK 93423	4.1	4.2	6		4.5	1000

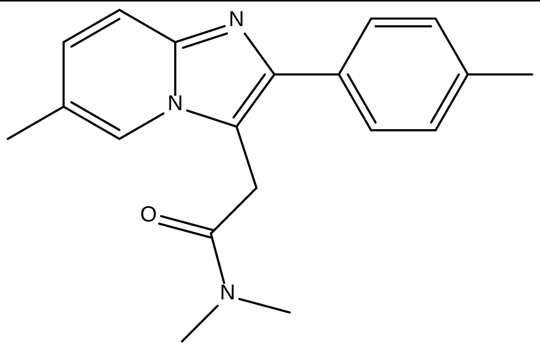 $C_{19}H_{21}N_3O$	ZOLPIDEM	26.7	156	383		10000	10000
